# *N*-to-*S* Acyl Transfer as an Enabling Strategy in Asymmetric and Chemoenzymatic Synthesis

**DOI:** 10.1021/jacsau.4c00257

**Published:** 2024-05-09

**Authors:** Woonkee S. Jo, Brian J. Curtis, Mohammad Rehan, Maria L. Adrover-Castellano, David H. Sherman, Alan R. Healy

**Affiliations:** † Chemistry Program, New York University Abu Dhabi (NYUAD), Saadiyat Island, Abu Dhabi 129188, United Arab Emirates (UAE); ‡ Life Sciences Institute, University of Michigan, 210 Washtenaw Avenue, Ann Arbor, MI 48109, USA; § Departments of Medicinal Chemistry, Chemistry, and Microbiology & Immunology, University of Michigan, Ann Arbor, MI 48109USA

**Keywords:** Chiral auxiliary, N-to-S acyl transfer, thioester, chemoenzymatic, native chemical ligation, noncanonical amino acids, SNAC

## Abstract

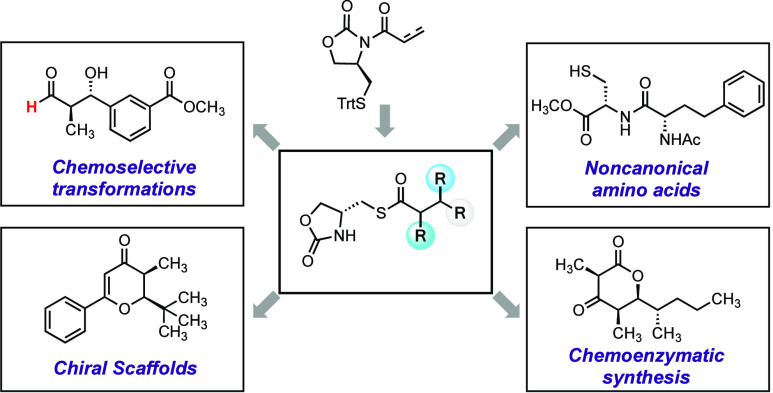

The observation of thioester-mediated acyl transfer processes in nature has inspired the development of novel protein synthesis and functionalization methodologies. The chemoselective transfer of an acyl group from *S*-to-*N* is the basis of several powerful ligation strategies. In this work, we sought to apply the reverse process, the transfer of an acyl group from *N*-to-*S*, as a method to convert stable chiral amides into more reactive thioesters. To this end, we developed a novel cysteine-derived oxazolidinone that serves as both a chiral imide auxiliary and an acyl transfer agent. This auxiliary combines the desirable features of rigid chiral imides as templates for asymmetric transformations with the synthetic applicability of thioesters. We demonstrate that the auxiliary can be applied in a range of highly selective asymmetric transformations. Subsequent intramolecular *N*-to-*S* acyl transfer of the chiral product and in situ trapping of the resulting thioester provides access to diverse carboxylic acid derivatives under mild conditions. The oxazolidinone thioester products can also be isolated and used in Pd-mediated transformations to furnish highly valuable chiral scaffolds, such as noncanonical amino acids, cyclic ketones, tetrahydropyrones, and dihydroquinolinones. Finally, we demonstrate that the oxazolidinone thioesters can also serve as a surrogate for SNAC-thioesters, enabling their seamless use as non-native substrates in biocatalytic transformations.

## Introduction

**Figure 1:**
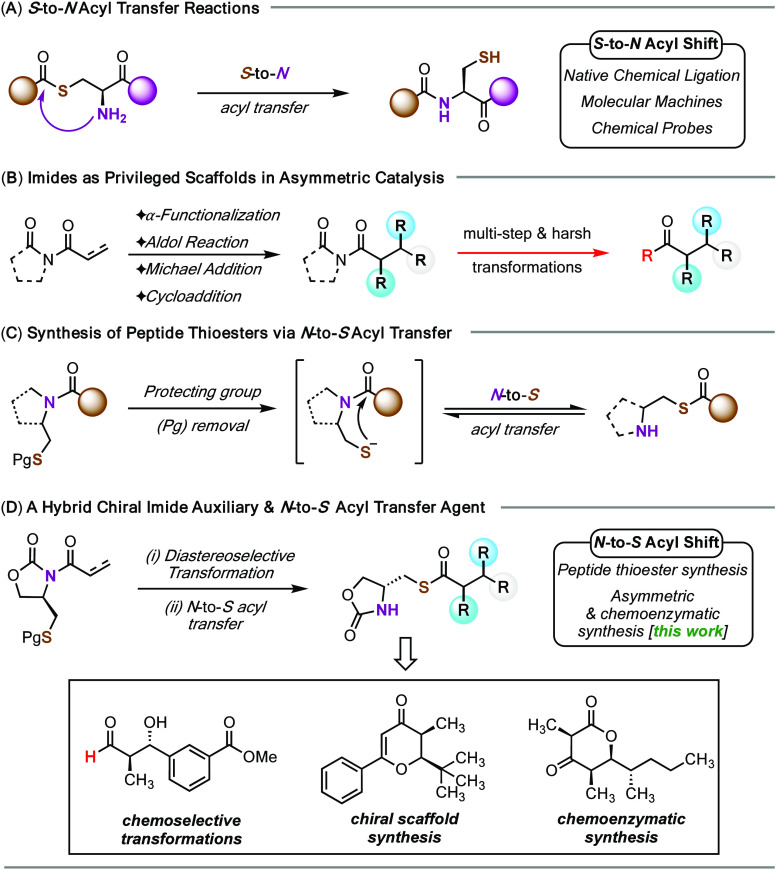
(A) Novel methodologies inspired by the *S*-to-*N* acyl transfer reaction. (B) The application of imide auxiliaries in asymmetric synthesis. (C) The application of *N*-to-*S* acyl transfer in the synthesis of peptide thioesters. (D) A cysteine-derived chiral auxiliary that unites the desirable features of an imide auxiliary with the downstream functionality of a thioester.

Thioester-mediated reactions are abundant in nature. In biosynthesis, thioesters serve as acyl donors in the thiotemplated synthesis of fatty acids, polyketides, and nonribosomal peptides.^1^ They also play a vital role as intermediates in several critical biological processes, such as intein-mediated protein splicing, protein ubiquitination, and transglutamination.^2^ In these processes, an acyl group undergoes an *S*-to-*N* transfer, enabling the efficient and chemoselective synthesis of amide bonds in a complex biological environment. New ligation methodologies that harness this selective *S*-to-*N* acyl transfer step have been developed and applied in protein synthesis and modification, and in the development of chemical probes and molecular machines (Figure 1A).^2^ The most notable of these methodologies is native chemical ligation (NCL), a process involving the coupling of a peptide containing a *C*-terminal thioester and a peptide containing an *N*-terminal cysteine.^3^ The reaction is initiated by an intermolecular transthioesterification to link the two peptide fragments together followed by a spontaneous intramolecular *S*-to-*N* acyl transfer to generate the peptide bond. The attractiveness of NCL lies in its ability to chemoselectively condense large peptide fragments under mild conditions.

Thioesters are also versatile precursors in the field of synthetic organic chemistry as they can be chemoselectively converted to diverse carboxylic acid derivatives under mild conditions.^4^ Moreover, they can participate in Pd-mediated reactions to generate ketones and aldehydes in one step under neutral conditions.^5^ Beyond traditional synthetic chemistry, thioesters have also been extensively used as substrates in enzymatic transformations.^6^ However, despite these attractive features, thioesters are not widely used as substrates in asymmetric synthesis. Although there have been some notable successes in the application of thioesters in asymmetric catalysis,^7^ particularly as substrates in the asymmetric aldol reaction,^8^ their lack of rigidity and higher reactivity has limited their potential as a general template for asymmetric reactions. In contrast, amides, and in particular imides, have been widely employed in asymmetric synthesis, as they provide a rigid template that can be activated through Lewis acid coordination, leading to high reactivity and excellent asymmetric induction. Chiral-nonracemic imides (such as oxazolidinones and imidazolidinones) have proven to be powerful mediators of asymmetric transformations, including α-functionalization of a carbonyl group, aldol addition, Michael addition, and cycloadditions (Figure 1B).^9^ Many of these methods have evolved from stoichiometric to catalytic using achiral imides in the presence of chiral catalysts.^10^ Although these auxiliaries have had tremendous success as templates in asymmetric catalysis, difficulties often arise when cleaving the chiral product from the auxiliary.^11,12^ A mild, chemoselective, and general method to convert the amide product to the respective chiral aldehyde, ketone, or carboxylic acid derivative remains elusive. These transformations often require harsh or nonstrategic synthetic steps and can be accompanied by unwanted side reactions or loss of stereochemical integrity.^13^ As a consequence, significant efforts have been dedicated to the identification of more readily functionalizable ester equivalents as substrates for asymmetric transformations.^14^

In this work, we sought to combine the attractive features of the imide auxiliaries for asymmetric transformations, with the downstream synthetic utility of a thioester group by employing an intramolecular *N*-to-*S* acyl transfer reaction. Similar to the *S*-to-*N* acyl transfer, the reverse reaction, the transfer of an acyl group from *N*-to-*S* to generate a thioester, is an important transformation in many biological processes, including the first step in intein-mediated protein splicing.^15^ However, this rearrangement is thermodynamically unfavorable and requires the scissile amide bond to be in a high-energy twisted conformation.^16^ While less common than *S*-to-*N* acyl transfer, a variety of *N*-to-*S* acyl transfer auxiliaries have been developed that mimic this process and are used in the formation of peptide thioesters (Figure 1C).^17^ These acyl transfer auxiliaries consist of a tertiary amide to aid distortion of the amide bond (ground-state destabilization), and a pendant thiol to enable an intramolecular proximity-driven *N*-to-*S* acyl transfer (Figure S1).^18^ The *N*-to-*S* acyl transfer reaction is typically reversible and, therefore, the transient thioester is either used in situ in a ligation reaction, or transthioesterified with excess thiol to form a stable *C*-terminal peptide thioester.^19^

We reasoned that an intramolecular *N*-to-*S* acyl transfer could provide a mild and chemoselective strategy to convert the chiral imide product to the more reactive and synthetically tractable thioesters. We chose to investigate this hypothesis using a cysteine-derived oxazolidinone chiral auxiliary (Figure 1D). Oxazolidinones have been extensively used as chiral auxiliaries in asymmetric transformation,^20^ and also have previously been shown to be suitable auxiliaries for the *N*-to-*S* acyl transfer reaction.^21^ The exocyclic amide bond of *N*-acyloxazolidinone derivatives is twisted out of planarity, resulting in ground-state destabilization.^22^ We hypothesized that on completion of the chiral auxiliary-mediated stereoselective transformation, deprotection of the proximal thiol group would result in an *N*-to-*S* acyl transfer to generate a chiral thioester product that could be further derivatized by synthetic or chemoenzymatic methods.

## Results and Discussion

### Auxiliary Development

**Figure 2:**
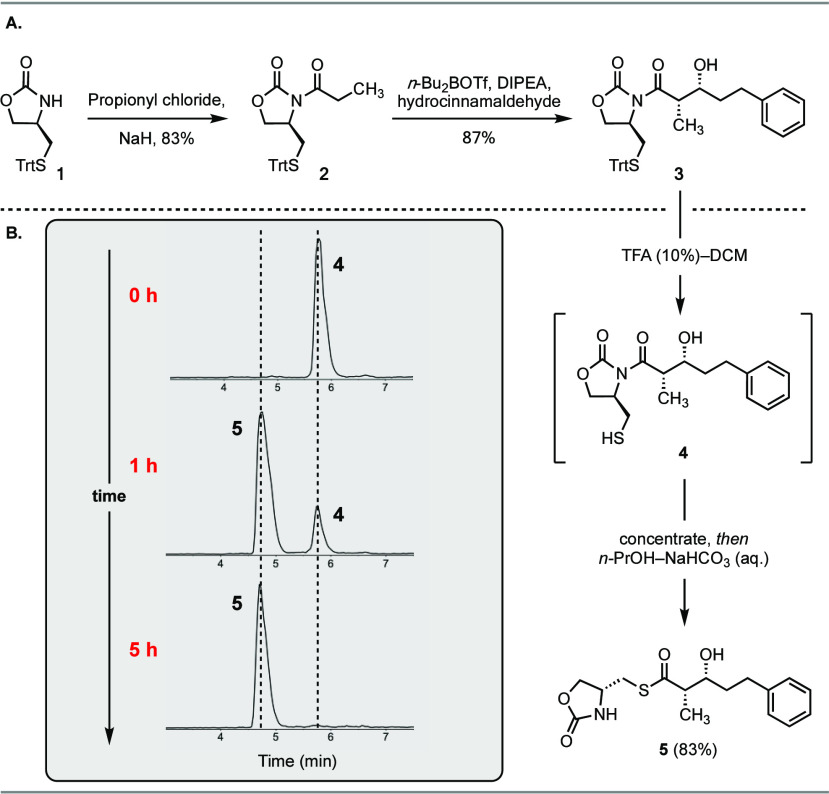
(A) Synthesis of *syn*-aldol product **3**. (B) Mass-selective (*m*/*z* = 346.11) LC-MS analysis of the conversion of **4** to **5** at 0 (top), 1 (middle), and 5 h (bottom).

We chose the readily accessible cysteine-derived oxazolidinone **1** to test our proposed strategy. The trityl protecting group was selected due to its facile and efficient removal under mildly acidic conditions. Additionally, we expected that upon coordination of a Lewis acid by oxazolidinone **1**, the sterically hindered trityl group would create a well-defined chiral environment, leading to enhanced selectivity. An efficient and scalable 3-step synthesis of *S*-trityl oxazolidinone **1** was achieved from commercially available l-cysteine (Scheme S1). This sequence only requires one column purification and has provided >200 g of **1** to date. We opted for the boron-mediated propionate aldol as a model reaction to investigate the efficiency of our thiol-based oxazolidinone auxiliary. *N*-Acylation of auxiliary **1** with propionyl chloride yielded *N*-acyloxazolidinone **2**. Subsequent condensation of **2** with hydrocinnamaldehyde under standard boron aldol conditions provided the desired Evans *syn*-aldol product **3** in high yield and stereoselectivity (Figure 2A).^23^ Exposure of **3** to 10% trifluoroacetic acid (TFA) in dichloromethane rapidly removed (30 min) the *S*-trityl group to quantitatively provide the free thiol **4**.^24^ Solvent removal and subsequent subjection of the crude thiol **4** to a mildly basic *n*-propanol solution smoothly effected the desired *N*-to-*S* acyl transfer to afford the thioester **5** in 83% isolated yield (Figure 2B). The acyl transfer reaction could be readily monitored by LC-MS, showing the complete conversion within 5 h.

### Chemoselective Auxiliary Cleavage

**Scheme 1:**
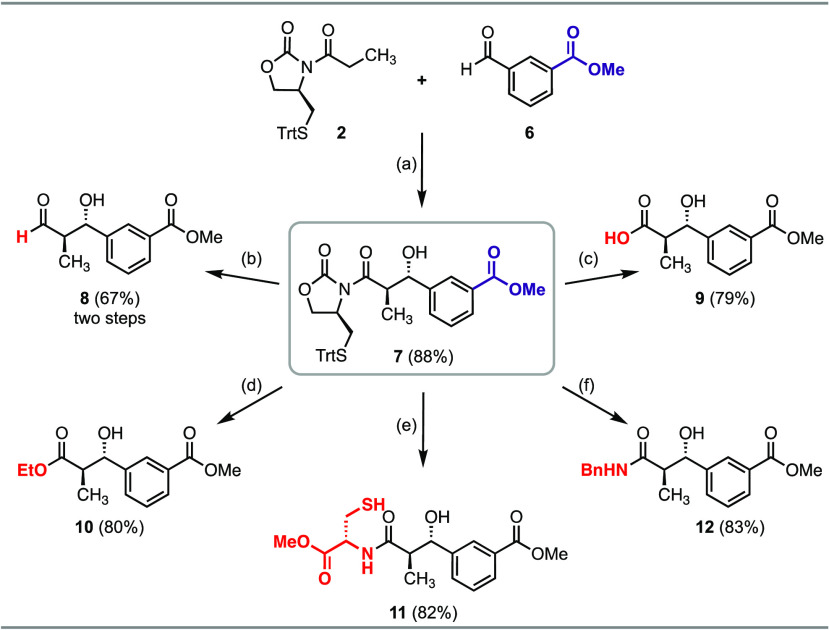
Synthesis and Derivatization of the *anti*-Aldol Product **7** Reagents and conditions: (a) MgCl_2_, trimethylsilyl chloride (TMSCl), Et_3_N, EtOAc, 23 °C; *then* TFA, MeOH, 23 °C, 88%; (b) (i) TFA, Et_3_SiH, CH_2_Cl_2_, *then*^*n*^PrOH-NaHCO_3_, 23 °C, 93%; (ii) Pd(OAc)_2_, Et_3_SiH, MgSO_4_, acetone, 23 °C, 72%; (c) TFA, Et_3_SiH, CH_2_Cl_2_, *then* THF-NaHCO_3_, 60 °C, 79%; (d) TFA, Et_3_SiH, CH_2_Cl_2_, *then* EtOH-NaHCO_3_, 60 °C, 80%; (e) TFA, Et_3_SiH, CH_2_Cl_2_, *then* DMF-NaHCO_3_, l-cysteine methyl ester, DL-dithiothreitol (DTT), 23 °C, 82%; (f) TFA, Et_3_SiH, CH_2_Cl_2_, *then* Et_2_O-Et_3_N, benzylamine, silver trifluoroacetate (AgTFA), 23 °C, 83%.

In addition to the Evans *syn*-aldol product **3**, we also accessed the non-Evans *anti*-aldol product **7** in high yield and selectivity using a magnesium-halide-catalyzed aldol (Scheme 1).^25^ A notable feature of this example is the use of the methyl-3-formylbenzoate **6** as the electrophile. The most common methods to cleave oxazolidinone auxiliaries are LiBH_4_-mediated reduction to the corresponding alcohol,^26^ or hydrolysis to the carboxylic acid using LiOOH.^27^ The resulting carboxylic acid can be converted to carboxylic acid derivatives using stochiometric coupling reagents. Alternatively, the alcohol can be oxidized to the aldehyde for subsequent carbon–carbon bond-forming transformations. A limitation of this approach is that substrates containing electrophilic functional groups (such as the ester in **6**) are typically not compatible with the conditions used to cleave the oxazolidinone auxiliary. This limitation can be overcome using the cysteine-derived auxiliary **1**, as the *N*-to-*S* transfer generates a more electrophilic thioester that can be chemoselectively functionalized in the presence of the ester (Scheme 1). To demonstrate this, we subjected the *anti*-aldol product **7** to the *N*-to-*S* acyl transfer conditions to form the corresponding oxazolidinone thioester. Subsequent Fukuyama reduction chemoselectively converted the thioester to the aldehyde **8**.^28^ We next investigated if it was possible to directly trap the thioester in situ with a range of nucleophiles. Indeed, changing the solvent to tetrahydrofuran (THF) and heating the acyl transfer step at 60 °C resulted in hydrolysis of the thioester intermediate to directly afford the carboxylic acid **9** in 79% yield. Changing the solvent to ethanol and heating at 60 °C effected the *N*-to-*S* transfer and subsequent esterification to yield the diester **10**. The thioester could also be trapped in situ with cysteine methyl ester in a native chemical ligation reaction to furnish **11** in 82% yield.

Finally, we sought to directly form an amide from the intermediate thioester using silver trifluoroacetate (AgTFA) as a thiophilic Lewis acid.^29^ To achieve this, nonaqueous conditions for the *N*-to-*S* acyl transfer were required. Initial screening revealed that the saturated aqueous sodium bicarbonate solution could be replaced with excess triethylamine (5–10 equiv). We subsequently discovered that complete *N*-to-*S* transfer is observed in 4 h in a wide variety of solvents (Table S1). Undertaking the acyl transfer reaction in diethyl ether–triethylamine generated the thioester intermediate that was trapped in situ by the addition of benzylamine and AgTFA to afford amide **12** in 83% yield. Importantly, all these transformations proved to be chemoselective with no cleavage of the methyl ester observed. Furthermore, this approach provided direct access to the corresponding chiral aldehyde, ester, and amide without the need for additional redox/protecting group manipulations or coupling synthetic steps.

### Noncanonical Amino Acid Synthesis

Outside of the 20 proteinogenic l-amino acids that serve as the primary protein building blocks are countless noncanonical amino acids (ncAAs), which contain atypical side chains, backbone connectivity, or stereochemistry (d). The distinct chemical and biological properties that ncAAs impart to natural products, peptide therapeutics, and unnatural proteins has elicited considerable interest.^30^ As a result, a general and efficient method for the synthesis and subsequent utilization of ncAAs would be desirable within both synthetic chemistry and biology.

β-Hydroxy, homo-, and d-amino acids are among the most common ncAAs in biologically active natural products and pharmaceuticals. Incorporation of these ncAAs into peptides can confer resistance to enzymatic degradation or alter the stability or activity of the peptide.^31^ However, these unnatural amino acids are typically expensive and only available in small quantities. A general method to access natural and unnatural α-amino acids was previously reported using Evans oxazolidinone.^32^ Using this strategy, we sought to synthesize l- and d-homophenylalanine (HPA), an unnatural isomer of phenylalanine containing an extra methylene at the α-position. Both isomers of homophenylalanine are essential building blocks of many bioactive small molecules and drugs, including angiotensin converting enzyme (ACE) inhibitors, proteasome inhibitors, acetylcholinesterase inhibitors, and caspase inhibitors.^33,34^

**Scheme 2:**
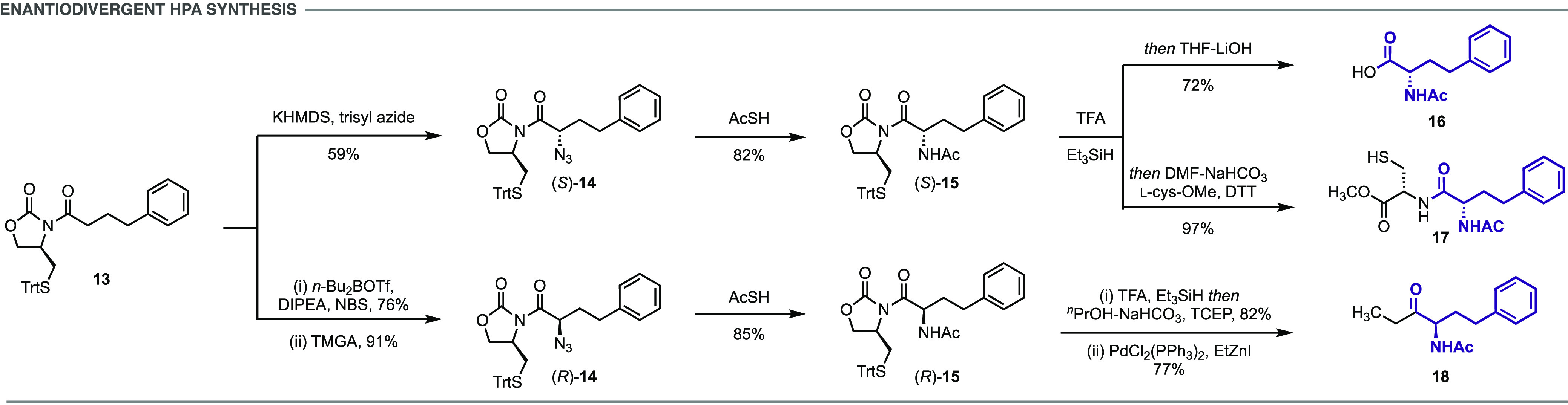
Synthesis and Derivatization of l- and d-Homophenylalanine; See the Supplementary Materials for Experimental Details

The *N*-acyloxazolidinone **13** was synthesized from readily available and inexpensive 4-phenylbutyric acid (see Supporting Information). A direct azide transfer to the enolate of carboxamide **13** was used to access the (*S*)-α-azidocarboximide **14** (Scheme 2). This process involved the treatment of the potassium enolate of **13** with trisyl azide at −78 °C followed by quenching with glacial acetic acid to yield (*S*)-**14** in 59% yield. The (*R*)-α-azidocarboximide **14** was synthesized by a two-step α-bromination/azidation sequence. Reaction of *N*-bromosuccinimide (NBS) with the dibutylboryl enolate derived from **13** afforded the corresponding α-brominated product. Treatment with tetramethylguanidinium azide (TMGA) provided the diastereomerically pure (*R*)-**14** in 68% isolated yield over the two steps.

The α-azidocarboximides are versatile intermediates as both the *N*- and *C*- termini can be orthogonally derivatized. Ligation of the azide with a thioacid provides direct access to amides without requiring prior reduction to the amine, or the need for activating or coupling agents. Stirring either (*S*)- or (*R*)-**14** in thioacetic acid at room temperature afforded the respective *N*-acetyl derivatives **15**.^35^*N*-to-*S* acyl transfer of (*S*)-**15** and in situ hydrolysis provided *N*-acetyl-l-homophenylalanine **16** (Scheme 2). Alternatively, *N*-to-*S* acyl transfer and in situ trapping with cysteine methyl ester provided the *N*-protected dipeptide **17**. This sequence involves the sequential application of three unique acyl transfer reactions, an *N*-to-*S* transfer to form the thioester of **15**, an *S*-to-*S* reaction to link the two amino acids, and an *S*-to-*N* transfer to yield the dipeptide.

In addition to their application in peptide synthesis, the HPA-auxiliary adducts provide a powerful entry point to chiral α-amino ketones. α-Amino ketones are present in numerous important natural products and pharmaceutical agents, and also serve as versatile building blocks for the synthesis of polyfunctional amino derivatives.^36^*N*-to-*S* acyl transfer of (*R*)-**15**, followed by Fukuyama coupling of the resulting thioester with ethylzinc iodide yielded the α-amino ketone **18** in 77% yield.^37^

### Synthesis of Chiral Scaffolds

**Scheme 3:**
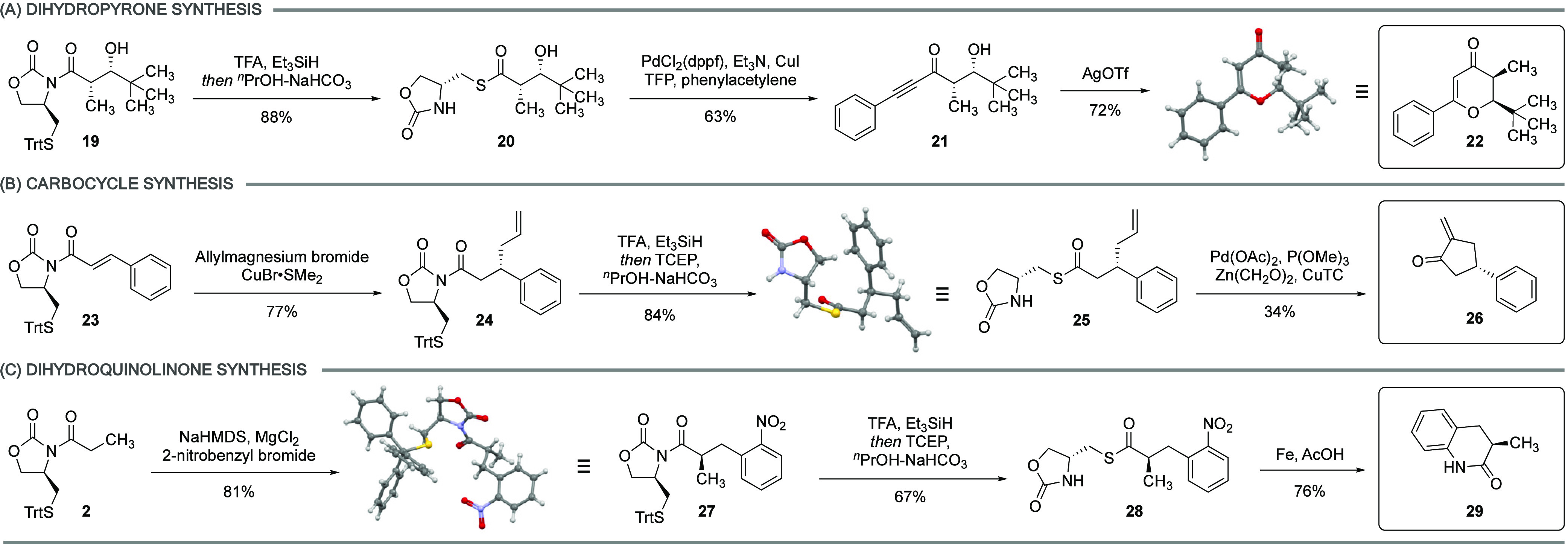
Synthesis of Dihydropyrone **22** (A), Cyclic Ketone **26** (B), and Dihydroquinolinone **29** (C); See the Supplementary Materials for Experimental Details; Red, Oxygen; Blue, Nitrogen; Gray, Carbon; Yellow, Sulfur; White, Hydrogen

Chiral auxiliary mediated synthesis has empowered the modern synthetic chemist with a rich arsenal of methods to rapidly assemble stereodefined acyclic molecules. However, the conversion of these linear precursors to complex cyclic motifs still poses many challenges. A notable advantage of using thioesters as carboxylic acid equivalents is their participation in mild Pd-mediated transformations with diverse coupling partners.^5^ We sought to investigate if the thioester intermediate formed after *N*-to-*S* acyl transfer could provide an expedient entry to a number of stereodefined cyclic scaffolds common in natural products and pharmaceutical agents. *N*-to-*S* acyl transfer of the *syn*-aldol product **19** and subsequent coupling with phenylacetylene yielded the chiral ynone **21**.^38^ A silver-mediated intramolecular oxy-Michael reaction directly afforded tetrahydropyrone **22** (Scheme 3A).^39^ To demonstrate the utility of the auxiliary beyond the aldol reaction, we subjected the unsaturated imide **23** to Michael addition with an allyl Grignard.^40^ The β-allyl product **24** was obtained as a single diastereomer in 77% yield. *N*-to-*S* transfer followed by intramolecular Pd-mediated carbocyclization provided the chiral enone **26** (Scheme 3B).^41^ Finally, alkylation of **2** with 2-nitrobenzyl bromide provided **27** in 81% yield.^42^ The thioester obtained after *N*-to-*S* transfer was treated with Fe/AcOH to effect nitro reduction and intramolecular amidation to directly yield the dihydroquinolinone **29** in one step (Scheme 3C).^43^

### Application in Chemoenzymatic Synthesis

**Figure 3:**
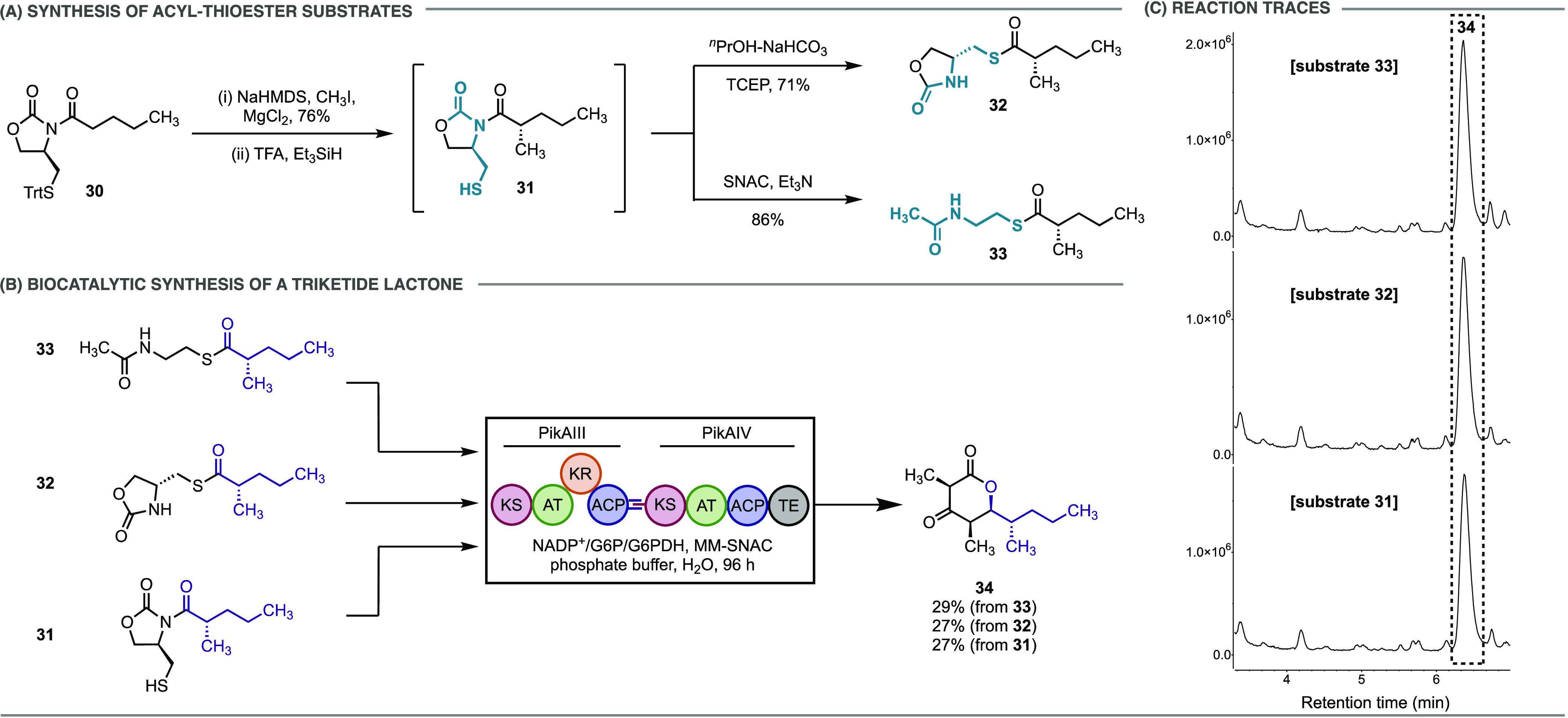
Chemoenzymatic synthesis of triketide lactone **34**. (A) Synthesis of acyl-thioester feeding substrates (**32** and **33**); see the Supporting Information for experimental details. (B) Formation of triketide lactone **34** catalyzed by PKS modules (PikAIII/PikAIV) from the pikromycin (Pik) biosynthetic pathway; see the Supporting Information for experimental details. (C) LC/MS traces (ESI^+^) showing the production of **34** from all three feeding substrates (**31**–**33**).

Biosynthetic pathways composed of polyketide synthases (PKSs), fatty acid synthases (FASs), and nonribosomal peptide synthetases (NRPSs) rely on thioester electrophiles as substrates for a range of chemical transformations.^6^ Typically, carboxylic acid building blocks are converted to activated acyl carrier protein (ACP) or acyl-coenzyme A (acyl-CoA) thioester substrates. Simpler thioester substrates, such as those containing the truncated CoA analogue, *N*-acetylcysteamine (SNAC), have found extensive use as model substrates to probe these biosynthetic pathways, which avoid the requirement for stoichiometric protein-bound substrates or expensive CoA-activated thioesters.^44^ A chiral auxiliary mediated synthetic approach remains the most common and reliable method to access polyketide intermediates, where the SNAC thioester is produced via coupling to the carboxylic acid that results from hydrolysis of the oxazolidinone-bound polyketide substrate.^45^ However, in many cases, this two-step strategy provides the desired SNAC thioester in low yield.^6^ We proposed that our auxiliary would provide facile access to SNAC thioester substrates by a one-pot *N*-to-*S* transfer and in situ transthioesterification with SNAC. Furthermore, due to the high structural similarity between the oxazolidinone thioester and SNAC-thioesters, we hypothesized it might be possible to directly use the oxazolidinone thioester as a substrate for enzymatic transformations. To test these hypotheses, we investigated the feasibility of non-native polyketide substrates (**32** and **33**) to be chain-extended and cyclized to a 6-membered triketide lactone by the last two PKS modules (PikAIII/PikAIV) from the pikromycin (Pik) biosynthetic pathway.^46^ Synthesis of the thioester substrates was initiated by α-methylation of **30** (Figure 3A).^42^ Removal of the trityl protecting group and subjecting the crude thiol **31** to a ^*n*^PrOH-NaHCO_3_ solution yielded oxazolidinone thioester **32**. Alternatively, in situ transthioesterification of the intermediate thiol **31** with SNAC directly furnished the SNAC-thioester **33** in 86% yield without the need for stoichiometric coupling reagents. Applying previously developed conditions involving purified PikAIII/PikAIV modules and methylmalonyl (MM)-SNAC as an extender unit,^44a,44b^ both substrates were evaluated for conversion to triketide lactone **34** (Figure 3B). Reactions involving 10 mg of both thioester substrates required 72–96 h for full substrate consumption as determined by LC-MS (Figure 3C). The formation of triketide lactone **34** was observed in comparable amounts for both substrates, where **34** was isolated in 29% yield from SNAC thioester **33** and 27% yield from oxazolidinone thioester **32**. Throughout the reaction course, we observed transthioesterification of oxazolidinone thioester **32** to SNAC-thioester **33**, where excess SNAC is derived from the MM-SNAC extender unit (Figure S2).^47^

Having demonstrated that the oxazolidinone thioester **32** was a viable thioester surrogate for biocatalysis, we sought to investigate if thiol **31** could also be processed by PikAIII/PikAIV. An initial investigation demonstrated that thiol **31** completely rearranged to the oxazolidinone thioester **32** in reaction phosphate buffer (pH 7.2, Scheme S2).^21,48^ Indeed, subjecting crude or purified thiol **31** to the enzymatic transformations produced lactone **34** in 25% and 27% yield, respectively, equal to that from oxazolidinone thioester **32**. In addition to exploring the feasibility of utilizing oxazolidinone thioesters as substrates in PKS biocatalysis, we wanted to investigate if the cyclization to form triketide lactone **34** is an enzyme-mediated or spontaneous process. Earlier studies have shown similar 6-membered ring lactones being formed spontaneously in the presence or absence of a thioesterase (TE).^45c,46b,46c,49^ We found that efficient formation of **34** requires the Pik TE terminal domain in PikAIV for cyclization,^50,51^ as the PikAIII/PikAIV bearing an inactive TE (S148A) variant produces only small amounts of **34** (Figure S3). Our chemoenzymatic approach illustrates the viability of employing non-native oxazolidinone thioester-containing substrates for biocatalytic transformations. The utilization of these short-chain substrates will facilitate future enzymatic explorations involving additional functional groups, as well as varying chain lengths, to produce a range of diverse products with varied architectures and ring sizes.^52,53^

## Conclusions

Numerous powerful ligation methodologies have been developed that employ a chemoselective *S*-to-*N* acyl transfer reaction. In contrast, the antipodal *N*-to-*S* acyl transfer reaction has been underutilized. In this work, we demonstrate that an intramolecular *N*-to-*S* acyl transfer can provide a powerful method to convert amide derivatives to versatile thioesters.

We report the application of a cysteine-derived oxazolidinone chiral auxiliary in a wide range of asymmetric transformations: the aldol reaction, Michael addition, α-alkylation, bromination, and azidation.^54^ The high yields and selectivity observed in the auxiliary **1**-mediated transformations and the relative ease of synthesis of the auxiliary make it an attractive alternative to traditional oxazolidinone auxiliaries. The mild conditions for both the acyl transfer reaction and subsequent transformation of the thioester products are compatible with a range of reactive functional groups. The thioester products were either converted in situ to diverse carboxylic acid derivatives or subjected to Pd-mediated cross-coupling reactions to provide efficient access to several biologically relevant chiral motifs and cyclic scaffolds. Importantly, these transformations did not require the use of protecting groups, coupling reagents, or nonstrategic functional group manipulation steps. Finally, we demonstrated that this strategy provided ready access to competent thioester probes for enzymatic transformations. Thiol **31**, obtained after a facile trityl deprotection, was converted to the 6-membered triketide lactone **34** by the action of two PKS modules from the pikromycin biosynthetic pathway, thereby seamlessly linking traditional polyketide synthetic strategies to emerging biocatalytic processes.

Beyond the potential broad application of this auxiliary in asymmetric and chemoenzymatic synthesis, we propose that the acyl transfer strategy outlined herein could provide a powerful method to couple the benefits of amides in asymmetric transformations with the downstream utility of thioesters. This strategy could also be extended to include the development of other thiol-functionalized chiral auxiliaries, and achiral *N*-to-*S*, or *N*-to-*O* acyl transfer agents for the development of catalytic methods.^55^

## Supporting Information Available

Schemes, figures, and tables; detailed experimental procedures; characterization data for all new compounds (PDF)

## Accession Codes

X-ray data for compounds **22**, **25**, **27**, **29**, and **S5** are freely available at the Cambridge Crystallographic Data Centre under deposition numbers 2287271, 2286532, 2286531, 2286539, and 2286538, respectively. These data can be obtained free of charge via www.ccdc.cam.ac.uk/data_request/cif, or by emailing data_request@ccdc.cam.ac.uk, or by contacting The Cambridge Crystallographic Data Centre, 12 Union Road, Cambridge CB2 1EZ, UK; fax: +44 1223 336033.

## ABBREVIATIONS

NCL: native chemical ligation
PKS: polyketide synthase
SNAC: *N*-acetylcysteamine
TE: thioesterase
