# Supplementary material for: N-to-S Acyl Transfer as an Enabling Strategy in Asymmetric and Chemoenzymatic Synthesis
Source: JACS Au. 2024 May 9;4(5):2058–66. doi: 10.1021/jacsau.4c00257 (PMC11134368; doi:10.1021/jacsau.4c00257)
Supplement: Supplementary file 1 — au4c00257_si_001.pdf [file au4c00257_si_001.pdf]

Supplementary Materials for

# *N*-to-*S* acyl transfer as an enabling strategy in asymmetric and chemoenzymatic synthesis

Woonkee S. Jo,[a] Brian J. Curtis,[b] Mohammad Rehan,[a] Maria L. Adrover-Castellano,[b] David H. Sherman*,[b,c], Alan R. Healy*,[a]

[a]Chemistry Program, New York University Abu Dhabi, Abu Dhabi, 129188, United Arab Emirates
[b]Life Sciences Institute, University of Michigan, 210 Washtenaw Avenue, Ann Arbor, MI 48109-2216 (USA)
[c]Departments of Medicinal Chemistry, Chemistry, and Microbiology & Immunology, University of Michigan (USA)

## Table of Contents

**Supplementary Schemes** ........ 1
**Supplementary Figures and Tables** ........ 7
**General Experimental Methods** ........ 15
**General Experimental Procedures** ........ 16
**Materials** ........ 17
**Instrumentation** ........ 17
**Synthetic Procedures** ........ 19
**Catalog of nuclear magnetic resonance spectra** ........ 67
**Catalog of X-ray data** ........ 153
**References** ........ 183

**Supplementary Schemes.**

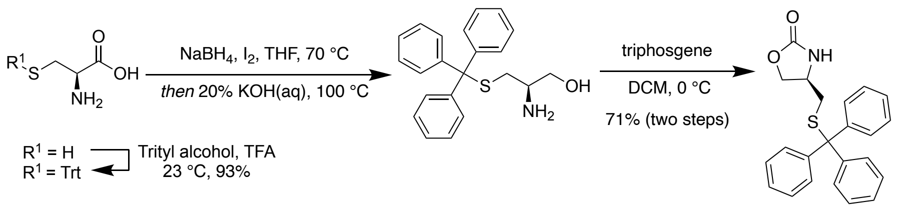


**S1** **1**

**Scheme S1. Synthesis of *S*-trityl oxazolidinone.** Auxiliary **1** is synthesized in 3 steps from L-cysteine ($1.4/g; Sigma-Aldrich), or 2 steps from *S*-trityl-L-cysteine ($21/g; Sigma-Aldrich). *S*-trityl-L-cysteine can be synthesized by acid-catalyzed tritylation of L-cysteine.[1] Reduction of the carboxylic acid followed by triphosgene mediated cyclization of the resulting amino alcohol **S1** yields oxazolidinone **1**.[2, 3] The synthetic route is scalable (>20 g prepared in one batch), involves only one column purification step, and has provided >200g of **1** to date. See the synthetic procedures for experimental details.

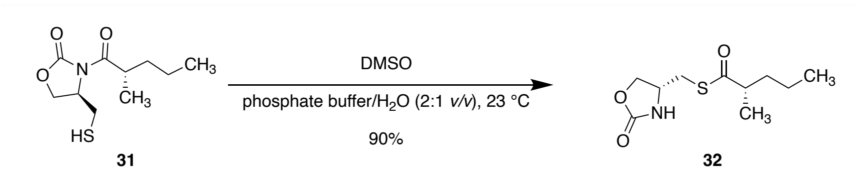


**Scheme S2. Synthesis of the oxazolidinone thioester 32.**

In a 50 mL falcon tube, a solution of free thiol **31** (5.00 mg, 22.0 µmol, 1 equiv) in dimethylsulfoxide (0.43 mL) were added water (11 mL) and phosphate buffer (5.4 mL) at 23 °C. The reaction mixture was incubated for 24 h at 23 °C after thorough mixing. The product mixture was extracted with dichloromethane (3 × 30 mL), dried over sodium sulphate and filtered. The filtrate was concentrated, and the residual dimethylsulfoxide was removed under a constant stream of nitrogen to afford the *oxazolidinone thioester* **32** (4.50 mg, 90%). The full analytical data is found on p. 61.

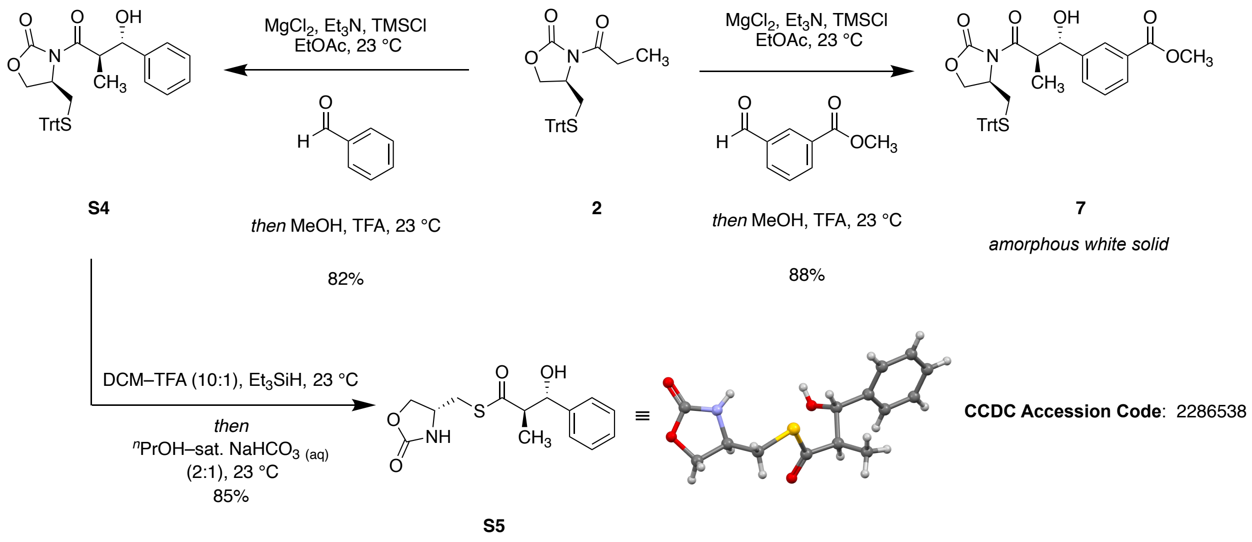


**Scheme S3. Proof of stereochemistry for the *anti*-aldol product 7.** Proving the absolute *anti*-stereochemistry of the aldol product involved the preparation of analogue **S4** followed by the *N*-to-*S* acyl transfer to deliver thioester **S5**. Crystals suitable for X-ray crystallography were obtained by slow evaporation from deuterated chloroform. The crystal structure of **S5** unambiguously proved the *anti*-stereochemistry that is consistent with the literature.[4] The stereochemistry of **7** was assumed to be same as **S5** by analogy.

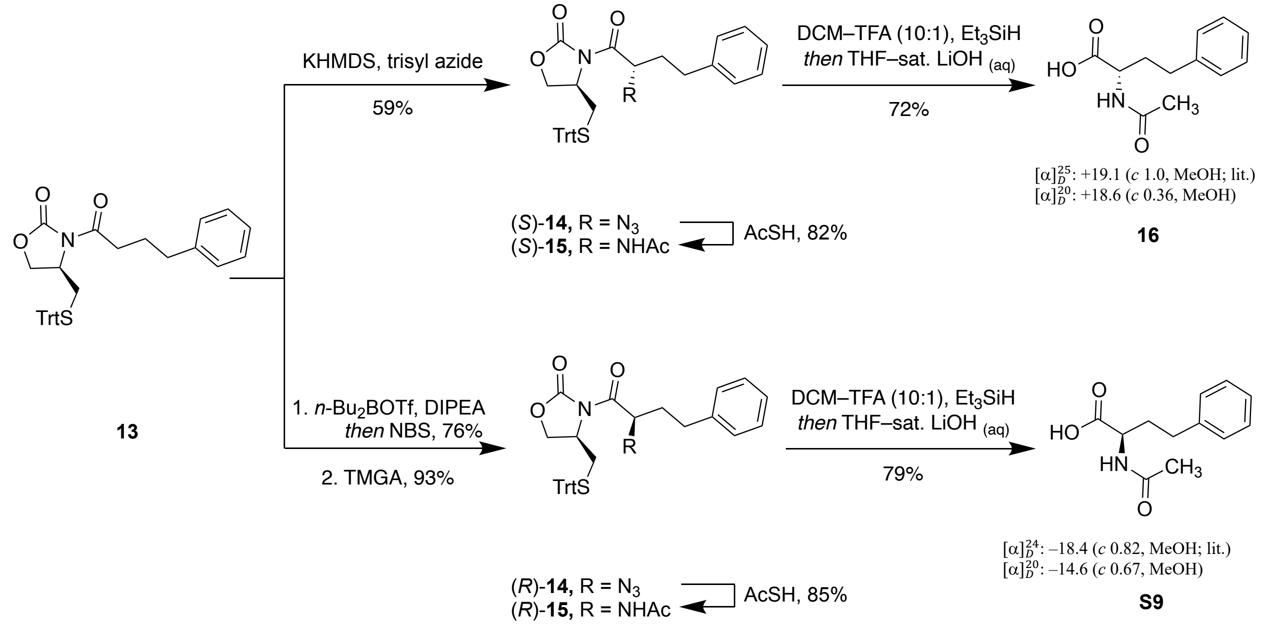


**Scheme S4. Proof of stereochemistry for the α-azido compounds (*R*) and (*S*)-14**. The absolute stereochemistry was proven by interconversion of **14** (both diastereomers) to known compounds **16** and **S9** by reductive acylation and subsequent hydrolysis. The specific optical rotation was measured for both enantiomers and compared with the literature.[5]

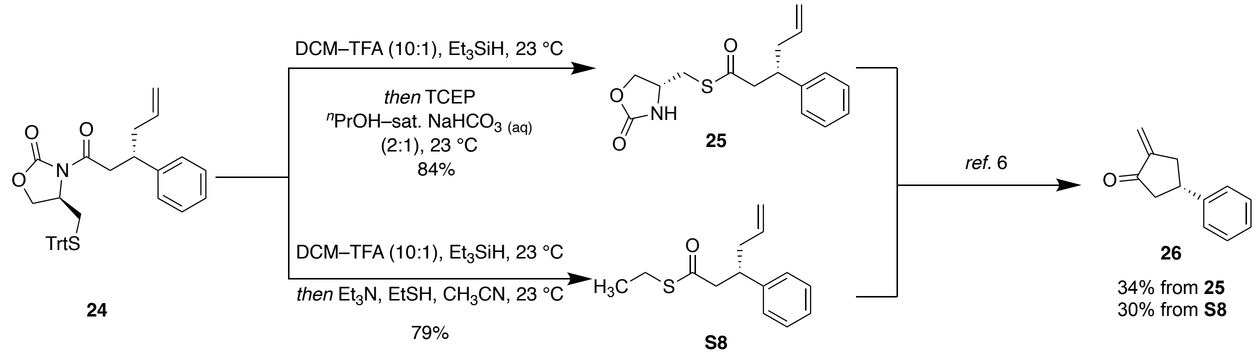


**Scheme S5. Synthesis of carbocycle 26.** Cyclic ketone **26** was obtained in 34% yield from thioester **25** under the literature conditions.[6] Attempts to improve the yield by modifying the reaction conditions (e.g., alternative workup/purification or number of equivalents of reagents) did not noticeably improve the yield. To identify if the oxazolidinone thioester was interfering with the reaction, we converted **24** to the known ethyl thieoster **S8**.[6] This was achieved by *N*-to-*S* acyl transfer and *in situ* transthioesterification with ethanethiol (see synthetic procedures for experimental details). Subjecting **S8** to the literature conditions provided **26** in 30% yield, indicating that the thioester was not the limiting factor. All attempts (in our hands) to replicate the reported yield for this transformation (76%) were unsuccesful.[6] Deviation from the standard conditions also failed to yield **26** in improved yields.[7, 8]

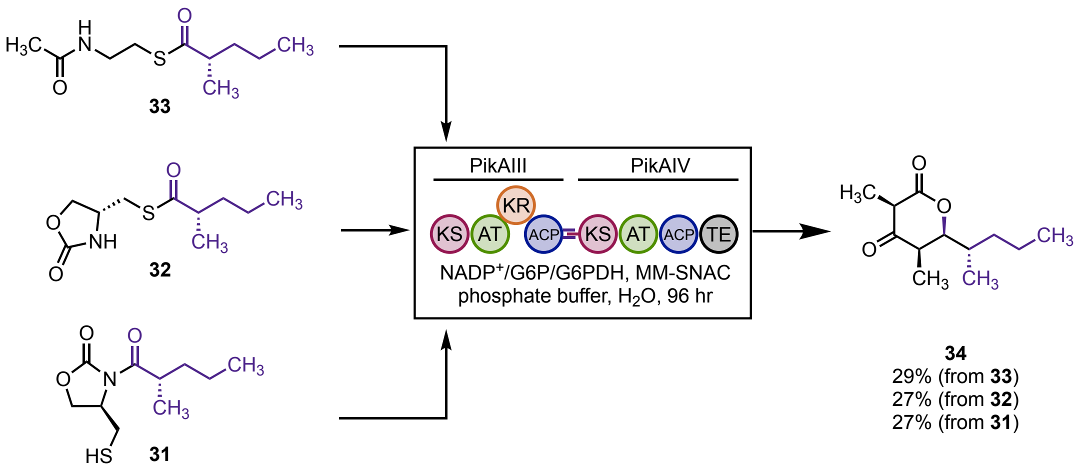


**Scheme S6. Chemoenzymatic synthesis of triketide lactone 34.** To assess reactivities of SNAC thioester **33**, oxazolidinone thioester **32**, and free thiol **31**, analytical scale reactions were performed using purified PikAIII and PikAIV-TE proteins (PikAIV bearing wild-type TE domain or the TE S148A variant). For a 50 µL reaction experiment, water, phosphate buffer (200 mM), nicotinamide adenine dinucleotide phosphate ($NADP^+$, 0.5 mM), glucose-6-phosphate (G6P, 10 mM), and glucose-6-phosphate dehydrogenase (G6PDH, 2 mU/µL) were added to a PCR tube. After 10 min, methylmalonyl *N*-acetylcysteamine (MM-SNAC, 20 mM), PikAIII (3 µM), PikAIV (3 µM) and substrate in dimethylsulfoxide (1 mM) were added. Following thorough mixing, each reaction was incubated at 23 °C for the appropriate period of time. Reactions were quenched by the addition methanol (150 µL) and clarified by centrifugation (10,000 × g, 10 min, 4 °C). Supernatants were analyzed by QTOF-LC-MS using 2 µL injections. QTOF-LC-MS conditions were as follows: mobile phase (A = deionized water + 0.1% formic acid, B = 95% acetonitrile/deionized water + 0.1% formic acid); 10% B for 1.0 min, 10% to 80% B over 7.0 min, 80% B for 2.0 min; flow rate = 0.4 mL/min.

To assess product conversion over time, the reactions were scaled based on the number of time points. For example, a 48 h experiment with two time points (24 h and 48 h) was performed on a 100 µL scale. After 24 h, 50 µL of the reaction was transferred to a separate PCR tube while the remaining was incubated for another 24 h. The reactions were quenched with 150 µL of MeOH, centrifuged, and analyzed by QTOF-LC-MS as above. See the synthetic procedures for the semi-preparative synthesis and characterization of **34**.

**Supplementary Figures and Tables**.

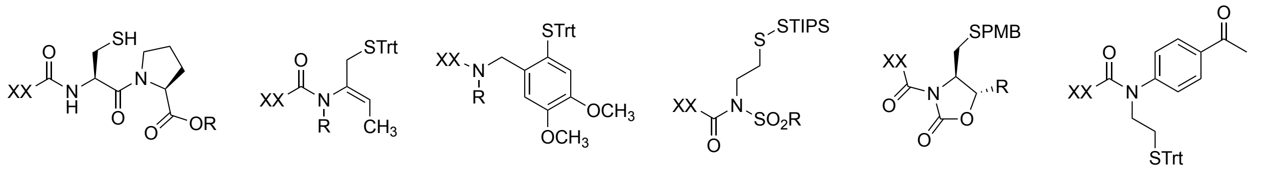


**Figure S1. Representative examples of *N*-to-*S* acyl transfer auxiliaries for peptide thioester synthesis**.[9-14] For a comprehensive review of *N*-to-*S* acyl transfer auxiliaries see ref. 15.[15]

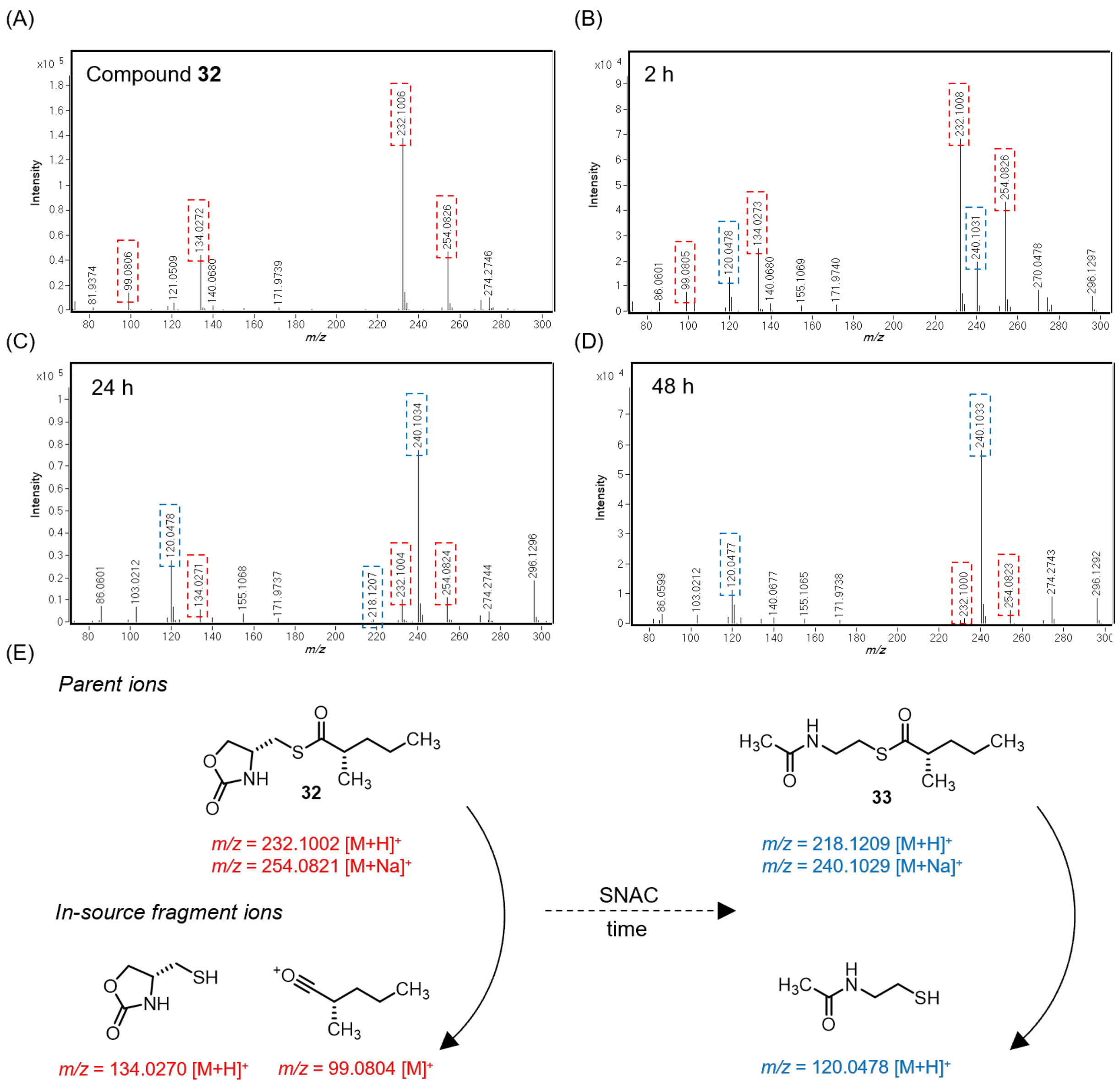


**Figure S2. Observation of the transthioesterification of oxazolidinone 32 to SNAC-thioester 33 during the chemoenzymatic synthesis of triketide lactone 34**. MS spectra (ESI$^+$; 6.0 – 6.2 min) are displayed for **32** (A), after 2 h (B), after 24 h (C), and after 48 h (D). (E). Masses of parent ions (ESI$^+$) of **32** and **33** and masses of corresponding in-source fragment ions.

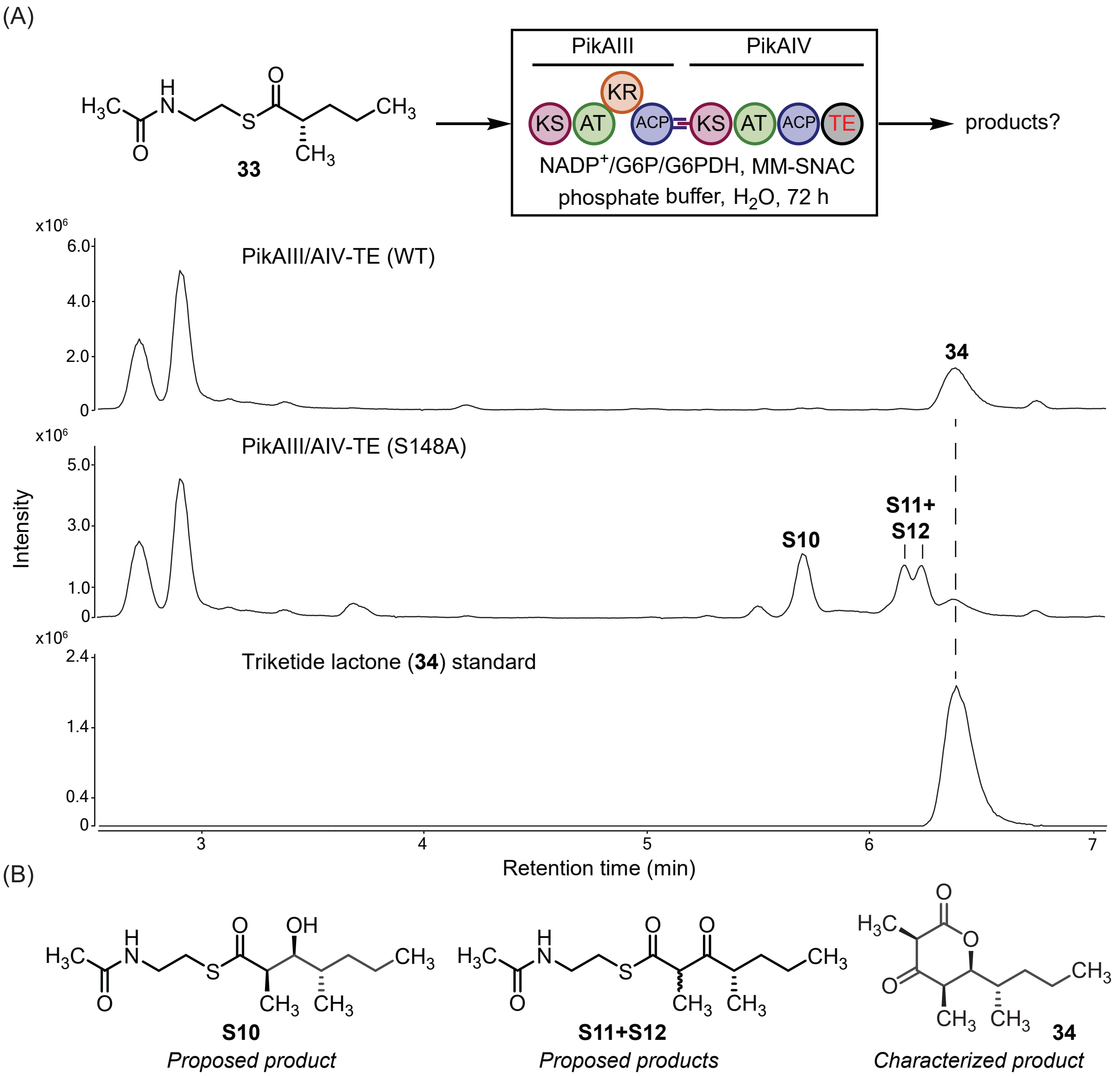


**Figure S3. The Pik TE terminal domain in PikAIV is required for efficient formation of triketide lactone 34.** (A) Reaction scheme and corresponding LC-MS traces ($ESI^+$) after 72 h. (B) Proposed reaction products **S10-S12** and characterized triketide lactone **34**. Production of mono extended products **S10-S12** increases when the Pik TE terminal domain is inactivated.[16, 17]

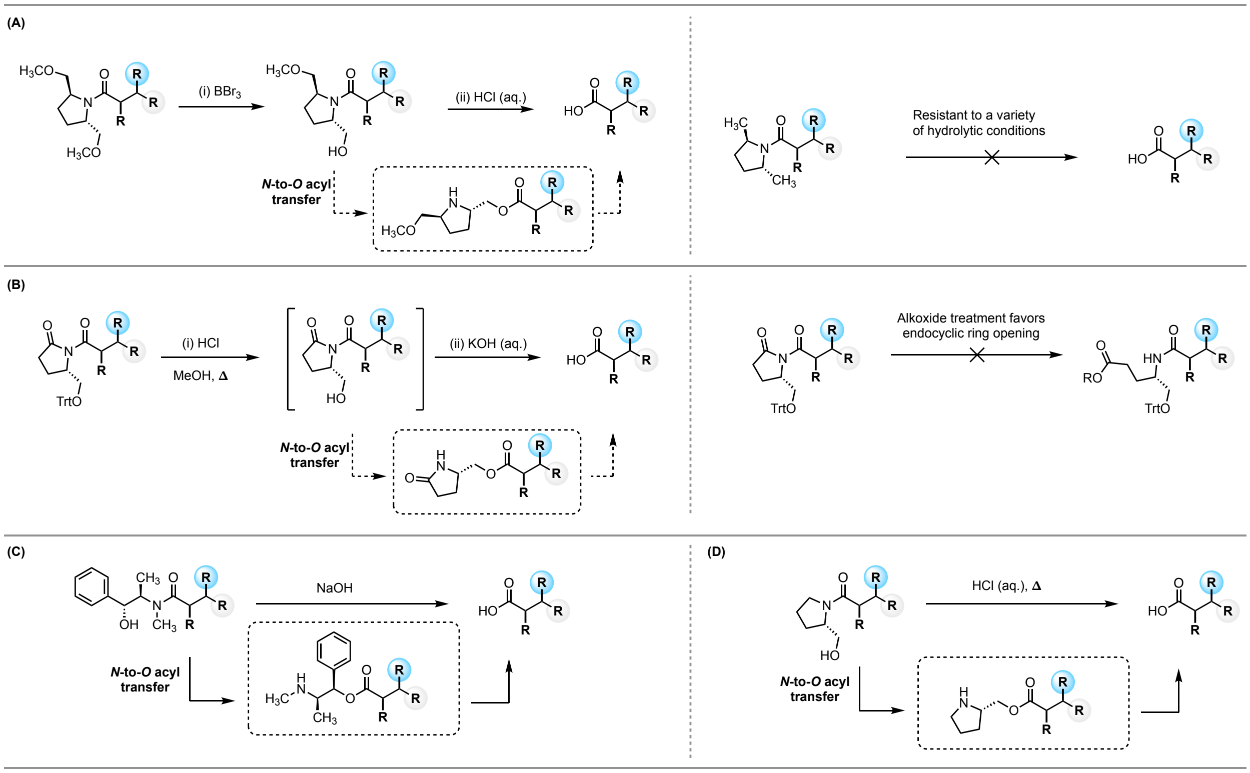


**Figure S4. Role of *N*-to-*O* acyl transfers in the cleavage of amide and imide auxiliaries**. (**A**) Cleavage of the bis(methoxymethyl)pyrrolidine chiral auxiliary requires a two-step process: first the mono-demethylation followed by hydrolysis. This was achieved using 3 M HCl under reflux or $BBr_3$ followed by aqueous HCl. The need to generate the free hydroxyl prior to hydrolysis would support a *N*-to-*O* transfer/ester hydrolysis sequence. This is further supported by the observation that the dimethylpyrrolidine chiral auxiliary was resistant to hydrolysis under a variety of hydrolytic conditions (*right*).[18] (**B**) Cleavage of Koga's chiral auxiliary requires a two-step process: first trityl deprotection followed by hydrolysis. We propose that the free hydroxyl derivative obtained after trityl-deprotection undergoes a *N*-to-*O* transfer to generate a transient ester intermediate, which subsequently gets hydrolyzed to furnish the carboxylic acid. This hypothesis is supported by the observation that hydrolysis under basic conditions, without cleavage of the trityl group, favored endocyclic ring opening over hydrolysis (*right*).[19, 20] (**C**) A *N*-to-*O* acyl transfer process has been proposed to be key to the mild and efficient hydrolysis of Myers pseudophedrine auxiliary.[21, 22] (**D**) An *N*-to-*O* acyl transfer process has been proposed to be key to the mild and efficient hydrolysis of the prolinol auxiliary.[23]

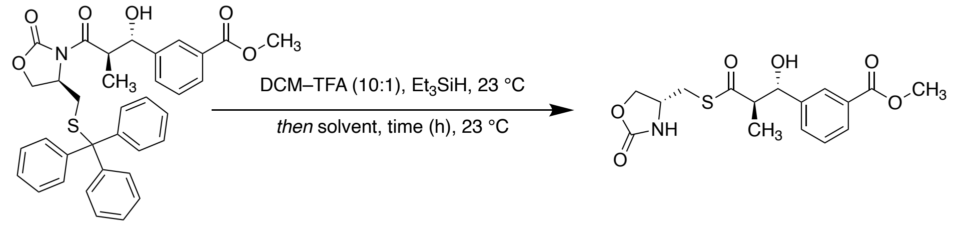


| entry | solvent 1 (0.4 M) | solvent 2 (0.2 M) | time (h) | equiv. of $Et_3N$ | NMR yield (%)[a,b] |
|---|---|---|---|---|---|
| 1 | $NaHCO_3$ (aq) | MeCN | 4 | - | 92 |
| 2 | $NaHCO_3$ (aq) | DMF | 4 | - | 88 |
| 3 | $NaHCO_3$ (aq) | IPA | 4 | - | 86 |
| 4 | $NaHCO_3$ (aq) | THF | 4 | - | 90 |
| 5 | - | MeCN | 4 | 5 | 97 |
| 6 | - | MeCN | 3.5 | 10 | 99 |
| 7 | - | IPA | 4 | 5 | 89 |
| 8 | - | IPA | 3.5 | 10 | 100 |
| 9 | - | THF | 4 | 5 | 88 |
| 10 | - | THF | 3.5 | 10 | 96 |
| 11 | - | acetone | 3.5 | 10 | 100 |
| 12 | - | toluene | 3.5 | 10 | 100 |
| 13 | - | EtOAc | 3.5 | 10 | 100 |
| 14 | - | $Et_2O$ | 3.5 | 10 | 99 |
| 15 | - | DMSO | 3.5 | 10 | 99 |
| 16 | - | 1,4-dioxane | 3.5 | 10 | 99 |
| 17 | - | DMF | 3.5 | 10 | 100 |
| 18 | - | 1-propanol | 3.5 | 10 | 96 |

**Table S1. Exploration of the reaction conditions to effect the *N*-to-*S* acyl transfer of compound 7.** All reactions were performed using the standard *N*-to-*S* acyl transfer conditions described in the synthetic procedures. [a]1,3,5-trimethoxybenzene was used as the internal standard. [b]All NMRs were measured in DMSO-$d_6$. Both polar protic and aprotic solvents successfully promoted the *N*-to-*S* acyl transfer reaction with near full conversion within the first 4 h (entries 1-

4). The acyl transfer reaction was further investigated under non-aqueous conditions employing excess triethylamine (entries 5-18). Although 5 equiv. of triethylamine was sufficient to affect the *N*-to-*S* acyl transfer, the use of 10 equiv. resulted in full conversion within 4 h. The *N*-to-*S* acyl transfer occurred in a wide range of solvents within 4 h. In summary, the desired solvent may be judiciously selected depending on the properties of the substrate, and the subsequent transformation of the obtained thioester.

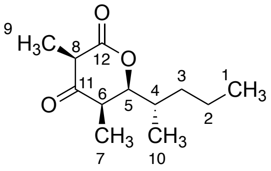


**34**

| Position | $^{13}$C [ppm] | $^{1}$H [ppm] | $J_{H,H}$ [Hz] | HMBC correlations | Key NOESY correlations |
|---|---|---|---|---|---|
| 1 | 14.4 | 0.94 | $J_{1,2}$ = 7.3 Hz | C-2, C-3 | |
| 2 | 19.6 | 1.47 (a), 1.29 (b) | - | C-1, C-3 | |
| 3 | 35.0 | 1.85 (a), 1.17 (b) | - | C-2, C-4 | |
| 4 | 33.2 | 1.87 | $J_{4,5}$ = 10.0 Hz, $J_{4,10}$ = 6.8 Hz | C-2, C-5, C-10 | H7, H10 |
| 5 | 81.1 | 4.34 | $J_{5,6}$ = 2.6 Hz | C-3, C-4, C-7, C-11 | H3b, H6, H8, H10 |
| 6 | 43.3 | 2.69 | $J_{6,7}$ = 7.6 Hz | C-7, C-11 | H5, H10 |
| 7 | 10.0 | 1.12 | - | C-5, C-6, C-11 | |
| 8 | 50.4 | 3.62 | $J_{8,9}$ = 6.6 Hz | C-9, C-11, C-12 | H5 |
| 9 | 8.2 | 1.36 | - | C-8, C-11, C-12 | |
| 10 | 14.4 | 0.91 | - | C-3, C-4, C-5 | |
| 11 | 206.1 | - | - | - | |
| 12 | 170.2 | - | - | - | |

**Table S2**. NMR spectroscopic data for triketide lactone **34** in $CDCl_3$. $^{1}$H (600 MHz), HSQC, and HMBC NMR spectroscopic data were acquired in $CDCl_3$. $^{1}$H chemical shifts were referenced to δ(C<u>H</u>$Cl_3$) = 7.26 ppm and $^{13}$C chemical shifts to δ($^{13}$<u>C</u>$DCl_3$) = 77.16 ppm. The data provided is for the keto tautomer, as the sample equilibrates to mainly the keto tautomer over time in $CDCl_3$.

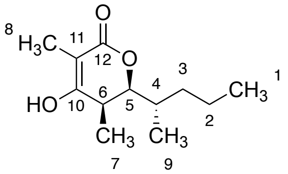


**34**

| Position | $^{13}$C [ppm] | $^{1}$H [ppm] | $J_{H,H}$ [Hz] | HMBC correlations | Key NOESY correlations |
|---|---|---|---|---|---|
| 1 | 14.7 | 0.94 | $J_{1,2}$ = 7.3 Hz | C-2, C-3 | |
| 2 | 20.5 | 1.48 (a), 1.30 (b) | - | C-1, C-3 | 2a and 2b: H1 |
| 3 | 35.8 | 1.81 (a), 1.16 (b) | - | C-1, C-2, C-4 | |
| 4 | 34.3 | 1.80 | $J_{4,5}$ = 10.3 Hz, $J_{4,9}$ = 6.8 Hz | C-5 | H7, H9 |
| 5 | 83.0 | 3.92 | $J_{5,6}$ = 3.0 Hz | C-4, C-7, C-10 | H3b, H6, H9 |
| 6 | 36.3 | 2.46 | $J_{6,7}$ = 7.0 Hz | C-7, C-10, C-11, C-12 | H5, H9 |
| 7 | 10.5 | 1.09 | - | C-5, C-6, C-10 | |
| 8 | 8.6 | 1.70 | - | C-6, C-7, C-10, C-11, C-12 | |
| 9 | 14.6 | 0.89 | - | C-3, C-4, C-5 | |
| 10 | 174.8 | - | - | - | |
| 11 | 97.8 | - | - | - | |
| 12 | 172.5 | - | - | - | |

**Table S3**. NMR spectroscopic data for triketide lactone **34** in $CD_3OD$. $^{1}$H (600 MHz), HSQC, and HMBC NMR spectroscopic data were acquired in $CD_3OD$. $^{1}$H chemical shifts were referenced to $\delta$(C$\underline{H}$$D_2$OD) = 3.31 ppm and $^{13}$C chemical shifts to $\delta$($^{13}\underline{C}D_3$OD) = 49.00 ppm. The data provided is for the enol tautomer, as the sample equilibrates to mainly the enol tautomer over time in $CD_3OD$.

**General Experimental Methods**.

**PKS Protein Biochemistry.** All protein biochemistry including protein expression and purification of PikAIII and PikAIV (50 μM stocks) was performed utilizing previously developed methods. [17, 24]

**Preparation of Phosphate Buffer.** Reaction phosphate buffer (800 mM) was prepared by dissolving sodium phosphate monobasic monohydrate (34.9 g), sodium phosphate dibasic anhydrous (77.7 g), and sodium chloride (23.4 g) in 1 L of milli-Q water. The pH was adjusted to 7.2 *via* addition of aqueous NaOH.

**Preparation of Recycling System Stock Solutions.** The following are recipes used to generate the reducing system stock solutions used for PKS reactions: Aqueous $NADP^+$ (50 mM): To a sterile, 50 mL conical tube was added milli-Q water (24 mL) and NADP disodium salt trihydrate (1.00 g, 1.19 mmol). The resulting mixture was vortexed to solubilize all contents. Aqueous G6P (500 mM): To a sterile, 50 mL conical tube was added milli-Q water (25 mL) and glucose-6-phosphate sodium salt (3.53 g, 12.5 mmol). The resulting mixture was vortexed to solubilize all contents. Aqueous G6PDH (100 mU/μL): Glucose-6-phosphate dehydrogenase (1 KU) was dissolved in sodium citrate buffer (10 mL, 5 mM, pH 7.3). Aqueous methylmalonyl *N*-acetylcysteamine (MM-SNAC 500 mM): The stock solution (pH = 7) was prepared as previously reported.[24] All aqueous stock solutions were flash frozen and stored at –80 °C until use.

**DNA cloning and mutagenesis.** *Pik TE S148A PCR Gene Amplification and Ligation into pET24-PikAIV Module Vector*

<u>PCR amplification for Pik TE S148A gene:</u> The HindIII and XhoI restriction sites were added by PCR amplification from pET28-Pik TE S148A with DNA primers for HindIII (forward primer: 5'-CCAACCAAGCTTTCCGGGGCCGACACCGGC-3') and XhoI (reverse primer: 5'-CCAACCCTCGAGGCCCGCCCCCTCGATGCCCTC-3'). 50 μL reaction: A modified QuikChange method (Stratagene) PCR contained distilled water, 4% DMSO, 0.2 μM each forward/reverse primers, 100 ng plasmid DNA template, 1X *Pyrococcus furiosus* (*Pfu*) ultra II DNA polymerase reaction buffer, 0.2 mM dNTP mix (Promega: U151A) and 1 unit of *Pfu* ultra II fusion HotStart DNA polymerase (Agilent: 600672). PCR cycle conditions: 1) polymerase activation: 95 °C for 3 min, 2) denature: 95 °C for 30 sec, 3) annealing: 68 °C for 30 sec, 4) extension: 65 °C for 1.5 min, 5) terminal extension: 65 °C for 5 min. Cycles run: 30 (for steps 2-4). Upon completion of PCR amplification, 2 μL of DpnI (NEB: R0176S) were added and incubated at 37 °C for 2 hours. After DpnI digestion, the DNA fragments were purified with QIAGEN PCR clean up kit.

<u>Restriction digest Pik TE S148A amplicon and pET24-PikAIV module vector:</u> The restriction enzymes utilized for this purpose included HindIII and XhoI located at both start and end of the Pik TE gene in the pET24-PikAIV vector. 55 μL reaction: DNA (~100-200 ng either PCR

amplicon product or vector), cut-smart 10X buffer, and water (if needed) were added to PCR tubes. Both restriction enzymes (120-140 ng) were added to the tubes, and they were incubated at 37 °C for 1.5 hours at which time a 5 µL aliquot of the two reactions was taken and diluted with 5 µL water and 2 µL Purple DNA loading dye. Also, prepared a sample of the uncut plasmid as a control: 2 µL of plasmid, 8 µL water, 2 µL dye. These were run on 1% agarose DNA gel, at 135 volts for 30 min to determine if the reactions were complete. Once reaction completion was determined, 5 µL of DNA loading dye was added to the reaction of the digested vector and it was run on a 1% agarose DNA gel fitted with large comb at 114 volts for 60 min. The band was extracted under UV light and subjected to a gel purification kit. Run alongside the gel purification was a PCR clean up kit on the digested amplicon DNA for ligation.
Ligation Pik TE S148A to pET24-PikAIV module vector: Water, T4 DNA ligase buffer (NEB: B0202S), vector DNA, and insert DNA (ratio of insert:vector = 5:1) were added to a PCR tube and T4 DNA ligase (NEB: M0202S) was lastly added. The 20 µL reaction was mixed gently by up and down pipetting. The reaction was incubated in a PCR thermocycler overnight at 16 °C. The reaction was chilled on ice, 1 aliquot of chemically BL21(DE3) competent cells was defrosted on ice, and 5 µL of DNA was added to the tube. Heat shock: 5 mins on ice, 47 °C warm bath for 45 sec. These were allowed to sit on ice for 5 min, 400 µL of SOC media was added, and the reactions were shaken at 37 °C for 1 hour. The reactions were plated on LB kanamycin (50 µg/mL) plates using glass beads and grown overnight in 37 °C incubator. The plasmid miniprep kit from QIAGEN was utilized to extract DNA for sequencing plasmids and confirm successful Pik TE S148A ligation to the desired PikAIV module for further protein expression and purification following previously developed methods.[17, 24]

**General Experimental Procedures**. All reactions were magnetically stirred and conducted in a flame-dried single neck round-bottomed flask under a positive pressure of argon (i.e., balloon) unless otherwise noted. Air- and moisture sensitive liquids and solutions were transferred with a plastic syringe (microsyringe for small scale reactions) or by an oven-dried stainless-steel cannula through rubber septa. All purified products were dried under high vacuum (overnight if necessary). Organic solutions and volatiles were concentrated by rotary evaporation using a water bath at 30–35 °C. Low temperature reactions were carried out in a Dewar vessel filled with a cooling agent: acetone/dry ice (–78 °C) and water/ice (0 °C). Alternatively, a Julabo FT-902 cryo cooler was used for multi-gram scale reactions. High temperature (> 23 °C) reactions were carried out in a heat-on block and external block temperatures were measured. Thin layer chromatography was performed with SiliaPlate glass back scored plates (60Å). Visualization of the spots was achieved by UV irradiation at 245/365 nm (Analytikjena, 6W/0.16A) or by potassium permanganate staining then charring. Purification of crude products was done by flash column chromatography (Biotage Selekt) using silica (particle size 230-400 mesh) unless otherwise noted. Anion-exchange chromatography was performed as described by Béland *et al.*,[25] employing trimethylamine acetate-functionalized silica gel (SiliaBond® TMA Acetate). Yields refer to spectroscopically ($^1$H, $^{13}$C) pure materials unless otherwise noted.

**Materials.** Commercial solvents reagents were used as received with the following exceptions: Dichloromethane, tetrahydrofuran, diethyl ether, hexane, acetonitrile, and toluene were dried with a solvent dispensing system (Inertcorp; model number: PS-MD-7-EN). Ethyl acetate was dried over activated (>12 h, 200 °C) 4 Å molecular sieves for 24 h. Hydrocinnamaldehyde (technical grade; 90% purity) and benzaldehyde were distilled under vacuum prior to use and stored in a Schlenk flask at 5 °C. *N*,*N*-Diisopropylethylamine and triethylamine were distilled over potassium hydroxide and stored under inert conditions. CuBr•DMS was freshly prepared according to the method of House[26] and recrystallized prior to use. Di-*n*-butylboryl trifluoromethanesulfonate was freshly prepared according to a protocol by Evans *et al*. and stored in a Schlenk flask at 5 °C.[27] Copper(I) thiophene-2-carboxylate was prepared according to Liebiskind *et al.*[28] pH 7 buffer solution was prepared according to Sorensen's phosphate buffer table (0.1 M). Deuterochloroform was dried over activated (200 °C, 12 h, high vacuum) 4 Å molecular sieves. *S*-trityl-L-cysteine,[1] 2,4,6-triisopropylbenzenesulfonyl azide (trisyl azide),[29] tetramethylguanidinium azide,[30] and tributylborane[31] were prepared according to the literature. Ethylzinc iodide was freshly prepared according to Fukuyama's protocol.[32] Trimethylamine acetate-functionalized silica gel (SiliaBond® TMA Acetate) were purchased from SiliCycle (Quebec City, CA).

**Instrumentation**. $^{1}$H NMR (500 MHz) and $^{13}$C NMR (126 MHz) were recorded at standard temperature and pressure on a Bruker Avance III Spectrometer in the indicated solvent ($CDCl_3$ or $CD_3OD$). Chemical shifts ($^{1}$H) are expressed in parts per million (ppm, δ scale) downfield from tetramethylsilane and are referenced to residual protium in the NMR solvent ($CDCl_3$, δ 7.26; $CD_3OD$, δ 3.31). Data are represented as follows: chemical shift, multiplicity (s = singlet, d = doublet, t = triplet, q = quarter, m = multiplet and/or multiple resonances, br = broad, app = apparent), coupling constant in Hertz, integration, and assignment. Chemical shifts ($^{13}$C) are expressed in parts per million (ppm, δ scale) downfield from tetramethylsilane and are referenced to the carbon resonances of the solvent ($CDCl_3$, δ 77.0; $CD_3OD$, δ 49.0). All NMR *δ* values are given in ppm, and all *J* values are in Hz. 2D-NMR spectroscopy such as [1H, 1H] COSY (Correlation Spectroscopy), [1H, 13C] HSQC (Heteronuclear Single Quantum Coherence) and long range [1H, 13C] HMBC (Heteronuclear Multiple Bond Connectivity), were also recorded to unambiguously assign all proton and carbon signals.

Analytical liquid chromatography/mass spectrometry (LC/MS) was performed on a Agilent LC/MS instrument (1260 Infinity II) equipped with a reverse-phase $C_{18}$ column (2.7 µm particle size, 3.0 × 100 mm), electrospray (ESI) mass spectrometry detector, and photodiode array detector. Samples were eluted with a linear gradient of 30% acetonitrile–water containing 0.1% formic acid→95% acetonitrile-water containing 0.1% formic acid over 3.00 min, followed by 95% acetonitrile-water containing 0.1% formic acid for 1.00 min, at a flow rate of 800 µL/min. High-resolution mass spectrometry (HRMS) were obtained on an UPLC/HRMS instrument (Agilent 1290 Infinity II) equipped with a Q-TOF (UHD Accurate-Mass) and photodiode array detector. Unless otherwise noted, samples were eluted over a reverse-phase $C_{18}$ column (1.8 µm particle

size, 2.1 × 50 mm) with a linear gradient of 5% acetonitrile–water containing 0.1% formic acid→100% acetonitrile–water containing 0.1% formic acid for 10 min, at a flow rate of 300 μL/min.

Optical rotations were measured on a polarimeter Anton Paar (MCP 5100) at 20 °C at a wavelength of 589 nm in a 1 mL quartz cell (0.5 dm length). The concentration is given in g/100 ml.

The following abbreviations are used: **rpm**: rounds per minute; **DCM**: dichloromethane; **DMAP**: 4-dimethylaminopyridine; **THF**: tetrahydrofuran; **DMSO**: dimethylsulfoxide; **Boc**: *tert*-butoxycarbonyl; **EtOAc**: ethyl acetate; **TFA**: trifluoroacetic acid. ***n*-$Bu_2$BOTf**: di-*n*-butylboryl trifluoromethanesulfonate; **DIPEA**: *N*,*N*-diisopropylethylamine; **DMF**: *N*,*N*-dimethylformamide; **$^n$PrOH**: 1-propanol; **$Et_3$N**: triethylamine; **TMSCl**: chlorotriemethylsilane; **MeOH**: methanol; **$Et_3$-SiH**: triethylsilane; **DTT**: *DL*-dithiothreitol; **AgTFA**: silver trifluoroacetate ; **NaHMDS**: sodium bis(trimethylsilyl)amide; **TCEP**: tris(2-carboxyethyl)phosphine; **$PdCl_2$(dppf)**: [1,1′-Bis(diphenylphosphino)ferrocene]dichloropalladium(II); **TFP**: tri-2-furylphosphine; **AgOTf**: silver trifluoromethanesulfonate; **PivCl**: pivaloyl chlroide; **LDA**: lithium di-*iso*-propyl amide; **CuTC**: copper(I) thiophene-2-carboxylate; ***n*-BuLi**: *n*-butyllithium; **TMGA**: tetramethylguanidinium azide; **IPA**: isopropyl alcohol; **AcSH**: thioacetic acid; **NBS**: *N*-bromosuccinimide; **SNAC**: *N*-acetylcysteamine; **MeCN**: acetonitrile; **NaHMDS**: sodium bis(trimethylsilyl)amide; **KHMDS**: potassium bis(trimethylsilyl)amide; **EtOH**: ethanol.

**Synthetic Procedures.**

*Synthesis of the S-trityl amino alcohol **S1***:

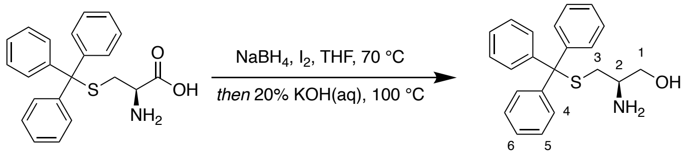


*S*-Trityl-L-Cysteine **S1**

The experimental procedure has been modified from the literature.[2] To a suspension of sodium borohydride (5.00 g, 132 mmol, 2.40 equiv) in tetrahydrofuran (110 mL) was added *S*-trityl-L-cysteine (20.0 g, 55.0 mmol, 1 equiv) *portionwise* at 0 ºC. A solution of elemental iodine (14.0 g, 55.0 mmol, 1.00 equiv) in tetrahydrofuran (70 mL) was slowly poured into the reaction mixture and the resulting brown solution was refluxed at 70 °C for 18 h. The product mixture was left to cool to 23 °C then methanol (25–30 mL) was added slowly (*CAUTION: effervescence)* until the cloudy solution turned clear yellow. The product mixture was concentrated. The resulting white paste was treated with 20% (*w*/*v*) aqueous KOH solution (300 mL) and refluxed at 100 °C for 4 h. After cooling to 23 °C, the aqueous solution was extracted with dichloromethane (4 × 100 mL). The combined organic extracts were dried over sodium sulphate and the dried solution was filtered. The filtrate was concentrated to afford a yellow sticky gum (*ca*. 15.8 g) which was used in the next step without further purification. The spectroscopic data are in agreement with the literature.[33]

$^{1}$H NMR (500 MHz, $CDCl_3$) δ 7.47 – 7.41 (m, 6H, $H_4$), 7.32 – 7.27 (m, 6H, $H_5$), 7.24 – 7.19 (m, 3H, $H_6$), 3.41 (dd, $J$ = 10.8, 4.1 Hz, 1H, $H_1$), 3.18 (dd, $J$ = 10.8, 6.9 Hz, 1H, $H_1$), 2.63 – 2.53 (m, 1H, $H_2$), 2.33 (dd, $J$ = 12.6, 5.2 Hz, 1H, $H_3$), 2.23 (dd, $J$ = 12.5, 7.6 Hz, 1H, $H_3$), 1.91 (br s, 3H, –N$H_2$, –O$H$).

*Synthesis of the S-trityl oxazolidinone **1***:

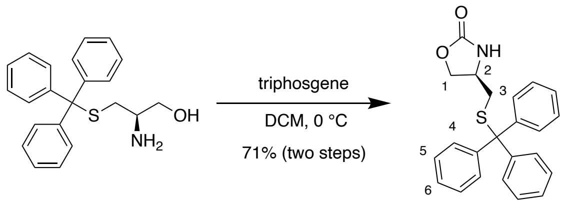


**S1** **1**

To a solution of the crude amino alcohol **S1** obtained above (*ca*. 45 mmol, 1 equiv) in dichloromethane (113 mL) was added triethylamine (12.6 mL, 90.3 mmol, 2.00 equiv) at 0 °C. A solution of triphosgene (13.4 g, 45.1 mmol, 1.00 equiv) in dichloromethane (113 mL) was added dropwise to the reaction mixture at 0 °C over 10 min using an addition funnel. The reaction mixture was stirred for 2 h at 0 °C. The product mixture was diluted by addition of diethyl ether (150 mL). The resulting heterogeneous mixture was filtered and the filtrate was concentrated to afford a pale yellow foamy solid. The residue was purified by flash-column chromatography (eluting with 30% ethyl acetate-hexane initially, grading to 60% ethyl acetate-hexane, linear gradient) to provide the *S-trityl oxazolidinone* **1** as a white solid (14.8 g, 71% over two steps).

$R_f$ = 0.52 (hexane/EtOAc = 1:1, UV)

$[\alpha]_D^{20}$: +5.31 (*c* 0.49, $CHCl_3$)

$^1$H NMR (500 MHz, $CDCl_3$) δ 7.44 – 7.37 (m, 6H, $H_4$), 7.30 (dd, *J* = 8.4, 6.7 Hz, 6H, $H_5$), 7.26 – 7.21 (m, 3H, $H_6$), 5.05 (s, 1H, –N*H*), 4.26 (app t, *J* = 8.6 Hz, 1H, $H_1$), 3.85 (dd, *J* = 8.9, 5.3 Hz, 1H, $H_1$), 3.46 – 3.16 (m, 1H, $H_2$), 2.44 (app qd, *J* = 12.9, 6.7 Hz, 2H, $H_3$).

$^{13}$C NMR (126 MHz, $CDCl_3$) δ 158.6 (C), 144.3 (3 × C), 129.6 (6 × CH), 128.3 (6 × CH), 127.2 (3 × CH), 69.5 ($CH_2$), 67.4 (C), 51.4 (CH), 36.8 ($CH_2$).

HRMS-CI (m/z): $[M + H]^+$ calcd for $C_{23}H_{22}NO_2S$, 376.1366; found, 376.1375.

*Synthesis of the N-propionyl trityl oxazolidinone* ***2***:

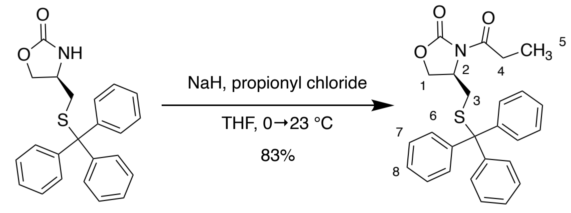


To a solution of *S*-trityl oxazolidinone **1** (5.20 g, 13.8 mmol, 1 equiv) in tetrahydrofuran (70 mL) was added sodium hydride (60% dispersion in oil, 1.39 g, 34.6 mmol, 2.50 equiv) at 0 °C and the reaction mixture was stirred for 1 h. To the reaction mixture was added propionyl chloride (2.40 mL, 27.7 mmol, 2.00 equiv) dropwise at 0 °C. The reaction mixture was warmed to 23 °C and stirred for 18 h. The product mixture was quenched by the addition of saturated aqueous ammonium chloride solution (50 mL). The quenched solution was partially concentrated and transferred to a separatory funnel. The layers were separated, and the aqueous layer was extracted with ethyl acetate (3 × 50 mL). The combined organic extracts were sequentially washed with saturated aqueous sodium bicarbonate solution (3 × 50 mL) and saturated aqueous sodium chloride solution (50 mL). The washed organic extracts were dried over sodium sulphate and filtered. The filtrate was concentrated, and the yellow residue was purified by flash-column chromatography (eluting with 10% ethyl acetate-hexane initially, grading to 30% ethyl acetate-hexane, linear gradient) to afford the *N-propionyl trityl oxazolidinone* **2** as a white solid (4.93 g, 83%).

$R_f$ = 0.46 (hexane/EtOAc = 8:2)

$[\alpha]_D^{20}$: +32.0 (*c* 0.44, $CHCl_3$)

$^1$H NMR (500 MHz, $CDCl_3$) δ 7.38 – 7.32 (m, 6H, $H_6$), 7.26 – 7.20 (m, 6H, $H_7$), 7.18 – 7.13 (m, 3H, $H_8$), 4.10 – 3.96 (m, 2H, $H_{1\text{-}2}$), 3.83 – 3.71 (m, 1H, $H_1$), 2.84 – 2.73 (m, 2H, $H_4$), 2.70 (m, 1H, $H_3$), 2.55 – 2.45 (m, 1H, $H_3$), 1.05 (t, *J* = 7.3 Hz, 3H, $H_5$).

$^{13}$C NMR (126 MHz, $CDCl_3$) δ 173.9 (C), 153.5 (C), 144.4 (3 × C), 129.6 (6 × CH), 128.2 (6 × CH), 127.1 (3 × CH), 67.2 (C), 66.3 ($CH_2$), 53.0 (CH), 33.6 ($CH_2$), 29.2 ($CH_2$), 8.3 ($CH_3$).

HRMS-CI (m/z): $[M + Na]^+$ calcd for $C_{26}H_{25}NO_3SNa$, 454.1447; found, 454.1454.

*Synthesis of the syn-aldol product **3***:

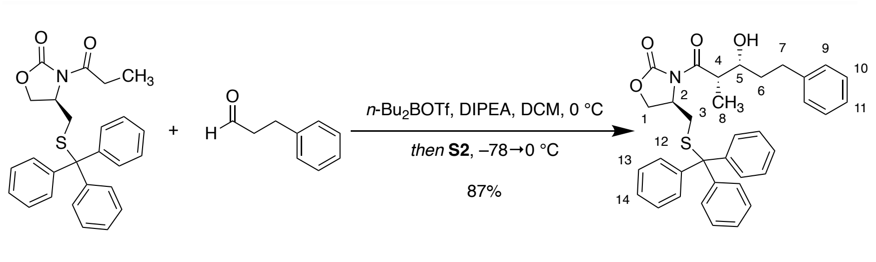


To a solution of *N*-propionyl oxazolidinone **2** (500 mg, 1.16 mmol, 1 equiv) in dichloromethane (4.6 mL) were sequentially added dropwise di-*n*-butylboryl trifluoromethanesulfonate (1.0 M in dichloromethane, 2.50 mL, 2.55 mmol, 2.20 equiv) and *N*,*N*-diisopropylethylamine (0.480 mL, 2.78 mmol, 2.40 equiv) over 10 minutes (syringe pump; 5 min each) at 0 °C. The reaction mixture was stirred at 0 °C for 1 h then cooled to –78 °C. Hydrocinnamaldehyde (**S2**, 0.230 mL, 1.74 mmol, 1.50 equiv) was added dropwise over 5 min (syringe pump) and the reaction mixture was stirred at –78 °C for 30 min and allowed to slowly warm to 0 °C over 2 h. The product mixture was quenched at 0 °C with pH 7 phosphate buffer (1.4 mL) followed by the dropwise addition of methanol (4.2 mL). A 30% hydrogen peroxide–methanol solution (4.2 mL, 2:1 *v/v*) was added over 10 minutes (syringe pump) and the quenched product mixture was stirred at 0 °C for 30 min. The quenched product mixture was transferred to a separatory funnel and the layers were separated. The aqueous layer was diluted with saturated aqueous sodium chloride solution (15 mL) and then extracted with ethyl acetate (3 × 20 mL). The combined organic extracts were washed sequentially with 1.0 M aqueous hydrogen chloride solution (25 mL), saturated aqueous sodium bicarbonate solution (25 mL) and saturated aqueous sodium chloride solution (25 mL). The washed organic extracts were dried over sodium sulphate and filtered. The filtrate was concentrated to provide a yellow residue that was purified by flash-column chromatography (eluting with 10% ethyl acetate-hexane initially, grading to 30% ethyl acetate-hexane, linear gradient) to afford the *syn-aldol product* **3** as a white solid (573 mg, 87%).

*Note*: The reaction mixture turns orange-red upon the addition of the boron reagent and then turns dark navy when the base is added. The reaction mixture gradually turns light yellow over the course of the reaction.

$R_f$ = 0.54 (hexane/EtOAc = 7:3)

$[\alpha]_D^{20}$: +24.1 (*c* 0.22, $CHCl_3$)

$^{1}$H NMR (500 MHz, $CDCl_3$) δ 7.43 – 7.38 (m, 6H, $H_{12}$), 7.33 – 7.27 (m, 7H, $H_{9-11,}$ $H_{13-14}$), 7.25 – 7.15 (m, 7H, $H_{9-11,}$ $H_{13-14}$), 4.16 (m, 1H, $H_2$), 4.11 (t, *J* = 8.6 Hz, 1H, $H_1$), 3.89 (m, 1H, $H_5$), 3.84 (dd, *J* = 8.8, 3.2 Hz, 1H, $H_1$), 3.67 (qd, *J* = 7.1, 2.5 Hz, 1H, $H_4$), 2.82 (m, 1H, $H_7$), 2.72 – 2.66 (m, 1H, $H_7$), 2.66 (app d, *J* = 1.9 Hz, 1H, $H_3$), 2.56 (dd, *J* = 12.6, 3.1 Hz, 1H, $H_3$), 1.86 (m, 1H, $H_6$), 1.65 (m, 1H, $H_6$), 1.22 (d, *J* = 7.2 Hz, 3H, $H_8$).

$^{13}$C NMR (126 MHz, $CDCl_3$) δ 177.6 (C), 152.9 (C), 144.3 (3 × C), 142.0 (C), 129.6 (6 × CH), 128.7 (2 × CH), 128.5 (2 × CH), 128.3 (6 × CH), 127.2 (3 × CH), 126.0 (CH), 70.5 (CH), 67.2 (C), 66.2 ($CH_2$), 53.0 (CH), 42.3 (CH), 35.4 ($CH_2$), 33.4 ($CH_2$), 32.3 ($CH_2$), 10.7 ($CH_3$).

HRMS-CI (m/z): $[M + Na]^+$ calcd for $C_{35}H_{35}NO_4SNa$, 588.2179; found, 588.2181.

*Synthesis of the oxazolidinone thioester **5***:

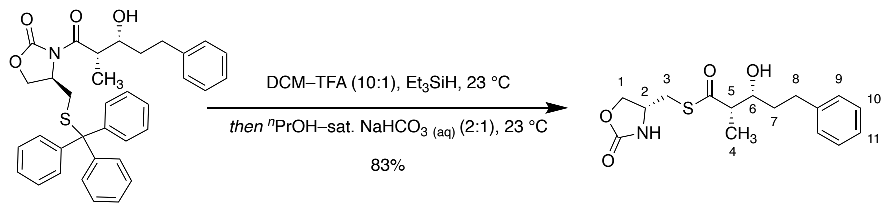


**3** **5**

To a solution of *syn*-aldol product **3** (376 mg, 0.665 mmol, 1 equiv) in dichloromethane (2.0 mL) were added trifluoroacetic acid (204 µL, 2.66 mmol, 4.00 equiv) and triethylsilane (296 µL, 1.86 mmol, 2.80 equiv) at 23 °C. The reaction mixture was stirred for 30 min at 23 °C. The reaction mixture was concentrated and dried under high vacuum to afford a white solid that was used in the next step without further purification.

To the trityl deprotected adduct was added 1-propanol (3.3 mL) and a saturated aqueous sodium bicarbonate solution (1.7 mL). The reaction mixture was stirred for 4 h at 23 °C. The product mixture was diluted with saturated aqueous sodium chloride solution (8 mL). The diluted product mixture was transferred to a separatory funnel and extracted with ethyl acetate (3 × 10 mL). The combined organic extracts were dried over sodium sulphate and filtered. The filtrate was concentrated, and the white residue was purified by flash-column chromatography (eluting with 100% dichloromethane initially, grading to 20% methanol-dichloromethane, linear gradient) to afford the *oxazolidinone thioester* **5** as a pale-yellow oil (179 mg, 83%).

$R_f$ = 0.35 (DCM/MeOH = 95:5)

$[\alpha]_D^{20}$: +8.04 (*c* 0.55, $CHCl_3$)

$^1$H NMR (500 MHz, $CDCl_3$) δ 7.29 (app dd, *J* = 8.1, 6.9 Hz, 3H, $H_{9\text{-}11}$), 7.20 (app dt, *J* = 8.1, 2.0 Hz, 2H, $H_{9\text{-}11}$), 5.87 (s, 1H,–N*H*), 4.48 (app t, *J* = 8.3 Hz, 1H, $H_1$), 4.15 – 4.03 (m, 2H, $H_{1\text{-}2}$), 4.03 – 3.95 (m, 1H, $H_6$), 3.25 (dd, *J* = 14.2, 4.8 Hz, 1H, $H_3$), 2.94 (dd, *J* = 14.2, 5.0 Hz, 1H, $H_3$), 2.87 – 2.82 (m, 1H, $H_8$), 2.78 (qd, *J* = 7.0, 3.4 Hz, 1H, $H_5$), 2.65 (m, 1H, $H_8$), 1.83 (m, 1H, $H_7$), 1.77 – 1.61 (m, 1H, $H_7$), 1.21 (d, *J* = 7.0 Hz, 3H, $H_4$).

$^{13}$C NMR (126 MHz, $CDCl_3$) δ 202.7 (C), 159.7 (C), 141.8 (C), 128.6 (4 × CH), 126.1 (CH), 71.6 (CH), 69.3 ($CH_2$), 53.9 (CH), 51.8 (CH), 36.3 ($CH_2$), 33.2 ($CH_2$), 32.5 ($CH_2$), 10.7 ($CH_3$).

HRMS-CI (m/z): $[M + H]^+$ calcd for $C_{16}H_{22}NO_4S$, 324.1264; found, 324.1267.

*Synthesis of the anti-aldol product 7*:

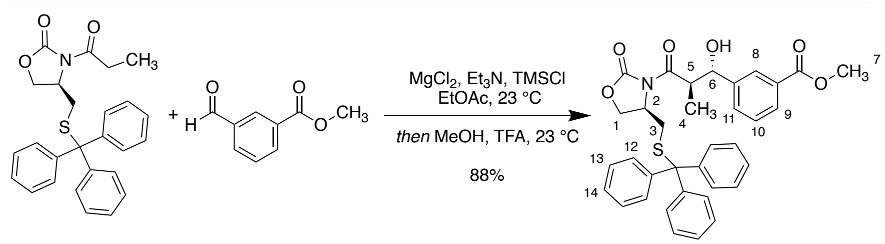


**2** **6** **7**

To a solution of *N*-propionyl oxazolidinone **2** (3.50 g, 8.11 mmol, 1 equiv) in anhydrous ethyl acetate (16 mL) were added magnesium chloride (77.3 mg, 0.81 mmol, 0.10 equiv), triethylamine (2.30 mL, 16.2 mmol, 2.00 equiv), methyl 3-formylbenzoate (1.60 g, 9.73 mmol, 1.20 equiv), and trimethylsilyl chloride (1.50 mL, 12.1 mmol, 1.50 equiv). The reaction mixture was stirred for 20 h at 23 °C and filtered through a short plug of silica eluting with diethyl ether (2 × 10 mL). The filtrate was concentrated then dissolved in methanol (40 mL). Three drops of trifluoroacetic acid were added and the reaction mixture was stirred for 30 min at 23 °C. The product mixture was concentrated, and the residue was purified by flash-column chromatography (eluting with 15% ethyl acetate-hexane initially, grading to 30% ethyl acetate-hexane, linear gradient) to afford a the *anti-aldol product* **7** as a white solid (4.23 g, 88%).

$R_f$ = 0.33 (hexane/EtOAc = 8:2)

$[\alpha]_D^{20}$: –29.8 (*c* 0.28, $CHCl_3$)

$^1$H NMR (500 MHz, $CDCl_3$) δ 8.00 (s, 1H, $H_8$), 7.94 (app dt, *J* = 7.8, 1.5 Hz, 1H, $H_9$), 7.54 (app dt, *J* = 7.8, 1.5 Hz, 1H, $H_{11}$), 7.40 (dd, *J* = 8.5, 1.3 Hz, 6H, $H_{12}$), 7.31 (dd, *J* = 8.5, 6.8 Hz, 6H, $H_{13}$), 7.25 – 7.21 (m, 4H, $H_{10,14}$), 4.78 (app t, *J* = 7.4 Hz, 1H, $H_6$), 4.17 (app p, *J* = 7.0 Hz, 1H, $H_5$), 4.12 – 4.00 (m, 2H, $H_{1\text{-}2}$), 3.91 (s, 3H, $H_7$), 3.81 (dd, *J* = 8.7, 2.9 Hz, 1H, $H_1$), 3.15 (d, *J* = 7.3 Hz, 1H, –O*H*), 2.65 (dd, *J* = 12.8, 3.3 Hz, 1H, $H_3$), 2.41 (dd, *J* = 12.8, 9.1 Hz, 1H, $H_3$), 1.05 (d, *J* = 6.9 Hz, 3H, $H_4$).

$^{13}$C NMR (126 MHz, $CDCl_3$) δ 176.2 (C), 166.9 (C), 153.2 (C), 144.3 (3 × C), 142.4 (C), 131.0 (CH), 130.4 (C), 129.6 (6 × CH), 129.3 (CH), 128.8 (CH), 128.3 (6 × CH), 128.0 (CH), 127.2 (3 × CH), 76.7 (CH), 67.4 (C), 66.3 ($CH_2$), 53.4 (CH), 52.3 ($CH_3$), 44.4 (CH), 33.3 ($CH_2$), 14.8 ($CH_3$).

HRMS-CI (m/z): $[M + NH_4]^+$ calcd for $C_{35}H_{33}NO_6SNH_4$, 613.2367; found, 613.2371.

*Synthesis of the oxazolidinone thioester **S3***:

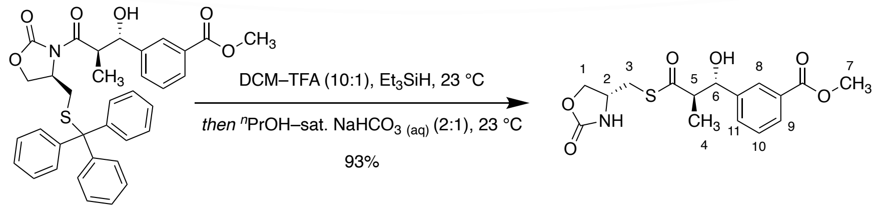


**7** **S3**

To a solution of *anti*-aldol product **7** (1.50 g, 2.52 mmol, 1 equiv) in dichloromethane (7.6 mL) were added trifluoroacetic acid (0.77 mL, 10.1 mmol, 4.00 equiv) and triethylsilane (1.10 mL, 7.05 mmol, 2.80 equiv) at 23 °C. The reaction mixture was stirred for 30 min at 23 °C. The reaction mixture was concentrated and dried under high vacuum to afford a white solid that was used in the next step without further purification.

To the trityl deprotected adduct was added 1-propanol (13 mL) and a saturated aqueous sodium bicarbonate solution (6.3 mL). The reaction mixture was stirred for 4 h at 23 °C. The product mixture was diluted with saturated aqueous sodium chloride solution (20 mL). The diluted product mixture was transferred to a separatory funnel and extracted with ethyl acetate (3 × 30 mL). The combined organic extracts were dried over sodium sulphate and filtered. The filtrate was concentrated, and the white residue was purified by flash- column chromatography (eluting with 100% dichloromethane initially, grading to 20% methanol-dichloromethane, linear gradient) to afford the *oxazolidinone thioester* **S3** as a white foam (830 mg, 93%).

$R_f$ = 0.48 (DCM/MeOH = 95:5)

$[\alpha]_D^{20}$: –100 (*c* 0.40, $CHCl_3$)

$^1$H NMR (500 MHz, $CDCl_3$) δ 8.02 (s, 1H, $H_8$), 7.99 (app dt, *J* = 7.7, 1.5 Hz, 1H, $H_9$), 7.55 (app dt, *J* = 7.7, 1.6 Hz, 1H, $H_{11}$), 7.45 (app t, *J* = 7.7 Hz, 1H, $H_{10}$), 5.52 (s, 1H, –N*H*), 4.87 (d, *J* = 8.7 Hz, 1H, $H_6$), 4.55 – 4.33 (m, 1H, $H_1$), 4.23 – 3.98 (m, 2H, $H_{1-2}$), 3.93 (s, 3H, $H_7$), 3.19 – 3.12 (m, 2H, $H_3$), 3.10 – 3.03 (m, 1H, $H_5$), 2.69 (s, 1H, –O*H*), 1.02 (d, *J* = 7.1 Hz, 3H, $H_4$).

$^{13}$C NMR (126 MHz, $CDCl_3$) δ 202.2 (C), 166.9 (C), 159.1 (C), 142.0 (C), 131.4 (CH), 130.7 (C), 129.7 (CH), 128.9 (CH), 127.9 (CH), 76.5 (CH), 69.2 ($CH_2$), 56.1 (CH), 52.4 ($CH_3$), 52.1 (CH), 33.1 ($CH_2$), 15.1 ($CH_3$).

HRMS-CI (m/z): $[M + H]^+$ calcd for $C_{16}H_{20}NO_6S$, 354.1006; found, 354.1008.

*Synthesis of the aldehyde **8***:

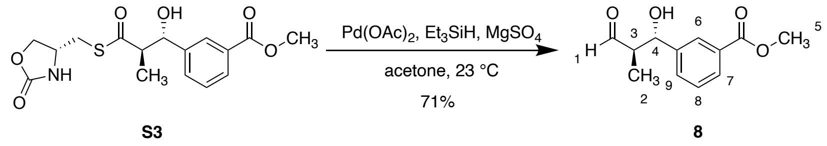


To a solution of oxazolidinone thioester **S3** (154 mg, 0.436 mmol, 1 equiv) in acetone (15 mL) were added palladium acetate (29.4 mg, 0.131 mmol, 0.30 equiv) and anhydrous magnesium sulphate (420 mg, 3.49 mmol, 8.00 equiv). Triethylsilane (0.420 mL, 2.62 mmol, 6.00 equiv) was added dropwise over 15 min (syringe pump) and the reaction mixture was stirred for 2 h at 23 °C. The black product mixture was quenched with methanol (5 mL) and concentrated. The oily residue was purified by flash column chromatography (eluting with 100% hexane initially, grading to 40% ethyl acetate-hexane, linear gradient) to afford the *aldehyde* **8** as a colourless oil (69.0 mg, 71%).

$R_f$ = 0.39 (hexane/EtOAc = 6:4)

$[\alpha]_D^{20}$: +30.8 (*c* 0.55, $CHCl_3$)

$^1H$ NMR (500 MHz, $CDCl_3$) δ 9.83 (d, *J* = 1.7 Hz, 1H, $H_1$), 8.02 (s, 1H, $H_6$), 7.99 (dt, *J* = 7.7, 1.5 Hz, 1H, $H_7$), 7.56 (dt, *J* = 7.8, 1.6 Hz, 1H, $H_9$), 7.45 (app t, *J* = 7.7 Hz, 1H, $H_8$), 4.88 (d, *J* = 8.4 Hz, 1H, $H_4$), 3.92 (s, 3H, $H_5$), 2.90 (app d, *J* = 3.3 Hz, 1H, –O*H*), 2.78 (dqd, *J* = 8.9, 7.4, 1.6 Hz, 1H, $H_3$), 0.94 (d, *J* = 7.3 Hz, 3H, $H_2$).

$^{13}C$ NMR (126 MHz, $CDCl_3$) δ 204.7 (C), 167.0 (C), 142.0 (C), 131.3 (CH), 130.7 (C), 129.6 (CH), 128.9 (CH), 128.0 (CH), 75.2 (CH), 53.3 (CH), 52.4 ($CH_3$), 11.2 ($CH_3$).

HRMS-CI (m/z): $[M + NH_4]^+$ calcd for $C_{12}H_{14}O_4NH_4$, 240.1230; found, 240.1228.

*Synthesis of the anti-aldol product analogue **S4***:

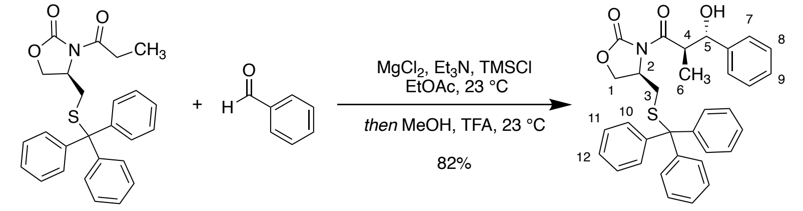


**2** **S4**

To a solution of *N*-propionyl oxazolidinone **2** (100 mg, 0.232 mmol, 1 equiv) in dry ethyl acetate (0.46 mL) were added magnesium chloride (2.20 mg, 23.0 µmol, 0.100 equiv), triethylamine (64.0 µL, 0.463 mmol, 2.00 equiv), benzaldeyde (28.0 µL, 0.278 mmol, 1.20 equiv), and trimethylsilyl chloride (44.0 µL, 0.348 mmol, 1.50 equiv). The reaction mixture was stirred for 20 h at 23 °C and filtered through a short plug of silica eluting with diethyl ether (2 × 5 mL). The filtrate was concentrated then dissolved in methanol (1.2 mL). Three drops of trifluoroacetic acid were added and the reaction mixture was stirred for 30 min at 23 °C. The product mixture was concentrated, and the residue was purified by flash-column chromatography (eluting with 15% ethyl acetate-hexane initially, grading to 30% ethyl acetate-hexane, linear gradient) to afford the *anti-aldol product analogue* **S4** as a white solid (102 mg, 82%)

$R_f$ = 0.42 (hexane/EtOAc = 8:2)

$[\alpha]_D^{20}$: –26.4 (*c* 0.24, $CHCl_3$)

$^1H$ NMR (500 MHz, $CDCl_3$) δ 7.43 – 7.38 (m, 6H, $H_{7\text{-}12}$), 7.34 – 7.29 (m, 8H, $H_{7\text{-}12}$), 7.26 – 7.20 (m, 6H, $H_{7\text{-}12}$), 4.71 (d, *J* = 7.4 Hz, 1H, $H_5$), 4.20 (app p, *J* = 7.0 Hz, 1H, $H_4$), 4.10 – 4.03 (app t, *J* = 8.6 Hz, 1H, $H_1$), 3.99 (m, 1H, $H_2$), 3.82 (dd, *J* = 9.0, 3.2 Hz, 1H, $H_1$), 3.05 (d, *J* = 7.5 Hz, 1H, –O*H*), 2.69 – 2.64 (dd, *J* = 12.8, 3.3 Hz, 1H, $H_3$), 2.27 (dd, *J* = 12.8, 9.7 Hz, 1H, $H_3$), 1.06 (d, *J* = 6.9 Hz, 3H, $H_6$).

$^{13}C$ NMR (126 MHz, $CDCl_3$) δ 176.4 (C), 153.3 (C), 144.3 (3 × C), 141.9 (C), 129.6 (6 × CH), 128.6 (2 × CH), 128.2 (6 × CH), 128.0 (CH), 127.1 (3 × CH), 126.5 (2× CH), 77.1 (CH), 67.4 (C), 66.3 ($CH_2$), 53.3 (CH), 44.2 (CH), 33.1 ($CH_2$), 14.8 ($CH_3$).

HRMS-CI (m/z): $[M + Na]^+$ calcd for $C_{33}H_{31}NO_4SNa$, 560.1866; found, 560.1868.

*Synthesis of oxazolidinone thioester **S5***:

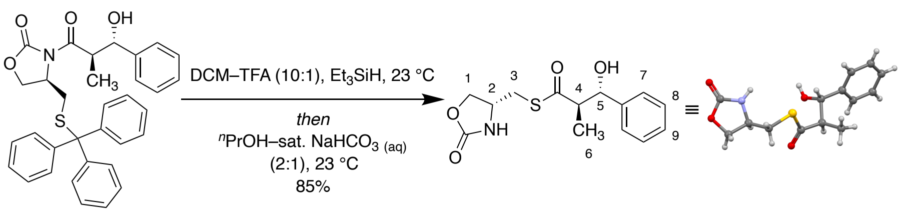


To a solution of *anti*-aldol product analogue **S4** (150 mg, 0.279 mmol, 1 equiv) in dichloromethane (0.85 mL) were added trifluoroacetic acid (85.0 µL, 1.12 mmol, 4.00 equiv) and triethylsilane (124 µL, 0.781 mmol, 2.80 equiv) at 23 °C. The reaction mixture was stirred for 30 min at 23 °C. The reaction mixture was concentrated and dried under high vacuum to afford a white solid that was used in the next step without purification.
The trityl deprotected adduct was dissolved in 1-propanol (1.4 mL) and a saturated aqueous sodium bicarbonate solution (0.70 mL). The reaction mixture was stirred for 4 h at 23 °C. The product mixture was diluted with saturated aqueous sodium chloride solution (3 mL). The diluted product mixture was transferred to a separatory funnel and extracted with ethyl acetate (3 × 5 mL). The combined organic extracts were dried over sodium sulphate and filtered. The filtrate was concentrated, and the white residue was purified by flash column chromatography (eluting with 100% dichloromethane initially, grading to 20% methanol-dichloromethane, linear gradient) to afford the *oxazolidinone thioester* **S5** as a pale-yellow oil which crystallized upon standing at 23 °C (70.2 mg, 85%).

*Note*: The absolute stereochemistry was unambiguously confirmed by X-ray crystallography. Crystals were grown from slow evaporation of deuterated chloroform.

$R_f$ = 0.40 (DCM/MeOH = 95:5)

$[\alpha]_D^{20}$: –45.2 (*c* 0.31, $CHCl_3$)

$^1$H NMR (500 MHz, $CDCl_3$) δ 7.50 – 7.28 (m, 5H, $H_{7\text{-}9}$), 5.40 (s, 1H, –N*H*), 4.80 (d, *J* = 9.0 Hz, 1H, $H_5$), 4.55 – 4.39 (m, 1H, $H_1$), 4.24 – 3.96 (m, 2H, $H_1$, $H_2$), 3.14 (app d, *J* = 5.4 Hz, 2H, $H_3$), 3.05 (app dd, *J* = 9.0, 7.0 Hz, 1H, $H_4$), 1.00 (d, *J* = 7.0 Hz, 3H, $H_6$).

$^{13}$C NMR (126 MHz, $CDCl_3$) δ 202.4 (C), 159.0 (C), 141.4 (C), 128.8 (2 × CH), 128.6 (2 × CH), 126.8 (CH), 76.7 (CH), 69.2 ($CH_2$), 56.2 (CH), 52.1 (CH), 33.0 ($CH_2$), 15.2 ($CH_3$).

HRMS-CI (m/z): $[M + H]^+$ calcd for $C_{14}H_{18}NO_4S$, 296.0951; found, 296.0955.

*Synthesis of the carboxylic acid **9***:

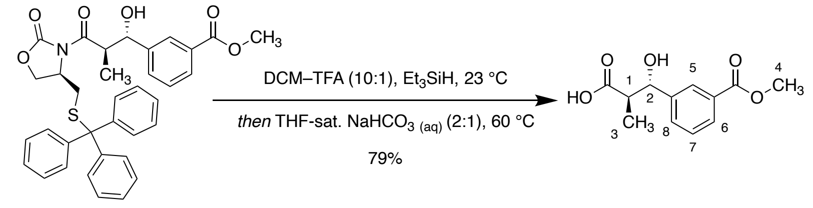


To a solution of *anti*-aldol product **7** (179 mg, 0.300 mmol, 1 equiv) in dichloromethane (0.90 mL) were added trifluoroacetic acid (92.0 µL, 1.20 mmol, 4.00 equiv) and triethylsilane (134 µL, 0.840 mmol, 2.80 equiv) at 23 °C. The reaction mixture was stirred for 30 min at 23 °C. The reaction mixture was concentrated and dried under high vacuum to afford a white solid that was used in the next step without further purification.

The trityl deprotected adduct was dissolved in tetrahydrofuran (1.5 mL) and a saturated aqueous sodium bicarbonate solution (0.75 mL). The reaction mixture was stirred for 24 h at 60 °C and cooled to 23 °C. The product mixture was quenched with 1.0 M aqueous hydrogen chloride solution (2 mL) and transferred to a separatory funnel. The diluted product mixture was extracted with ethyl acetate (3 × 10 mL). The combined organic extracts were dried over sodium sulphate and filtered. The filtrate was concentrated, and the residue was purified by flash-column chromatography (eluting with 30% ethyl acetate-hexane + 1% acetic acid initially, grading to 50% ethyl acetate-hexane + 1% acetic acid, linear gradient) to afford the *carboxylic acid* **9** as a pale-yellow oil (56.2 mg, 79%).

$R_f$ = 0.29 (hexane/EtOAc = 1:1 + 0.1% acetic acid)

$[\alpha]_D^{20}$: –33.0 (*c* 0.29, $CHCl_3$)

$^1$H NMR (500 MHz, $CDCl_3$) δ 8.01 (s, 1H, $H_5$), 7.96 (dt, *J* = 7.8, 1.5 Hz, 1H, $H_6$), 7.54 (dt, *J* = 7.7, 1.5 Hz, 1H, $H_8$), 7.42 (app t, *J* = 7.7 Hz, 1H, $H_7$), 4.80 (d, *J* = 8.7 Hz, 1H, $H_2$), 3.90 (s, 3H, $H_4$), 2.83 (app dt, *J* = 9.2, 7.1 Hz, 1H, $H_1$), 1.01 (d, *J* = 7.5 Hz, 3H, $H_3$).

$^{13}$C NMR (126 MHz, $CDCl_3$) δ 180.2 (C), 167.1 (C), 141.9 (C), 131.5 (CH), 130.4 (C), 129.5 (CH), 128.8 (CH), 128.1 (CH), 75.9 (CH), 52.4 ($CH_3$), 47.1 (CH), 14.4 ($CH_3$).

HRMS-CI (m/z): $[M + NH_4]^+$ calcd for $C_{12}H_{14}O_5NH_4$, 256.1179; found, 256.1190.

*Synthesis of the ethyl ester **10***:

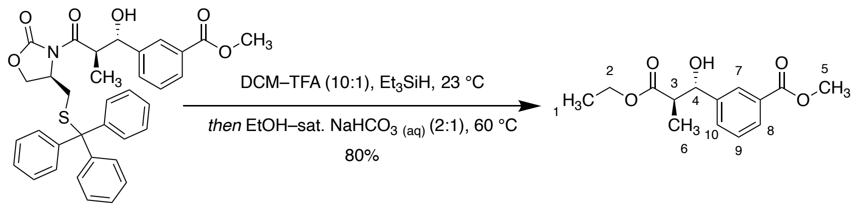


To a solution of *anti*-aldol product **7** (179 mg, 0.300 mmol, 1 equiv) in dichloromethane (0.90 mL) were added trifluoroacetic acid (92.0 µL, 1.20 mmol, 4.00 equiv) and triethylsilane (134 µL, 0.840 mmol, 2.80 equiv) at 23 °C. The reaction mixture was stirred for 30 min at 23 °C. The reaction mixture was concentrated and dried under high vacuum to afford a white solid that was used in the next step without further purification.

The trityl deprotected adduct was dissolved in ethanol (1.5 mL) and a saturated aqueous sodium bicarbonate solution (0.75 mL). The reaction mixture was stirred for 16 h at 60 °C and cooled to 23 °C. The product mixture was diluted with saturated aqueous sodium chloride solution (2 mL) and transferred to a separatory funnel. The aqueous layer was extracted with ethyl acetate (3 × 10 mL). The combined organic extracts were dried over sodium sulphate and filtered. The filtrate was concentrated, and the residue was purified by flash-column chromatography (eluting with 30% ethyl acetate-hexane initially, grading to 50% ethyl acetate-hexane, linear gradient) to afford the *ethyl ester* **10** as a clear oil (63.8 mg, 80%).

$R_f$ = 0.26 (hexane/EtOAc = 8:2)

$[\alpha]_D^{20}$: –38.1 (*c* 0.40, $CHCl_3$)

$^1$H NMR (500 MHz, $CDCl_3$) δ 8.00 (s, 1H, $H_7$), 7.95 (dt, $J$ = 7.7, 1.5 Hz, 1H, $H_8$), 7.54 (dt, $J$ = 7.7, 1.6 Hz, 1H, $H_{10}$), 7.42 (app t, $J$ = 7.7 Hz, 1H, $H_9$), 4.79 (d, $J$ = 8.1 Hz, 1H, $H_4$), 4.16 (q, $J$ = 7.1 Hz, 2H, $H_2$), 3.90 (s, 3H, $H_5$), 2.94 – 2.67 (m, 1H, $H_3$), 1.23 (t, $J$ = 7.2 Hz, 3H, $H_1$), 1.03 (d, $J$ = 7.2 Hz, 3H, $H_6$).

$^{13}$C NMR (126 MHz, $CDCl_3$) δ 175.6 (C), 166.9 (C), 142.2 (C), 131.2 (C), 130.3 (C), 129.2 (C), 128.6 (C), 127.8 (C), 75.8 (CH), 60.9 ($CH_2$), 52.2 ($CH_3$), 47.0 (CH), 14.4($CH_3$), 14.1 ($CH_3$).

HRMS-CI (m/z): $[M + Na]^+$ calcd for $C_{14}H_{18}O_5Na$, 289.1046; found, 289.1046.

*Synthesis of the amide derivative* ***11***:

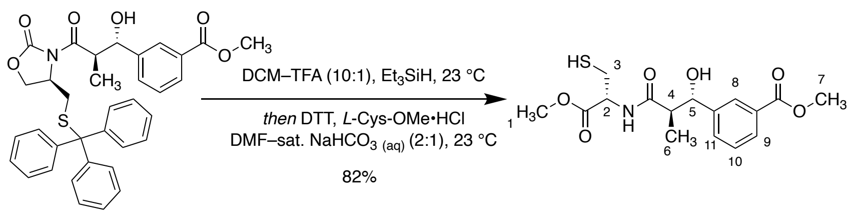


To a solution of *anti*-aldol product **7** (179 mg, 0.300 mmol, 1 equiv) in dichloromethane (0.90 mL) were added trifluoroacetic acid (92.0 µL, 1.20 mmol, 4.00 equiv) and triethylsilane (134 µL, 0.840 mmol, 2.80 equiv) at 23 °C. The reaction mixture was stirred for 30 min at 23 °C. The reaction mixture was concentrated and dried under high vacuum to afford a white solid that was used in the next step without further purification.

The trityl deprotected adduct was dissolved in *N*,*N*-dimethylformamide (1.5 mL) and a saturated aqueous sodium bicarbonate solution (0.75 mL). *DL*-dithiothreitol (DTT, 46.3 mg, 0.300 mmol, 1.00 equiv) and *L*-cysteine methyl ester hydrochloride (103 mg, 0.600 mmol, 2.00 equiv) were added and the reaction mixture was stirred for 16 h at 23 °C. The product mixture was diluted with ethyl acetate (15 mL) and transferred to a separatory funnel. The diluted product mixture was washed with water (5 × 10 mL) and saturated aqueous sodium chloride solution (10 mL). The washed organic extracts were dried over sodium sulphate and filtered. The filtrate was concentrated, and the residue was purified by flash-column chromatography (triethylamine neutralized silica, eluting with 30% ethyl acetate-hexane initially, grading to 50% ethyl acetate-hexane, linear gradient) to afford the *amide derivative* **11** as a clear oil (88.4 mg, 82%).

*Note*: An alternative workup procedure involves fully concentrating the product mixture and directly loading the residue unto the column.

$R_f$ = 0.36 (DCM/MeOH = 95:5)

$[\alpha]_D^{20}$: –2.84 (*c* 0.35, $CHCl_3$)

$^1$H NMR (500 MHz, $CDCl_3$) δ 8.01 (s, 1H, $H_8$), 7.94 (dt, *J* = 7.8, 1.4 Hz, 1H, $H_9$), 7.54 (dt, *J* = 7.7, 1.5 Hz, 1H, $H_{11}$), 7.41 (app t, *J* = 7.7 Hz, 1H, $H_{10}$), 6.96 (d, *J* = 7.9 Hz, 1H, –N*H*), 4.85 (app ddd, *J* = 7.8, 6.1, 5.1 Hz, 1H, $H_2$), 4.79 (d, *J* = 7.9 Hz, 1H. $H_5$), 3.88 (s, 3H, $H_7$), 3.76 (s, 3H, $H_1$), 3.16 – 3.11 (dd, *J* = 14.1, 5.2 Hz, 1H, $H_3$), 3.08 (dd, *J* = 14.1, 5.2 Hz, 1H, $H_3$), 2.78 – 2.65 (m, 1H, $H_4$), 1.03 (d, *J* = 7.0 Hz, 3H, $H_6$).

$^{13}$C NMR (126 MHz, $CDCl_3$) δ 175.5 (C), 171.0 (C), 167.2 (C), 142.9 (C), 131.5 (CH), 130.5 (C), 129.2 (CH), 128.7 (CH), 127.8 (CH), 76.1 (CH), 53.0 ($CH_3$), 52.4 ($CH_3$), 52.0 (CH), 48.2 ($CH_2$), 41.2 (CH), 15.1 ($CH_3$).

HRMS-CI (m/z): $[M + H]^+$ calcd for $C_{16}H_{22}NO_6S$, 356.1162; found, 356.1148.

*Synthesis of the amide **12***:

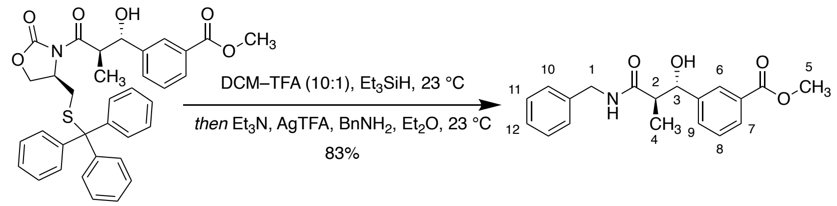


**7** **12**

To a solution of *anti*-aldol product **7** (179 mg, 0.300 mmol, 1 equiv) in dichloromethane (0.90 mL) were added trifluoroacetic acid (92.0 µL, 1.20 mmol, 4.00 equiv) and triethylsilane (134 µL, 0.840 mmol, 2.80 equiv) at 23 °C. The reaction mixture was stirred for 30 min at 23 °C. The reaction mixture was concentrated and dried under high vacuum to afford a white solid that was used in the next step without further purification.

The trityl deprotected adducted was dissolved in diethyl ether (1.5 mL) and triethylamine (420 µL, 3.00 mmol, 10 equiv) was added. The reaction mixture was stirred for 4 h at 23°C, then benzyl amine (66 µL, 0.600 mmol, 2.00 equiv) and silver trifluoroacetate (73.0 mg, 0.330 mmol, 1.10 equiv) were added. The reaction mixture was stirred for 14 h at 23 °C and filtered through celite eluting with ethyl acetate (20 mL). The filtrate was concentrated, and the residue was purified by flash column chromatography (eluting with 30% ethyl acetate-hexane initially, grading to 50% ethyl acetate-hexane, linear gradient) to afford the *amide* **12** as a pale-yellow oil (81.4 mg, 83%).

$R_f$ = 0.29 (hexane/EtOAc = 1:1)

$[\alpha]_D^{20}$: –54.8 (*c* 0.55, $CHCl_3$)

$^1$H NMR (500 MHz, $CDCl_3$) δ 8.01 (s, 1H, $H_6$), 7.97 (dt, *J* = 7.8, 1.5 Hz, 1H, $H_7$), 7.60 – 7.51 (m, 1H, $H_9$), 7.42 (app t, *J* = 7.7 Hz, 1H, $H_8$), 7.32 – 7.18 (m, 3H, $H_{11\text{-}13}$), 7.07 (app dd, *J* = 7.4, 2.1 Hz, 2H, $H_{11\text{-}13}$), 6.17 (app t, *J* = 5.8 Hz, 1H, –N*H*), 4.81 (d, *J* = 6.2 Hz, 1H, $H_3$), 4.44 (dd, *J* = 14.9, 6.1 Hz, 1H, $H_1$), 4.30 (dd, *J* = 14.9, 5.4 Hz, 1H, $H_1$), 3.91 (s, 3H, $H_5$), 2.71 – 2.49 (m, 1H, $H_2$), 1.22 (d, *J* = 7.1 Hz, 3H, $H_4$).

$^{13}$C NMR (126 MHz, $CDCl_3$) δ 175.3 (C), 167.1 (C), 143.3 (C), 137.9 (C), 131.0 (CH), 130.2 (C), 128.8 (CH), 128.6 (2 × CH), 127.6 (2 × CH), 127.4 (3 × CH), 75.9 (CH), 52.2 ($CH_3$), 47.7 (CH), 43.2 ($CH_2$), 15.6 ($CH_3$).

HRMS-CI (m/z): $[M + H]^+$ calcd for $C_{19}H_{22}NO_4$, 328.1543; found, 328.1556.

*Synthesis of the N-4-phenylbutanoyl oxazolidinone* ***13***:

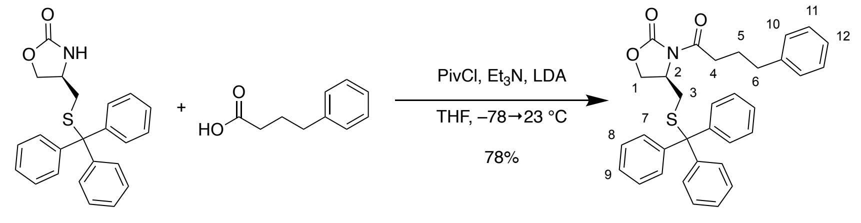


**1** **13**

To a solution of *S*-trityl auxiliary **1** (3.00 g, 8.00 mmol, 1 equiv) in tetrahydrofuran (17 mL) was added lithium diisopropylamide (2.0 M solution in tetrahydrofuran/heptane/ethylbenzene, 4.50 mL, 9.03 mmol, 1.10 equiv) dropwise at –78 °C, and the reaction mixture was stirred for 10 min at –78 °C. The dry ice-acetone bath was swapped with an ice bath and the reaction mixture was stirred at 0 °C for 10 min. The reaction mixture was recooled to –78 °C and used in the acylation step below.

In a separate round-bottomed flask, a solution of 4-phenylbutyric acid (1.60 g, 9.75 mmol, 1.20 equiv) in tetrahydrofuran (14 mL) were added triethylamine (1.70 mL, 11.9 mmol, 1.50 equiv) and pivaloyl chloride (1.30 mL, 10.8 mmol, 1.36 equiv) at –78 °C, and the reaction mixture was stirred for 10 min at –78 °C. The dry ice-acetone bath was swapped with an ice bath and the reaction mixture was stirred at 0 °C for 10 min. The reaction mixture was recooled to –78 °C and the above solution was cannulated at –78 °C. The dry ice-acetone bath was removed, and the reaction mixture was stirred for 2 h at 23 °C. The product mixture was quenched with saturated aqueous ammonium chloride solution (30 mL) and partially concentrated. The partially concentrated product mixture was transferred to a separatory funnel and extracted with ethyl acetate (3 × 30 mL). The combined organic extracts were sequentially washed with saturated aqueous citric acid (40 mL), saturated aqueous sodium bicarbonate (40 mL), and saturated aqueous sodium chloride solution (40 mL). The washed organic extracts were dried over sodium sulphate and filtered. The filtrate was concentrated to give an oily yellow residue that was purified by flash-column chromatography (15 $\mu$m silica, eluting with 5% ethyl acetate-hexane initially, grading to 15% ethyl acetate-hexane, linear gradient) to afford the *N-4-phenylbutanoyl oxazolidinone* **13** as a white solid (3.26 g, 78%).

*Note*: Purification with irregular silica (40–63 µm) did not fully remove the pivaloyl chloride derived impurities.

$R_f$ = 0.47 (hexane/EtOAc = 8:2)

$[\alpha]_D^{20}$: +25.3 (*c* 0.30, $CHCl_3$)

$^1H$ NMR (500 MHz, $CDCl_3$) δ 7.46 – 7.39 (m, 6H, $H_{7\text{-}12}$), 7.32 – 7.27 (m, 7H, $H_{7\text{-}12}$), 7.26 – 7.16 (m, 7H, $H_{7\text{-}12}$), 4.18 – 4.01 (m, 2H, $H_1$, $H_2$), 3.86 – 3.80 (m, 1H, $H_1$), 2.96 – 2.80 (m, 2H, $H_6$), 2.72

(dd, $J$ = 13.4, 2.5 Hz, 1H, $H_3$), 2.66 (t, $J$ = 7.7 Hz, 2H, $H_4$), 2.63 – 2.56 (m, 1H, $H_3$), 2.11 – 1.76 (m, 2H, $H_5$).

$^{13}$C NMR (126 MHz, $CDCl_3$) δ 172.8 (C), 153.4 (C), 144.4 (3 × C), 141.6 (C), 129.6 (6 × CH), 128.6 (2 × CH), 128.5 (2 × CH), 128.2 (6 × CH), 127.1 (3 × CH), 126.1 (CH), 67.2 (C), 66.3 ($CH_2$), 53.0 (CH), 35.2 ($CH_2$), 35.0 ($CH_2$), 33.6 ($CH_2$), 25.9 ($CH_2$).

HRMS-CI (m/z): $[M + Na]^+$ calcd for $C_{33}H_{31}NO_3SNa$, 544.1917; found, 544.1919.

*Synthesis of the α-(S)-azido oxazolidinone (S)-**14***:

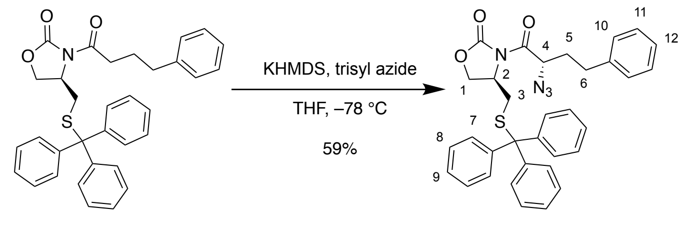


To a cooled (–78 °C) solution of potassium bis(trimethylsilyl)amide (KHMDS, 1.0 M in THF, 4.20 mL, 1.10 equiv) in tetrahydrofuran (13 mL) was added a precooled (–78 °C) solution of *N*-4-phenylbutanoyl oxazolidinone **13** (2.00 g, 3.83 mmol, 1 equiv) in tetrahydrofuran (13 mL + 1 mL rinse) with a cannula. The reaction mixture was stirred at –78 °C for 30 min. A precooled (–78 °C) solution of 2,4,6-triisopropylbenzenesulfonyl (trisyl) azide (1.48 g, 4.79 mmol, 1.25 equiv) in tetrahydrofuran (13 mL) was rapidly cannulated into the reaction mixture and stirred for 2 min at –78 °C. The product mixture was quenched with glacial acetic acid (1.0 mL, 17.6 mmol, 4.60 equiv) and immediately warmed with a warm water bath. The product mixture was stirred for 30 min at 30 °C and concentrated. The oily residue was purified by flash-column chromatography (eluting with 20% dichloromethane-hexane initially, grading to 70% dichloromethane-hexane, linear gradient) to afford the *α-(S)-azido oxazolidinone* (*S*)-**14** as a white solid (1.27 g, 59%)

$R_f$ = 0.63 (hexane/EtOAc = 8:2)

$[\alpha]_D^{20}$: +42.0 (*c* 0.24, $CHCl_3$)

$^1$H NMR (500 MHz, $CDCl_3$) δ 7.48 – 7.39 (m, 6H, $H_{7\text{-}12}$), 7.33 – 7.27 (m, 8H, $H_{7\text{-}12}$), 7.25 – 7.16 (m, 6H, $H_{7\text{-}12}$), 4.89 (dd, *J* = 9.1, 4.3 Hz, 1H, $H_4$), 4.05 (app t, *J* = 8.6 Hz, 1H, $H_1$), 3.92 (app tt, *J* = 8.4, 3.2 Hz, 1H, $H_2$), 3.85 (dd, *J* = 9.1, 3.3 Hz, 1H, $H_1$), 2.85 (app ddd, *J* = 14.6, 9.8, 5.3 Hz, 1H, $H_6$), 2.78 – 2.68 (m, 2H, $H_3$, $H_6$), 2.61 (dd, *J* = 12.7, 8.8 Hz, 1H, $H_3$), 2.11 (app dddd, *J* = 14.0, 9.8, 6.7, 4.3 Hz, 1H, $H_5$), 2.02 (app dtd, *J* = 14.3, 9.4, 5.3 Hz, 1H, $H_5$).

$^{13}$C NMR (126 MHz, $CDCl_3$) δ 170.4 (C), 152.8 (C), 144.2 (3 × C), 140.3 (C), 129.6 (6 × CH), 128.7 (2 × CH), 128.6 (2 × CH), 128.3 (6 × CH), 127.2 (3 × CH), 126.5 (C), 67.4 (C), 66.7 (CH), 60.4 ($CH_2$), 53.3 (CH), 33.2 ($CH_2$), 33.1 ($CH_2$), 32.5 ($CH_2$).

HRMS-CI (m/z): $[M + NH_4]^+$ calcd for $C_{33}H_{30}N_4O_3SNH_4$, 580.2377; found, 580.2380.

*Synthesis of the α-(S)-N-acetyl oxazolidinone (S)-**15***:

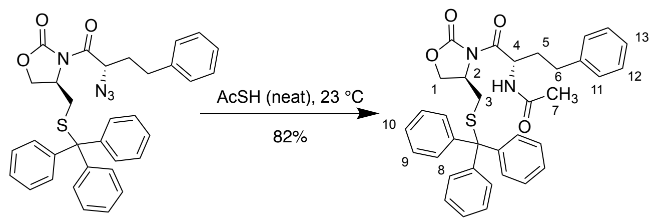


(*S*)-**14** (*S*)-**15**

A solution of α-(*S*)-azido oxazolidinone **14** (244 mg, 0.434 mmol, 1 equiv) in thioacetic acid (0.43 mL, neat) was stirred for 48 h at 23 °C. The product mixture was concentrated from toluene (2 × 10 mL). The oily residue was purified by flash-column chromatography (eluting with 100% dichloromethane initially, grading to 10% methanol-dichloromethane, linear gradient) to provide the *α-(S)-N-acetyl oxazolidinone* (*S*)-**15** as a white foamy solid (213 mg, 82%).

$R_f$ = 0.44 (DCM/MeOH = 95:5)

$[\alpha]_D^{20}$: +38.2 (*c* 0.12, $CHCl_3$)

$^1$H NMR (500 MHz, $CDCl_3$) δ 7.45 – 7.38 (m, 6H, $H_{8-13}$), 7.32 – 7.27 (m, 6H, $H_{8-13}$), 7.26 – 7.20 (m, 5H, $H_{8-13}$), 7.19 – 7.08 (m, 3H, $H_{8-13}$), 6.23 (d, *J* = 8.3 Hz, 1H, –N*H*), 5.66 (app td, *J* = 8.3, 3.7 Hz, 1H, $H_4$), 4.02 – 3.90 (m, 1H, $H_1$), 3.85 (app dd, *J* = 9.2, 3.1 Hz, 1H, $H_2$), 3.72 (app ddt, *J* = 9.5, 8.1, 3.3 Hz, 1H, $H_1$), 2.95 – 2.82 (m, 1H, $H_3$), 2.74 (app ddd, *J* = 14.1, 9.7, 6.0 Hz, 1H, $H_6$), 2.64 (app ddd, *J* = 14.1, 9.8, 6.1 Hz, 1H, $H_6$), 2.49 (dd, *J* = 12.9, 9.6 Hz, 1H, $H_3$), 2.13 (app dddd, *J* = 13.7, 9.8, 6.1, 3.8 Hz, 1H, $H_5$), 1.99 (s, 3H, $H_7$), 1.83 (app dddd, *J* = 14.2, 9.7, 8.3, 6.1 Hz, 1H, $H_5$).

$^{13}$C NMR (126 MHz, $CDCl_3$) δ 172.2 (C), 169.7 (C), 152.4 (C), 144.2 (3 × C), 141.0 (C), 129.5 (6 × CH), 128.5 (4 × CH), 128.2 (6 × CH), 127.1 (3 × CH), 126.2 (CH), 67.3 (C), 66.7 ($CH_2$), 53.4 (CH), 52.2 (CH), 34.2 ($CH_2$), 33.2 ($CH_2$), 31.9 ($CH_2$), 23.3 ($CH_3$).

HRMS-CI (m/z): $[M + NH_4]^+$ calcd for $C_{35}H_{34}N_2O_4SNH_4$, 596.2578; found, 596.2580.

*Synthesis of (S)-N-acetylhomophenylalanine **16***:

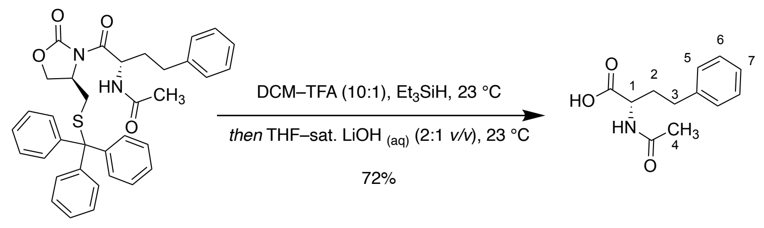


To a solution of α-(*S*)-*N*-acetyl oxazolidinone **15** (55.0 mg, 95.0 µmol, 1 equiv) in dichloromethane (0.29 mL) were added trifluoroacetic acid (29.0 µL, 0.380 mmol, 4.00 equiv) and triethylsilane (42.0 µL, 0.169 mmol, 2.80 equiv) at 23 °C. The reaction mixture was stirred for 30 min at 23 °C. The reaction mixture was concentrated and dried under high vacuum to afford a white solid that was used in the next step without further purification.
The trityl deprotected adduct was dissolved in tetrahydrofuran (0.48 mL) and a saturated aqueous lithium hydroxide solution (0.24 mL). The reaction mixture was stirred for 3.5 h at 23 °C. The product mixture was acidified with 1.0 M aqueous hydrogen chloride solution until the pH reached 2~3. The quenched product mixture was diluted with ethyl acetate (1 mL) and transferred to a separatory funnel. The layers were separated, and the aqueous layer was extracted with ethyl acetate (3 × 0.5 mL). The combined organic extracts were dried over sodium sulphate and filtered. The filtrate was concentrated, and the residue was redissolved in methanol (3 mL). The methanolic solution was filtered through a column (inner diameter = 0.7 cm; height = 3 cm; primed with methanol) of trimethyl amine acetate-functionalized silica (Si-TMA acetate) eluting with methanol (2 × 3 mL). The filtrate was discarded, and the column was washed again with 2% acetic acid–methanol (3 × 2 mL). The fraction containing product was collected and concentrated to afford *(S)-N-acetylhomophenylalanine* **16** as a white solid (15.1 mg, 72%).

$R_f$ = 0.42 (DCM/MeOH = 95:5)

$[\alpha]_D^{20}$: +18.6 (*c* 0.36, MeOH)

$^1$H NMR (500 MHz, $CD_3OD$) δ 7.27 (app t, *J* = 7.5 Hz, 2H, $H_{5-7}$), 7.18 (app dd, *J* = 14.6, 7.3 Hz, 3H, $H_{5-7}$), 4.34 (dd, *J* = 9.3, 4.7 Hz, 1H, $H_1$), 2.74 – 2.59 (m, 2H, $H_3$), 2.22 – 2.09 (m, 1H, $H_2$), 2.01 (s, 3H, $H_4$), 1.94 (app dd, *J* = 9.1, 5.3 Hz, 1H, $H_2$).

$^{13}$C NMR (126 MHz, $CD_3OD$) δ 175.7 (C), 173.5 (C), 142.2 (C), 129.5 (4 × CH), 127.1 (CH), 53.4 (CH), 34.5 ($CH_2$), 33.1 ($CH_2$), 22.4 ($CH_3$).

HRMS-CI (m/z): $[M + H]^+$ calcd for $C_{12}H_{16}NO_3$, 222.1125; found, 222.1131.

*Synthesis of (R)-N-acetylhomophenylalanine* ***S9***:

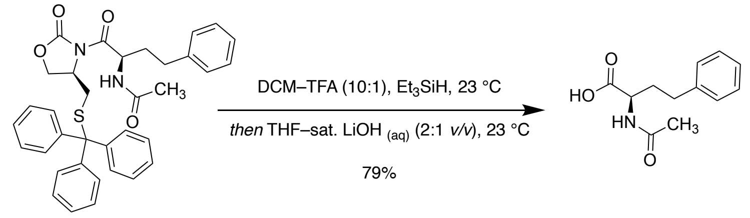


(*R*)-**15** **S9**

To a solution of α-(*R*)-*N*-acetyl oxazolidinone **15** (50.0 mg, 86.0 μmol, 1 equiv) in dichloromethane (0.26 mL) were added trifluoroacetic acid (26.5 μL, 0.346 mmol, 4.00 equiv) and triethylsilane (38.5 μL, 0.242 mmol, 2.80 equiv) at 23 °C. The reaction mixture was stirred for 30 min at 23 °C. The reaction mixture was concentrated and dried under high vacuum to afford a white solid that was used in the next step without further purification.

The trityl deprotected adduct was dissolved in tetrahydrofuran (0.43 mL) and a saturated aqueous lithium hydroxide solution (0.22 mL). The reaction mixture was stirred for 3.5 h at 23 °C. The product mixture was acidified with 1.0 M aqueous hydrogen chloride solution until the pH reached 2~3. The quenched product mixture was diluted with ethyl acetate (1 mL) and transferred to a separatory funnel. The layers were separated, and the aqueous layer was extracted with ethyl acetate (3 × 0.5 mL). The combined organic extracts were dried over sodium sulphate and filtered. The filtrate was concentrated, and the residue was redissolved in methanol (3 mL). The methanolic solution was filtered through a column (inner diameter = 0.7 cm; height = 3 cm; primed with methanol) of trimethyl amine acetate-functionalized silica (Si-TMA acetate) eluting with methanol (2 × 3 mL). The filtrate was discarded, and the column was washed again with 2% acetic acid–methanol (3 × 2 mL). The fraction containing product was collected and concentrated to afford *of (R)-N-acetylhomophenylalanine* **S9** a white solid (15.0 mg, 79%).

$[\alpha]_D^{20}$: –14.6 (*c* 0.67, MeOH)

*Synthesis of the peptide **17***:

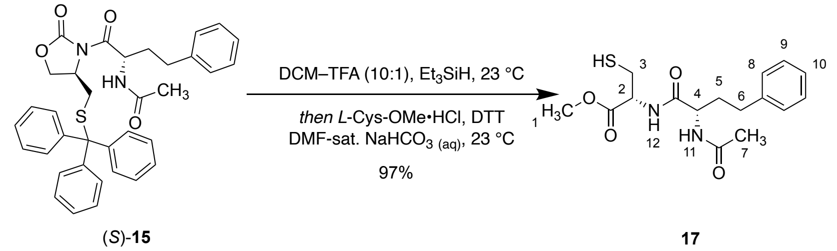

To a solution of α-(*S*)-*N*-acetyl oxazolidinone **15** (88.0 mg, 0.152 mmol, 1 equiv) in dichloromethane (0.46 mL) were added trifluoroacetic acid (47.0 μL, 0.608 mmol, 4.00 equiv) and triethylsilane (68.0 μL, 0.426 mmol mmol, 2.80 equiv) at 23 °C. The reaction mixture was stirred for 30 min at 23 °C. The reaction mixture was concentrated and dried under high vacuum to afford a white solid that was used in the next step without further purification.
The trityl deprotected adduct was dissolved in *N*,*N*-dimethylformamide (0.76 mL) and a saturated aqueous sodium bicarbonate solution (0.38 mL). *L*-Cysteine methyl ester hydrochloride (52.2 mg, 0.304 mmol, 2.00 equiv) and *DL*-dithiothreitol (DTT, 23.5 mg, 0.152 mmol, 1.00 equiv) were added then the reaction mixture was stirred for 16 h at 23 °C. The product mixture was diluted with saturated aqueous sodium chloride solution (1.5 mL) and transferred to a separatory funnel. The diluted product mixture was extracted with ethyl acetate (3 × 5 mL). The combined organic extracts were washed with water (5 × 5 mL), dried over sodium sulphate and filtered. The filtrate was concentrated, and the residue was purified by flash-column chromatography (triethylamine neutralized silica, eluting with 100% dichloromethane initially, grading to 20% methanol-dichloromethane, linear gradient) to afford the *peptide* **17** as a white solid (49.8 mg, 97%).

$R_f$ = 0.47 (DCM/MeOH = 95:5, $KMnO_4$)

$[\alpha]_D^{20}$: +15.8 (*c* 0.36, $CHCl_3$)

$^1$H NMR (500 MHz, $CDCl_3$) δ 7.32 – 7.27 (m, 2H, $H_{8\text{-}10}$), 7.23 – 7.16 (m, 3H, $H_{8\text{-}10}$), 7.04 (d, *J* = 7.8 Hz, 1H, $H_{12}$), 6.29 (d, *J* = 7.8 Hz, 1H, $H_{11}$), 4.83 (app dt, *J* = 7.8, 4.4 Hz, 1H, $H_2$), 4.58 – 4.43 (m, 1H, $H_4$), 3.79 (s, 3H, $H_1$), 2.99 (app ddd, *J* = 8.9, 4.4, 1.3 Hz, 2H, $H_3$), 2.89 – 2.56 (m, 2H, $H_6$), 2.18 (m, 1H, $H_5$), 2.10 – 1.88 (app s, 4H, $H_5$, $H_7$).

$^{13}$C NMR (126 MHz, $CDCl_3$) δ 171.7 (C), 170.6 (C), 170.3 (C), 140.8 (C), 128.7 (2 × CH), 128.5 (2 × CH), 126.4 (CH), 54.0 (CH), 53.1 (CH), 53.0 ($CH_3$), 33.9 ($CH_2$), 31.8 ($CH_2$), 26.7 ($CH_2$), 23.2 ($CH_3$).

HRMS-CI (m/z): $[M + H]^+$ calcd for $C_{16}H_{23}N_2O_4S$, 339.1373; found, 339.1379.

*Synthesis of the α-bromo oxazolidinone **S6**:*

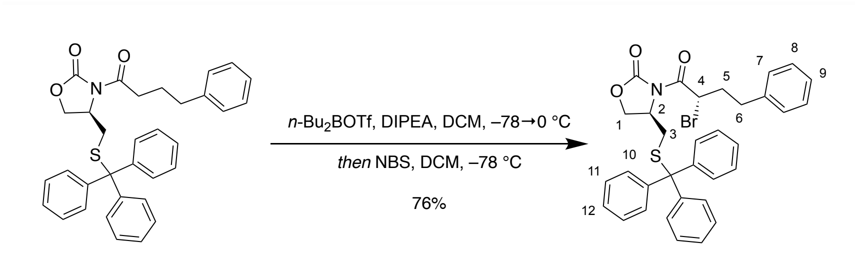


To a cooled (–78 °C) solution of *N*-4-phenylbutanoyl oxazolidinone **13** (400 mg, 0.767 mmol, 1 equiv) in dichloromethane (3.8 mL) were added dropwise *N*,*N*-diisopropylethylamine (0.160 mL, 0.921 mmol, 1.20 equiv) and di-*n*-butylboryl trifluoromethanesulfonate (0.203 mL, 0.805 mmol, 1.05 equiv) and stirred at –78 °C for 15 min then at 0 °C for 1 h. In a separate round-bottomed flask, a cooled (–78 °C) slurry of *N*-bromosuccinimide (NBS, 150 mg, 0.843 mmol, 1.10 equiv) in dichloromethane (1.9 mL) was prepared, and the precooled (–78 °C) boron enolate solution was rapidly cannulated into the slurry. The reaction mixture was stirred at –78 °C for 1.25 h. The product mixture was quenched with 0.5 M sodium bisulfate-brine (6 mL, 1:1 *v/v*). The diluted product mixture was transferred to a separatory funnel and the layers were separated. The aqueous layer was extracted with ethyl acetate (3 × 15 mL). The combined organic extracts were washed with 0.5 M sodium thiosulfate-brine (30 mL, 1:1 *v/v*) and saturated aqueous sodium chloride solution (30 mL). The washed organic extracts were dried over sodium sulfate and filtered. The filtrate was concentrated, and the residue was purified by flash-column chromatography (eluting with 10% ethyl acetate-hexane initially, grading to 40% ethyl acetate-hexane, linear gradient) to afford the *α-bromo oxazolidinone* **S6** as a white foamy solid (349 mg, 76%).

*Note*: NBS does not fully dissolve at –78 °C. The reaction mixture homogenizes upon the addition of the boron enolate.

$R_f$ = 0.51 (hexane/EtOAc = 8:2)

$[\alpha]_D^{20}$: –4.89 (*c* 0.43, $CHCl_3$)

$^1$H NMR (500 MHz, $CDCl_3$) δ 7.49 – 7.41 (m, 5H, $H_{7\text{-}12}$), 7.35 – 7.11 (m, 15H, $H_{7\text{-}12}$), 5.50 (dd, *J* = 7.9, 6.4 Hz, 1H, $H_4$), 4.09 – 3.99 (m, 1H, $H_1$), 3.96 – 3.73 (m, 2H, $H_1$, $H_2$), 2.90 – 2.77 (m, 1H, $H_6$), 2.76 – 2.52 (m, 3H, $H_3$, $H_6$), 2.43 – 2.25 (m, 2H, $H_5$).

$^{13}$C NMR (126 MHz, $CDCl_3$) δ 168.9 (C), 152.3 (C), 144.3 (3 × C), 140.1 (C), 129.6 (6 × CH), 128.7 (4 × CH), 128.3 (6 × CH), 127.1 (3 × CH), 126.5 (CH), 67.3 (C), 66.3 ($CH_2$), 53.0 (CH), 43.5 (CH), 35.5 ($CH_2$), 33.5 ($CH_2$), 32.7 ($CH_2$).
HRMS-CI (m/z): $[M + NH_4]^+$ calcd for $C_{33}H_{30}BrNO_3SNH_4$, 617.1468; found, 617.1470.

*Synthesis of the α-(R)-azido oxazolidinone (R)-**14**:*

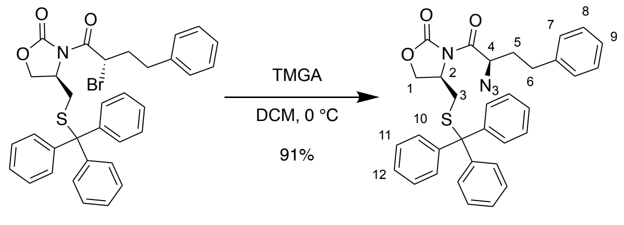


**S6** (*R*)-**14**

To a cooled (0 °C) solution of α-bromo oxazolidinone **S6** (300 mg, 0.500 mmol, 1 equiv) in dichloromethane (2.0 mL) was added a solution of tetramethylguanidinium azide (TMGA, 119 mg, 0.749 mmol, 1.50 equiv) in dichloromethane (1.0 mL) dropwise. The reaction mixture was stirred for 30 min at 0 °C and 1 h at 23 °C. The product mixture was concentrated, and the residue was purified by flash-column chromatography (eluting with 5% ethyl acetate-hexane initially, grading to 15% ethyl acetate-hexane, linear gradient) to afford the *α-(R)-azido oxazolidinone* (*R*)-**14** as a white solid (256 mg, 91%).

$R_f$ = 0.42 (hexane/EtOAc = 8:2)

$[\alpha]_D^{20}$: –2.86 (*c* 0.42, $CHCl_3$)

$^1$H NMR (500 MHz, $CDCl_3$) δ 7.43 – 7.37 (m, 5H, $H_{7\text{-}12}$), 7.32 – 7.26 (m, 9H, $H_{7\text{-}12}$), 7.25 – 7.15 (m, 6H, $H_{7\text{-}12}$), 4.90 (dd, *J* = 9.3, 4.1 Hz, 1H, $H_4$), 4.21 – 4.08 (m, 2H, $H_1$, $H_2$), 3.81 (m, 1H, $H_1$), 2.95 – 2.63 (m, 3H, $H_3$, $H_6$), 2.53 (dd, *J* = 12.9, 2.5 Hz, 1H, $H_3$), 2.27 – 2.13 (m, 1H, $H_5$), 2.05 (m, 1H, $H_5$).

$^{13}$C NMR (126 MHz, $CDCl_3$) δ 170.6 (C), 152.8 (C), 144.2 (3 × C), 140.4 (C), 129.5 (6 × CH), 128.7 (2 × CH), 128.6 (2 × CH), 128.3 (6 × CH), 127.2 (3 × CH), 126.5 (CH), 67.3 (C), 66.7 ($CH_2$), 60.6 (CH), 53.0 (CH), 33.5 ($CH_2$), 33.2 ($CH_2$), 32.5 ($CH_2$).

HRMS-CI (m/z): $[M + NH_4]^+$ calcd for $C_{33}H_{30}N_4O_3SNH_4$, 580.2377; found, 580.2377.

*Synthesis of the α-(R)-N-acetyl oxazolidinone (R)-**15***:

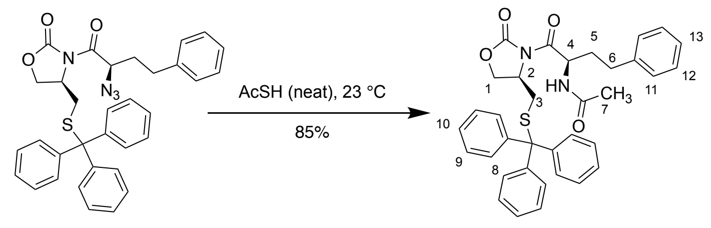


A solution of α-(*R*)-azido oxazolidinone **14** (180 mg, 0.320 mmol, 1 equiv) in thioacetic acid (0.32 mL, neat) was stirred for 48 h at 23 °C. The product mixture was concentrated from toluene (2 × 10 mL). The oily residue was purified by flash-column chromatography (eluting with 100% dichloromethane initially, grading to 10% methanol-dichloromethane, linear gradient) to provide the *α-(R)-N-acetyl oxazolidinone* (*R*)-**15** as a white foamy solid (157 mg, 85%).

$R_f$ = 0.34 (DCM/MeOH = 95:5)

$[\alpha]_D^{20}$: –4.17 (*c* 0.21, $CHCl_3$)

$^1$H NMR (500 MHz, $CDCl_3$) δ 7.43 – 7.37 (m, 7H, $H_{8-13}$), 7.30 – 7.27 (m, 5H, $H_{8-13}$), 7.25 – 7.13 (m, 8H, $H_{8-13}$), 6.09 (d, *J* = 8.2 Hz, 1H, –N*H*), 5.65 (app td, *J* = 8.3, 3.6 Hz, 1H, $H_4$), 4.25 – 4.05 (m, 2H, $H_1$, $H_2$), 3.87 – 3.73 (m, 1H, $H_1$), 2.81 – 2.64 (m, 3H, $H_3$, $H_6$), 2.50 (dd, *J* = 12.7, 3.1 Hz, 1H, $H_3$), 2.28 – 2.15 (m, 1H, $H_5$), 1.97 (s, 3H, $H_7$), 1.86 (m, 1H, $H_5$).

$^{13}$C NMR (126 MHz, $CDCl_3$) δ 172.4 (C), 170.1 (C), 152.6 (C), 144.2 (3 × C), 141.0 (C), 129.5 (6 × CH), 128.6 (2 × CH), 128.5 (2 × CH), 128.2 (6 × CH), 127.1 (3 × CH), 126.2 (CH), 67.2 (C), 66.6 ($CH_2$), 52.9 (CH), 52.2 (CH), 34.3 ($CH_2$), 33.4 ($CH_2$), 32.1 ($CH_2$), 23.2 ($CH_3$).

HRMS-CI (m/z): $[M + Na]^+$ calcd for $C_{35}H_{34}N_2O_4SNa$, 601.2131; found, 601.2155.

*Synthesis of the N-acetyl oxazolidinone thioester **S7***:

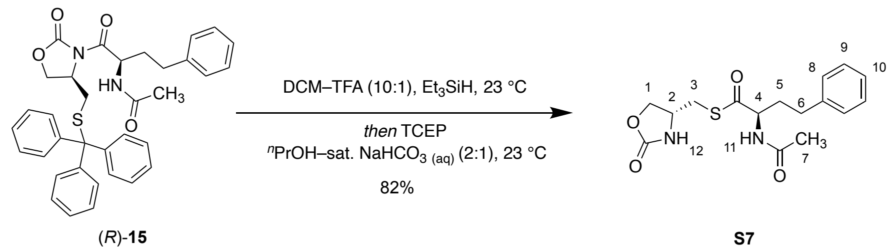


To a solution of α-(*R*)-*N*-acetyl oxazolidinone (*R*)-**15** (126 mg, 0.218 mmol, 1 equiv) in dichloromethane (0.66 mL) were added trifluoroacetic acid (67.0 µL, 0.873 mmol, 4.00 equiv) and triethylsilane (97.0 µL, 0.611 mmol, 2.80 equiv) at 23 °C. The reaction mixture was stirred for 30 min at 23 °C. The reaction mixture was concentrated and dried under high vacuum to afford a white solid that was used in the next step without further purification.

The trityl deprotected adduct was dissolved in 1-propanol (1.1 mL) and saturated aqueous sodium bicarbonate solution (0.55 mL). Tris(2-carboxyethyl)phosphine (6.30, 22.0 µmol, 10 mol%) was added and the reaction mixture was stirred for 16 h at 23 °C. The product mixture was diluted with saturated aqueous sodium chloride solution (2 mL). The diluted product mixture was transferred to a separatory funnel and the aqueous layer was extracted with ethyl acetate (3 × 5 mL). The combined organic extracts were dried over sodium sulphate and filtered. The filtrate was concentrated, and the white residue was purified by flash-column chromatography (eluting with 100% dichloromethane initially, grading to 5% methanol-dichloromethane, linear gradient) to afford the *oxazolidinone thioester* **S7** as a white solid (60.3 mg, 82%).

$R_f$ = 0.27 (DCM/MeOH = 95:5)

$[\alpha]_D^{20}$: –6.77 (*c* 0.35, $CHCl_3$)

$^1$H NMR (500 MHz, $CDCl_3$) δ 7.34 – 7.29 (m, 2H, $H_{8\text{-}10}$), 7.24 – 7.08 (m, 3H, $H_{8\text{-}10}$), 5.83 (d, $J$ = 8.2 Hz, 1H, $H_{11}$), 5.20 (s, 1H, $H_{12}$), 4.70 (app td, $J$ = 8.5, 4.6 Hz, 1H, $H_4$), 4.54 – 4.43 (m, 1H, $H_1$), 4.11 – 4.02 (m, 2H, $H_1$, $H_2$), 3.20 (dd, $J$ = 14.2, 4.8 Hz, 1H, $H_3$), 2.94 (dd, $J$ = 14.1, 5.0 Hz, 1H, $H_3$), 2.70 (app t, $J$ = 7.6 Hz, 2H, $H_6$), 2.33 – 2.19 (m, 1H, $H_5$), 2.01 (s, 3H, $H_7$), 2.00 – 1.93 (m, 1H, $H_5$)

$^{13}$C NMR (126 MHz, $CDCl_3$) δ 200.5 (C), 171.1 (C), 159.4 (C), 140.4 (C), 128.8 (2 × CH), 128.6 (2 × CH), 126.6 (CH), 69.2 ($CH_2$), 59.5 (CH), 51.8 (CH), 33.7 ($CH_2$), 33.1 ($CH_2$), 32.0 ($CH_2$), 23.0 ($CH_3$).

HRMS-CI (m/z): $[M + H]^+$ calcd for $C_{16}H_{21}N_2O_4S$, 337.1217; found, 337.1230.

*Synthesis of the ketone **18***:

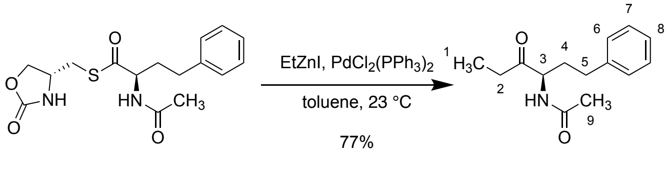


To a solution of oxazolidinone thioester **S7** (30.0 mg, 89.0 μmol, 1 equiv) in toluene (0.30 mL) were added bis(triphenylphosphine)palladium(II) dichloride (6.30 mg, 9.00 μmol, 10 mol%). Ethylzinc iodide (0.90 M in THF, 0.250 mL, 0.233 mmol, 2.50 equiv) was added to the suspension and the reaction mixture was stirred for 15 minutes at 23 °C. The product mixture was diluted with diethyl ether (3 mL) and filtered through a pad of celite. The filtrate was washed with 1.0 M aqueous hydrogen chloride solution (2 mL), saturated aqueous sodium bicarbonate solution (2 mL), and saturated aqueous sodium chloride solution (2 mL). The washed organic extracts were dried over sodium sulfate and filtered. The filtrate was concentrated, and the oily residue was purified by flash-column chromatography (eluting with 100% dichloromethane initially, grading to 10% methanol-dichloromethane, linear gradient) to afford the *ketone* **18** as a brown-yellow oil (16.1 mg, 77%).

*Note 1*: Traces of aromatic impurities are observed in the $^{1}$H NMR.

*Note 2*: The color of the reaction mixture changes from orange to black upon completion.

$R_f$ = 0.43 (DCM/MeOH = 95:5, $KMnO_4$)

$[\alpha]_D^{20}$: –48.0 (*c* 0.16, $CHCl_3$)

$^{1}$H NMR (500 MHz, $CDCl_3$) δ 7.22 (m, 2H, $H_{6\text{-}8}$), 7.16 – 7.11 (m, 1H, $H_{6\text{-}8}$), 7.10 – 7.06 (m, 2H, $H_{6\text{-}8}$), 6.13 (d, *J* = 7.4 Hz, 1H, –N*H*), 4.67 (app td, *J* = 7.3, 4.4 Hz, 1H, $H_3$), 2.66 – 2.32 (m, 4H, $H_2$, $H_5$), 2.19 (app dddd, *J* = 14.4, 10.5, 6.5, 4.3 Hz, 1H, $H_4$), 1.93 (s, 3H, $H_9$), 1.81 (app dddd, *J* = 14.0, 10.0, 7.2, 5.4 Hz, 1H, $H_4$), 1.00 (t, *J* = 7.3 Hz, 3H, $H_1$).

$^{13}$C NMR (126 MHz, $CDCl_3$) δ 209.6 (C), 170.2 (C), 140.8 (C), 128.7 (2 × CH), 128.4 (2 × CH), 126.4 (CH), 57.8 (CH), 33.3 (2 × $CH_2$), 31.5 ($CH_2$), 23.3 ($CH_3$), 7.7 ($CH_3$).

HRMS-CI (m/z): $[M + H]^+$ calcd for $C_{14}H_{20}NO_2$, 234.1489; found, 234.1480.

*Synthesis the syn-aldol product **19***:

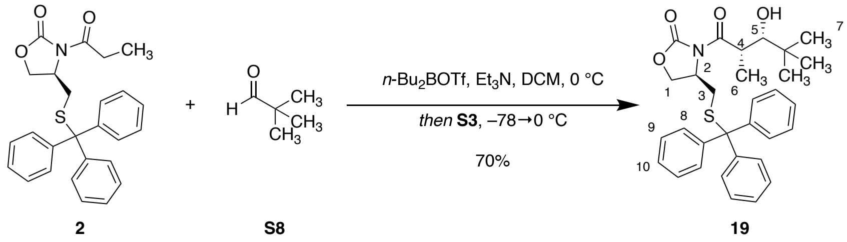


To a solution of *N*-propionyl trityl auxiliary **2** (500 mg, 1.16 mmol, 1 equiv) in dichloromethane (4.6 mL) were sequentially added dropwise di-*n*-butylboryl trifluoromethanesulfonate ($\rho$ = 1.086 g mL$^{-1}$; 0.640 mL, 2.55 mmol, 2.20 equiv) and triethylamine (0.390 mL, 2.78 mmol, 2.40 equiv) over 10 minutes (syringe pump; 5 min each) at 0 °C. The reaction mixture was stirred at 0 °C for 1 h then cooled to –78 °C. Trimethylacetaldehyde (0.200 mL, 1.74 mmol, 1.50 equiv) was added dropwise over 5 min (syringe pump) and the reaction mixture was stirred at –78 °C for 30 min and warmed to 0 °C over 2 h. The product mixture was quenched at 0 °C with pH 7 phosphate buffer (1.4 mL) and dropwise addition of methanol (4.2 mL). 30% hydrogen peroxide–methanol (4.2 mL, 2:1) was added over 10 minutes (syringe pump) and the quenched product mixture was stirred at 0 °C for 30 min. The quenched product mixture was transferred to a separatory funnel and the layers were separated. The aqueous layer was diluted with saturated aqueous sodium chloride solution (15 mL) and then extracted with ethyl acetate (3 × 20 mL). The combined organic extracts were washed with 1.0 M aqueous hydrogen chloride solution (25 mL), saturated aqueous solution of sodium bicarbonate (25 mL) and saturated aqueous sodium chloride solution (25 mL). The washed organic extracts were dried over sodium sulphate and filtered. The filtrate was concentrated to provide a yellow residue that was purified by flash-column chromatography (eluting with 10% ethyl acetate-hexane initially, grading to 30% ethyl acetate-hexane, linear gradient) to afford the *syn-aldol product* **19** as a white solid (417 mg, 70%).

$R_f$ = 0.45 (hexane/EtOAc = 8:2)

$[\alpha]_D^{20}$: +8.52 (*c* 0.25, $CHCl_3$)

$^1$H NMR (500 MHz, $CDCl_3$) δ 7.46 – 7.37 (m, 6H, $H_8$), 7.30 (dd, *J* = 8.4, 6.8 Hz, 6H, $H_9$), 7.25 – 7.18 (m, 3H, $H_{10}$), 4.27 – 4.05 (m, 2H, $H_{1,2}$), 4.00 (qd, *J* = 7.0, 3.0 Hz, 1H, $H_5$), 3.92 – 3.72 (m, 1H, $H_1$), 3.58 (app dd, *J* = 3.9, 3.0 Hz, 1H, $H_4$), 2.72 – 2.61 (m, 1H, $H_3$), 2.62 – 2.50 (m, 1H, $H_3$), 2.47 (app d, *J* = 3.9 Hz, 1H, –O*H*), 1.24 (d, *J* = 7.0 Hz, 3H, $H_6$), 0.93 (s, 9H, $H_7$).

$^{13}$C NMR (126 MHz, $CDCl_3$) δ 178.1 (C), 152.7 (C), 144.2 (3 × C), 129.5 (6 × CH), 128.1 (6 × CH), 127.0 (3 × CH), 77.5 (CH), 67.1 (C), 66.0 ($CH_2$), 52.9 (CH), 38.8 (CH), 35.5 (C), 33.3 ($CH_2$), 26.8 ($CH_3$), 12.8 (3 × $CH_3$)

HRMS-CI (m/z): $[M + Na]^+$ calcd for $C_{31}H_{35}NO_4SNa$, 540.2179; found, 540.2185.

*Synthesis of the oxazolidinone thioester **20***:

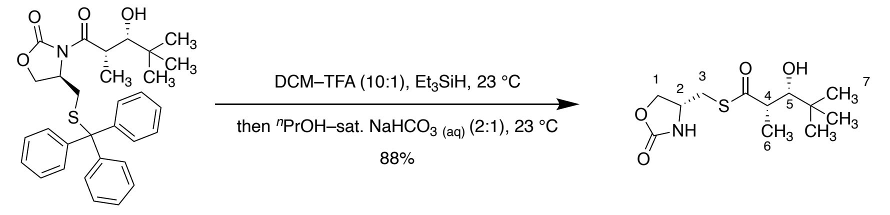


**19** **20**

To a solution of *syn*-aldol product **19** (970 mg, 1.87 mmol, 1 equiv) in dichloromethane (5.7 mL) were added trifluoroacetic acid (574 µL, 7.50 mmol, 4.00 equiv) and triethylsilane (835 µL, 5.25 mmol, 2.80 equiv at 23 °C. The reaction mixture was stirred for 30 min at 23 °C. The reaction mixture was concentrated and dried under high vacuum to afford a white solid that was used in the next step without purification.

The trityl deprotected adduct was dissolved in 1-propanol (9.4 mL) and saturated aqueous sodium bicarbonate solution (4.7 mL). The reaction mixture was stirred for 4 h at 23 °C. The product mixture was diluted with saturated aqueous sodium chloride solution (15 mL). The diluted product mixture was transferred to a separatory funnel and extracted with ethyl acetate (3 × 25 mL). The combined organic extracts were dried over sodium sulphate and filtered. The filtrate was concentrated, and the white residue was purified by flash column chromatography (eluting with 100% dichloromethane initially, grading to 20% methanol-dichloromethane, linear gradient) to afford the *oxazolidinone thioester* **20** as an oil which solidified upon standing at 23 °C (456 mg, 88%).

$R_f$ = 0.35 (DCM/MeOH = 95:5)

$[\alpha]_D^{20}$: –17.1 (*c* 0.36, $CHCl_3$)

$^1H$ NMR (500 MHz, $CDCl_3$) δ 5.46 (s, 1H, –N*H*), 4.59 – 4.41 (m, 1H. $H_1$), 4.23 – 3.99 (m, 2H, $H_{1,2}$), 3.67 (d, *J* = 3.2 Hz, 1H, $H_5$), 3.51 (s, 1H,–O*H*), 3.27 – 3.15 (m, 1H, $H_3$), 2.95 (ddd, *J* = 16.7, 8.9, 3.9 Hz, 2H, $H_3$, $H_4$), 1.25 (d, *J* = 6.9 Hz, 3H, $H_6$), 0.96 (s, 9H, $H_7$).

$^{13}C$ NMR (126 MHz, $CDCl_3$) δ 203.5 (C), 159.3 (C), 78.3 (CH), 69.2 ($CH_2$), 51.9 (CH), 50.5 (CH), 36.2 (C), 33.3 ($CH_2$), 26.8 ($CH_3$), 12.6 (3 × $CH_3$).

HRMS-CI (m/z): $[M + H]^+$ calcd for $C_{12}H_{22}NO_4S$, 276.1264; found, 276.1268.

*Synthesis of the alkyne **21***:

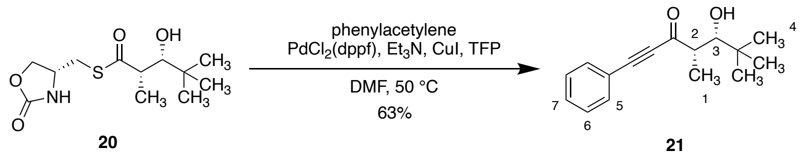


To a solution of oxazolidinone thioester **20** (120 mg, 0.436 mmol, 1 equiv) in degassed (freeze-pump-thaw; 3 cycles) *N*,*N*-dimethylformamide (0.75 mL) were added [1,1' bis(diphenyl phosphino)ferrocene] palladium(II) dichloride ($PdCl_2$(dppf), 31.9 mg, 44 µmol, 10 mol%), copper (I) iodide (141 mg, 0.741 mmol, 1.70 equiv), tri-2-furylphosphine (25.3 mg, 0.109 mmol, 0.25 equiv), triethylamine (61.0 µL, 0.436 mmol, 1.00 equiv), and phenylacetylene (96.0 µL, 0.872 mmol, 2.000 equiv). The reaction mixture was stirred for 6 h at 50 °C and cooled to 23 °C. The product mixture was quenched with water (1 mL) and diluted with diethyl ether (2 mL). Celite (50 mg) was added and stirred for 5 min at 23 °C. The diluted product mixture was filtered through celite eluting with diethyl ether (20 mL). The filtrate was transferred to a separatory funnel and sequentially washed with 1 M aqueous hydrogen chloride solution (10 mL) and water (5 × 5 mL). The washed organic extracts were dried over sodium sulphate and filtered. The filtrate was concentrated, and the dark residue was purified by flash-column chromatography (eluting with 10% ethyl acetate-hexane initially, grading to 20% ethyl acetate-hexane, linear gradient) to afford the *alkyne* **21** as a brown oil (67.4 mg, 63%).

$R_f$ = 0.58 (hexane/EtOAc = 8:2)

$[\alpha]_D^{20}$: –15.4 (*c* 0.24, $CHCl_3$)

$^1$H NMR (500 MHz, $CDCl_3$) δ 7.62 – 7.54 (m, 2H, $H_5$), 7.51 – 7.43 (m, 1H, $H_7$), 7.42 – 7.34 (m, 2H, $H_6$), 3.92 (app t, *J* = 3.2 Hz, 1H, $H_3$), 2.97 (qd, *J* = 7.1, 3.6 Hz, 1H, $H_2$), 2.11 – 1.97 (br s, 1H, –O*H*), 1.33 (d, *J* = 7.1 Hz, 3H, $H_1$), 0.99 (s, 9H, $H_4$).

$^{13}$C NMR (126 MHz, $CDCl_3$) δ 191.8 (C), 133.2 (2 × CH), 130.9 (2 × CH), 128.8 (CH), 120.0 (C), 92.2 (C), 87.4 (C), 77.2 (CH), 50.3 (CH), 35.9 (C), 26.8 ($CH_3$), 11.7 (3 × $CH_3$).

HRMS-CI (m/z): $[M + H]^+$ calcd for $C_{16}H_{21}O_2$, 245.1536; found, 245.1542.

*Synthesis of the dihydropyrone **22***:

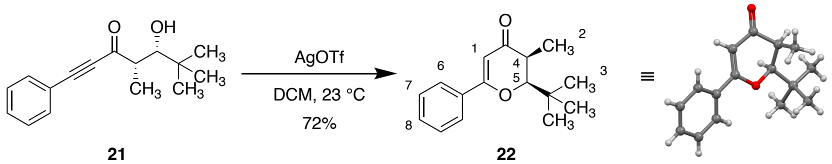


To a solution of alkyne **21** (36.0 mg, 0.147 mmol, 1 equiv) in dichloromethane (15 mL) was added silver trifluoromethanesulfonate (41.6 mg, 0.162 mmol, 1.10 equiv) at 23 °C under the strict exclusion of light. The reaction mixture was stirred for 18 h at 23 °C and filtered through a short plug of silica eluting with ethyl acetate (2 × 5 mL). The filtrate was concentrated, and the residue was purified by flash-column chromatography (eluting with 10% ethyl acetate-hexane initially, grading to 20% ethyl acetate-hexane, linear gradient) to afford the *dihydropyrone* **22** as a white crystalline solid (25.8 mg, 72%). The spectroscopic data are in agreement with the literature.[34]

*Note*: The round-bottom flask was covered with an aluminum foil.

The absolute stereochemistry was unambiguously confirmed by X-ray crystallography. Crystals were grown from slow evaporation of dichloromethane.

$R_f$ = 0.31 (hexane/EtOAc = 9:1)

$^1$H NMR (500 MHz, $CDCl_3$) δ 7.81 – 7.72 (m, 2H, $H_{6\text{-}8}$), 7.54 – 7.42 (m, 3H, $H_{6\text{-}8}$), 5.94 (s, 1H, $H_1$), 4.14 (d, $J$ = 2.6 Hz, 1H, $H_5$), 2.63 – 2.56 (m, 1H, $H_4$), 1.20 (d, $J$ = 7.3 Hz, 3H, $H_2$), 1.17 (s, 9H, $H_3$).

*Synthesis of the N-cinnamoyl oxazolidinone* **23**:

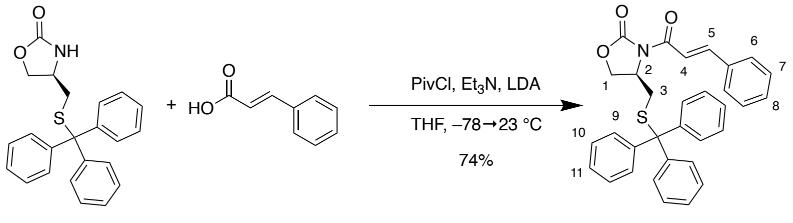


To a solution of *S*-trityl auxiliary **1** (4.00 g, 10.6 mmol, 1 equiv) in tetrahydrofuran (23 mL) was added lithium diisopropylamide (2.0 M solution in tetrahydrofuran/heptane/ethylbenzene, 8.00 mL, 12.8 mmol, 1.10 equiv) dropwise at –78 °C, and the reaction mixture was stirred for 10 min at –78 °C. The dry ice-acetone bath was swapped with an ice bath and the reaction mixture was stirred at 0 °C for 10 min. The reaction mixture was recooled to –78 °C and used in the acylation step below.

In a separate round-bottomed flask, a solution of cinnamic acid (1.89 g, 12.8 mmol, 1.20 equiv) in tetrahydrofuran 19 mL) were added triethylamine (2.20 mL, 16.0 mmol, 1.50 equiv) and pivaloyl chloride (1.80 mL, 14.9 mmol, 1.36 equiv) at –78 °C, and the reaction mixture was stirred for 10 min at –78 °C. The dry ice-acetone bath was swapped with an ice bath and the reaction mixture was stirred at 0 °C for 10 min. The reaction mixture was recooled to –78 °C and the above solution was cannulated at –78 °C. The dry ice-acetone bath was removed, and the reaction mixture was stirred at 23 °C for 2 h. The product mixture was quenched with saturated aqueous ammonium chloride solution (40 mL) and partially concentrated. The partially concentrated product mixture was transferred to a separatory funnel and extracted with ethyl acetate (3 × 50 mL). The combined organic extracts were sequentially washed with saturated aqueous citric acid (60 mL), saturated aqueous sodium bicarbonate (70 mL), and saturated aqueous sodium chloride solution (70 mL). The washed organic extracts were dried over sodium sulphate and filtered. The filtrate was concentrated to give an oily yellow residue what was purified by flash-column chromatography (15 $\mu$m silica, eluting with 5% ethyl acetate-hexane initially, grading to 15% ethyl acetate-hexane, linear gradient) to afford the *N-cinnamoyl oxazolidinone* **23** as a light-yellow solid (4.20 g, 74%).

*Note*: Purification with irregular silica (40-63 μm) did not fully remove pivaloyl chloride derived impurities.

$R_f$ = 0.56 (hexane/EtOAc = 8:2)

$[\alpha]_D^{20}$: –1.10 (*c* 0.29, $CHCl_3$)

$^1$H NMR (500 MHz, $CDCl_3$) δ 7.81 (app s, 2H, $H_4$, $H_5$), 7.63 – 7.57 (m, 2H, $H_{6\text{-}8}$), 7.48 – 7.40 (m, 6H, $H_9$), 7.39 (dd, $J$ = 5.1, 1.8 Hz, 3H, $H_{6\text{-}8}$), 7.31 (dd, $J$ = 8.4, 6.8 Hz, 6H, $H_{10}$), 7.25 – 7.17 (m,

3H, $H_{11}$), 4.22 (app tt, *J* = 8.5, 3.3 Hz, 1H, $H_2$), 4.19 – 4.09 (m, 1H, $H_1$), 3.92 (app dd, *J* = 8.9, 3.2 Hz, 1H, $H_1$), 2.89 (dd, *J* = 12.7, 3.2 Hz, 1H, $H_3$), 2.63 (dd, *J* = 12.7, 8.8 Hz, 1H, $H_3$).

$^{13}$C NMR (126 MHz, $CDCl_3$) δ 165.0 (C), 153.5 (C), 146.5 (CH), 144.4 (3 × C), 134.7 (C), 130.8 (CH), 129.6 (6 × CH), 129.0 (2 × CH), 128.8 (2 × CH), 128.3 (6 × CH), 127.1 (3 × CH), 117.1 (CH), 67.3 (C), 66.3 ($CH_2$), 53.3 (CH), 33.7 ($CH_2$).

HRMS-CI (m/z): $[M + Na]^+$ calcd for $C_{32}H_{27}NO_3SNa$, 528.1604; found, 528.1610.

*Synthesis of the terminal alkene **24***:

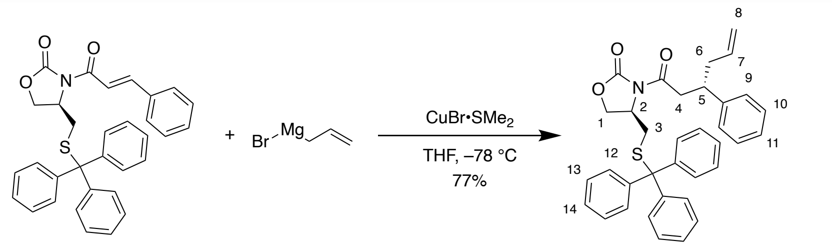


To a suspension of freshly prepared copper (I) bromide dimethyl sulfide complex (CuBr•$SMe_2$, 488 mg, 2.37 mmol, 1.50 equiv) in tetrahydrofuran (10 mL) was added a solution of allylmagnesium bromide (1.0 M solution in diethyl ether, 4.10 mL, 4.11 mmol, 2.60 equiv) at –78 °C. The reaction mixture was stirred for 1.5 h at –78 °C. A solution of *N*-cinnamoyl oxazolidinone **23** (800 mg, 1.58 mmol, 1 equiv) in tetrahydrofuran (14 mL) was cannulated into the reaction mixture and stirred for 2.5 h at –78 °C. The product mixture was quenched with saturated aqueous ammonium chloride solution (25 mL). The quenched product mixture was transferred to a separatory funnel and the layers were separated. The aqueous layer was extracted with ethyl acetate (3 × 20 mL). The combined organic extracts were dried over sodium sulphate and filtered. The filtrate was concentrated to afford a pale-yellow foam that was purified by flash-column chromatography (eluting with 10% ethyl acetate-hexane initially, grading to 20% ethyl acetate-hexane, linear gradient) to afford the *terminal alkene* **24** as a white solid (670 mg, 77%).

$R_f$ = 0.60 (hexane/EtOAc = 8:2)

$[\alpha]_D^{20}$: +49.7 (*c* 0.27, $CHCl_3$)

$^1$H NMR (500 MHz, $CDCl_3$) δ 7.42 – 7.37 (m, 6H, $H_{12}$), 7.29 (dd, *J* = 8.4, 6.9 Hz, 6H, $H_{13}$), 7.26 – 7.21 (m, 5H, $H_{9\text{-}11,14}$), 7.19 – 7.15 (m, 3H, $H_{9\text{-}11}$), 5.74 – 5.58 (m, 1H, $H_7$), 5.08 – 4.84 (m, 2H, $H_8$), 3.97 – 3.85 (m, 2H, $H_{1,2}$), 3.77 – 3.65 (m, 1H, $H_1$), 3.33 – 3.19 (m, 2H, $H_{4,5}$), 3.19 – 3.07 (m, 1H, $H_4$), 2.68 – 2.61 (m, 1H, $H_3$), 2.49 – 2.29 (m, 3H, $H_{3,6}$).

$^{13}$C NMR (126 MHz, $CDCl_3$) δ 171.7 (C), 153.3 (C), 144.4 (3 × CH), 143.6 (CH), 136.2 (CH), 129.6 (6 × CH), 128.5 (2 × CH), 128.2 (6 × CH), 127.8 (2 × CH), 127.1 (3 × CH), 126.7 (CH), 117.1 ($CH_2$), 67.3 (CH), 66.3 ($CH_2$), 53.0 (CH), 41.6 (CH), 41.2 ($CH_2$), 41.0 ($CH_2$), 33.4 ($CH_2$).

HRMS-CI (m/z): $[M + Na]^+$ calcd for $C_{35}H_{33}NO_3SNa$, 570.2073; found, 570.2077.

*Synthesis of the oxazolidinone thioester **25***:

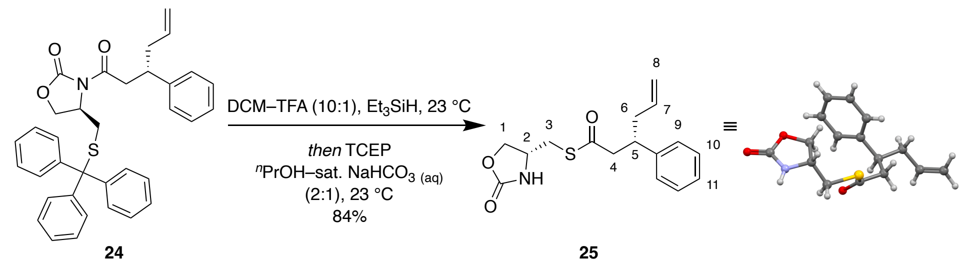


To a solution of terminal alkene **24** (367 mg, 0.670 mmol, 1 equiv) in dichloromethane (2.0 mL) were added trifluoroacetic acid (0.200 mL, 2.68 mmol, 4.00 equiv) and triethylsilane (0.300 mL, 1.88 mmol, 2.80 equiv) at 23 °C. The reaction mixture was stirred for 30 min at 23 °C. The reaction mixture was concentrated and dried under high vacuum to afford a white solid that was used in the next step without purification.

The trityl deprotected adduct was dissolved in 1-propanol (3.4 mL) and saturated aqueous sodium bicarbonate solution (1.7 mL). Tris(2-carboxyethyl)phosphine (19.2 mg, 67.0 μmol, 10 mol%) was added and the reaction mixture was stirred for 20 h at 23 °C. The product mixture was diluted with saturated aqueous sodium chloride solution (12 mL). The diluted product mixture was transferred to a separatory funnel and the layers were separated. The aqueous layer was extracted with ethyl acetate (3 × 10 mL). The combined organic extracts were dried over sodium sulphate and filtered. The filtrate was concentrated, and the white residue was purified by flash-column chromatography (eluting with 100% dichloromethane initially, grading to 20% methanol-dichloromethane, linear gradient) to afford the *oxazolidinone thioester* **25** as a pale-yellow oil which solidified upon standing at 23 °C (171 mg, 84%).

*Note*: The absolute stereochemistry was unambigously confirmed by X-ray crystallography. Crystals were obtained from slow evaporation of deuterated chloroform.

$R_f$ = 0.40 (DCM/MeOH = 95:5)

$[\alpha]_D^{20}$: +37.5 (*c* 0.38, $CHCl_3$)

$^1$H NMR (500 MHz, $CDCl_3$) δ 7.31 (app t, *J* = 7.4 Hz, 2H, $H_{10}$), 7.24 (app t, *J* = 7.4 Hz, 1H, $H_{11}$), 7.19 – 7.13 (m, 2H, $H_9$), 5.65 (app ddt, *J* = 17.1, 10.3, 7.0 Hz, 1H, $H_7$), 5.08 (s, 1H, –N*H*), 5.05 – 4.98 (m, 2H, $H_8$), 4.35 (app t, *J* = 8.6 Hz, 1H, $H_1$), 3.93 (dd, *J* = 9.0, 5.1 Hz, 1H, $H_1$), 3.85 – 3.73 (m, 1H, $H_2$), 3.24 (app dq, *J* = 9.5, 7.0 Hz, 1H, $H_5$), 3.04 (dd, *J* = 14.0, 5.5 Hz, 1H, $H_3$), 2.95 (dd, *J* = 14.8, 5.9 Hz, 1H, $H_4$), 2.90 – 2.76 (m, 2H, $H_{3,4}$), 2.40 (app qt, *J* = 14.2, 7.2 Hz, 2H, $H_6$).

$^{13}$C NMR (126 MHz, $CDCl_3$) δ 197.5 (C), 158.7 (C), 142.6 (C), 135.6 (CH), 128.7 (2 × CH), 127.7 (2 × CH), 127.1 (CH), 117.5 (CH), 69.1 ($CH_2$), 51.7 (CH), 50.0 ($CH_2$), 42.8 (CH), 40.7 ($CH_2$), 33.3 ($CH_2$).

HRMS-CI (m/z): $[M + H]^+$ calcd for $C_{16}H_{20}NO_3S$, 306.1158; found, 306.1166.

*Synthesis of the carbocycle **26***:

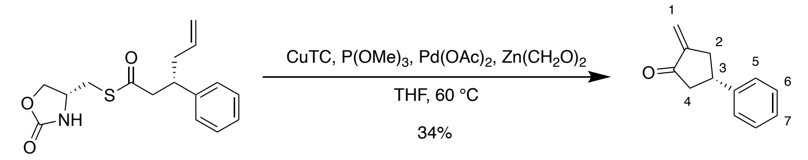


To a solution of the oxazolidinone thioester **25** (100 mg, 0.327 mmol, 1 equiv) in degassed (freeze-pump-thaw; 3 cycles) tetrahydrofuran (2.7 mL) were added palladium acetate (7.40 mg, 33.0 µmol, 5 mol%), copper(I) thiophene-2-carboxylate (CuTC, 200 mg, mmol, 3.20 equiv), zinc formate (50.9 mg, 0.327 mmol, 1.00 equiv) and trimethyl phosphite (39.0 µL, 0.327 mmol, 1.00 equiv). The brown reaction mixture was stirred for 2 h at 60 °C and cooled to 23 °C. The product mixture was quenched with saturated aqueous sodium bicarbonate (6 mL). The quenched product mixture was transferred to a separatory funnel and the layers were separated. The aqueous layer was extracted with ethyl acetate (3 × 10 mL). The combined organic extracts were washed with saturated aqueous sodium chloride solution (20 mL). The washed organic extracts were dried over sodium sulphate and filtered through celite. The filtrate was concentrated and the oily residue was purified by flash-column chromatography (eluting with 5% ethyl acetate-hexane initially, grading to 20% ethyl acetate-hexane, linear gradient) to afford the *carbocycle* **26** as a pale-yellow oil (19.1 mg, 34%). The spectroscopic data are in agreement with the literature.[6]

$^1$H NMR (500 MHz, $CDCl_3$) δ 7.35 (app dd, *J* = 8.3, 7.0 Hz, 2H, $H_{5\text{-}7}$), 7.26 (app td, *J* = 5.1, 2.3 Hz, 3H, $H_{5\text{-}7}$), 6.07 (s, 1H, $H_1$), 5.38 (s, 1H, $H_1$), 3.42 (app tt, *J* = 10.0, 7.5 Hz, 1H, $H_3$), 3.14 (app ddt, *J* = 16.4, 7.5, 1.9 Hz, 1H, $H_2$), 2.88 – 2.77 (m, 1H, $H_4$), 2.75 (app ddd, *J* = 12.7, 6.2, 3.0 Hz, 1H, $H_2$), 2.53 (app dd, *J* = 18.0, 10.4 Hz, 1H, $H_4$).

$^{13}$C NMR (126 MHz, $CDCl_3$) δ 205.7 (C), 144.7 (C), 143.4 (C), 128.9 (2 × CH), 126.9 (2 × CH), 126.8 (CH), 117.6 ($CH_2$), 46.1 ($CH_2$), 39.1 (CH), 38.2 ($CH_2$).

*Synthesis of the alkylation product* **27**:

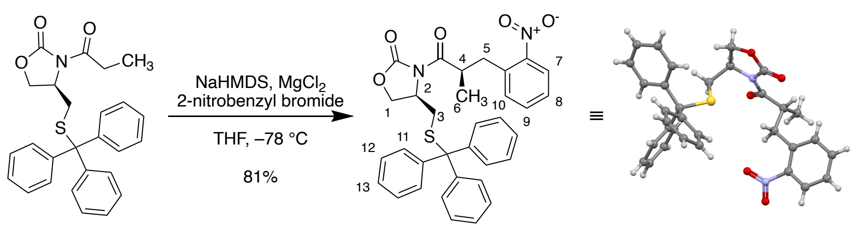


**2** **27**

To a solution of *N*-propionyl trityl auxiliary **2** (100 mg, 0.232 mmol, 1 equiv) and magnesium chloride (22.1 mg, 0.232 mmol, 1.00 equiv) in tetrahydrofuran (0.7 mL) was added sodium bis(trimethylsilyl)amide (NaHMDS, 1.0 M in tetrahydrofuran, 0.460 mL, 0.463 mmol, 2.00 equiv) dropwise at −78 °C. A solution of 2-nitrobenzyl bromide (150 mg, 0.695 mmol, 3.00 equiv) in tetrahydrofuran (0.7 mL) was added immediately dropwise and the reaction mixture was stirred for 1 h at –78 °C. The product mixture was quenched with saturated aqueous ammonium chloride solution (1.5 mL). The quenched product mixture was transferred to a separatory funnel and the layers were separated. The aqueous layer was extracted with diethyl ether (3 × 5 mL). The combined organic extracts were dried over sodium sulphate and filtered. The filtrate was concentrated to afford a yellow residue that was purified by flash column chromatography (50 µm silica, eluting with 10% ethyl acetate-hexane initially, grading to 30% ethyl acetate-hexane, linear gradient) to afford the *alkylation product* **27** as an off-white solid (106 mg, 81%).

*Note*: The absolute stereochemistry was unambiguosly confirmed by X-ray crystallography. Crystals were obtained from slow evaporation of hexane–ethyl acetate (8:2)

$R_f$ = 0.34 (hexane/EtOAc = 8:2)

$[\alpha]_D^{20}$: –22.0 (*c* 0.25, $CHCl_3$)

$^1$H NMR (500 MHz, $CDCl_3$) δ 7.90 – 7.82 (m, 1H, $H_7$), 7.41 – 7.37 (m, 5H, $H_{8\text{-}13}$), 7.34 – 7.29 (m, 7H, $H_{8\text{-}13}$), 7.29 – 7.22 (m, 6H, $H_{8\text{-}13}$), 4.15 – 4.10 (m, 2H, $H_{2,4}$), 4.09 – 4.01 (m, 1H, $H_1$), 3.72 (app dd, *J* = 8.9, 3.3 Hz, 1H, $H_1$), 3.24 (dd, *J* = 13.7, 8.2 Hz, 1H, $H_5$), 3.13 (dd, *J* = 13.7, 6.2 Hz, 1H, $H_5$), 2.60 – 2.47 (m, 1H, $H_3$), 2.36 (dd, *J* = 12.5, 8.9 Hz, 1H, $H_3$), 1.18 (d, *J* = 6.8 Hz, 3H, $H_6$).

$^{13}$C NMR (126 MHz, $CDCl_3$) δ 175.7 (C), 152.7 (C), 149.8 (C), 144.2 (3 × C), 134.2 (C), 132.7 (CH), 132.3 (CH), 129.5 (6 × CH), 128.1 (6 × CH), 127.4 (CH), 127.1 (3 × CH), 124.8 (CH), 67.2 (C), 66.0 ($CH_2$), 53.1 (CH), 38.7 (CH), 35.9 ($CH_2$), 33.3 ($CH_2$), 17.4 ($CH_3$).

HRMS-CI (m/z): $[M + NH_4]^+$ calcd for $C_{33}H_{30}N_2O_5SNH_4$, 584.2214; found, 584.2233.

*Synthesis of the oxazolidinone thioester* **28**:

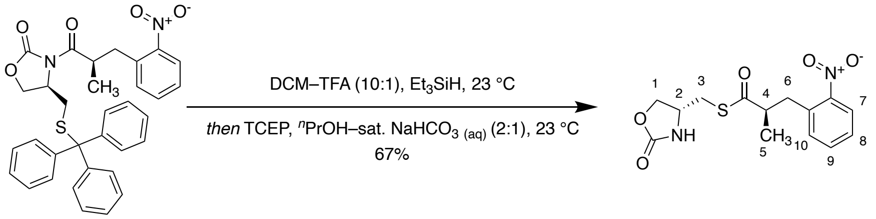


To a solution of alkylation product **27** (197 mg, 0.348 mmol, 1 equiv) in dichloromethane (1.1 mL) were added trifluoroacetic acid (106 µL, 1.39 mmol, 4.00 equiv) and triethylsilane (155 µL, 0.973 mmol, 2.80 equiv) at 23 °C. The reaction mixture was stirred for 30 min at 23 °C. The reaction mixture was concentrated and dried under high vacuum to afford a white solid that was used in the next step without further purification.
The trityl deprotected adduct was dissolved in 1-propanol (1.7 mL) and saturated aqueous solution of sodium bicarbonate (0.90 mL). Tris(2-carboxyethyl)phosphine (10.0 mg, 35.0 µmol, 10 mol%) was added and the reaction mixture was stirred for 4 h at 23 °C. The product mixture was quenched with saturated aqueous sodium chloride solution (3 mL). The diluted product mixture was transferred to a separatory funnel and the aqueous layer was extracted with ethyl acetate (3 × 7 mL). The combined organic extracts were dried over sodium sulphate and filtered. The filtrate was concentrated, and the white residue was purified by flash-column chromatography (eluting with 100% dichloromethane initially, grading to 10% methanol-dichloromethane, linear gradient) to afford the *oxazolidinone thioester* **28** as an orange-yellow oil (75.0 mg, 67%).

$R_f$ = 0.48 (DCM/MeOH = 95:5)

$[\alpha]_D^{20}$: –94.5 (*c* 0.13, $CHCl_3$)

$^1$H NMR (500 MHz, $CDCl_3$) δ 7.99 (app dd, *J* = 8.2, 1.4 Hz, 1H, $H_7$), 7.55 (app td, *J* = 7.5, 1.4 Hz, 1H, $H_8$), 7.42 (app ddd, *J* = 8.5, 7.5, 1.5 Hz, 1H, $H_9$), 7.29 (app dd, *J* = 7.6, 1.5 Hz, 1H, $H_{10}$), 5.25 (s, 1H, –N*H*), 4.43 – 4.35 (m, 1H, $H_1$), 4.02 – 3.88 (m, 2H, $H_{1,2}$), 3.27 (dd, *J* = 13.0, 7.9 Hz, 1H, $H_6$), 3.19 – 3.11 (m, 1H, $H_4$), 3.11 – 3.01 (m, 2H, $H_{3,6}$), 2.99 – 2.93 (m, 1H, $H_3$), 1.28 (d, *J* = 6.7 Hz, 3H, $H_5$).

$^{13}$C NMR (126 MHz, $CDCl_3$) δ 202.1 (C), 158.8 (C), 149.3 (C), 134.0 (C), 133.3 (CH), 133.1 (CH), 128.2 (CH), 125.4 (CH), 69.1 ($CH_2$), 51.8 (CH), 49.5 (CH), 37.2 ($CH_2$), 33.0 ($CH_2$), 18.3 ($CH_3$).

HRMS-CI (m/z): $[M + H]^+$ calcd for $C_{14}H_{17}N_2O_5S$, 325.0853; found, 325.0861.

*Synthesis of the dihydroquinolinone **29***:

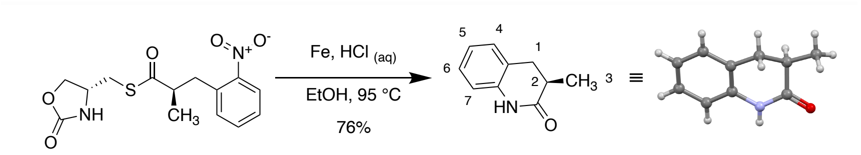


**28** **29**

To a solution of the oxazolidinone thioester **28** (88.0 mg, 0.271 mmol, 1 equiv) in ethanol (0.80 mL) were added iron powder (60.6 mg, 1.09 mmol, 4.00 equiv) and 0.1 M aqueous hydrogen chloride solution (136 µL). The reaction mixture was stirred at 95 °C for 1 h. The product mixture was cooled to 23 °C and quenched with saturated aqueous sodium bicarbonate solution (1 mL). The quenched product mixture was transferred to a separatory funnel and the layers were separted. The aqueous layer was extracted with ethyl acetate (3 × 3 mL). The combined organic extracts were dried over sodium sulphate and filtered. The filtrate was concentrated to give a brown oil. The residue was purified by flash-column chromatography (eluting with 20% ethyl acetate-hexane initially, grading to 30% ethyl acetate-hexane, linear gradient) to afford the *dihydroquinolinone* **29** as a white crystalline solid (33.3 mg, 76%).

The absolute stereochemistry was unambiguously confirmed be X-ray crystallography. Crystals were obtained from slow evaporation of hexane–ethyl acetate (8:2).

$R_f$ = 0.38 (hexane/EtOAc = 7:3)

$[\alpha]_D^{20}$: +33.2 (*c* 0.25, $CHCl_3$)

$^1$H NMR (500 MHz, $CDCl_3$) δ 8.48 (s, 1H, –N*H*), 7.17 (app td, *J* = 7.6, 6.1 Hz, 2H, $H_4$, $H_6$), 6.98 (td, *J* = 7.5, 1.2 Hz, 1H, $H_5$), 6.79 (app dd, *J* = 7.9, 1.1 Hz, 1H, $H_7$), 3.00 (dd, *J* = 15.2, 5.6 Hz, 1H, $H_1$), 2.74 (dd, *J* = 15.2, 10.9 Hz, 1H, $H_1$), 2.71 – 2.63 (m, 1H, $H_2$), 1.29 (d, *J* = 6.7 Hz, 3H, $H_3$).

$^{13}$C NMR (126 MHz, $CDCl_3$) δ 174.7 (C), 137.3 (C), 128.2 (CH), 127.6 (CH), 123.7 (C), 123.1 (CH), 115.2 (CH), 35.1 (CH), 33.6 ($CH_2$), 15.5 ($CH_3$).

HRMS-CI (m/z): $[M + H]^+$ calcd for $C_{10}H_{12}NO$, 162.0913; found, 162.0917.

*Synthesis of the N-pentanoyl oxazolidinone **30***:

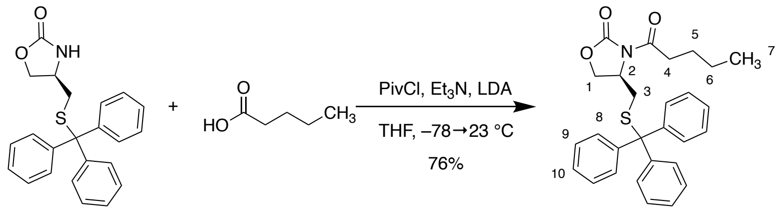


**1** **30**

To a solution of *S*-trityl auxiliary **1** (4.00 g, 10.7 mmol, 1 equiv) in tetrahydrofuran (23 mL) was added lithium diisopropylamide (2.0 M solution in tetrahydrofuran/heptane/ethylbenzene, 6.00 mL, 12.0 mmol, 1.10 equiv) dropwise at –78 °C, and the reaction mixture was stirred for 10 min at –78 °C. The dry ice-acetone bath was swapped with an ice bath and the reaction mixture was stirred at 0 °C for 10 min. The reaction mixture was recooled to –78 °C and used in the acylation step below.

In a separate round-bottomed flask, a solution of pentanoic acid (1.40 mL, 13.0 mmol, 1.20 equiv) in tetrahydrofuran (19.0 mL) were added triethylamine (2.20 mL, 15.8 mmol, 1.50 equiv) and pivaloyl chloride (1.80 mL, 14.5 mmol, 1.36 equiv) at –78 °C, and the reaction mixture was stirred for 10 min at –78 °C. The dry ice-acetone bath was swapped with an ice bath and the reaction mixture was stirred at 0 °C for 10 min. The reaction mixture was recooled to –78 °C and the above solution was cannulated at –78 °C. The dry ice-acetone bath was removed, and the reaction mixture was stirred at 23 °C for 2 h. The product mixture was quenched with saturated aqueous ammonium chloride solution (40 mL) and partially concentrated. The partially concentrated product mixture was transferred to a separatory funnel and the layers were separated. The aqueous layer was extracted with ethyl acetate (3 × 40 mL). The combined organic extracts were sequentially washed with saturated aqueous citric acid (50 mL), saturated aqueous sodium bicarbonate (50 mL), and saturated aqueous sodium chloride solution (50 mL). The washed organic extracts were dried over sodium sulphate and filtered. The filtrate was concentrated to give an oily yellow residue what was purified by flash-column chromatography (15 $\mu$m silica, eluting with 5% ethyl acetate-hexane initially, grading to 15% ethyl acetate-hexane, linear gradient) to afford the *N-pentanoyl oxazolidinone* **30** as a white solid (3.75 g, 77%).

*Note 1*: Purification with irregular silica (40-63 μm) did not fully remove pivaloyl chloride derived impurities.

*Note 2*: The product is initially a white sticky gum which later solidified under high vacuum.

*Note 3*: The reaction mixture becomes a thick slurry after cannulation thus vigorous stirring (> 1000 rpm) was necessary for best yields.

$R_f$ = 0.56 (hexane/EtOAc = 8:2)

$[\alpha]_D^{20}$: +26.0 ($c$ 0.41, $CHCl_3$)

$^1H$ NMR (500 MHz, $CDCl_3$) δ 7.46 – 7.42 (m, 6H, $H_8$), 7.30 (t, $J$ = 7.7 Hz, 6H, $H_9$), 7.25 – 7.20 (m, 3H, $H_{10}$), 4.22 – 3.98 (m, 2H, $H_{1,2}$), 3.91 – 3.82 (m, 1H, $H_1$), 2.91 – 2.76 (m, 2H, $H_4$), 2.73 (dd, $J$ = 12.5, 3.3 Hz, 1H, $H_3$), 2.64 – 2.54 (m, 1H, $H_3$), 1.83 – 1.43 (m, 2H, $H_5$), 1.36 (app h, $J$ = 7.4 Hz, 2H, $H_6$), 0.93 (t, $J$ = 7.4 Hz, 3H, $H_7$).

$^{13}C$ NMR (126 MHz, $CDCl_3$) δ 173.2 (C), 153.4 (C), 144.4 (3 × C), 129.6 (6 × CH), 128.2 (6 × CH), 127.1 (3 × CH), 67.2 (C), 66.2 ($CH_2$), 53.0 (CH), 35.3 ($CH_2$), 33.6 ($CH_2$), 26.4 ($CH_2$), 22.3 ($CH_2$), 14.0 ($CH_3$).

HRMS-CI (m/z): $[M + Na]^+$ calcd for $C_{28}H_{29}NO_3SNa$, 482.1760; found, 482.1764.

*Synthesis of the methylation product **S13***:

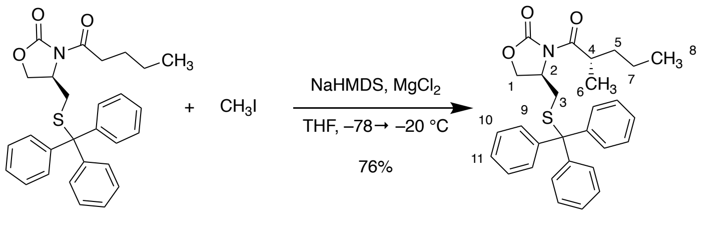


To a solution of *N*-pentanoyl oxazolidinone **30** (1.50 g, 3.26 mmol, 1 equiv) and magnesium chloride (0.311 g, 3.26 mmol, 1.00 equiv) in tetrahydrofuran (9.0 mL) was added sodium bis(trimethylsilyl)amide (NaHMDS, 1.0 M solution in tetrahydrofuran, 6.50 mL, 6.53 mmol, 2.00 equiv) dropwise at −78 °C. The reaction mixture was stirred for 30 min at this temperature. Methyl iodide (0.610 mL, 9.79 mmol, 3.00 equiv) was added dropwise and the reaction mixture was stirred at –78 °C for 1 h and warmed to –20 °C over 2 h. The product mixture was quenched with saturated aqueous ammonium chloride solution (10 mL) and transferred to a separatory funnel. The aqueous layer was extracted with diethyl ether (3 × 20 mL). The combined organic extracts were dried over sodium sulphate and filtered. The filtrate was concentrated to afford the crude product (orange oil) as a mixture of diastereomers (d.r. = 90:10). The diastereomers were separated by flash-column chromatography (15 µm silica, eluting with 3% ethyl acetate-hexane initially, grading to 6% ethyl acetate-hexane, linear gradient) to afford the *methylation product* **S13** as a white sticky gum (1.17 g, 76%; combined yield).

$R_f$ = 0.60 (hexane/EtOAc = 8:2)

$[\alpha]_D^{20}$: +45.2 (*c* 0.18, $CHCl_3$)

*Major diastereomer*:
$^1$H NMR (500 MHz, $CDCl_3$) δ 7.47 – 7.40 (m, 6H, $H_9$), 7.34 – 7.27 (m, 6H, $H_{10}$), 7.26 – 7.17 (m, 3H, $H_{11}$), 4.17 – 4.11 (m, 1H, $H_2$), 4.08 (app t, *J* = 8.5 Hz, 1H, $H_1$), 3.81 (dd, *J* = 8.8, 3.1 Hz, 1H, $H_1$), 3.65 (app h, *J* = 6.7 Hz, 1H, $H_4$), 2.74 – 2.53 (m, 2H, $H_3$), 1.66 (app ddt, *J* = 12.4, 8.9, 5.9 Hz, 1H, $H_5$), 1.44 – 1.21 (m, 3H, $H_5$, $H_7$), 1.17 (d, *J* = 6.9 Hz, 3H, $H_6$), 0.89 (t, *J* = 7.2 Hz, 3H, $H_8$).

$^{13}$C NMR (126 MHz, $CDCl_3$) δ 177.2 (C), 153.0 (C), 144.4 (3 × C), 129.6 (6 × CH), 128.2 (6 × CH), 127.1 (3 × CH), 67.1 (C), 66.0 ($CH_2$), 53.1 (CH), 37.5 (CH), 35.5 ($CH_2$), 33.6 ($CH_2$), 20.4 ($CH_2$), 17.4 ($CH_3$), 14.2 ($CH_3$).

HRMS-CI (m/z): $[M + Na]^+$ calcd for $C_{29}H_{31}NO_3SNa$, 496.1917; found, 496.1914.

*Synthesis of the oxazolidinone thioester **32***:

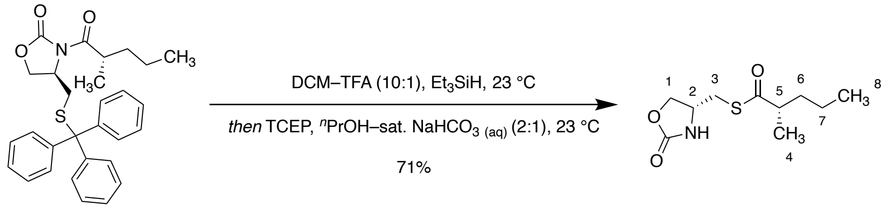


To a solution of methylation product **S13** (140 mg, 0.296 mmol, 1 equiv) in dichloromethane (0.90 mL) were added trifluoroacetic acid (91.0 μL, 1.18 mmol, 4.00 equiv) and triethylsilane (132 μL, 0.828 mmol, 2.80 equiv) at 23 °C. The reaction mixture was stirred for 30 min at 23 °C. The reaction mixture was concentrated and dried under high vacuum to afford a white solid that was used in the next step without further purification.

The trityl deprotected adduct was dissolved in 1-propanol (1.5 mL) and a saturated aqueous solution of sodium bicarbonate (0.74 mL). Tris(2-carboxyethyl)phosphine (8.50 mg, 30.0 μmol, 0.100 equiv) was added and the reaction mixture was stirred for 16 h at 23 °C. The product mixture was diluted with saturated aqueous sodium chloride solution (5 mL) and transferred to a separatory funnel. The diluted product mixture was extracted with ethyl acetate (3 × 10 mL). The combined organic extracts were dried over sodium sulphate and filtered. The filtrate was concentrated, and the residue was purified by flash-column chromatography (eluting with 100% dichloromethane initially, grading to 20% methanol-dichloromethane, linear gradient) to afford the *oxazolidinone thioester* **32** as a pale-yellow oil (48.2 mg, 71%).

$R_f$ = 0.51 (DCM/MeOH = 95:5)

$[\alpha]_D^{20}$: –7.27 (*c* 0.26, $CHCl_3$)

$^1$H NMR (500 MHz, $CDCl_3$) δ 5.22 (s, 1H, –N*H*), 4.54 – 4.43 (m, 1H, $H_1$), 4.12 – 3.99 (m, 2H, $H_{1,2}$), 3.26 – 3.12 (m, 1H, $H_3$), 2.98 (app d, $J$ = 5.4 Hz, 1H, $H_3$), 2.76 – 2.63 (m, 1H, $H_5$), 1.70 (app dddd, $J$ = 13.1, 9.3, 7.3, 5.8 Hz, 1H, $H_6$), 1.47 – 1.37 (m, 1H, $H_6$), 1.37 – 1.27 (m, 2H, $H_7$), 1.18 (d, $J$ = 6.9 Hz, 3H, $H_4$), 0.91 (t, $J$ = 7.3 Hz, 3H, $H_8$).

$^{13}$C NMR (126 MHz, $CDCl_3$) δ 203.2 (C), 159.0 (C), 69.2 ($CH_2$), 52.0 (CH), 48.8 (CH), 36.3 ($CH_2$), 32.9 ($CH_2$), 20.5 ($CH_2$), 17.6 ($CH_3$), 14.1 ($CH_3$).

HRMS-CI (m/z): $[M + H]^+$ calcd for $C_{10}H_{18}NO_3S$, 232.1002; found, 232.1005.

*Synthesis of the SNAC thioester **33***:

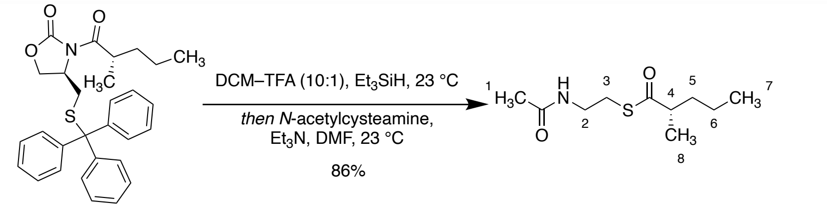


To a solution of methylation product **S13** (100 mg, 0.211 mmol, 1 equiv) in dichloromethane (0.64 mL) were added trifluoroacetic acid (65.0 µL, 0.845 mmol, 4.00 equiv) and triethylsilane (94.0 µL, 0.591 mmol, 2.80 equiv) at 23 °C. The reaction mixture was stirred for 30 min at 23 °C. The reaction mixture was concentrated and dried under high vacuum to afford a white solid that was used in the next step without further purification.
The trityl deprotected adduct was dissolved in *N*,*N*-dimethylformamide (1.1 mL) and triethyl amine (0.300 mL, 2.11 mmol, 10.0 equiv). The reaction mixture was stirred for 3 h at 23 °C then *N*-acetylcysteamine (0.220 mL, 2.11 mmol, 10.0 equiv) was added and stirred for 4 h at 23 °C. The product mixture was diluted with ethyl acetate (7 mL) and transferred to a separatory funnel. The diluted product mixture was sequentially washed with water (5 × 5 mL) and saturated aqueous sodium chloride solution (5 mL). The washed organic extracts were dried over sodium sulphate and filtered. The filtrate was concentrated, and the residue was purified by flash-column chromatography (triethylamine neutralized silica, eluting with 100% dichloromethane initially, grading to 10% methanol-dichloromethane, linear gradient) to afford the *SNAC thioester* **33** as a pale-yellow oil (39.4 mg, 86%).

$R_f$ = 0.51 (DCM/MeOH = 95:5)

$[\alpha]_D^{20}$: +7.43 (*c* 0.18, $CHCl_3$)

$^1$H NMR (500 MHz, $CDCl_3$) δ 5.84 (s, 1H,–N*H*), 3.44 (app q, *J* = 6.1 Hz, 2H, $H_2$), 3.02 (dd, *J* = 6.8, 5.9 Hz, 2H, $H_3$), 2.68 (app h, *J* = 7.0 Hz, 1H, $H_4$), 1.96 (s, 3H, $H_1$), 1.69 (app dddd, *J* = 13.4, 9.5, 7.6, 5.9 Hz, 1H, $H_5$), 1.49 – 1.26 (m, 3H, $H_{5,6}$), 1.17 (d, *J* = 6.9 Hz, 3H, $H_8$), 0.91 (t, *J* = 7.3 Hz, 3H, $H_7$).

$^{13}$C NMR (126 MHz, $CDCl_3$) δ 204.9 (C), 170.4 (C), 48.6 (CH), 40.1 ($CH_2$), 36.4 ($CH_2$), 28.3 ($CH_2$), 23.3 ($CH_3$), 20.5 ($CH_2$), 17.8 ($CH_3$), 14.1 ($CH_3$).

HRMS-CI (m/z): $[M + Na]^+$ calcd for $C_{10}H_{19}NO_2SNa$, 240.1029; found, 240.1029.

*Synthesis of the free thiol **31***:

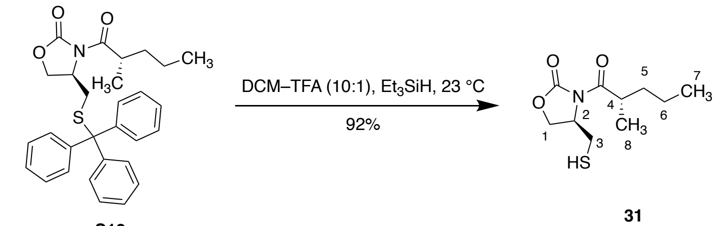


To a solution of methylation product **S15** (100 mg, 0.196 mmol, 1 equiv) in dichloromethane (0.60 mL) were added trifluoroacetic acid (60.0 µL, 0.785 mmol, 4.00 equiv) and triethylsilane (87.0 µL, 0.550 mmol, 2.80 equiv) at 23 °C. The reaction mixture was stirred for 30 min at 23 °C. The reaction mixture was concentrated and dried under high vacuum to afford a white solid. The residue was purified by flash-column chromatography (triethylamine neutralized silica, eluting with 100% dichloromethane initially, grading to 10% methanol-dichloromethane, linear gradient) to afford the *free thiol* **31** as a colourless oil (41.6 mg, 92%).

$R_f$ = 0.50 (hexane/EtOAc = 8:2)

$[\alpha]_D^{20}$: +84.0 (*c* 0.18, $CHCl_3$)

$^1$H NMR (500 MHz, $CDCl_3$) δ 4.62 – 4.48 (m, 1H, $H_2$), 4.43 (app t, *J* = 8.7 Hz, 1H, $H_1$), 4.30 (dd, *J* = 9.2, 3.3 Hz, 1H, $H_1$), 3.73 (app h, *J* = 6.8 Hz, 1H, $H_4$), 2.96 – 2.73 (m, 2H, $H_3$), 1.70 (app ddt, *J* = 12.6, 8.9, 6.1 Hz, 1H, $H_5$), 1.42 – 1.24 (m, 3H, $H_5$, $H_6$), 1.19 (d, *J* = 6.9 Hz, 3H, $H_8$), 0.90 (t, *J* = 7.2 Hz, 3H, $H_7$).

$^{13}$C NMR (126 MHz, $CDCl_3$) δ 177.6 (C), 153.1 (C), 65.9 ($CH_2$), 55.5 (CH), 37.7 (CH), 35.5 ($CH_2$), 26.5 ($CH_2$), 20.5 ($CH_2$), 17.6 ($CH_3$), 14.2 ($CH_3$).

HRMS-CI (m/z): $[M + H]^+$ calcd for $C_{10}H_{18}NO_3S$, 232.1002; found, 232.1008.

*Synthesis of the ethyl thioester **S8***:

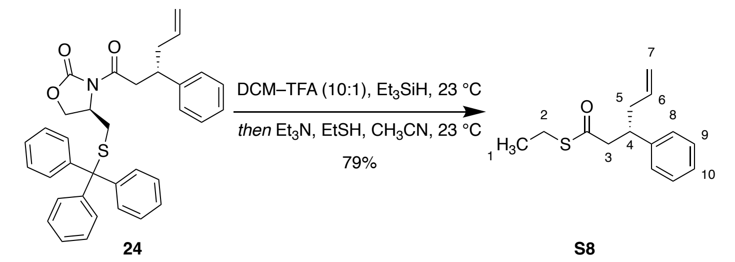


To a solution of terminal alkene **24** (780 mg, 1.42 mmol, 1 equiv) in dichloromethane (4.3 mL) were added trifluoroacetic acid (0.440 mL, 5.70 mmol, 4.00 equiv) and triethylsilane (0.630 mL, 0.591 mmol, 2.80 equiv) at 23 °C. The reaction mixture was stirred for 30 min at 23 °C. The reaction mixture was concentrated and dried under high vacuum to afford a white solid that was used in the next step without further purification.

The trityl deprotected adduct was dissolved in acetonitrile (7.1 mL) and triethyl amine (2.00 mL, 14.2 mmol, 10.0 equiv). The reaction mixture was stirred for 5 h at 23 °C then ethanethiol (1.10 mL, 14.2 mmol, 10.0 equiv) was added. The reaction mixture was stirred for 12 h at 23 °C. The product mixture was diluted with ethyl acetate (15 mL) and transferred to a separatory funnel. The diluted product mixture was sequentially washed with water (3 × 10 mL) and saturated aqueous sodium chloride solution (10 mL). The washed organic extract was dried over sodium sulphate and filtered. The filtrate was concentrated, and the residue was purified by flash-column chromatography (eluting with 100% cyclohexane initially, grading to 6% ethyl acetate-cyclohexane, linear gradient) to afford the *ethyl thioester* **S8** as a cloudy liquid (263 mg, 79%). The spectroscopic data are in agreement with the literature.[6]

$R_f$ = 0.48 (cyclohexane/EtOAc = 95:5)

$^1$H NMR (500 MHz, $CDCl_3$) δ 7.34 – 7.26 (m, 2H, $H_{8\text{-}10}$), 7.23 – 7.14 (m, 3H, $H_{8\text{-}10}$), 5.64 (app ddt, $J$ = 17.2, 10.2, 7.0 Hz, 1H, $H_6$), 5.09 – 4.84 (m, 2H, $H_7$), 3.28 (app p, $J$ = 7.3 Hz, 1H, $H_4$), 2.90 (dd, $J$ = 15.0, 6.8 Hz, 1H, $H_3$), 2.80 (qd, $J$ = 7.7, 2.8 Hz, 3H, $H_2$, $H_3$), 2.40 (app tt, $J$ = 7.2, 1.3 Hz, 2H, $H_5$), 1.16 (t, $J$ = 7.4 Hz, 3H, $H_1$).

$^{13}$C NMR (126 MHz, $CDCl_3$) δ 198.4 (C), 143.3 (C), 135.9 (CH), 128.5 (2 × CH), 127.7 (2 × CH), 126.7 (CH), 117.1 ($CH_2$), 50.0 ($CH_2$), 42.4 (CH), 40.5 ($CH_2$), 23.4 ($CH_2$), 14.8 ($CH_3$).

*Synthesis of the triketide lactone **34***:

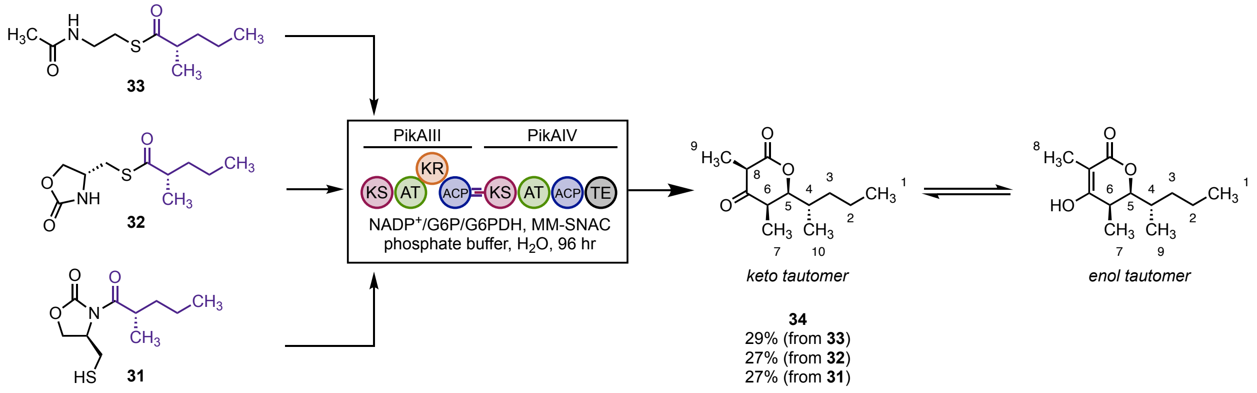


**General procedure**:

To a 50 mL falcon tube were added water, phosphate buffer (200 mM), nicotinamide adenine dinucleotide phosphate (NADP$^+$, 0.5 mM), glucose-6-phosphate (G6P, 10 mM), and glucose-6-phosphate dehydrogenase (G6PDH, 2 mU/μL). The resulting solution was incubated for 10 min at 23 °C. Then, methylmalonyl *N*-acetylcysteamine (MM-SNAC, 20 mM), PikAIII (3 μM), Pik AIV (3 μM), and monoketide substrate (1 mM) in dimethylsulfoxide were added and the solutions were thoroughly mixed. Following incubation for 96 h at 23 °C, (based on QTOF-LC-MS data of the crude reaction mixture), the reaction was separated into two 50 mL falcon tubes, diluted with acetone to the 50 mL marker, placed in a –20 °C freezer for 1 h, and centrifuged (4 °C, 3260 × g) for 10 min. The resulting supernatants were collected and concentrated *in vacuo* to remove all acetone and the aqueous mixture was acidified to pH = 4 using 1.0 M aqueous hydrogen chloride solution. The product mixture was extracted with dichloromethane (3 × 40 mL) and ethyl acetate (2 × 40 mL). The combined organic extracts were dried over sodium sulphate and filtered. The filtrate was concentrated, and the residue was dissolved in 1.2 mL methanol and purified using semi-preparative HPLC (6 injections) using a Phenomenex Synergi 5μm Hydro-RP column (80A, 250 ×10.00 mm, serial number 495511-1).

**Semi-prep purification condition**:

Mobile phase (A = milli-Q water + 0.1% formic acid, B = acetonitrile); 20% B for 2.0 min, 20% to 75% B over 38.0 min, 75% B for 5.0 min, 20% B for 5.0 min; flow rate = 3.0 mL/min.

SNAC-thioester **33**:

23.9 mL of water, 11.5 mL of phosphate buffer, 0.460 mL of NADP$^+$, 0.919 mL of G6P, 0.919 mL of G6PDH, 1.84 mL of MM-SNAC, 2.76 mL of PikAIII, 2.76 mL of PikAIV, and 10 mg (0.046 mmol) was added in 0.920 mL of dimethylsulfoxide. Semi-prep purification provided 2.70 mg (29%) of triketide lactone 35 as a white solid.

Oxazolidinone thioester **32** and free thiol **31**:
22.50 mL of water, 10.87 mL of phosphate buffer, 0.432 mL of $NADP^+$, 0.864 mL of G6P, 0.864 mL of G6PDH, 1.73 mL of MM-SNAC, 2.59 mL of PikAIII, 2.59 mL of PikAIV, and 10 mg (0.043 mmol) of substrate in 0.864 mL of dimethylsulfoxide. Semi-prep purification provided 2.5 mg (27%) of triketide lactone **35** as a white solid for both substrates.

*Keto tautomer*:

$R_f$ = 0.35 (hexane/EtOAc = 2:1)

$^1H$ NMR (600 MHz, $CDCl_3$): δ 4.34 (dd, $J$ = 10.0, 2.5 Hz, 1H, $H_5$), 3.62 (q, $J$ = 6.6 Hz, 1H, $H_8$), 2.69 (qd, $J$ = 7.5, 2.6 Hz, 1H, $H_6$), 1.91 – 1.81 (m, 2H, $H_3$, $H_4$), 1.53 – 1.42 (m, 1H, $H_2$), 1.36 (d, $J$ = 6.6 Hz, 3H, $H_9$), 1.33 – 1.23 (m, 1H, $H_2$), 1.21 – 1.14 (m, 1H, $H_3$), 1.12 (d, $J$ = 7.6 Hz, 3H, $H_7$), 0.94 (t, $J$ = 7.3 Hz, 3H, $H_1$), 0.91 (d, $J$ = 6.8 Hz, 3H, $H_{10}$).

$^{13}C$ NMR (151 MHz, $CDCl_3$): δ 206.1 (C), 170.2 (C), 81.1 (CH), 50.4 (CH), 43.3 (CH), 35.0 ($CH_2$), 33.2 (CH), 19.6 ($CH_2$), 14.4 (2 × $CH_3$), 10.0 ($CH_3$), 8.2 ($CH_3$).

HRMS-CI (m/z): $[M + H]^+$ calcd for $C_{12}H_{21}O_3$, 213.1485; found 213.1472.

*Enol tautomer*:

$[\alpha]_D^{25}$: +58.0 (*c* 0.25, MeOH)

$^1H$ NMR (600 MHz, $CD_3OD$): δ 3.92 (dd, $J$ = 10.3, 3.0 Hz, 1H, $H_5$), 2.46 (qd, $J$ = 7.0, 3.0 Hz, 1H, $H_6$), 1.87 – 1.74 (m, 2H, $H_{3\text{-}4}$), 1.70 (s, 3H, $H_8$), 1.54 – 1.41 (m, 1H, $H_2$), 1.36 – 1.24 (m, 1H, $H_2$), 1.18 – 1.12 (m, 1H, $H_3$), 1.09 (d, $J$ = 7.0 Hz, 3H, $H_7$), 0.94 (t, $J$ = 7.3 Hz, 3H, $H_1$), 0.89 (d, $J$ = 6.8 Hz, 3H, $H_9$).

$^{13}C$ NMR (151 MHz, $CD_3OD$): δ 174.8 (C), 172.5 (C), 97.8 (C), 83.0 (CH), 36.3 (CH), 35.8 ($CH_2$), 34.3 (CH), 20.5 ($CH_2$), 14.7 ($CH_3$), 14.6 ($CH_3$), 10.5 ($CH_3$), 8.6 ($CH_3$).

HRMS-CI (m/z): $[M + H]^+$ calcd for $C_{12}H_{19}O_2$, 195.1380; found 195.1365.

Chemical shifts and multiplicities are similar to several synthetic and natural triketide and tetraketide lactones. [35, 36]

## **Catalog of nuclear magnetic resonance spectra**:

$^1H$ NMR, 500 MHz, $CDCl_3$, **1**

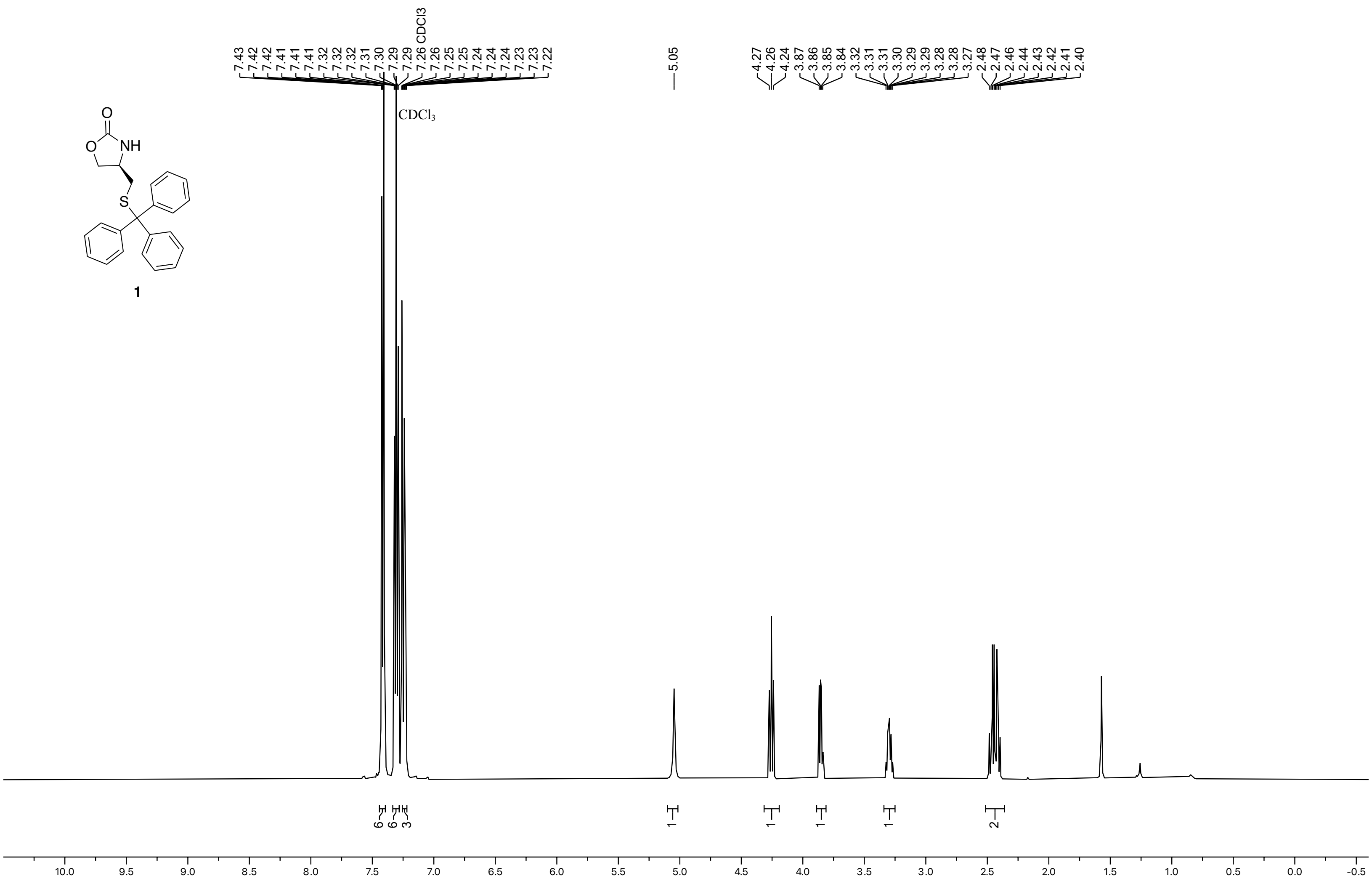

$^{13}C\{^{1}H\}$ NMR, 126 MHz, $CDCl_3$, **1**

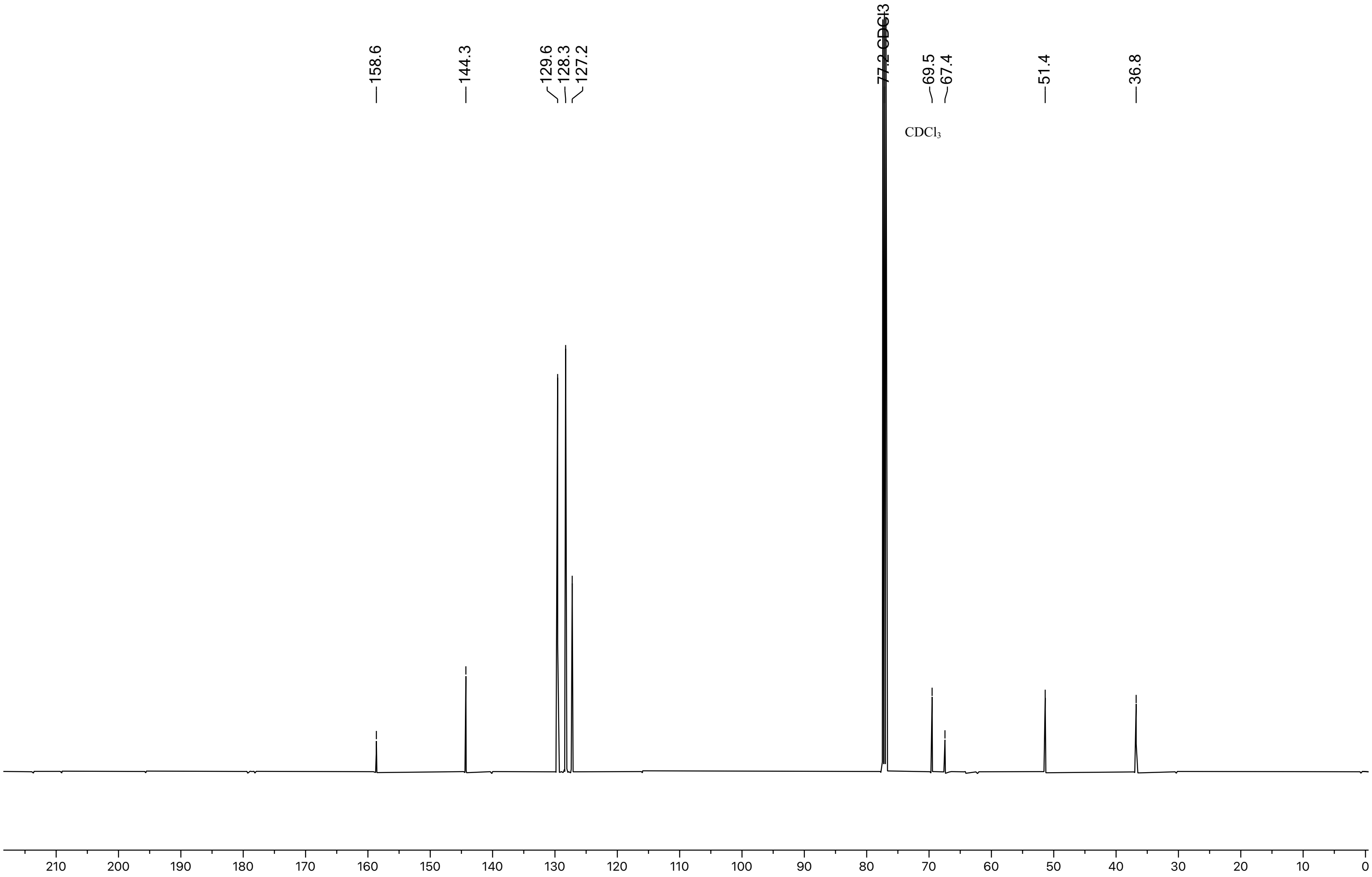

$^1$H NMR, 500 MHz, $CDCl_3$, **2**

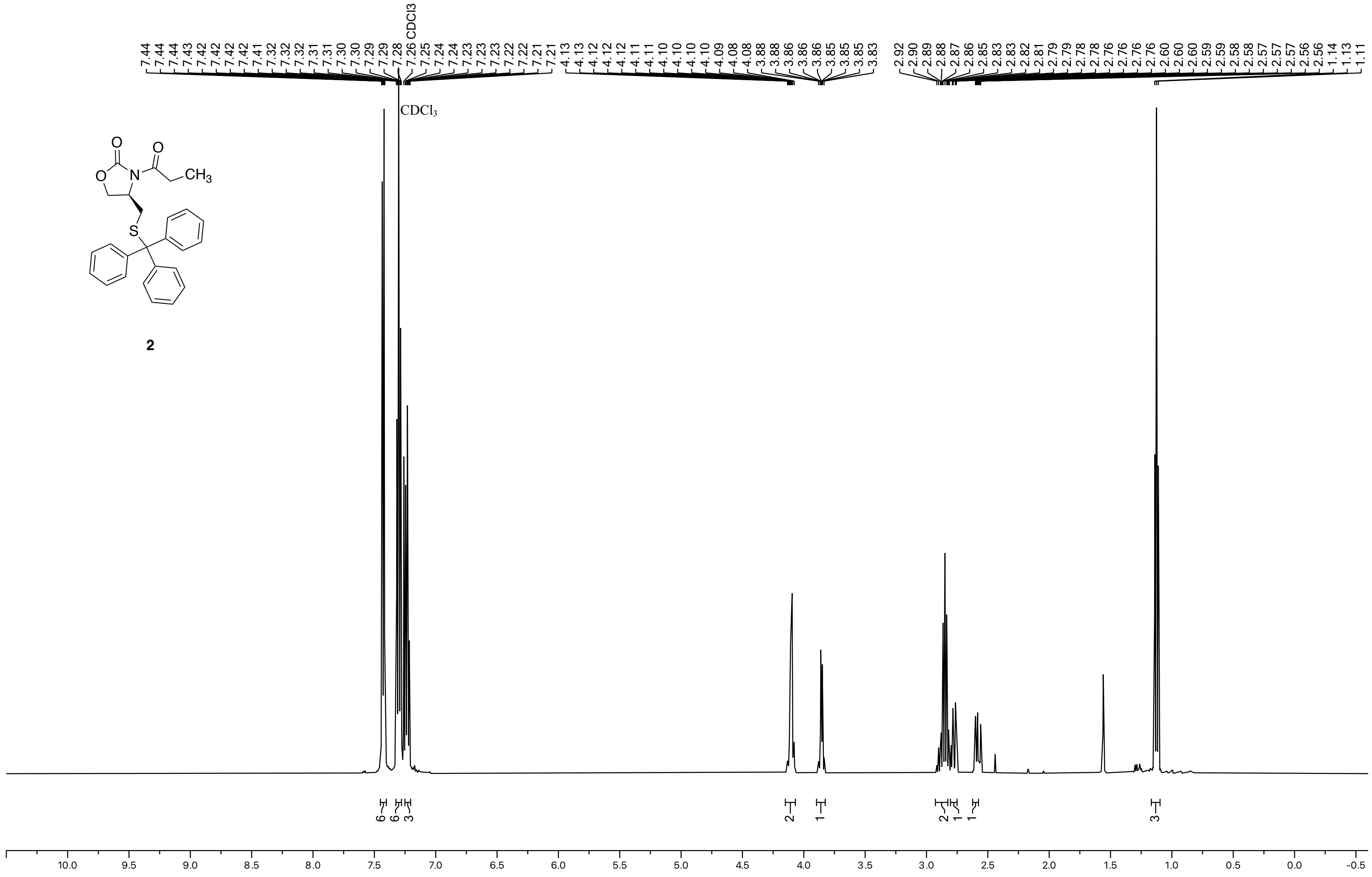

$^{13}C\{^1H\}$ NMR, 126 MHz, $CDCl_3$, **2**

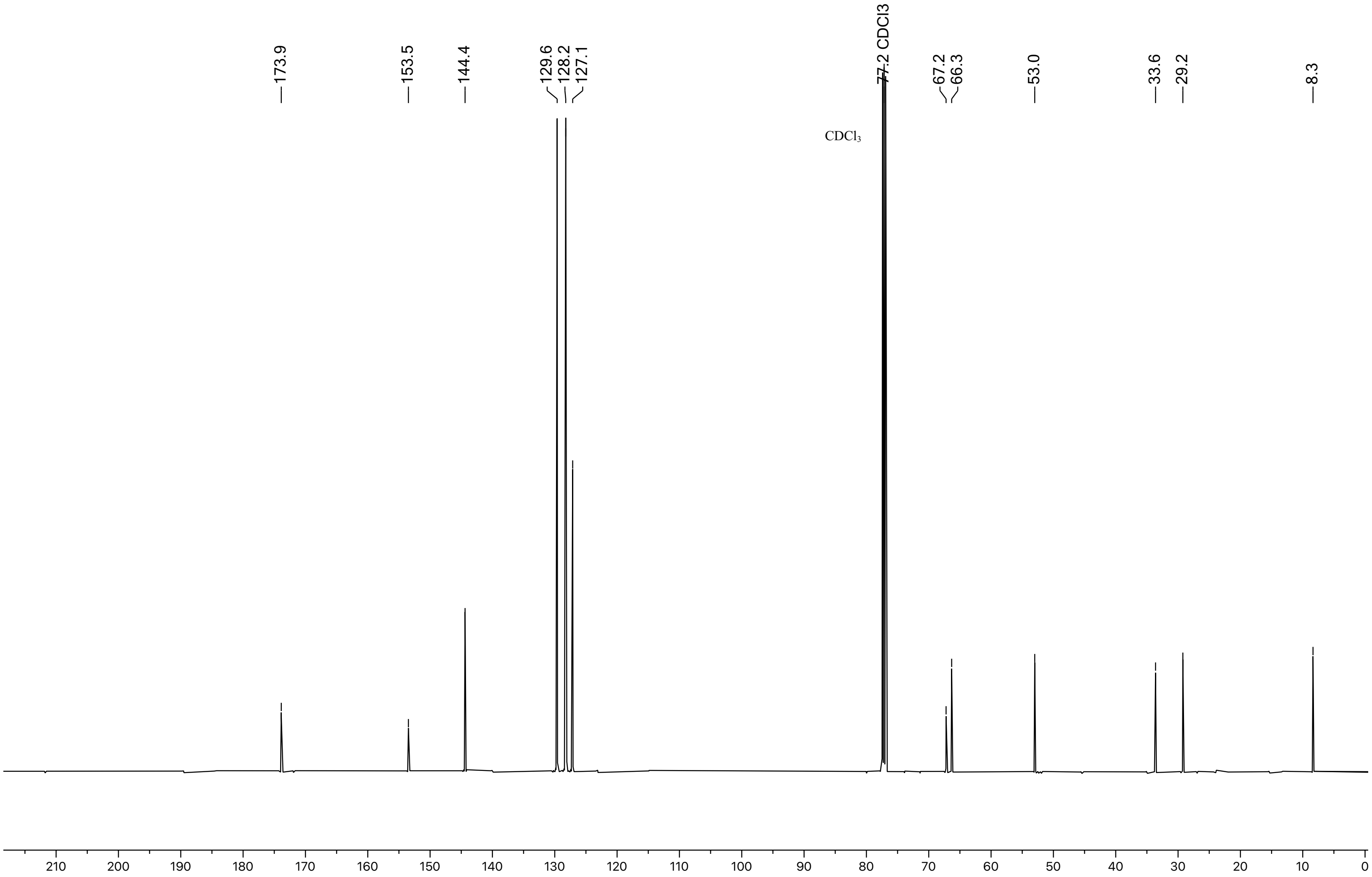

[1]H NMR, 500 MHz, $CDCl_3$, **3**

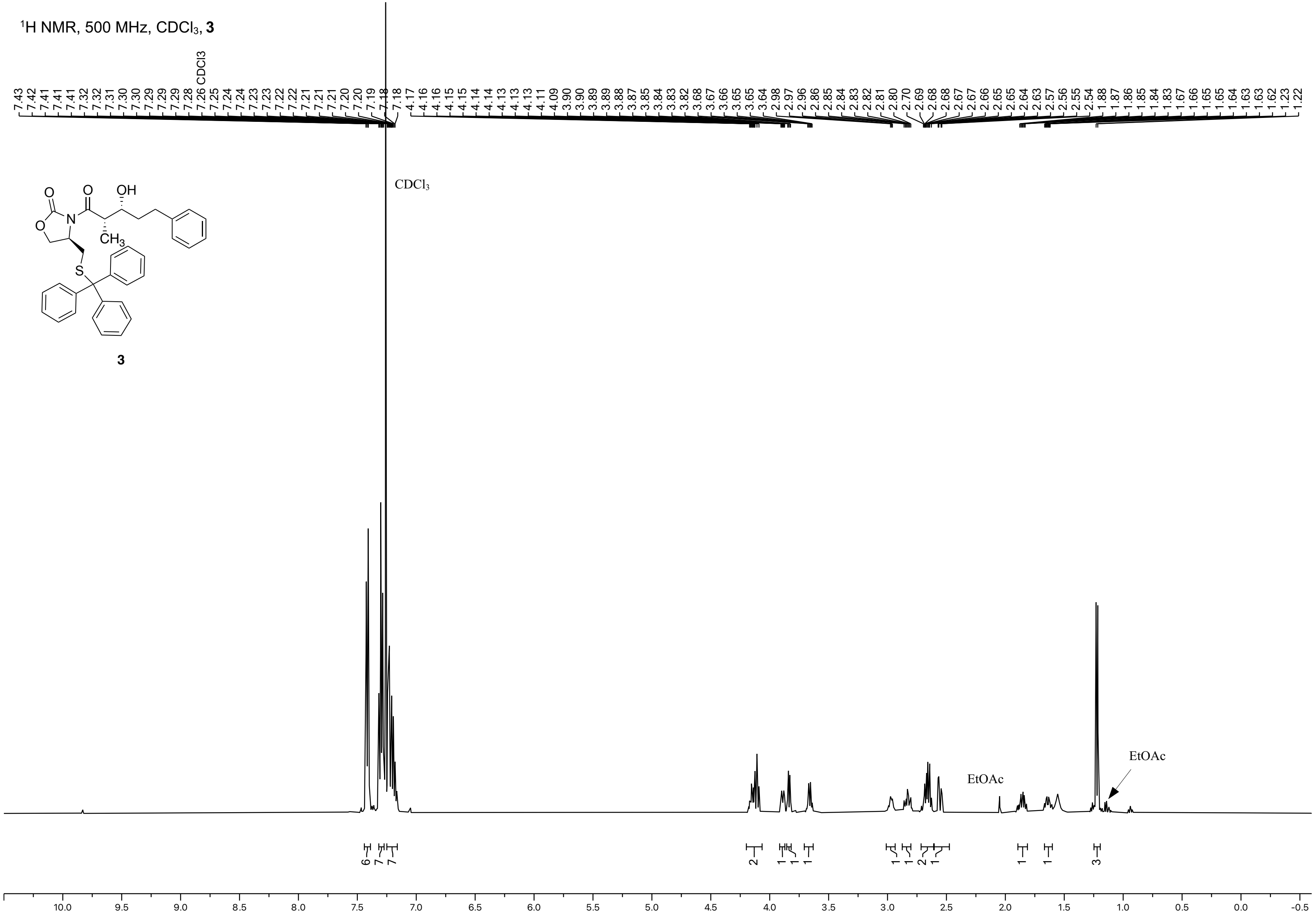

$^{13}C\{^1H\}$ NMR, 126 MHz, $CDCl_3$, **3**

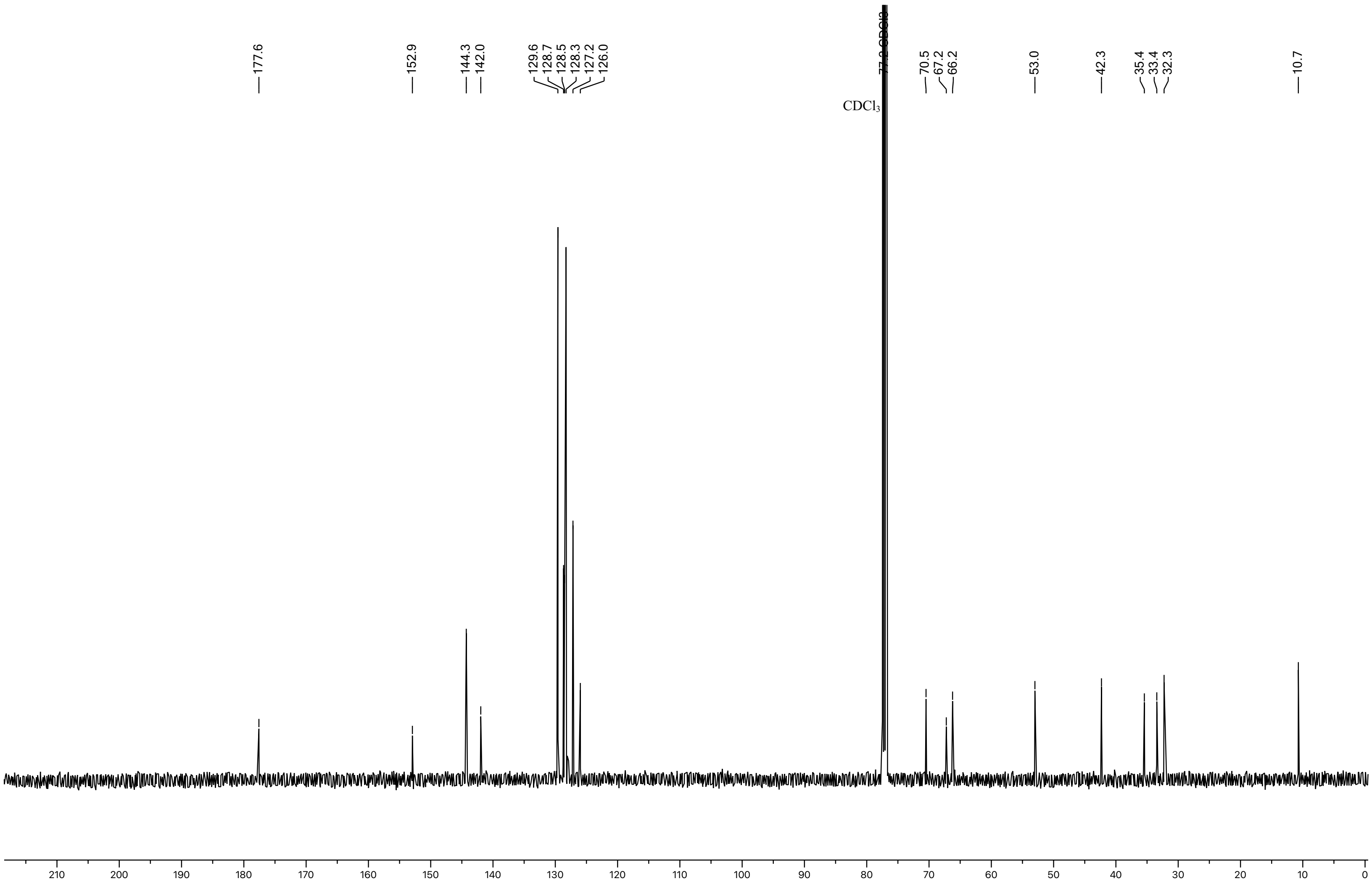

$^1H$ NMR, 500 MHz, $CDCl_3$, **5**

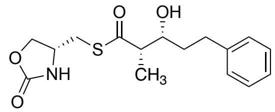


**5**

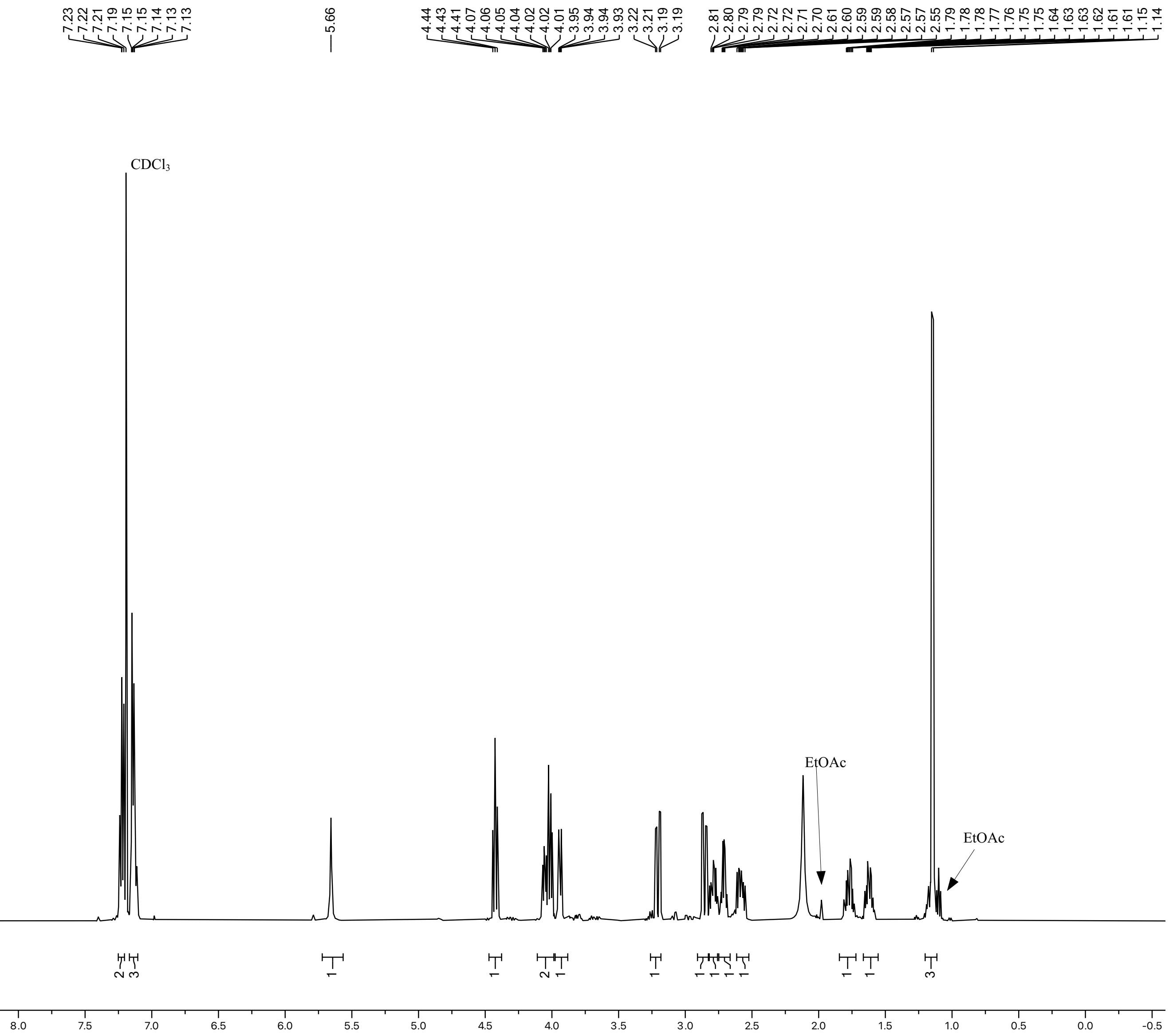

$^{13}C\{^1H\}$ NMR, 126 MHz, $CDCl_3$, **5**

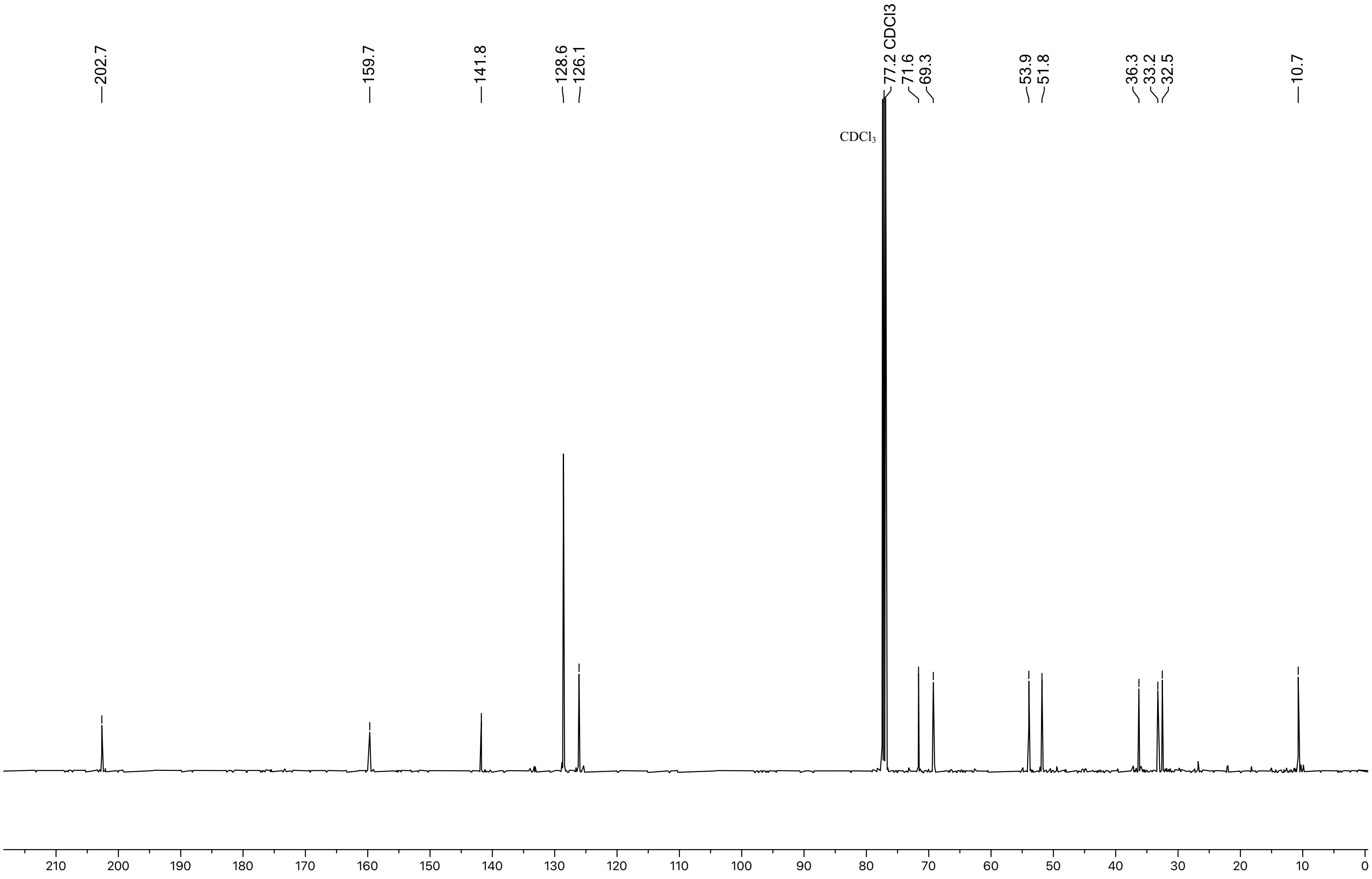

[1]H NMR, 500 MHz, $CDCl_3$, **7**

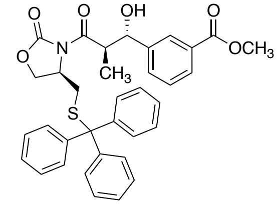


**7**

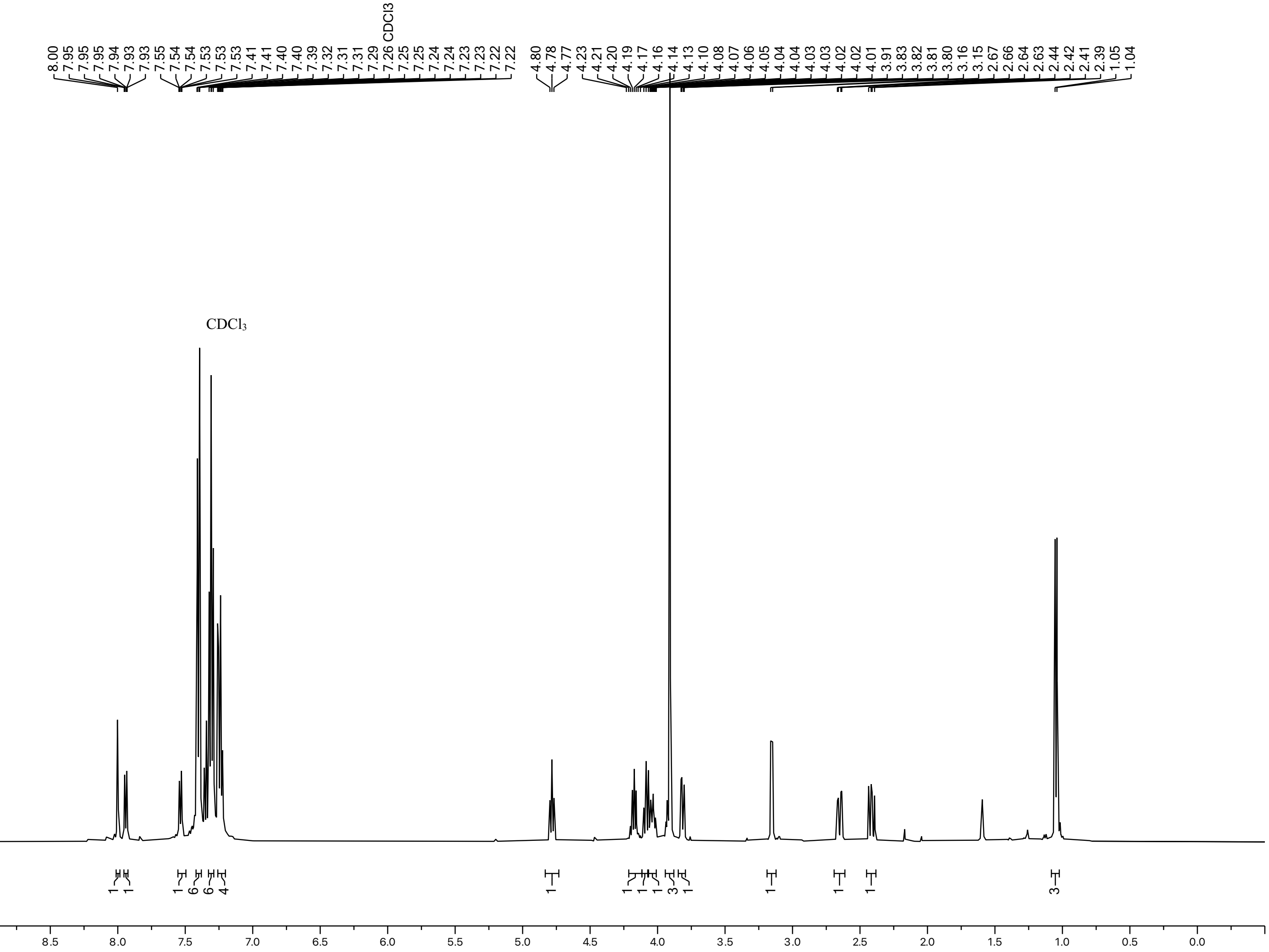

$^{13}C\{^{1}H\}$ NMR, 126 MHz, $CDCl_3$, **7**

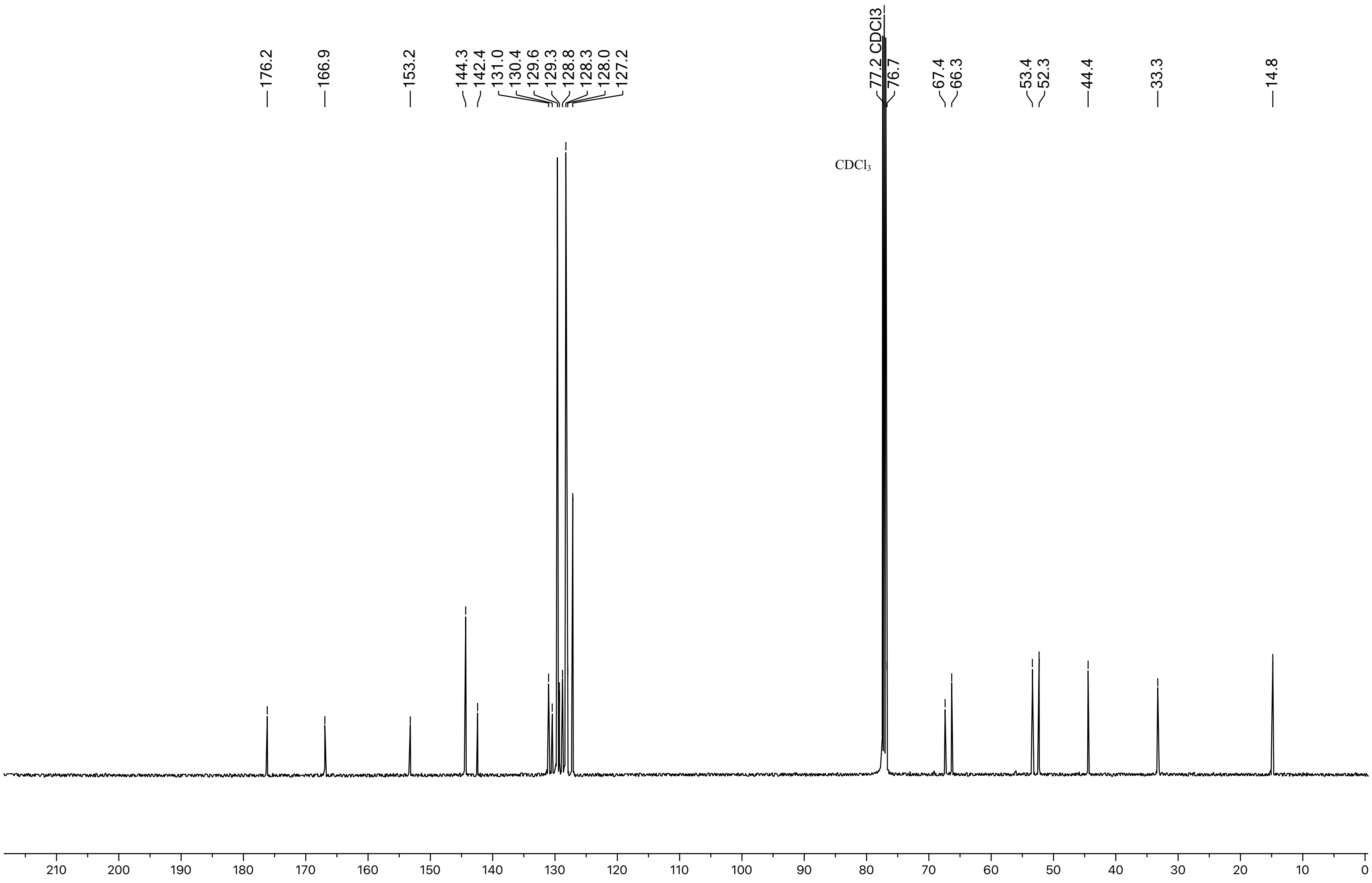

$^1$H NMR, 500 MHz, $CDCl_3$, **S3**

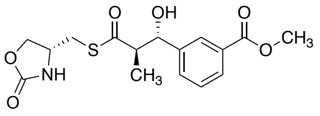

**S3**

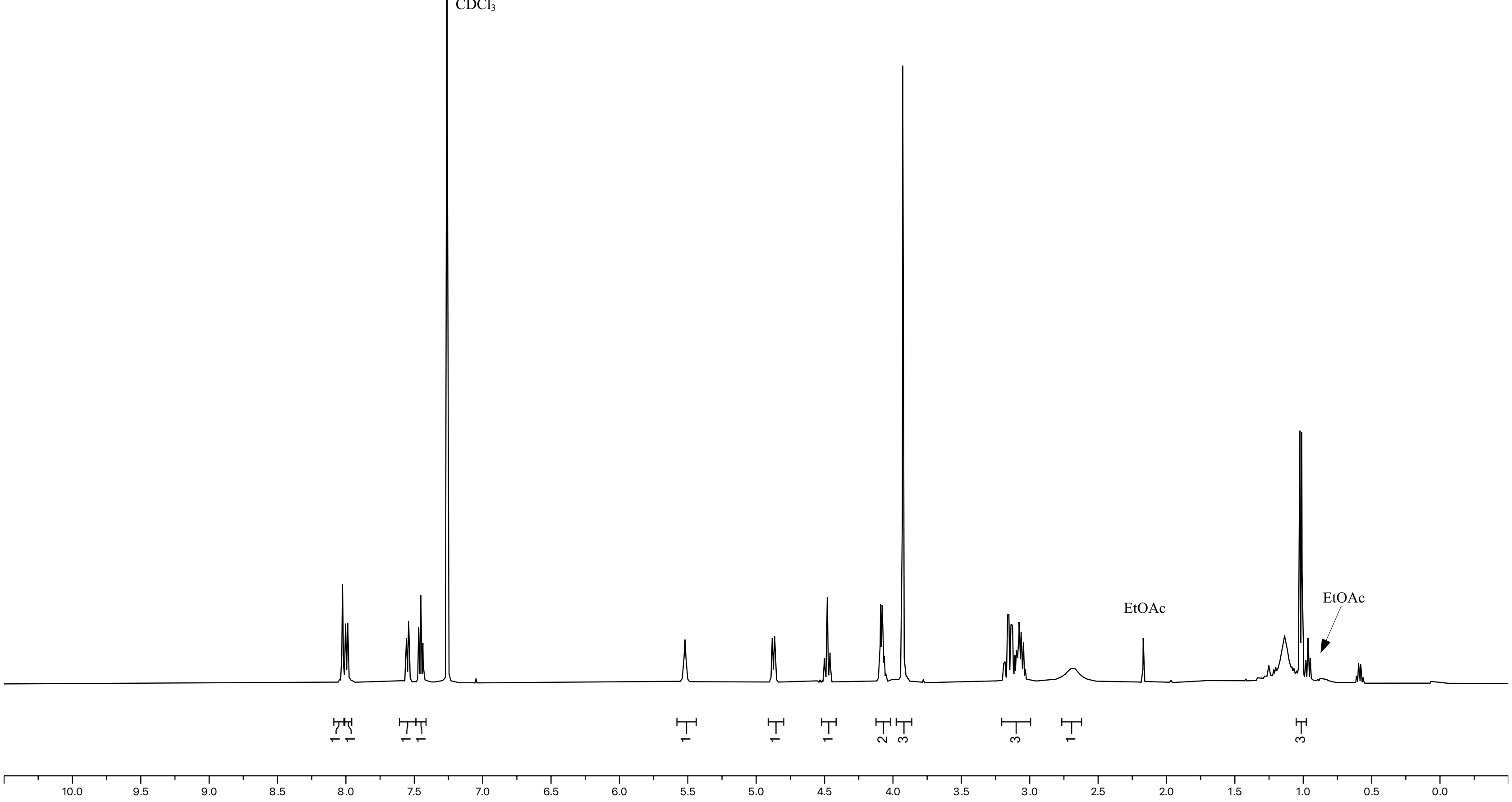

$^{13}C\{^{1}H\}$ NMR, 126 MHz, $CDCl_3$, **S3**

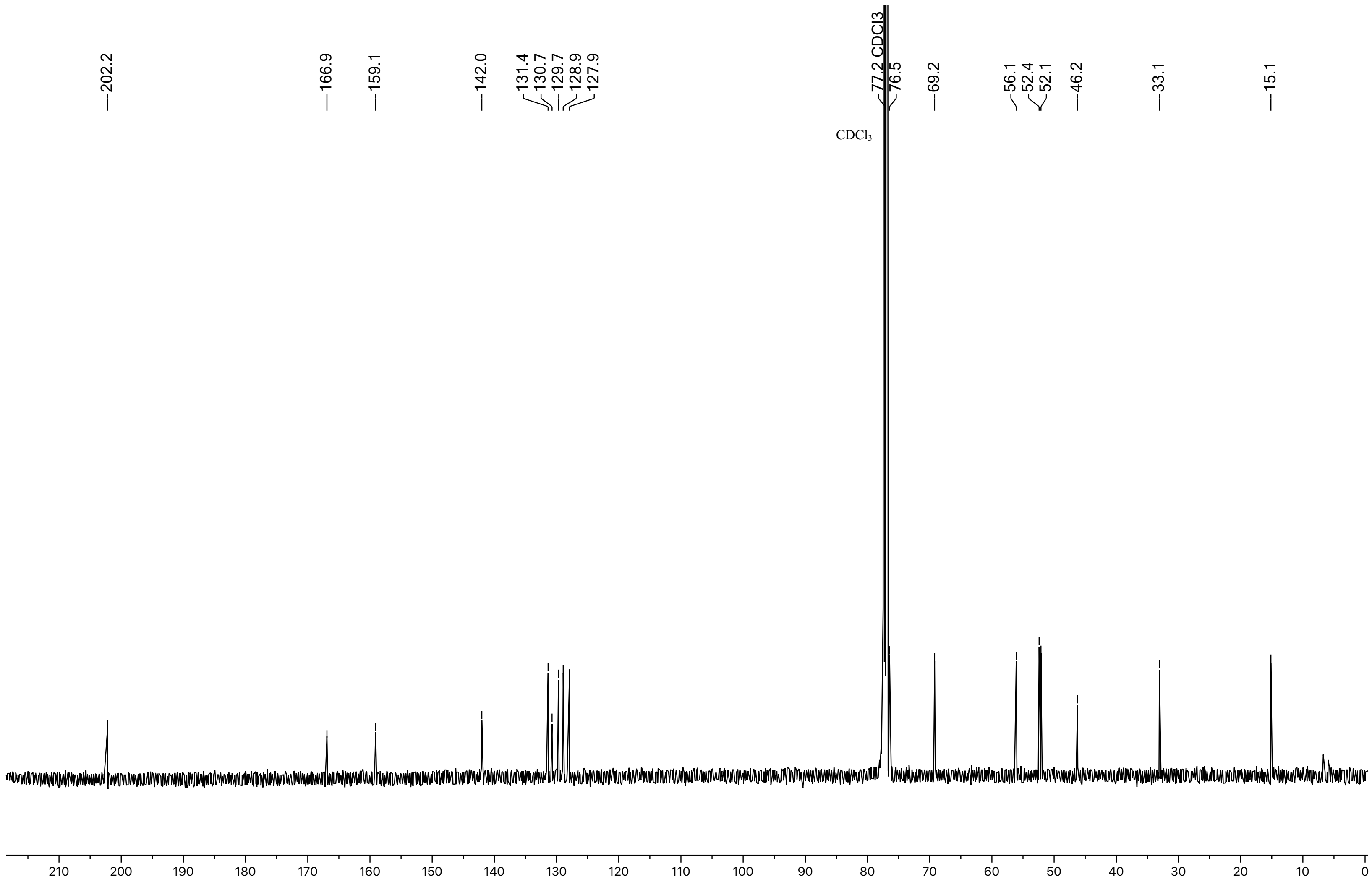

$^1$H NMR, 500 MHz, $CDCl_3$, **8**

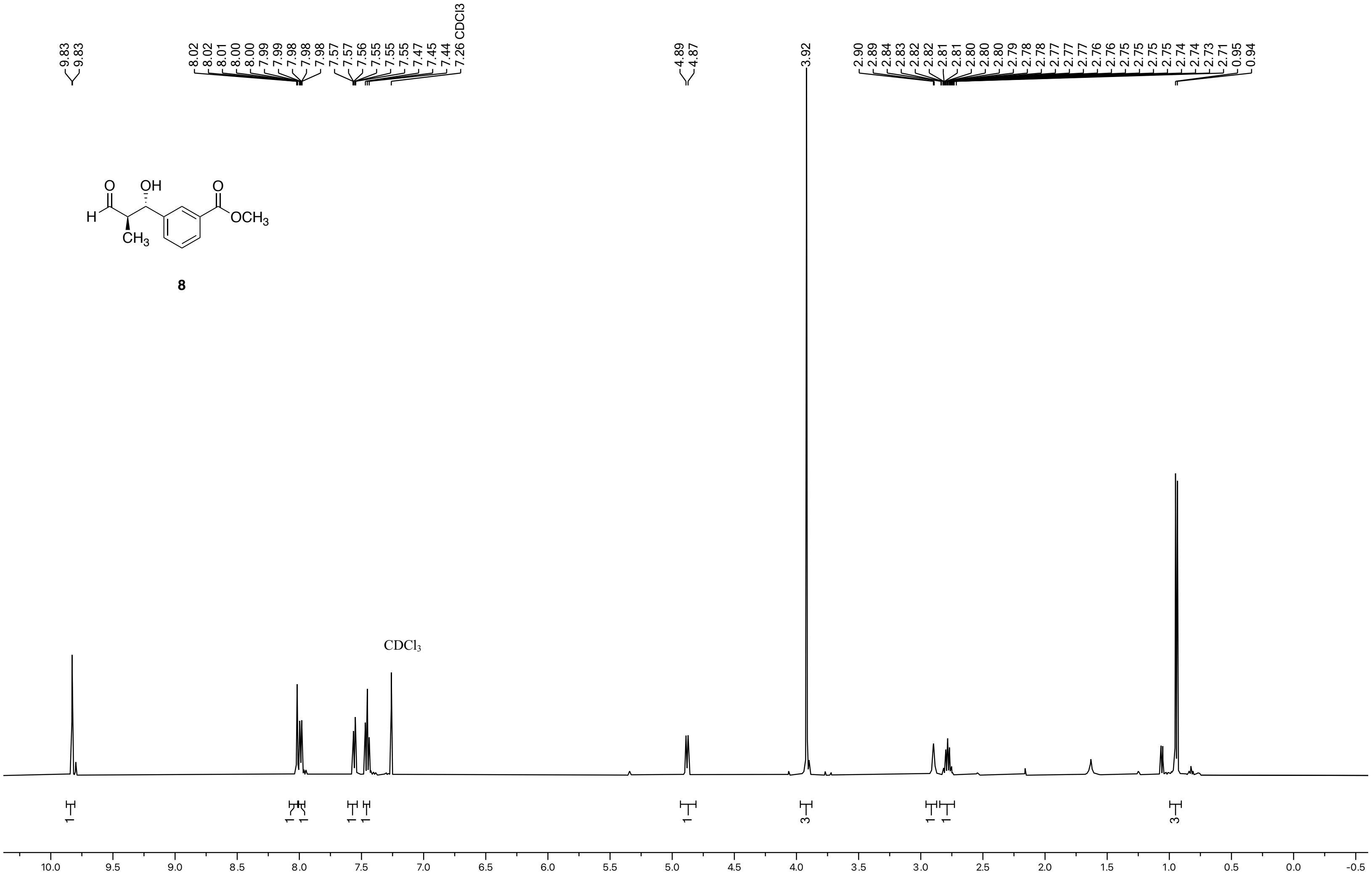

$^{13}C\{^{1}H\}$ NMR, 126 MHz, $CDCl_3$, **8**

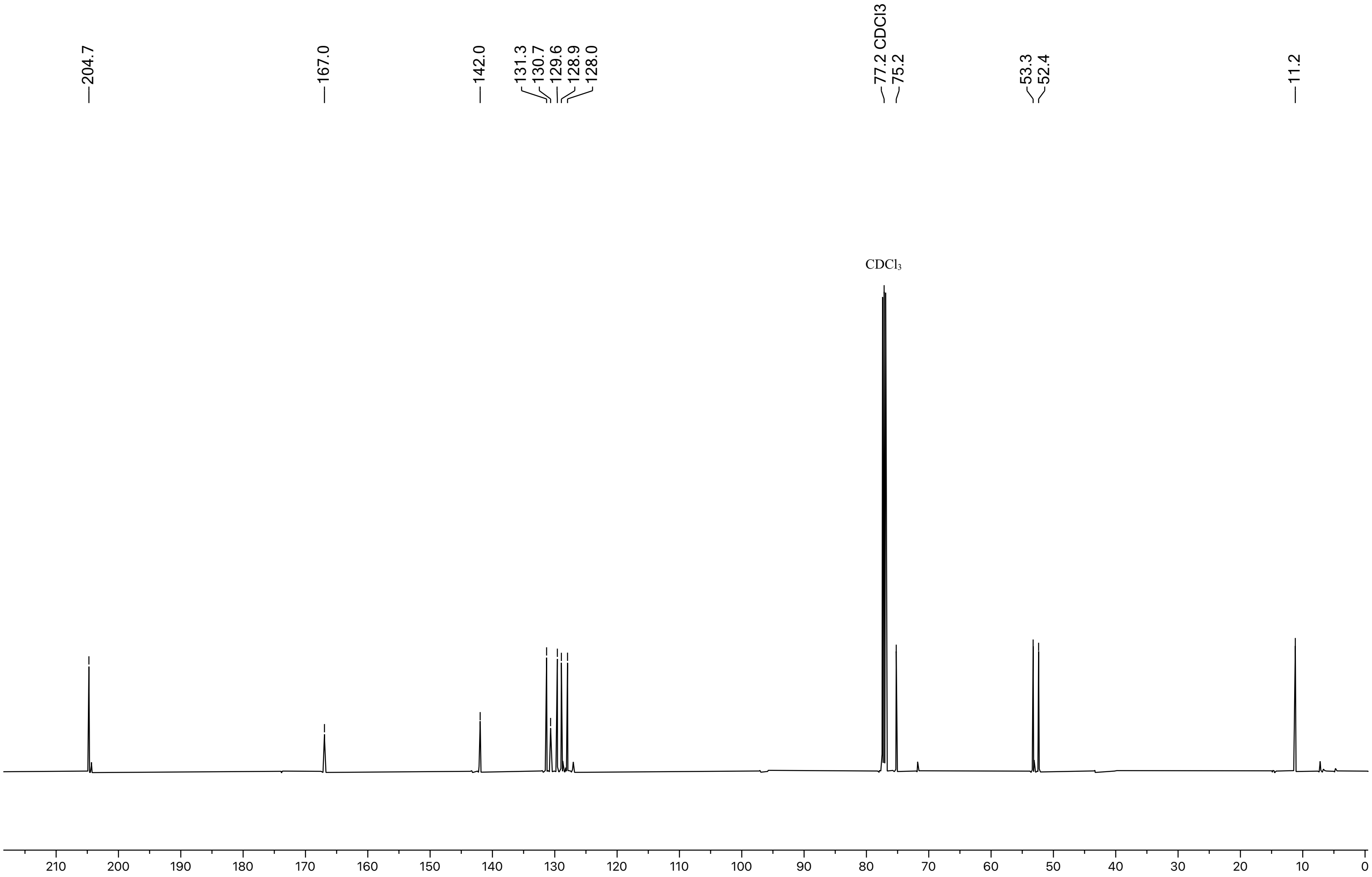

$^{1}H$ NMR, 500 MHz, $CDCl_3$, **10**

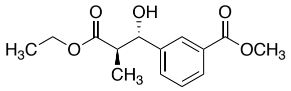

10

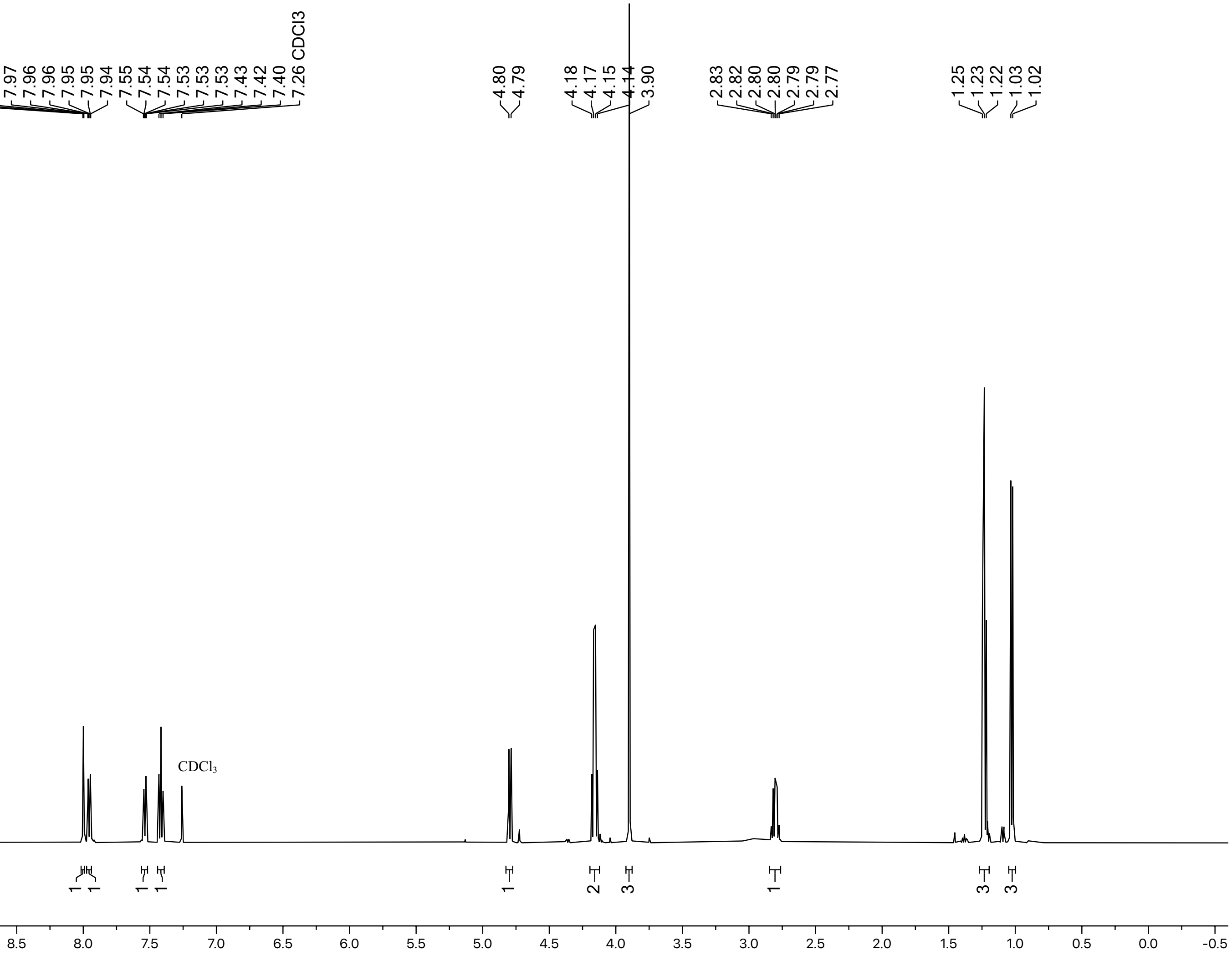

$^{13}C\{^1H\}$ NMR, 126 MHz, $CDCl_3$, **10**

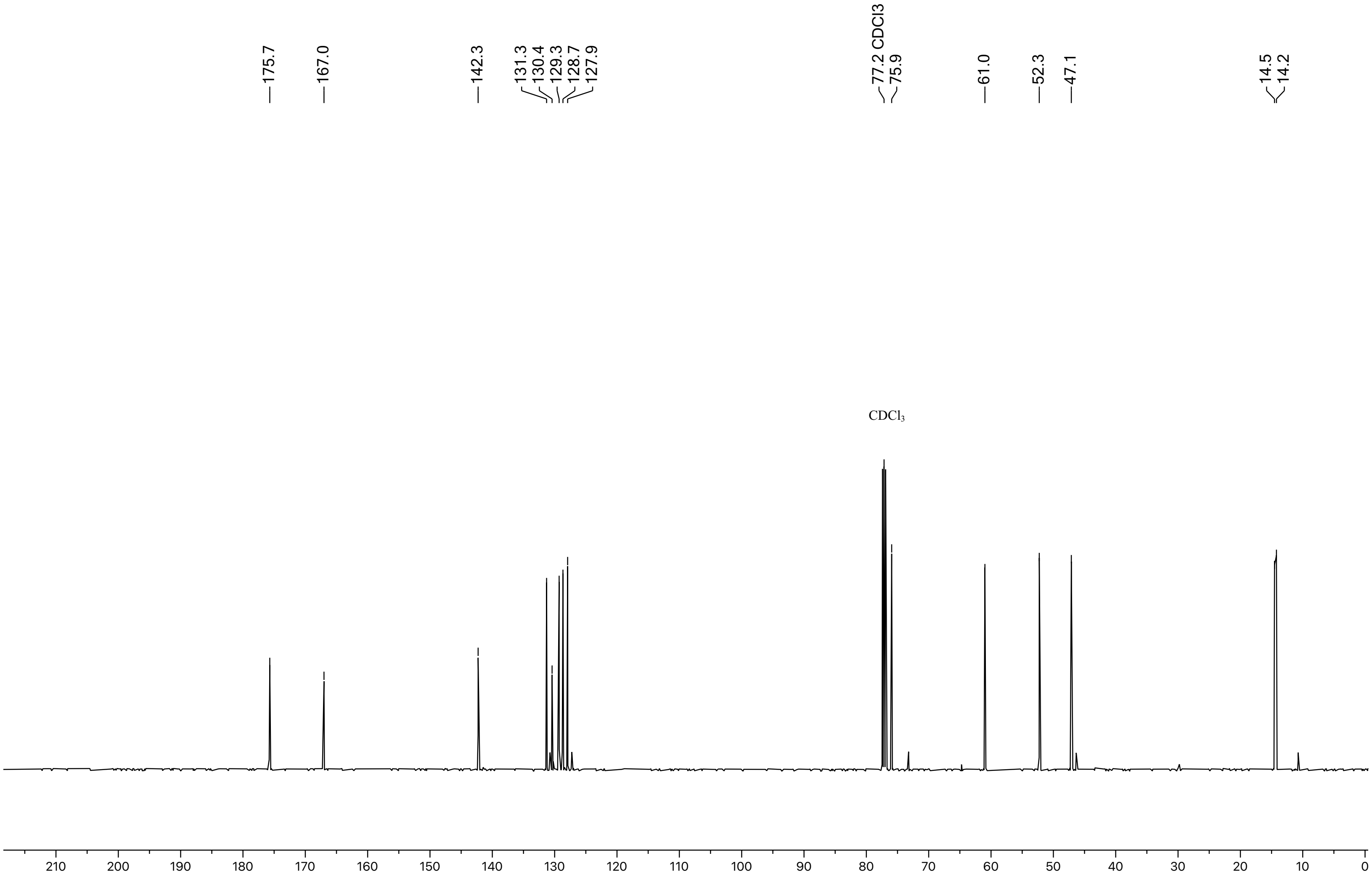

$^1H$ NMR, 500 MHz, $CDCl_3$, **11**

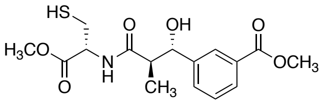

**11**

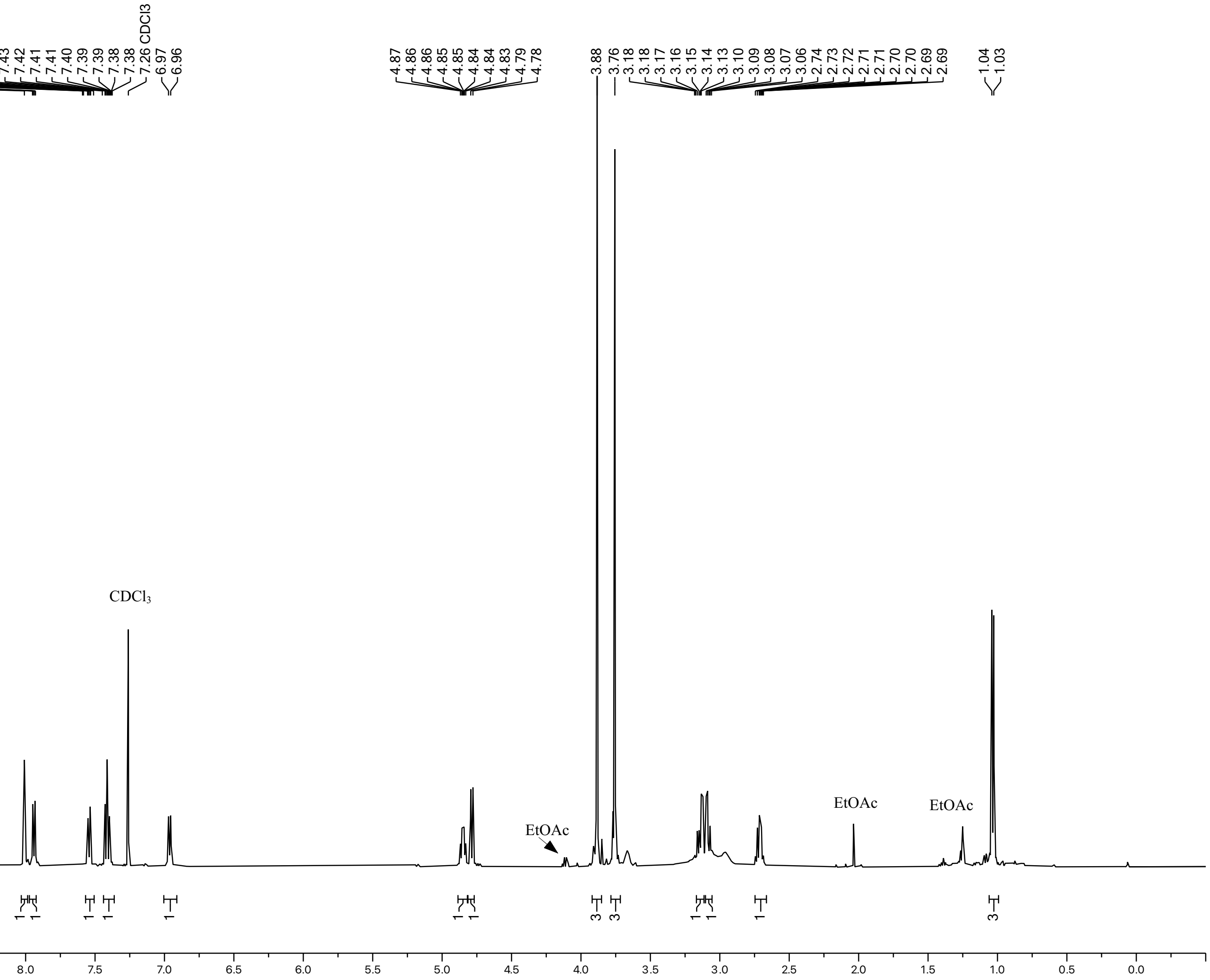

$^{13}C\{^{1}H\}$ NMR, 126 MHz, $CDCl_3$, **11**

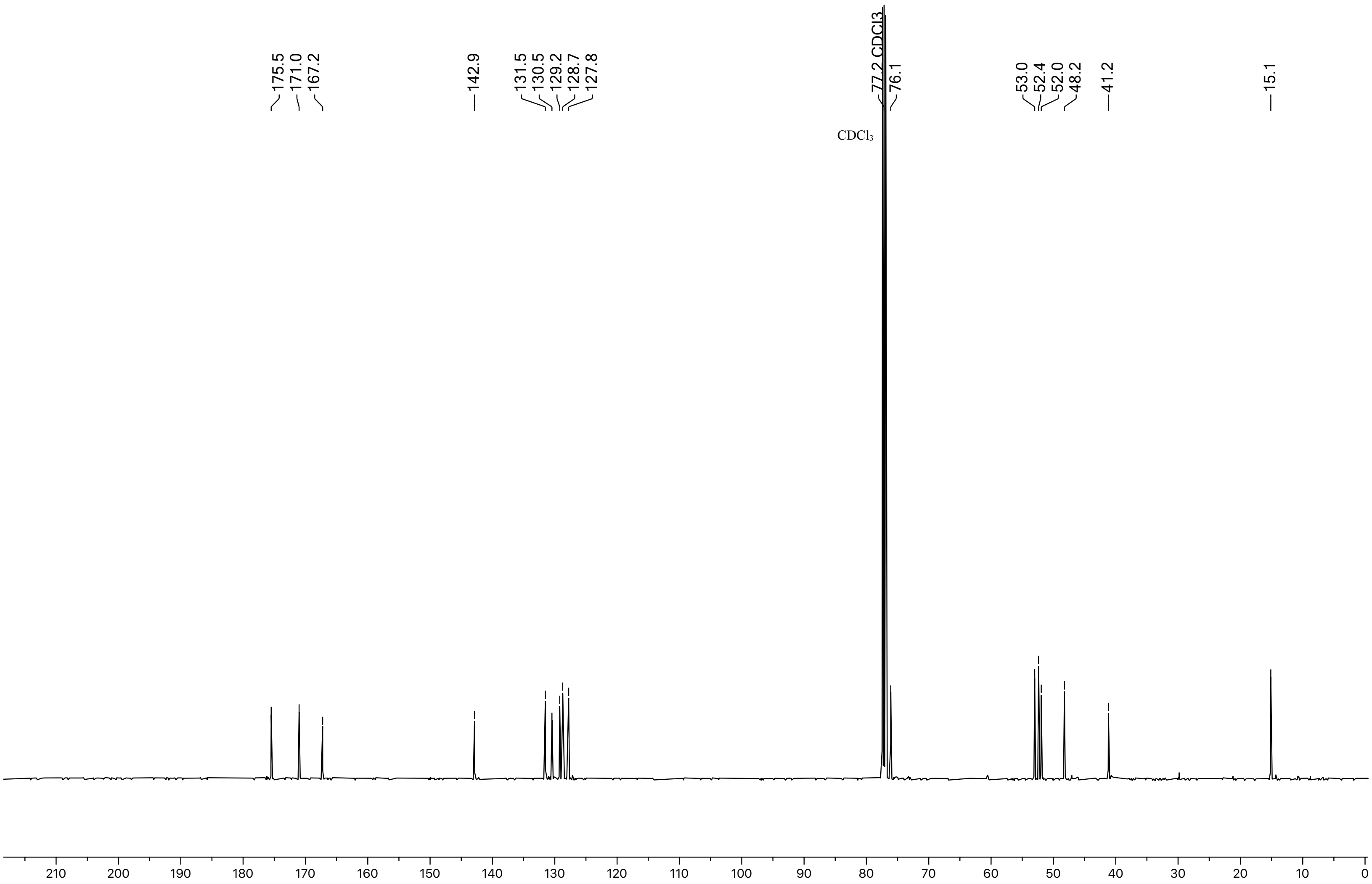

$^1$H NMR, 500 MHz, $CDCl_3$, **12**

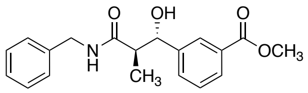


**12**

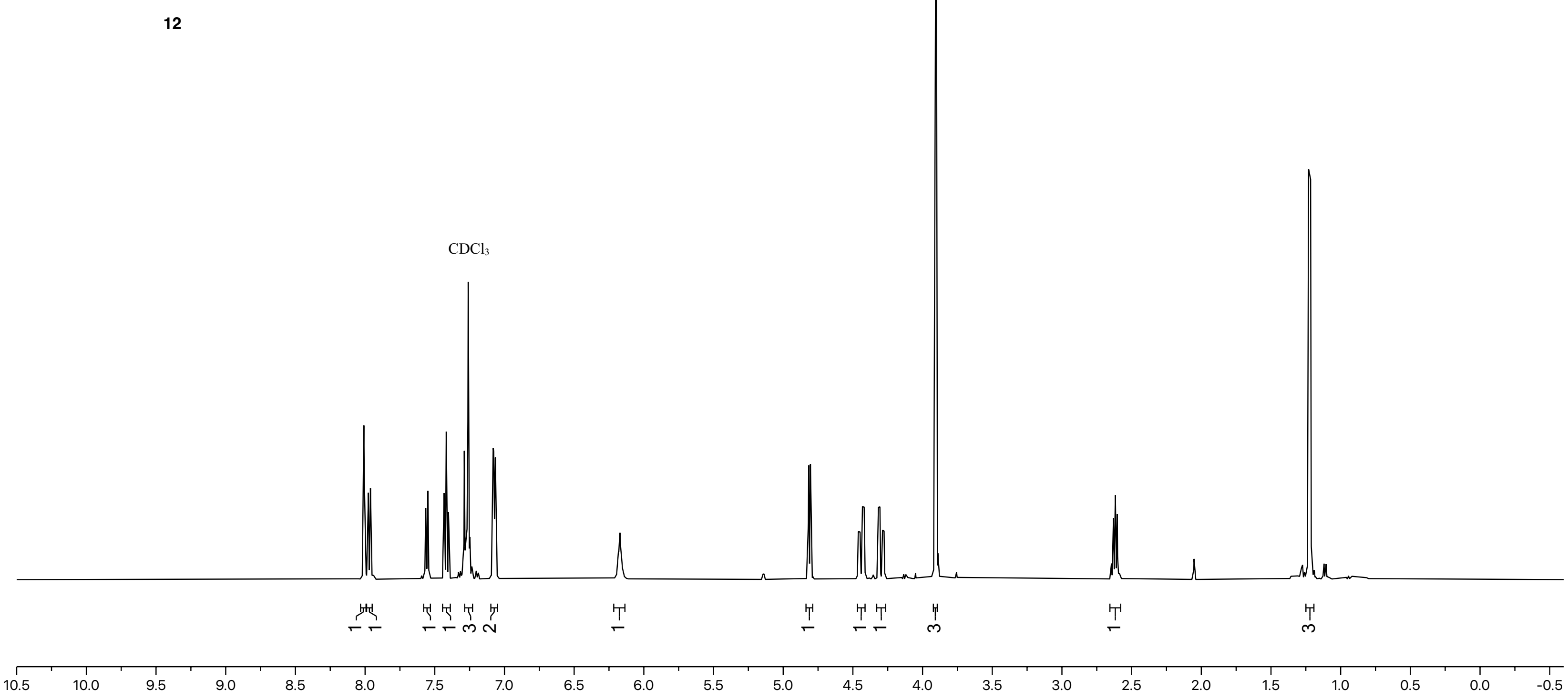

$^{13}C\{^{1}H\}$ NMR, 126 MHz, $CDCl_3$, **12**

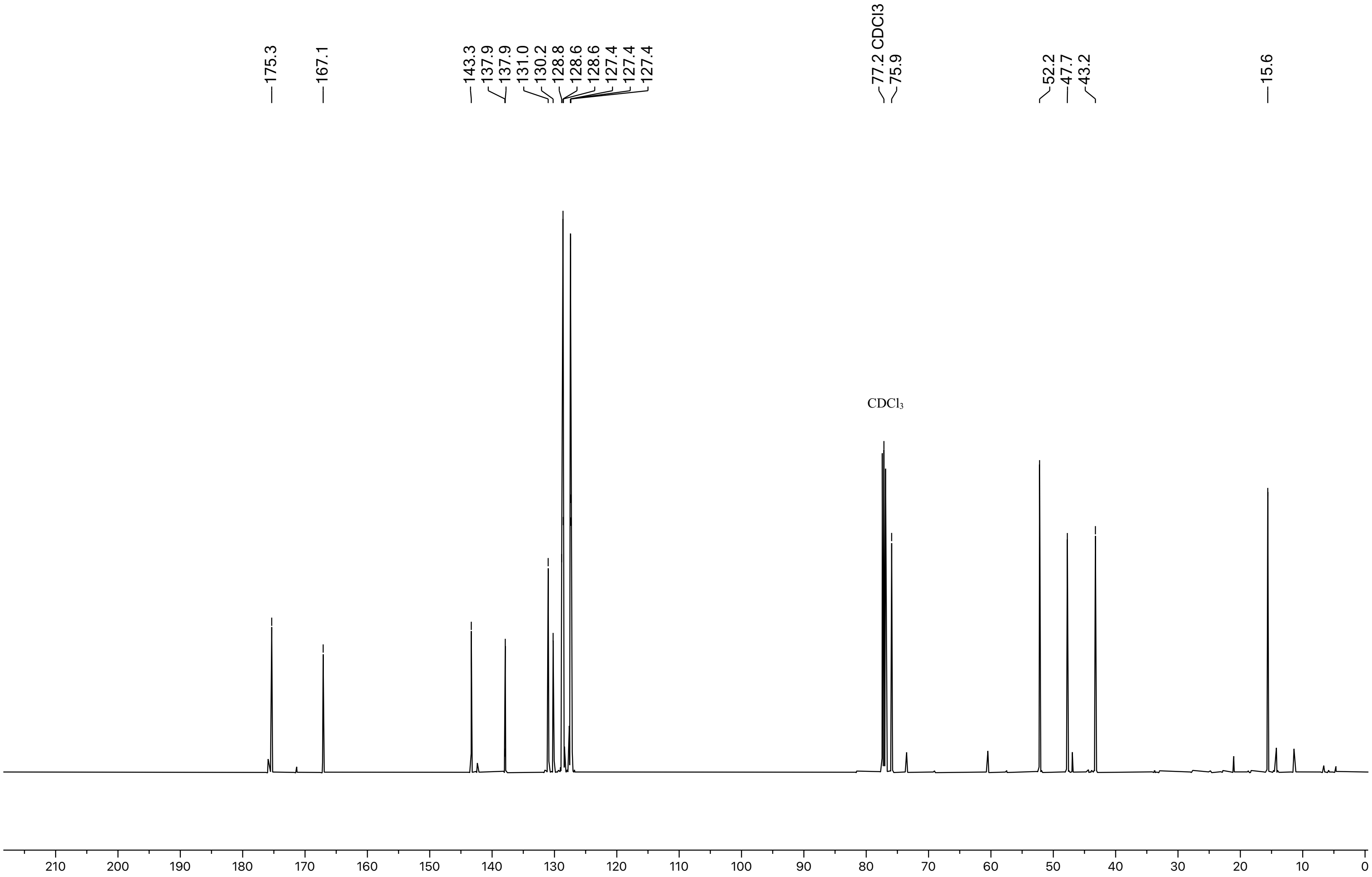

[1]H NMR, 500 MHz, $CDCl_3$, **9**

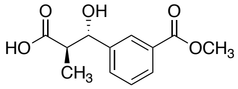

**9**

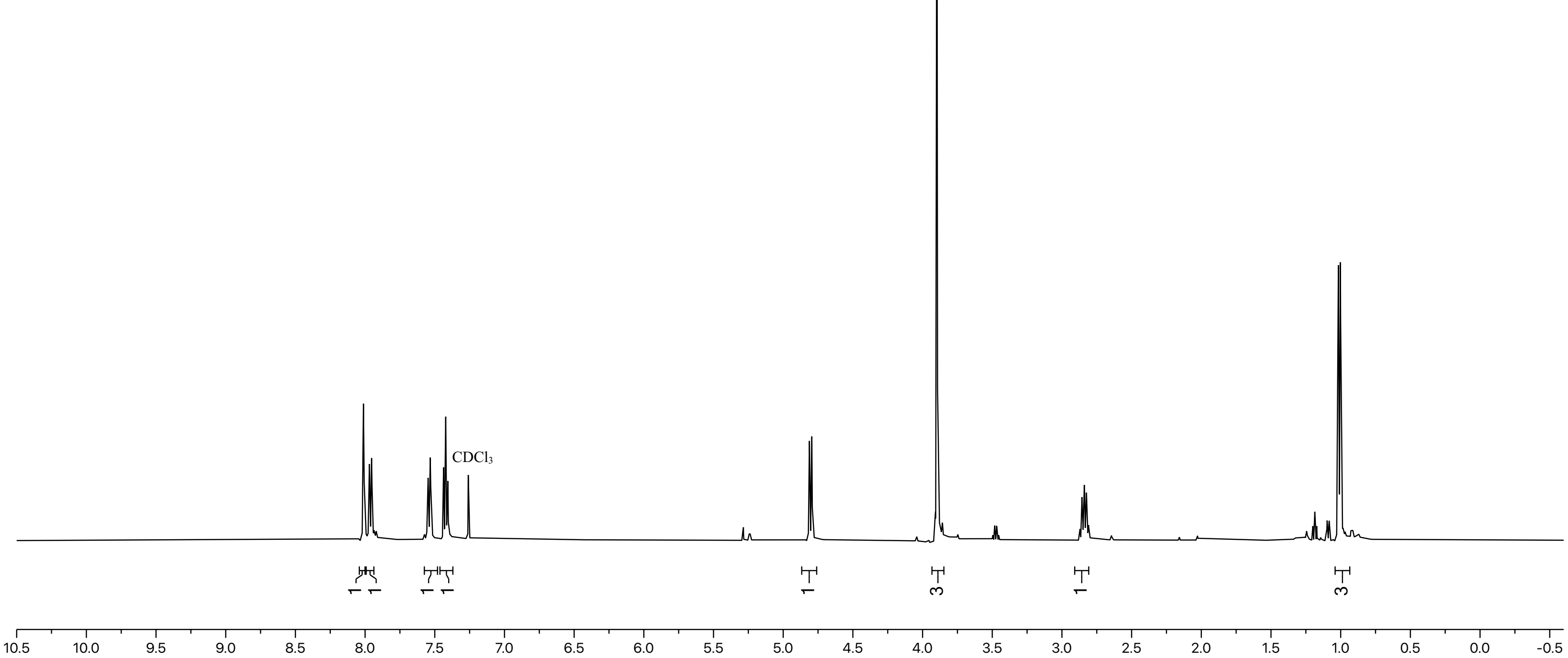

$^{13}C\{^1H\}$ NMR, 126 MHz, $CDCl_3$, **9**

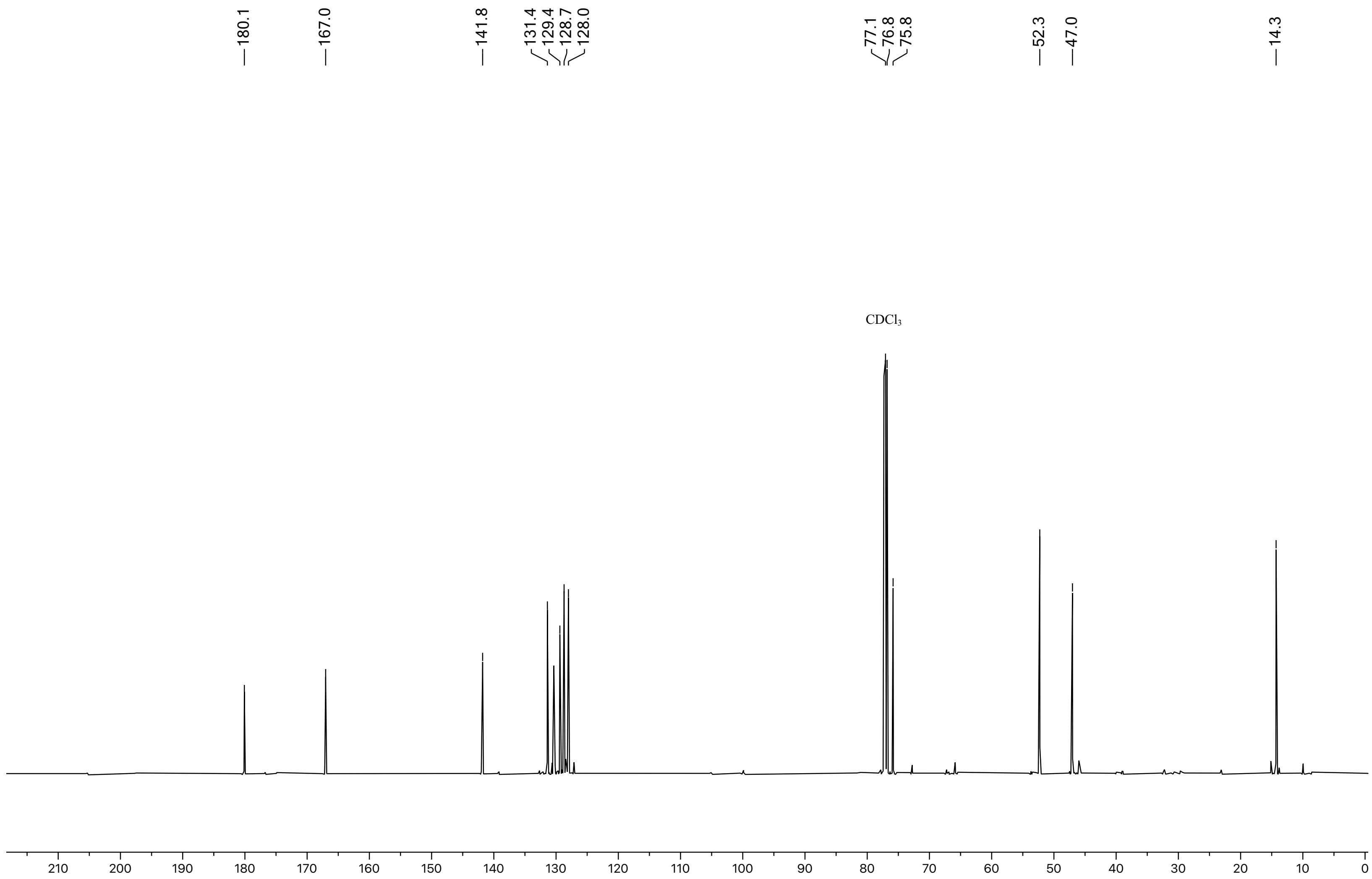

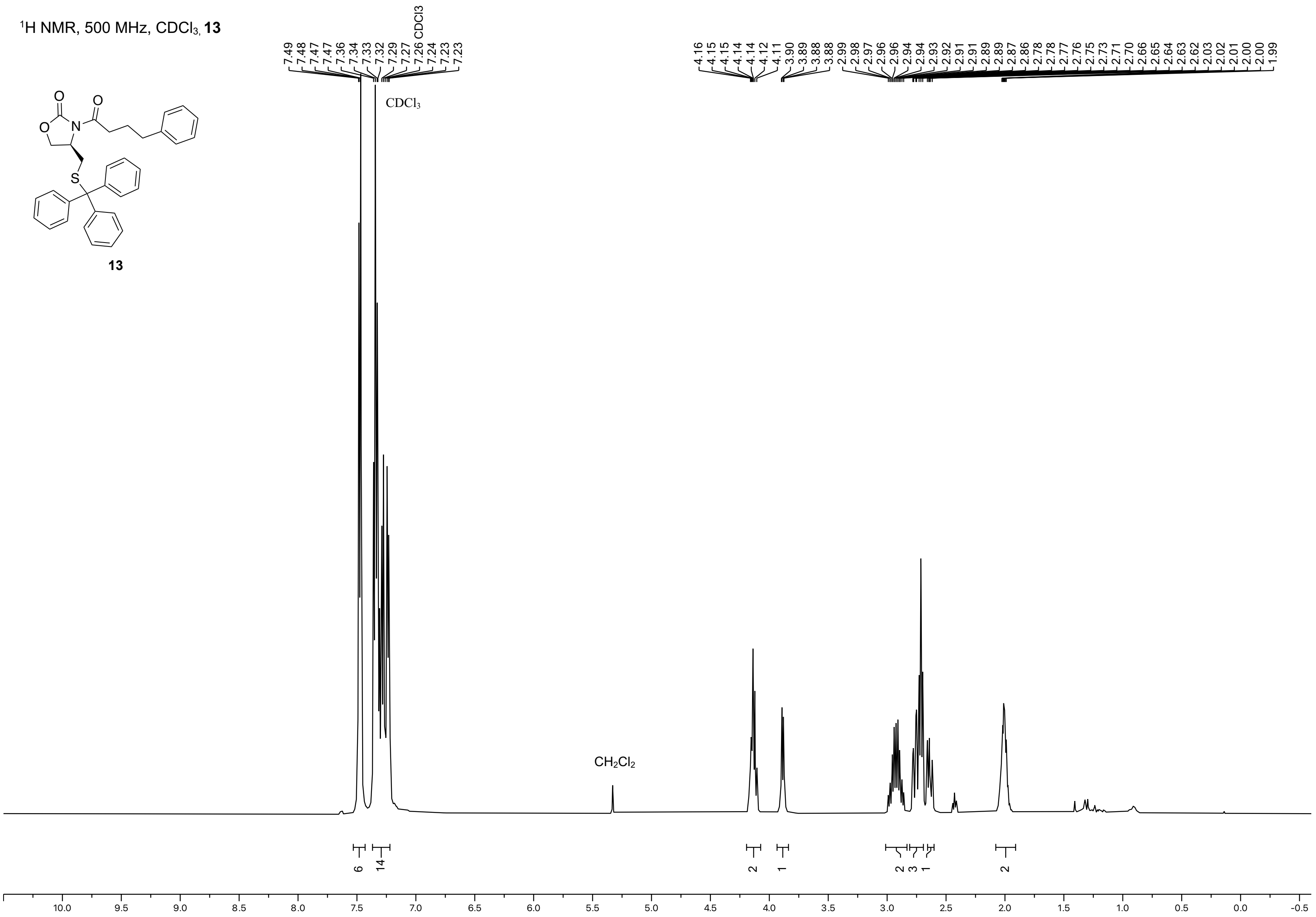

$^1$H NMR, 500 MHz, $CDCl_3$, **13**
13
$CDCl_3$
$CH_2Cl_2$

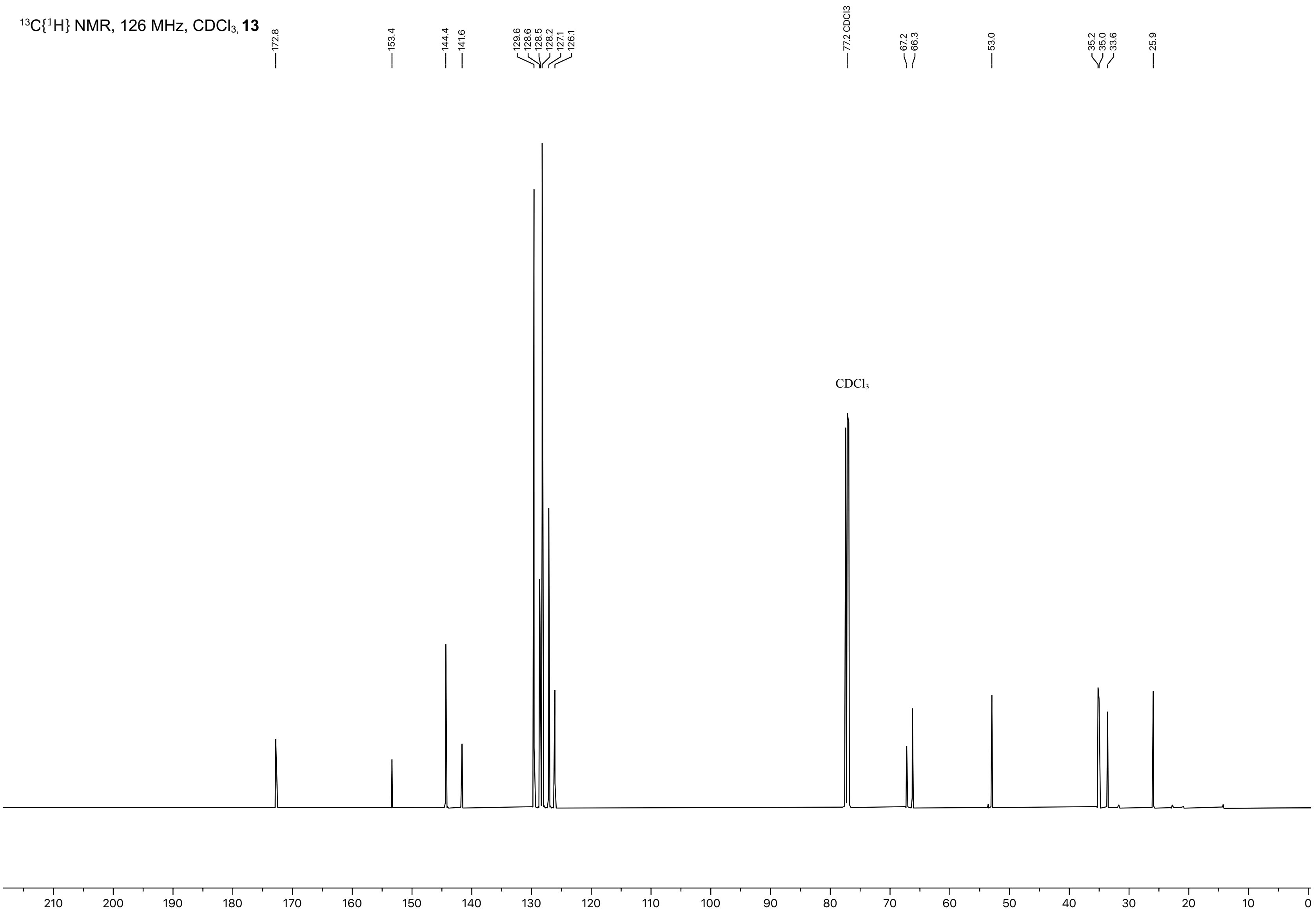

13C{1H} NMR, 126 MHz, CDCl3, 13
172.8
153.4
144.4
141.6
129.6
128.6
128.5
128.2
127.1
126.1
77.2 CDCl3
67.2
66.3
53.0
35.2
35.0
33.6
25.9
CDCl3
210 200 190 180 170 160 150 140 130 120 110 100 90 80 70 60 50 40 30 20 10 0

$^{1}$H NMR, 500 MHz, $CDCl_3$, (*S*)-**14**

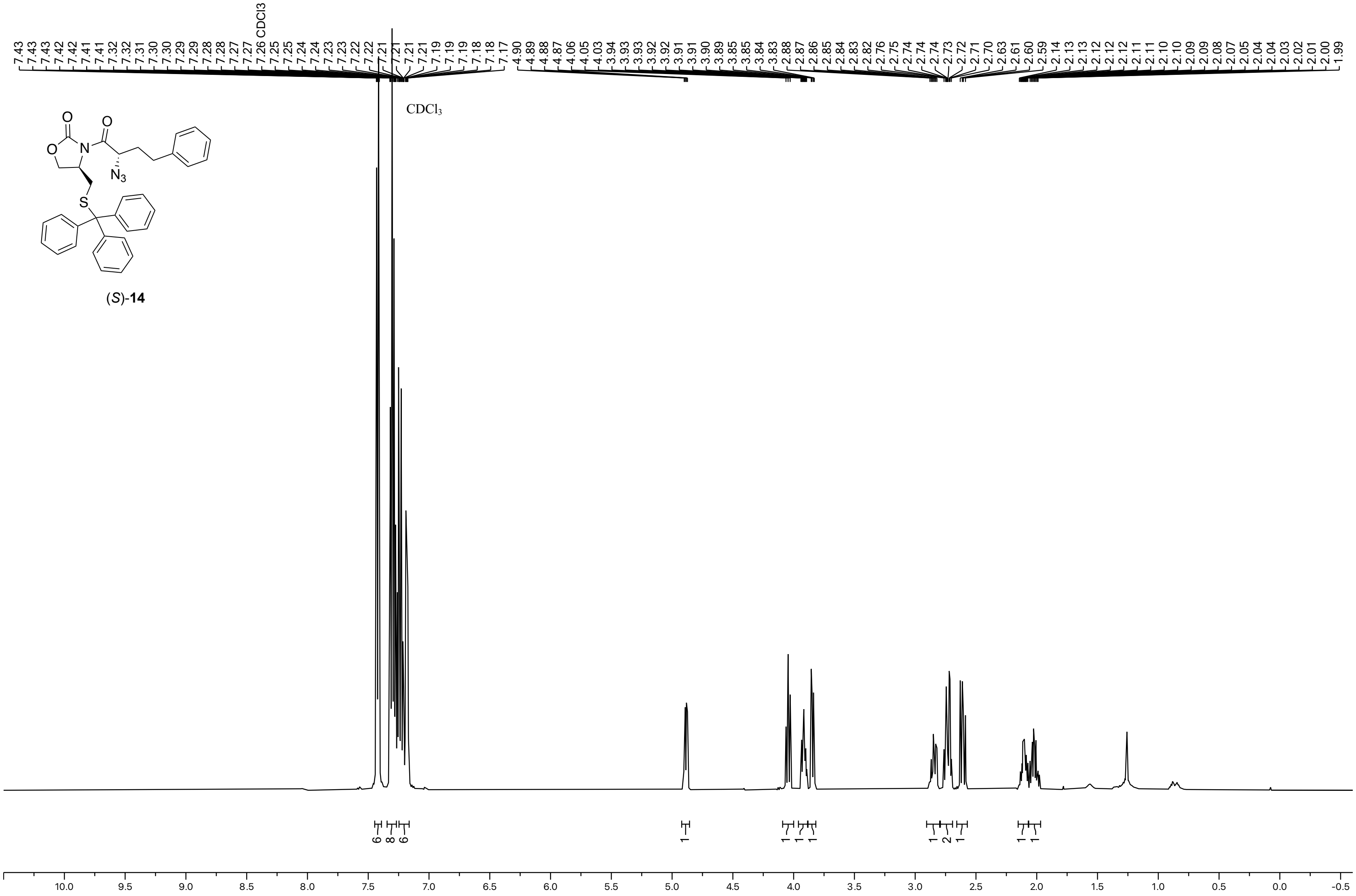

$^{13}C\{^1H\}$ NMR, 126 MHz, $CDCl_3$, (*S*)-**14**

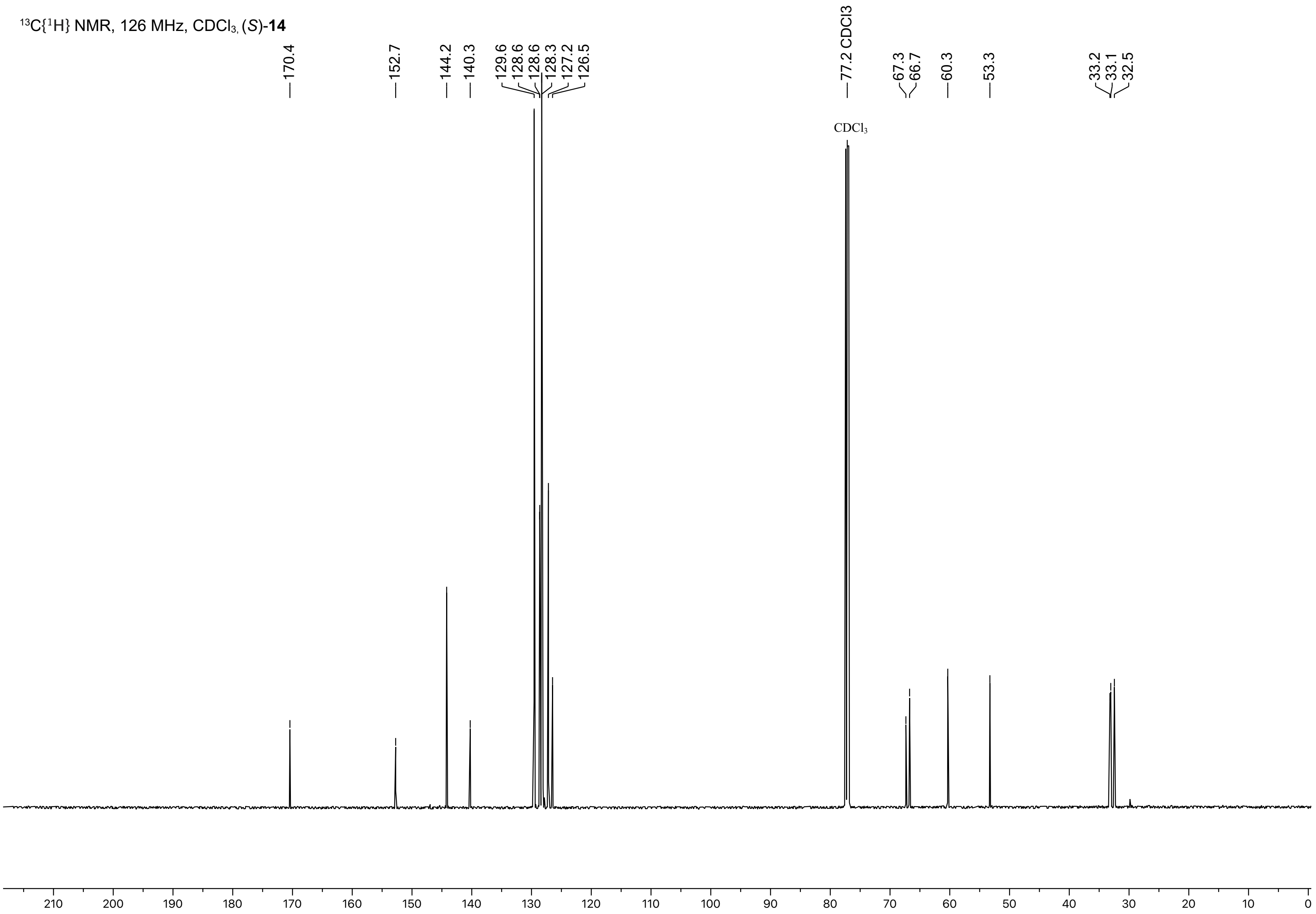

$^1$H NMR, 500 MHz, $CDCl_3$, (*S*)-**15**

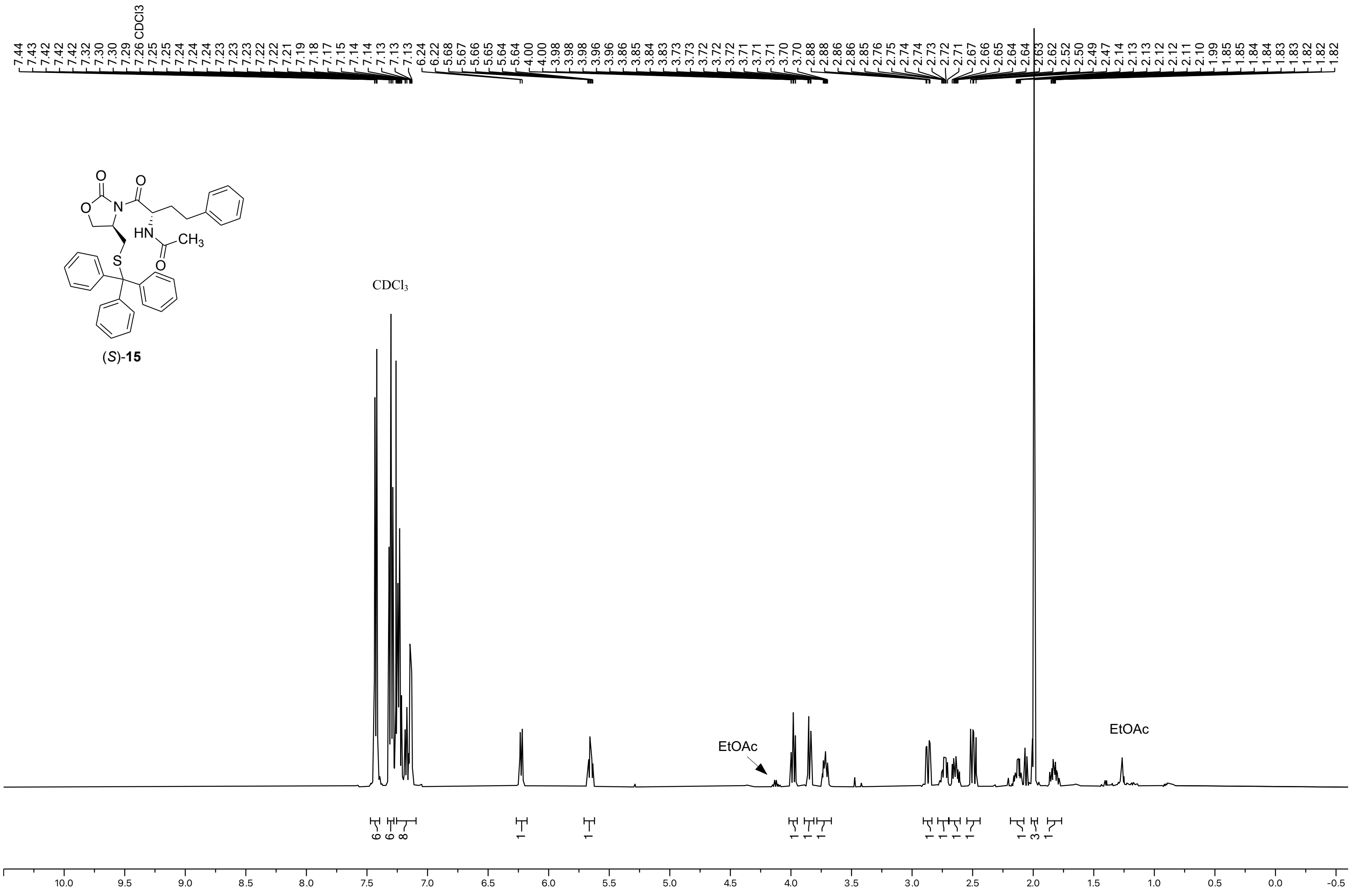

$^{13}C\{^{1}H\}$ NMR, 126 MHz, $CDCl_3$, (*S*)-**15**

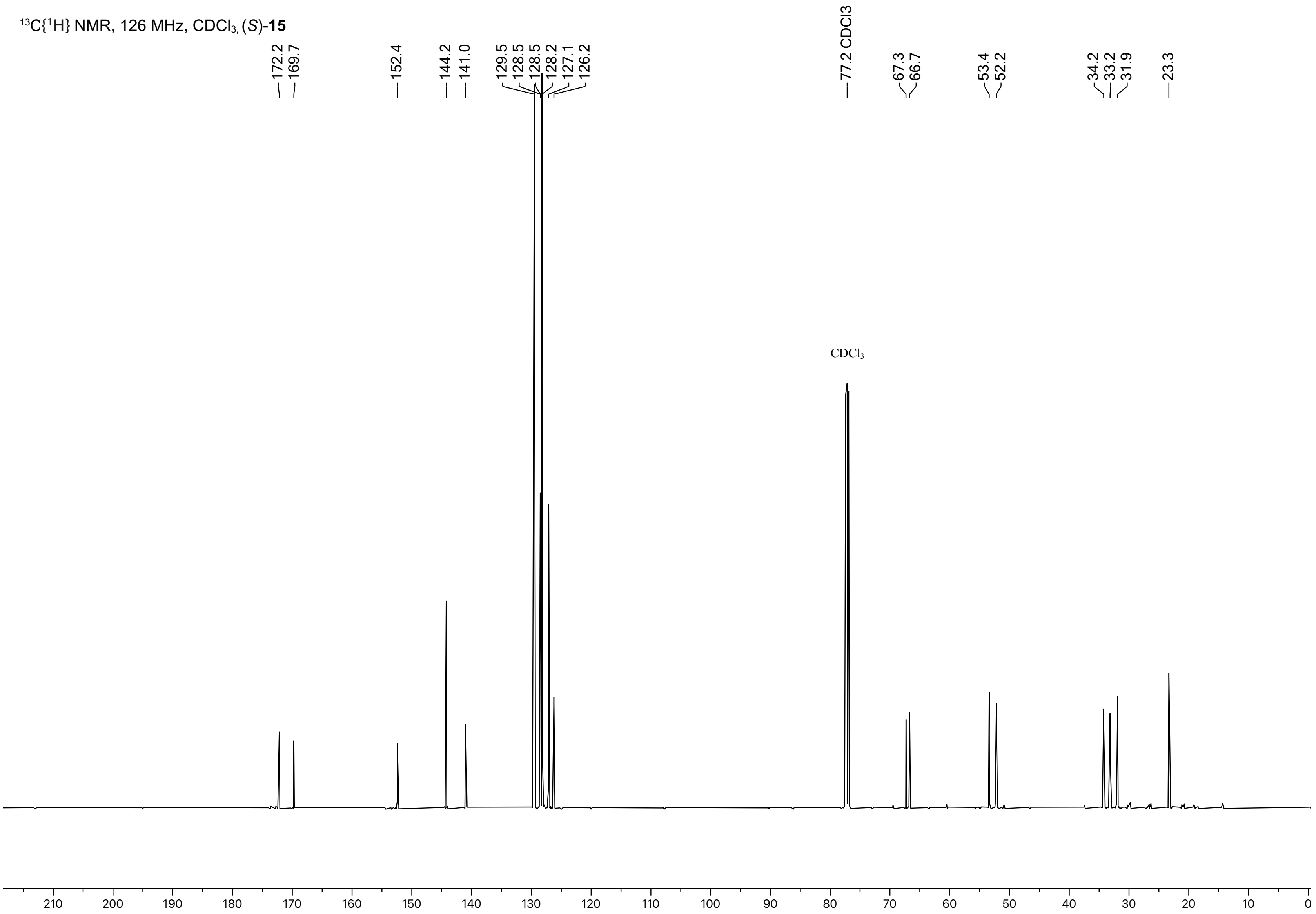

$^1H$ NMR, 500 MHz, $CD_3OD$, **16**

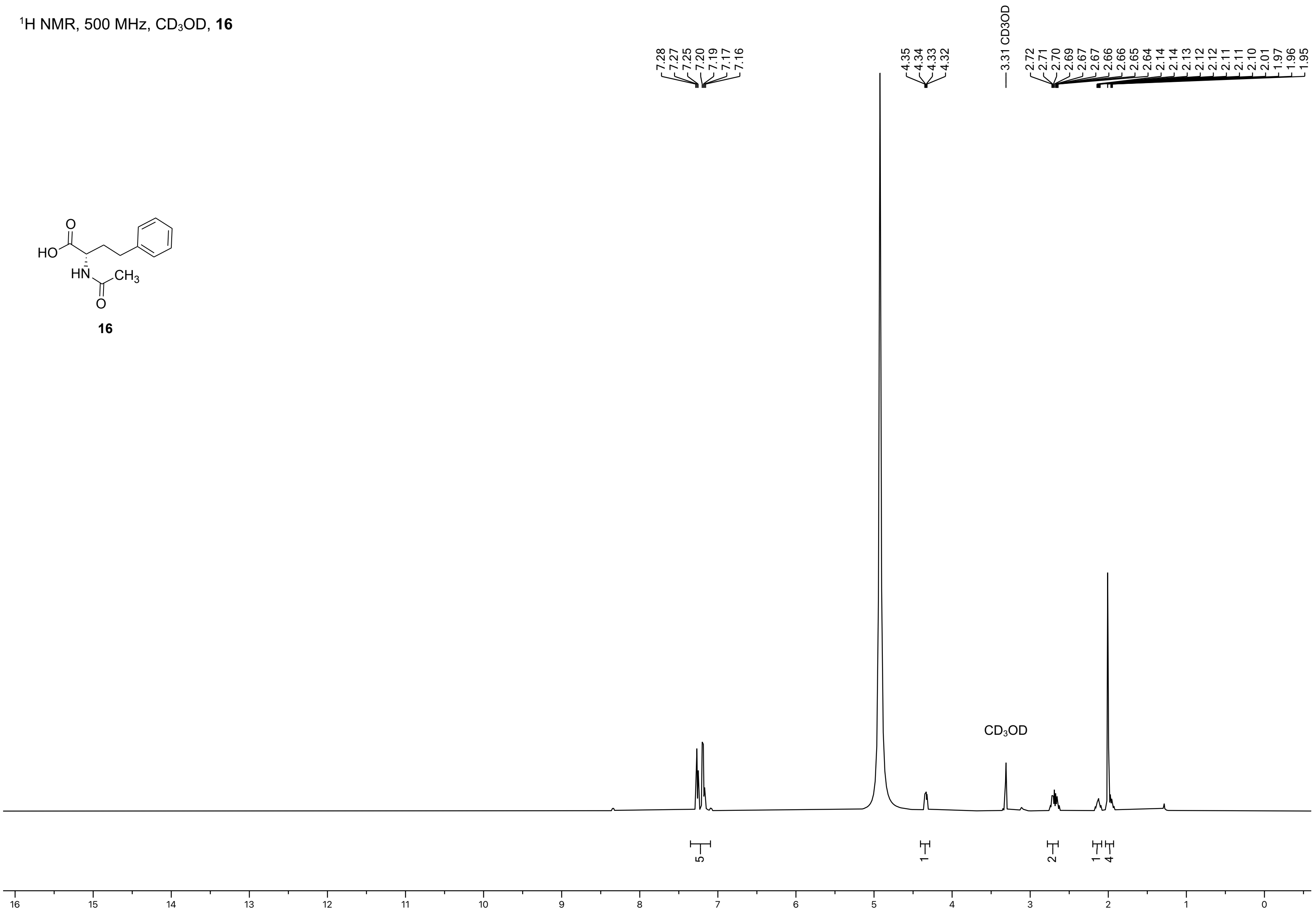

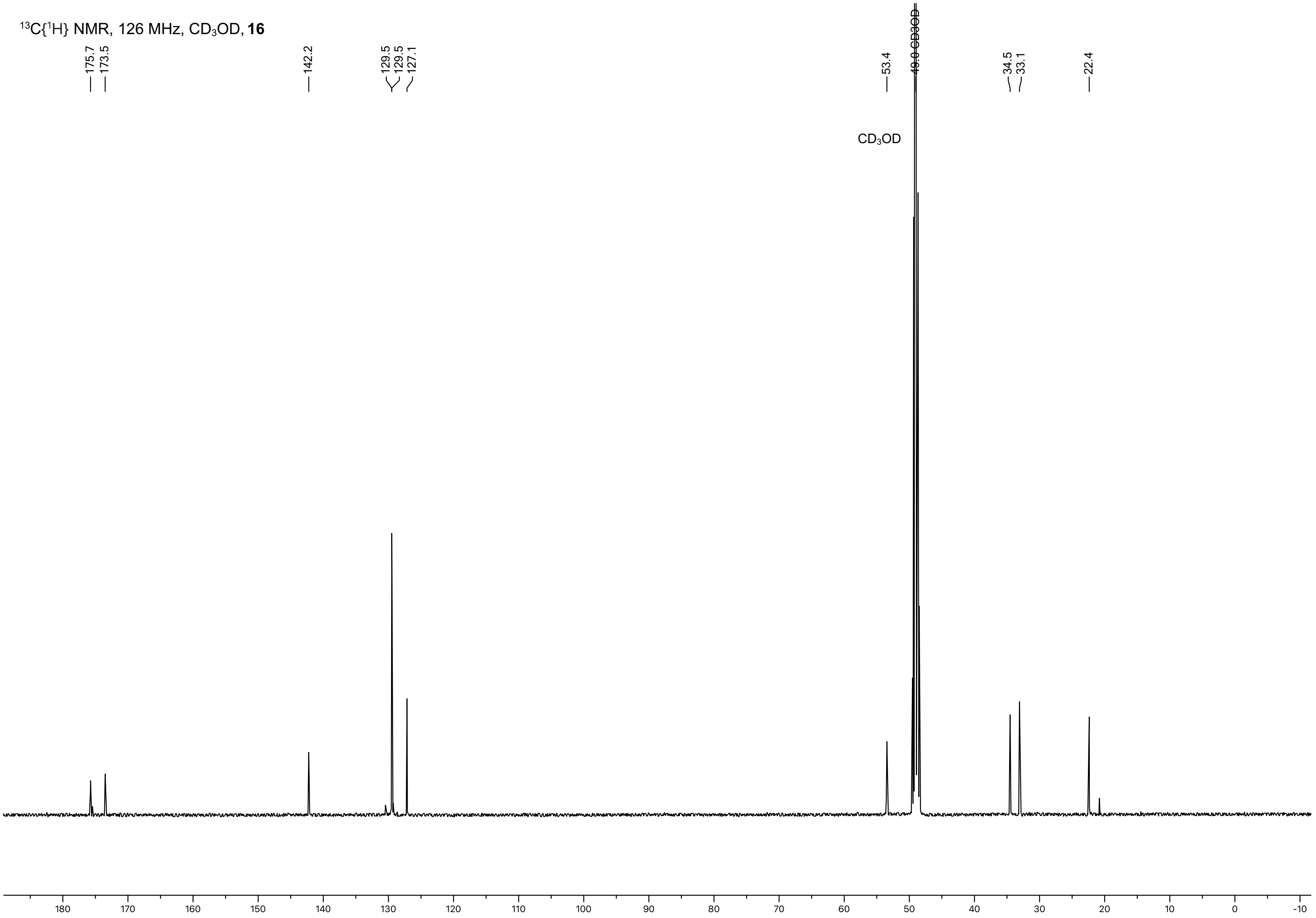

$^{13}C\{^1H\}$ NMR, 126 MHz, $CD_3OD$, **16**
175.7
173.5
142.2
129.5
129.5
127.1
53.4
49.0 CD3OD
34.5
33.1
22.4
$CD_3OD$
180 170 160 150 140 130 120 110 100 90 80 70 60 50 40 30 20 10 0 -10

$^{1}H$ NMR, 500 MHz, $CDCl_3$, **17**

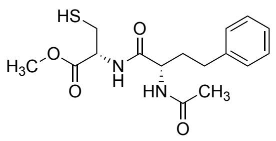

**17**

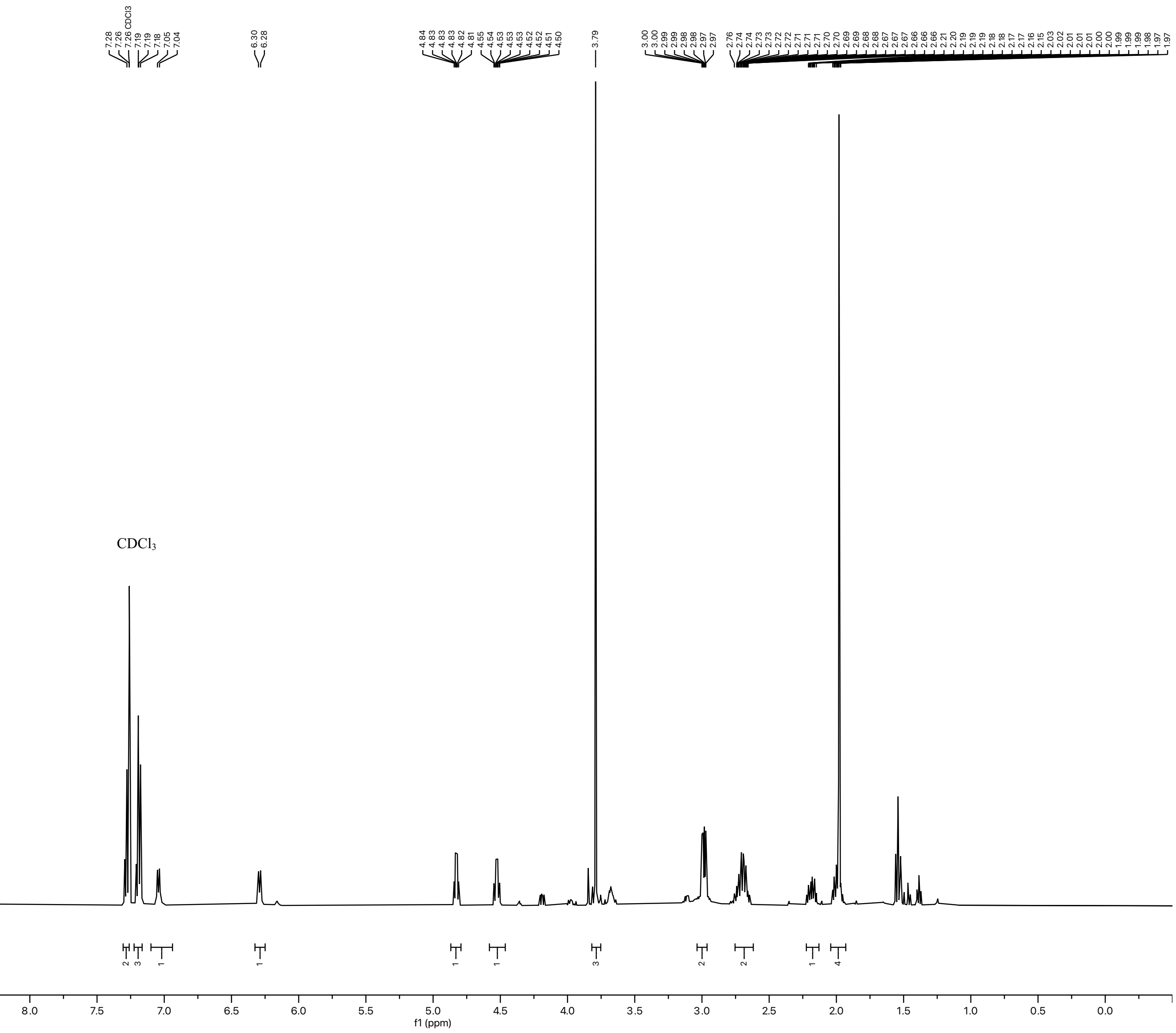

$^{13}C\{^1H\}$ NMR, 126 MHz, $CDCl_3$, **17**

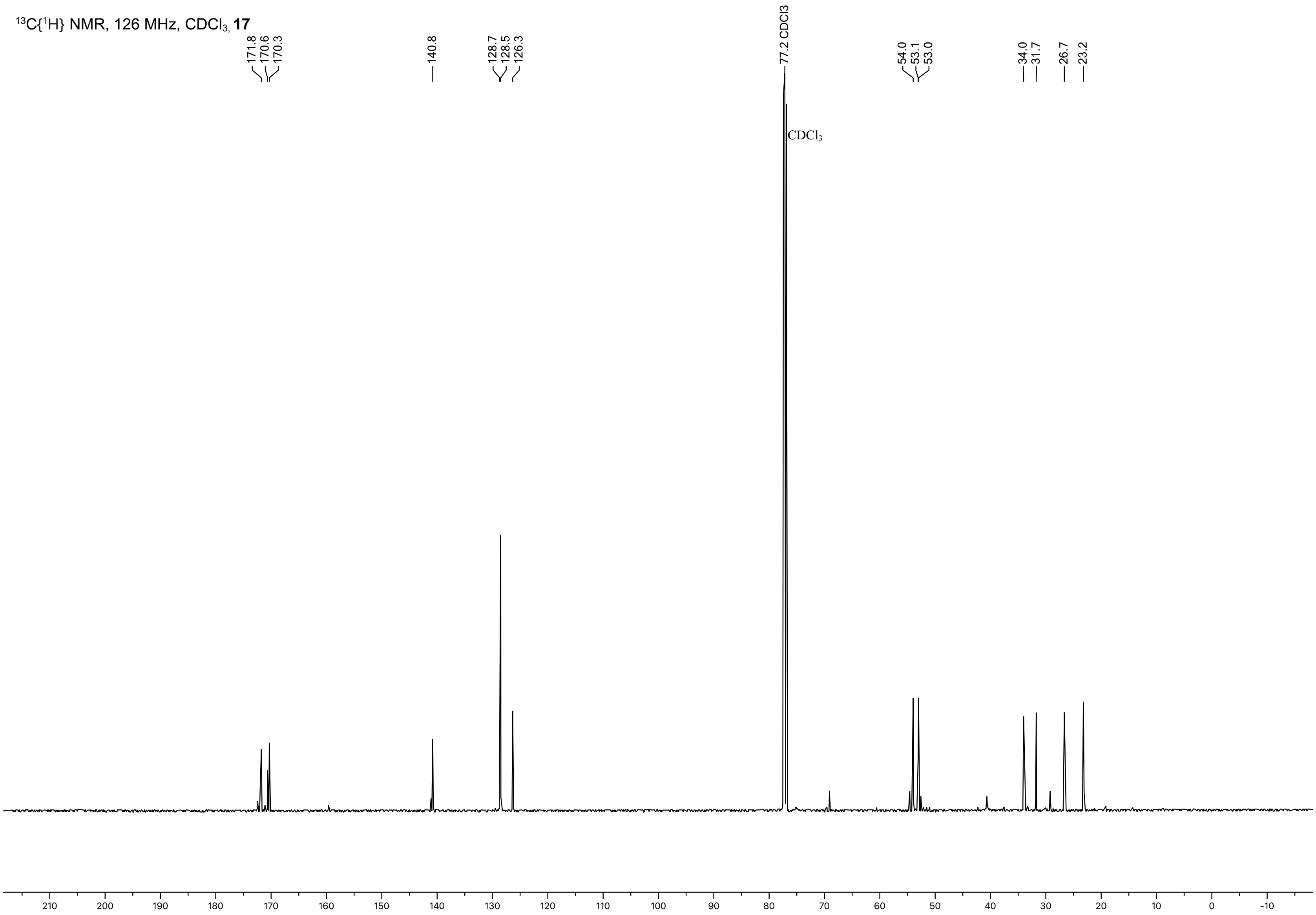

$^1$H NMR, 500 MHz, CDCl$_3$, **S6**

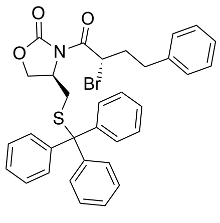

S6

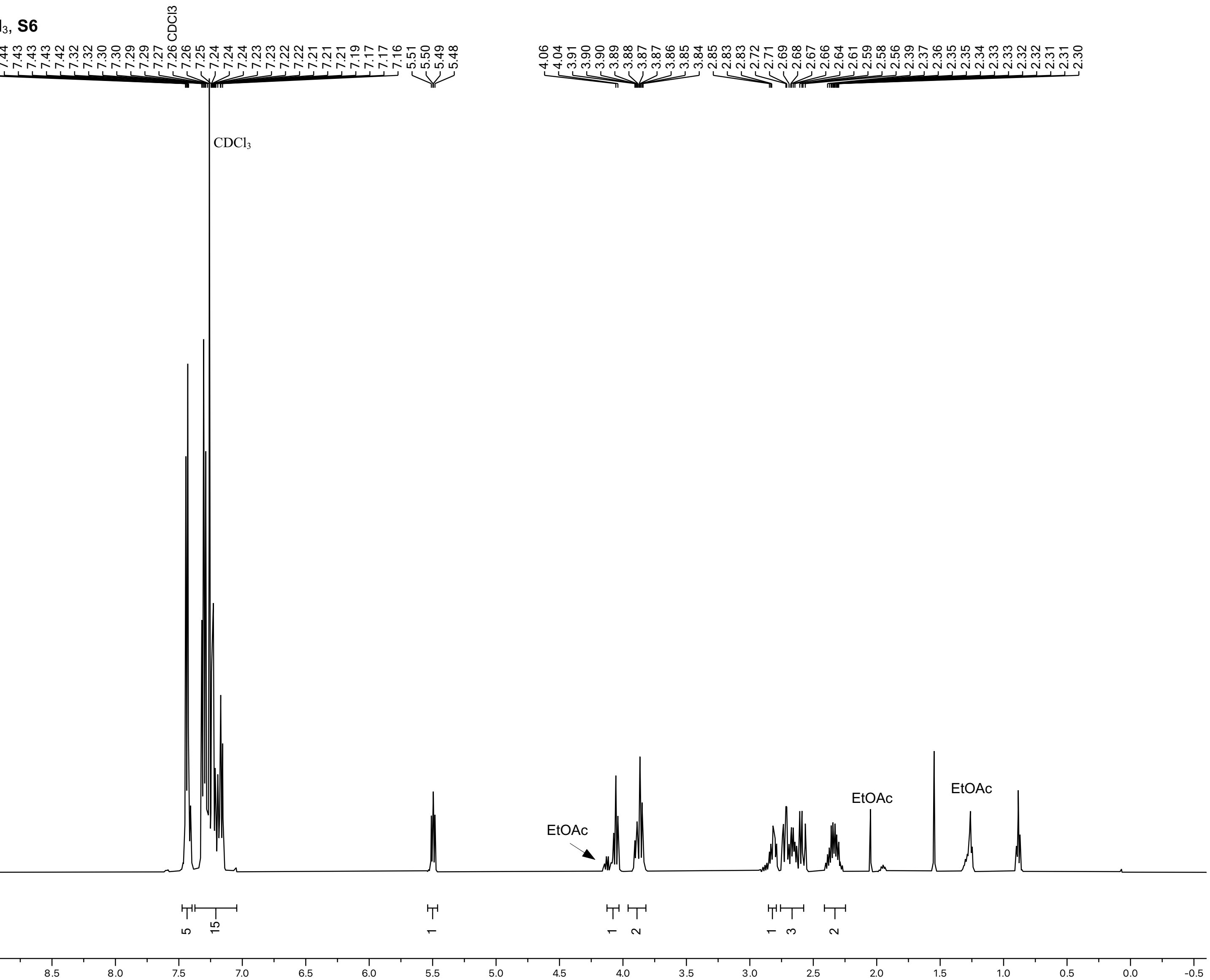

$^{13}C\{^{1}H\}$ NMR, 126 MHz, $CDCl_3$, **S6**

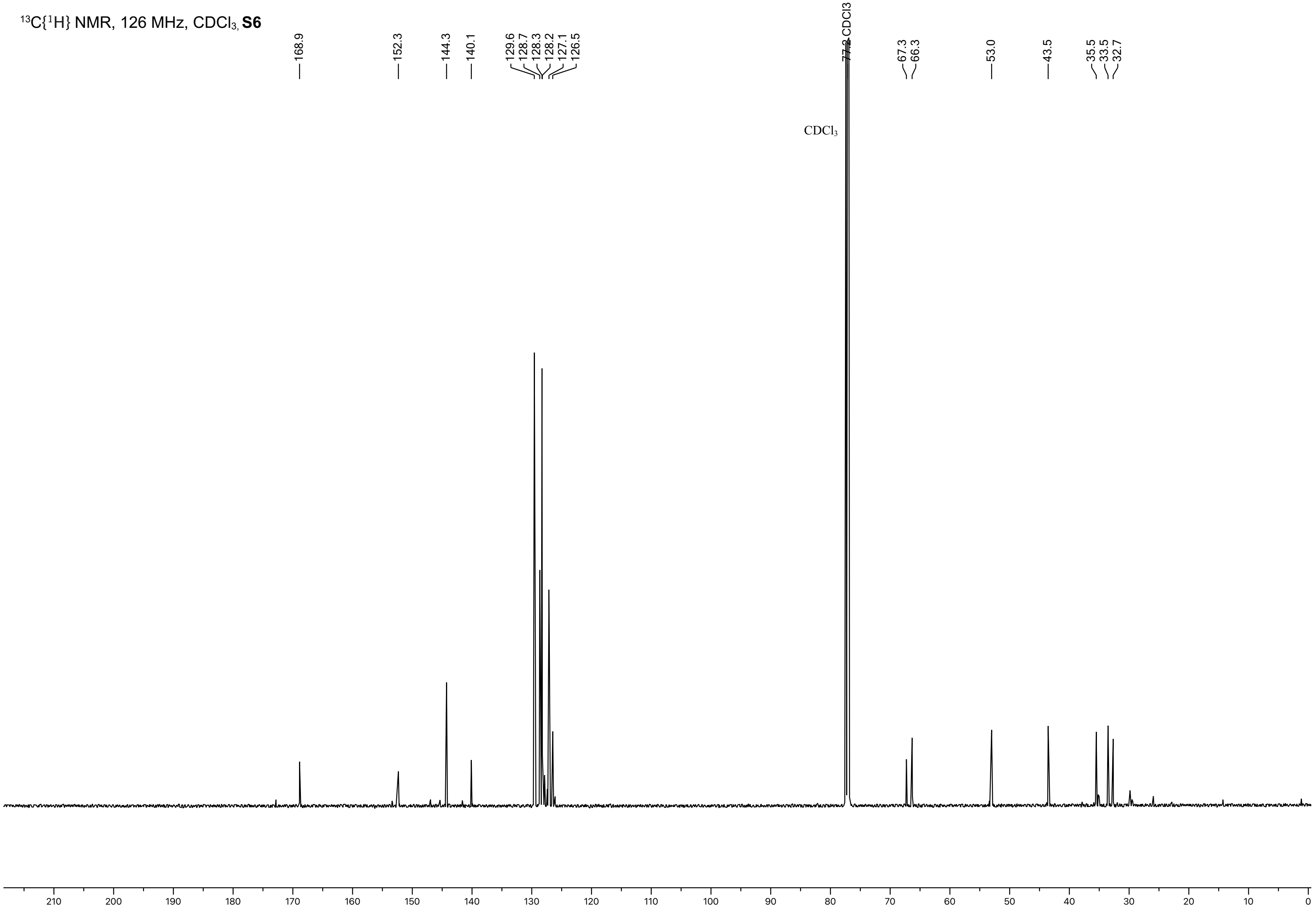

$^1$H NMR, 500 MHz, $CDCl_3$, (*R*)-**14**

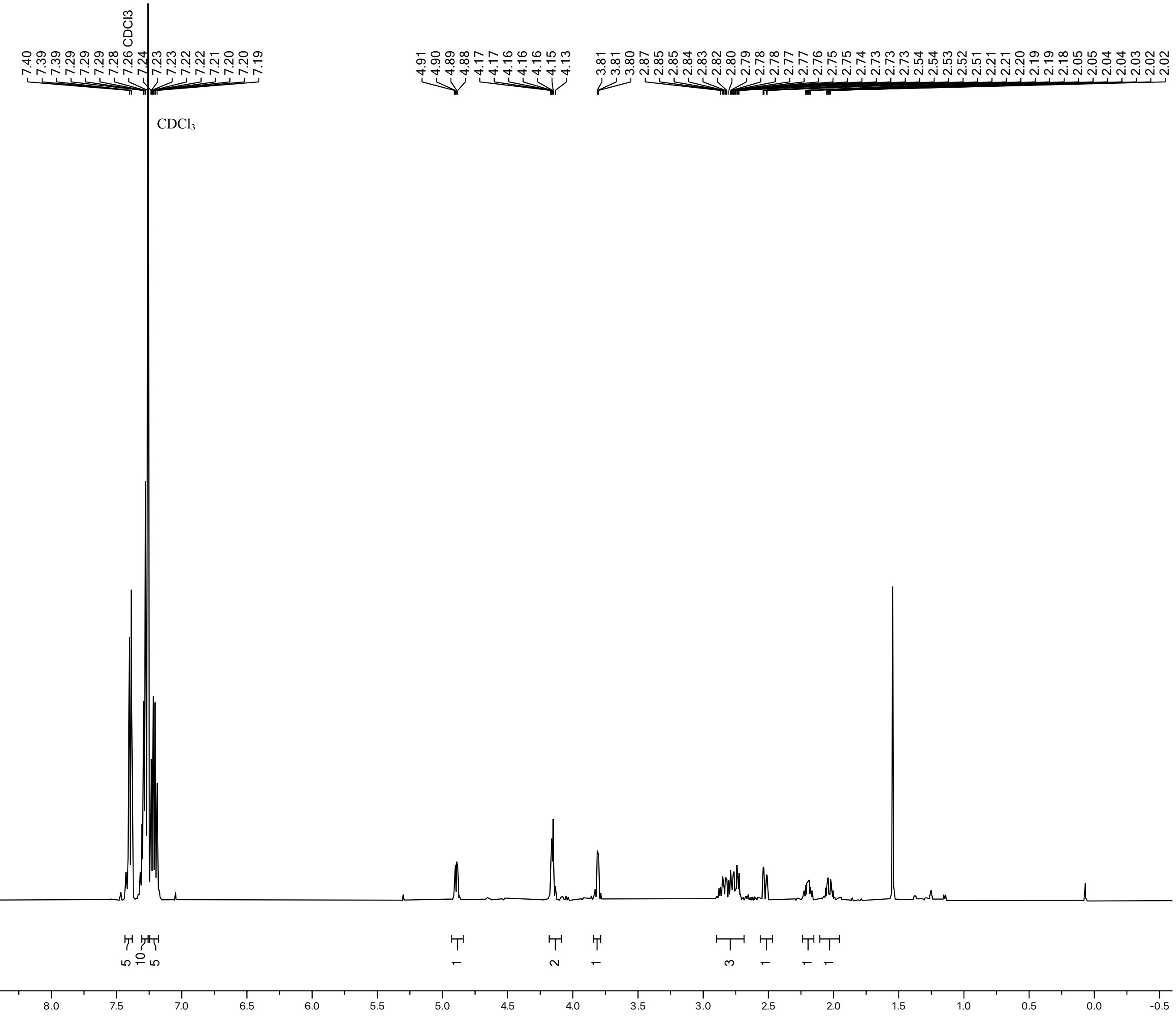


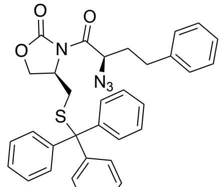


(*R*)-**14**

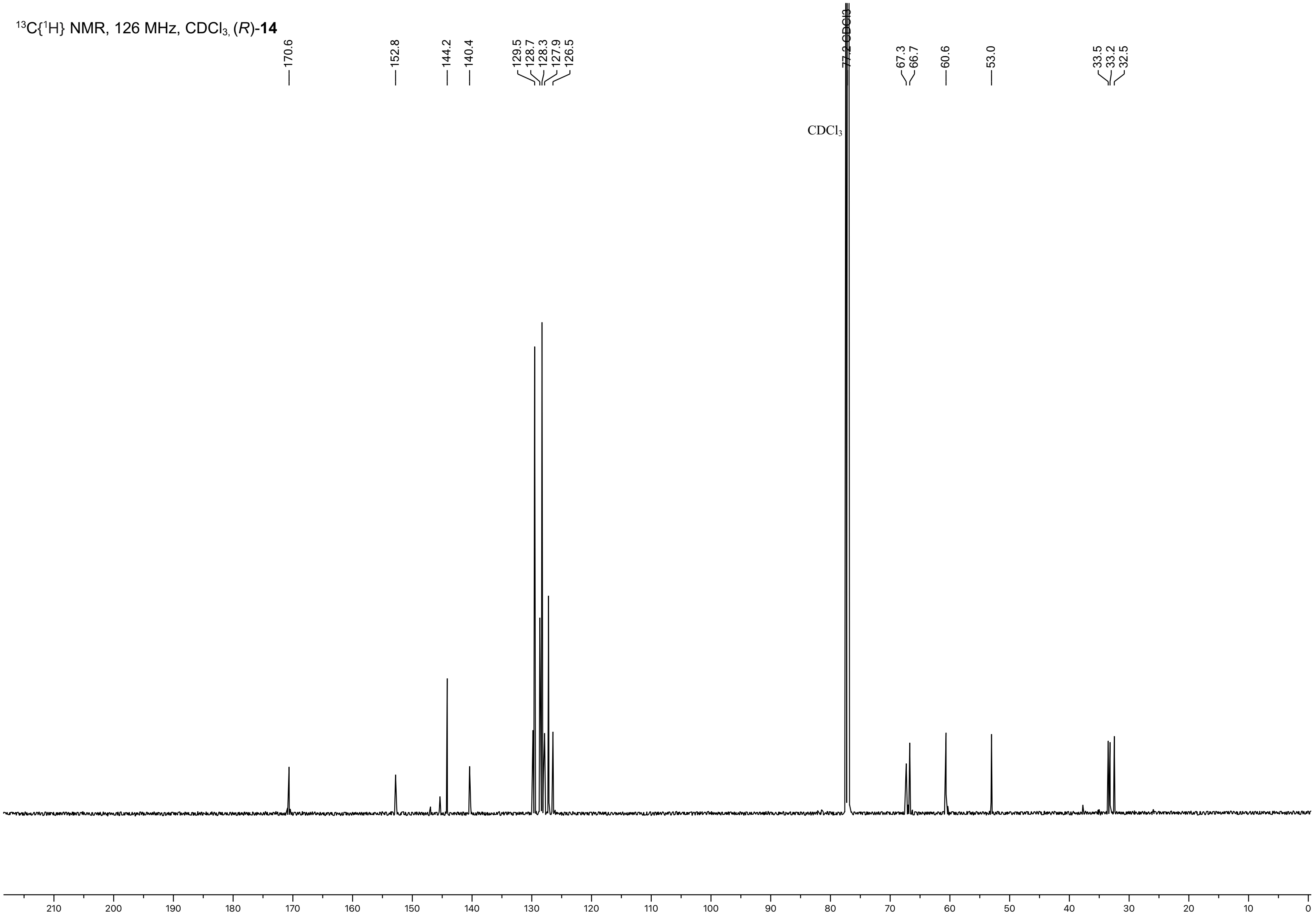

$^{13}C\{^1H\}$ NMR, 126 MHz, $CDCl_3$, (*R*)-**14**
170.6
152.8
144.2
140.4
129.5
128.7
128.3
127.9
126.5
77.2 CDCl3
CDCl3
67.3
66.7
60.6
53.0
33.5
33.2
32.5
210 200 190 180 170 160 150 140 130 120 110 100 90 80 70 60 50 40 30 20 10 0

$^1$H NMR, 500 MHz, $CDCl_3$, (*R*)-**15**

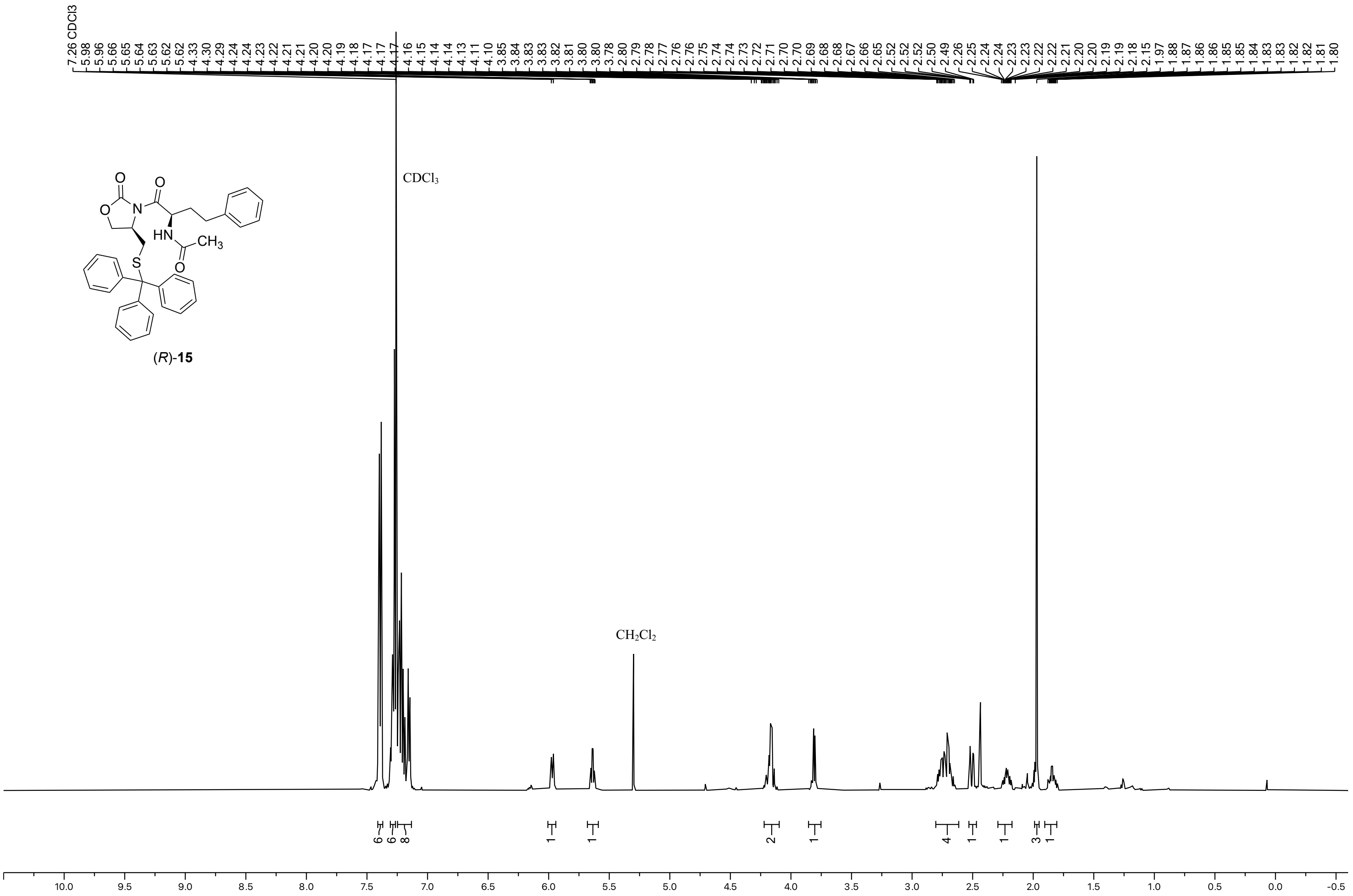

$^{13}C\{^1H\}$ NMR, 126 MHz, $CDCl_3$, (*R*)-**15**

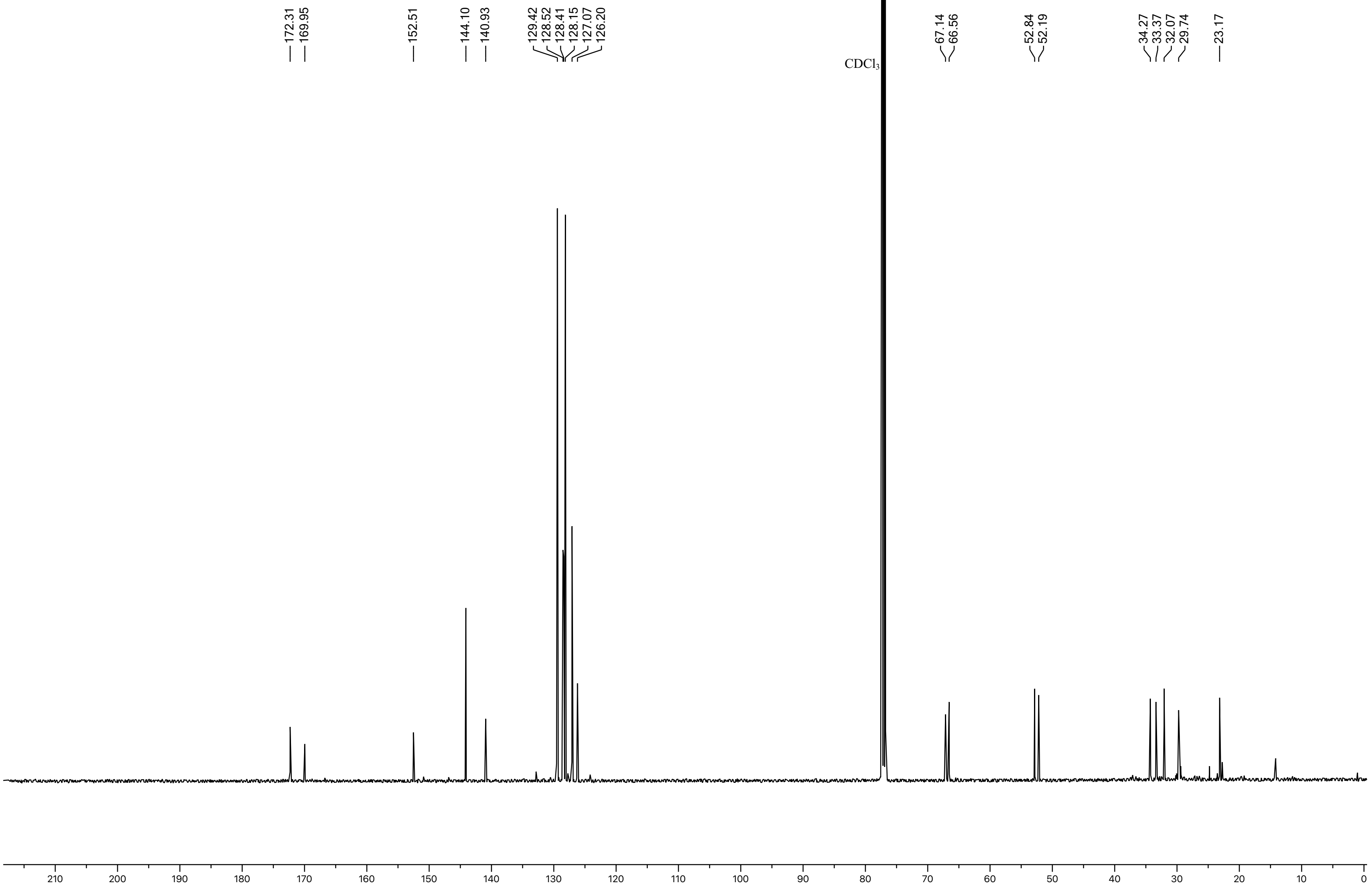

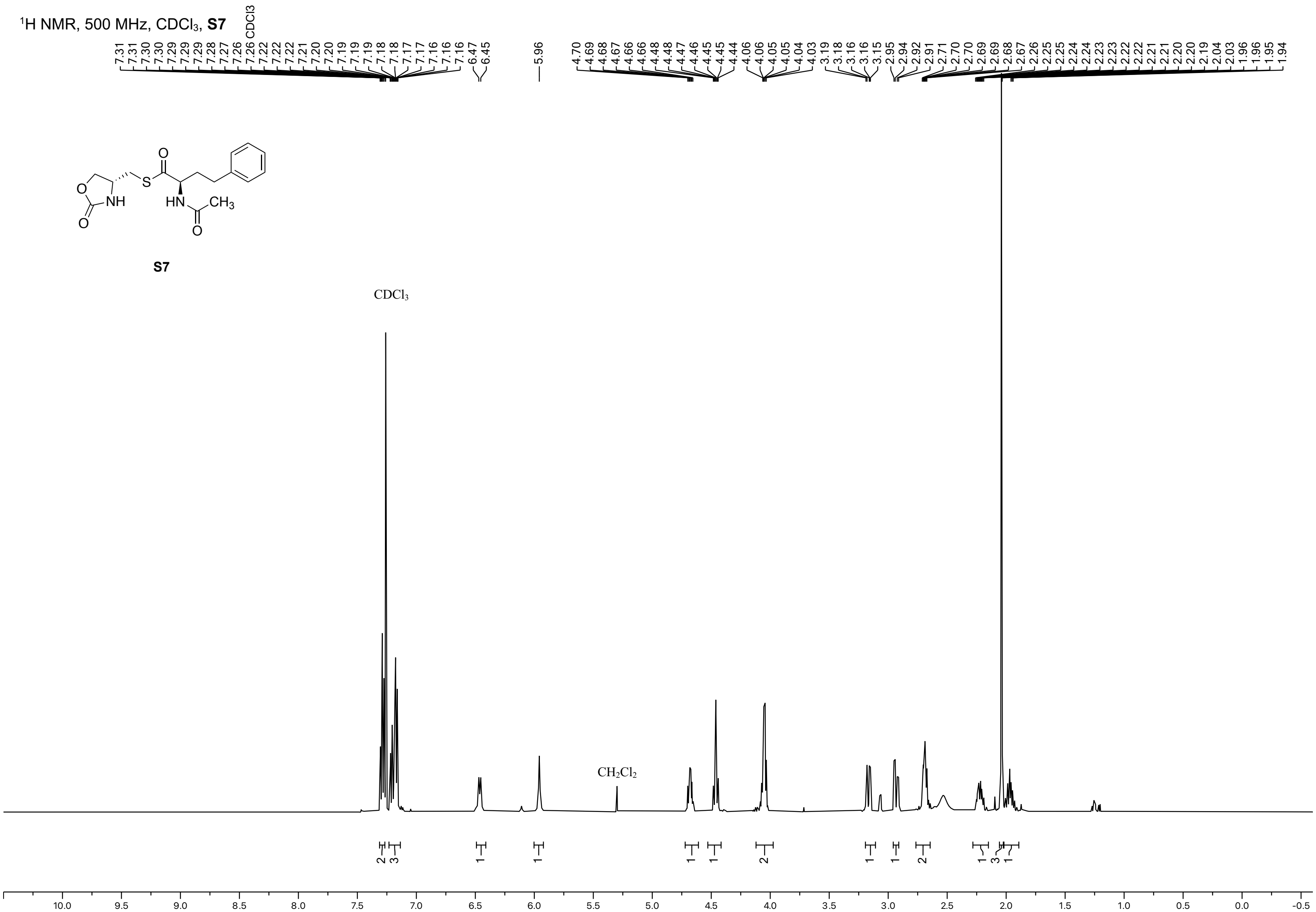
1H NMR, 500 MHz, CDCl3, S7
S7
CDCl3
CH2Cl2

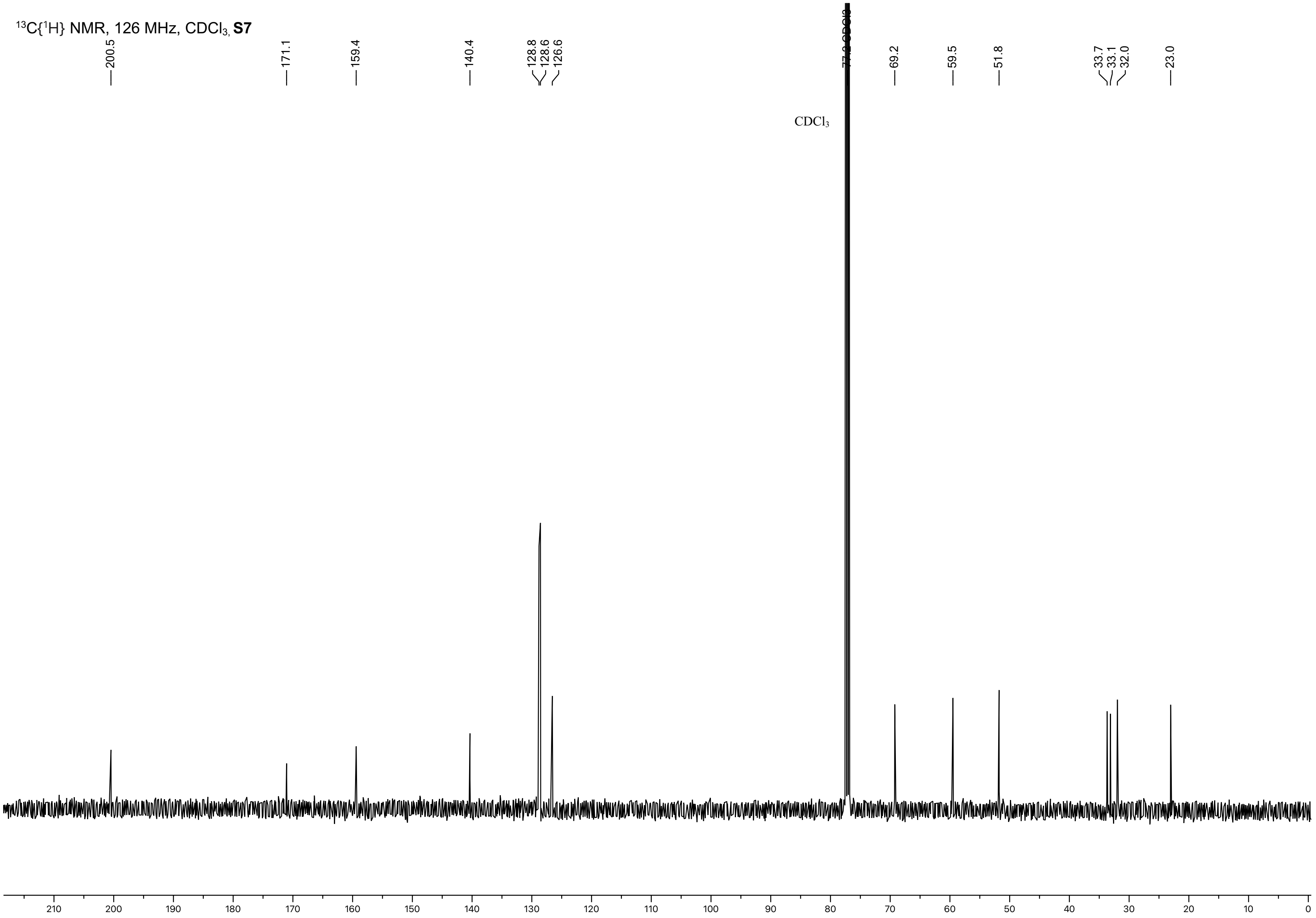
$^{13}C\{^1H\}$ NMR, 126 MHz, $CDCl_3$, **S7**
200.5
171.1
159.4
140.4
128.8
128.6
126.6
69.2
59.5
51.8
33.7
33.1
32.0
23.0
$CDCl_3$
210
200
190
180
170
160
150
140
130
120
110
100
90
80
70
60
50
40
30
20
10
0

$^1H$ NMR, 500 MHz, $CDCl_3$, **18**

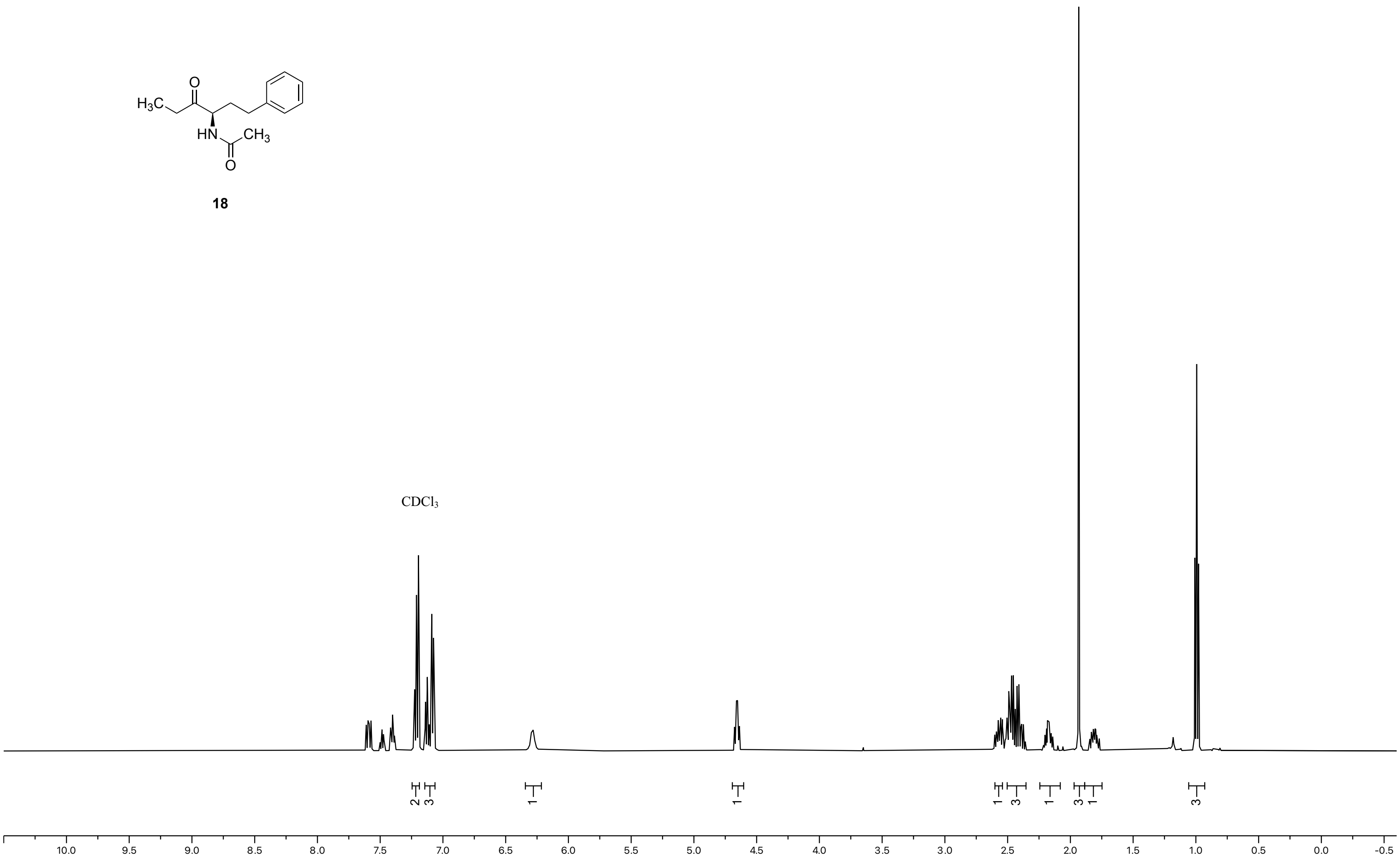

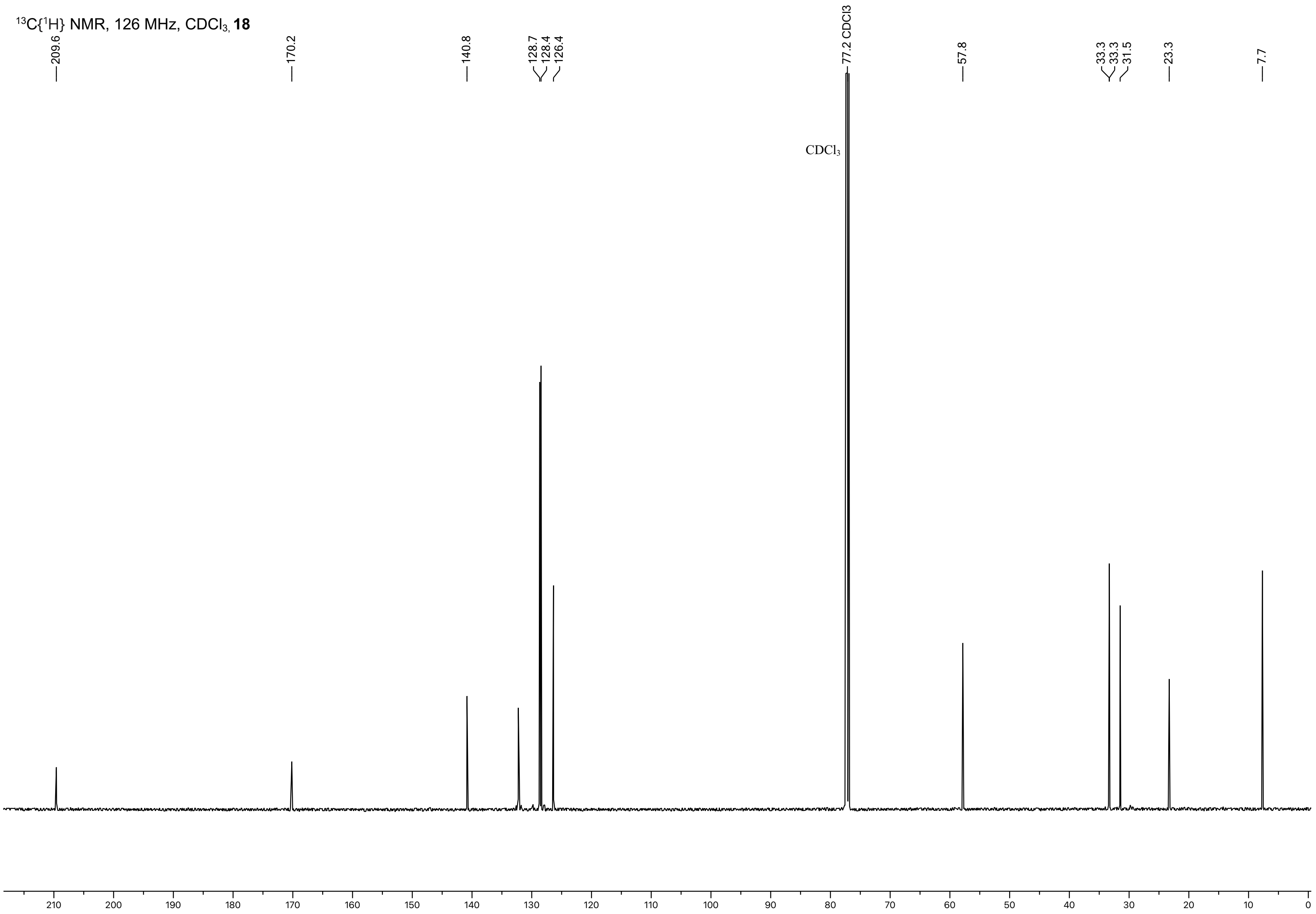

$^{13}C\{^{1}H\}$ NMR, 126 MHz, $CDCl_3$, **18**
209.6
170.2
140.8
128.7
128.4
126.4
77.2 CDCl3
57.8
33.3
33.3
31.5
23.3
7.7
CDCl3
210
200
190
180
170
160
150
140
130
120
110
100
90
80
70
60
50
40
30
20
10
0

[1]H NMR, 500 MHz, $CDCl_3$, **19**

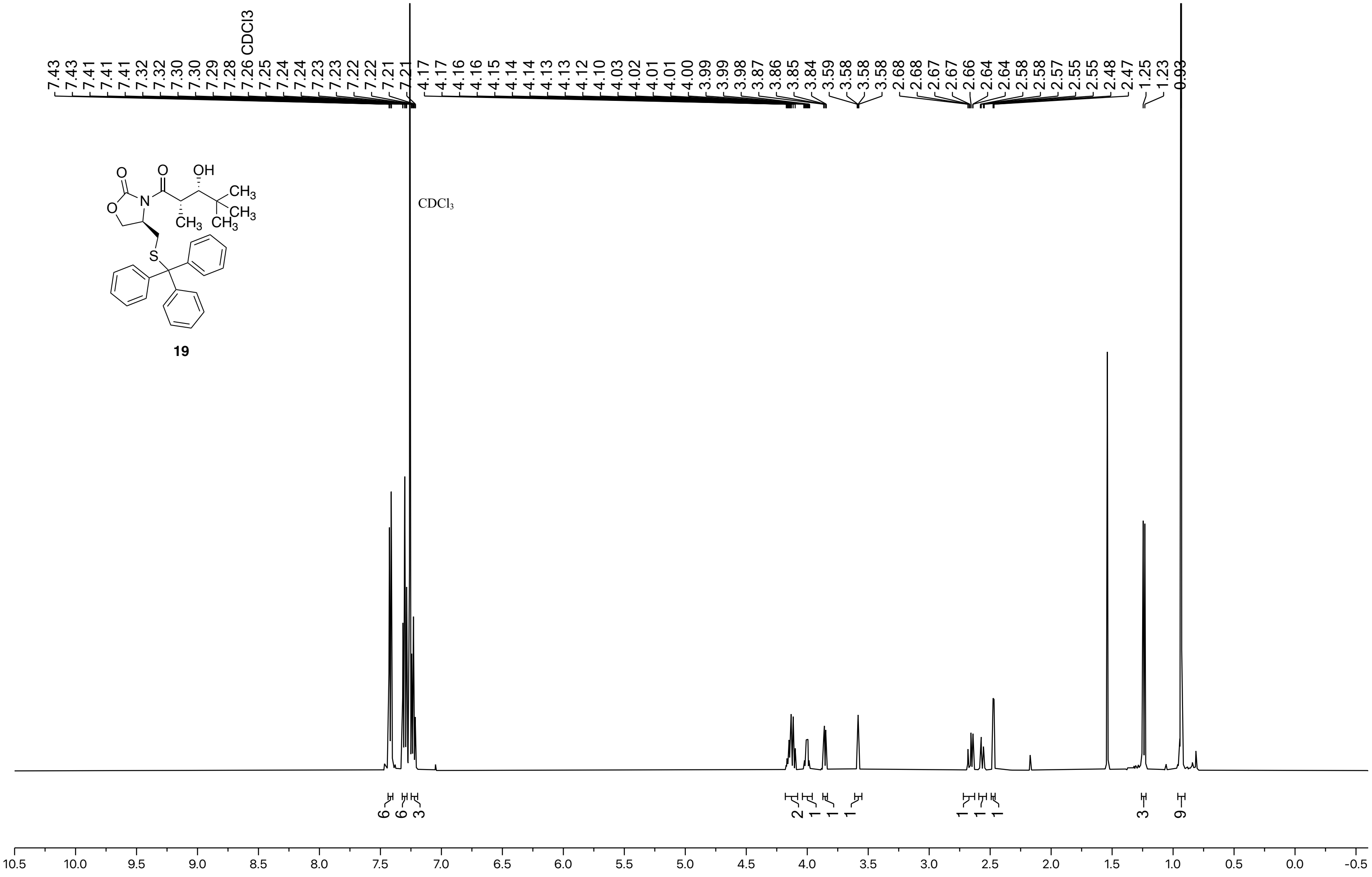

$^{13}C\{^1H\}$ NMR, 126 MHz, $CDCl_3$, **19**

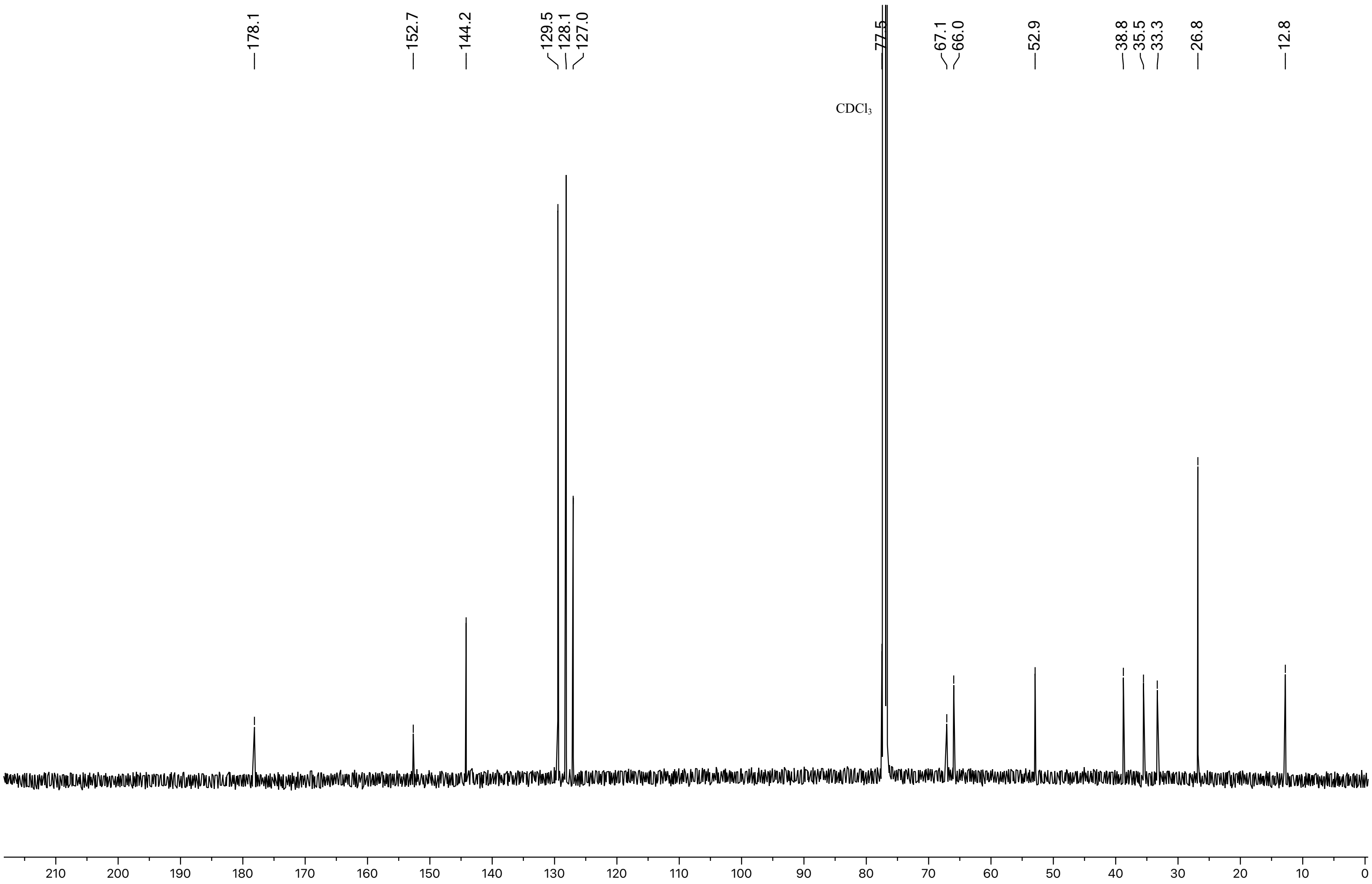

$^{1}H$ NMR, 500 MHz, $CDCl_3$, **20**

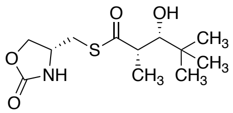


**20**

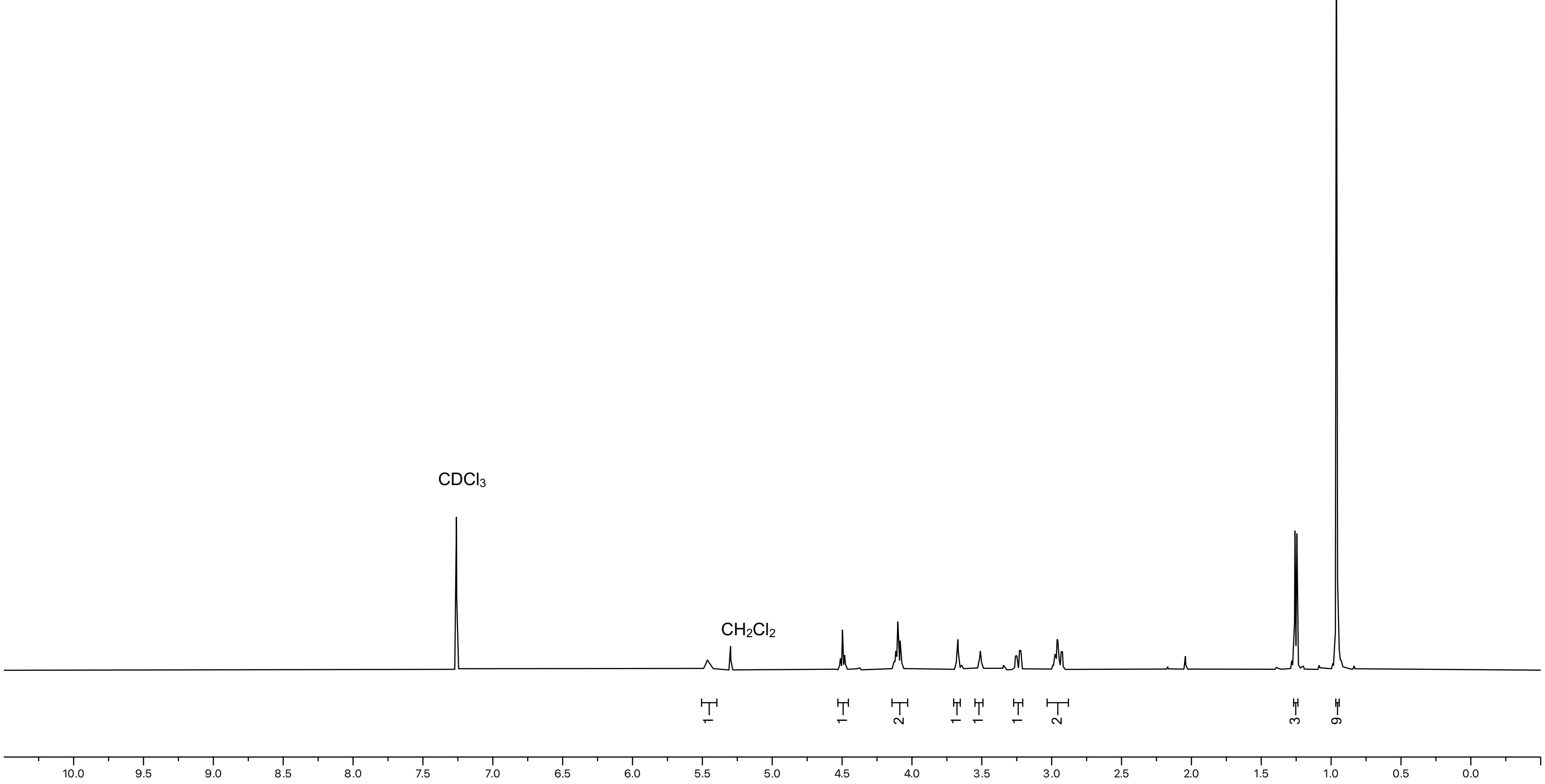

$^{13}C\{^{1}H\}$ NMR, 126 MHz, $CDCl_3$, **20**
203.5
159.3
78.3
77.4
77.4
77.2 CDCl3
69.2
51.9
50.5
36.2
33.3
26.8
12.6
CDCl3
210 200 190 180 170 160 150 140 130 120 110 100 90 80 70 60 50 40 30 20 10 0


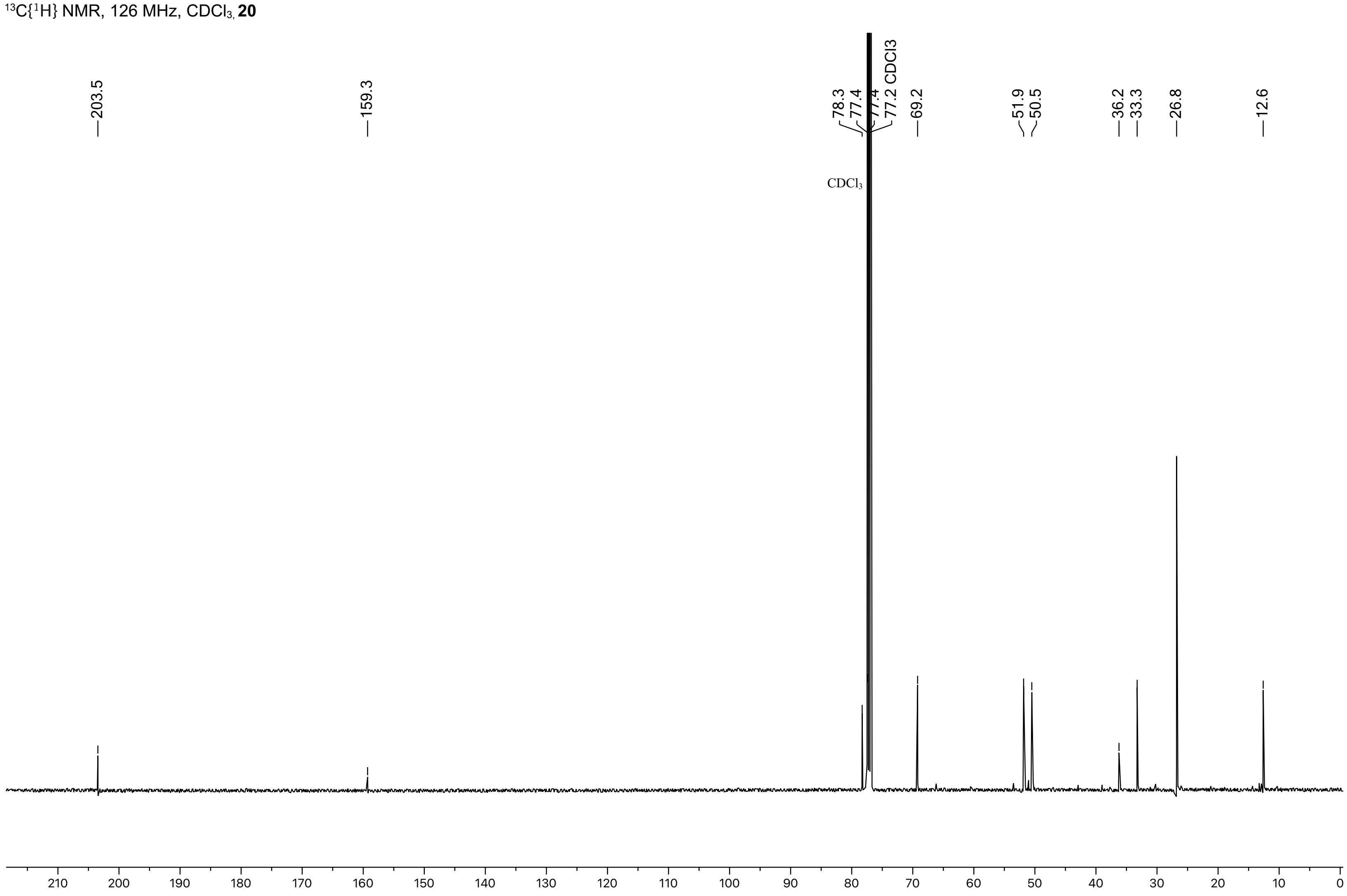

$^1$H NMR, 500 MHz, $CDCl_3$, **21**

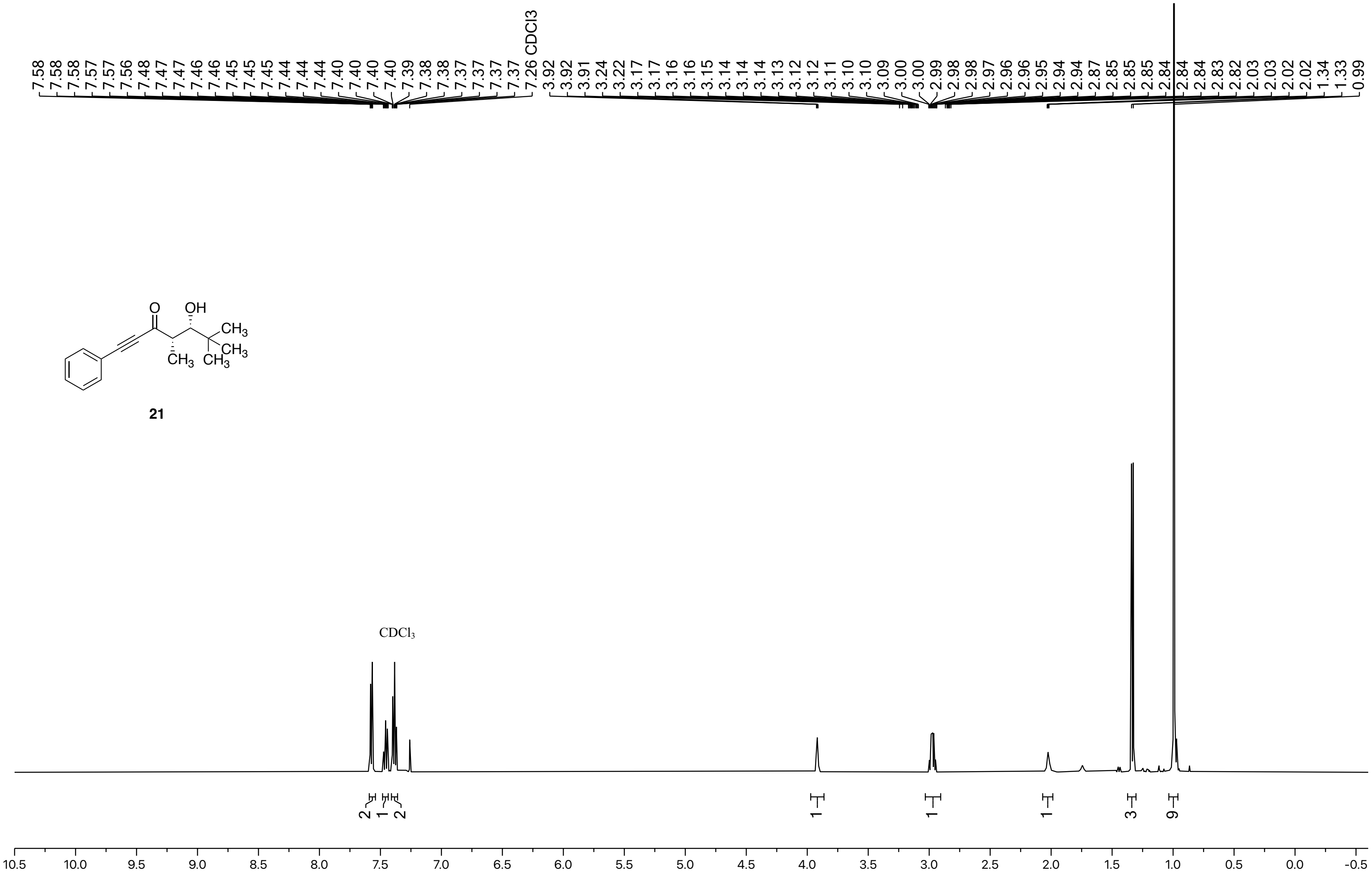

$^{13}C\{^1H\}$ NMR, 126 MHz, $CDCl_3$, **21**

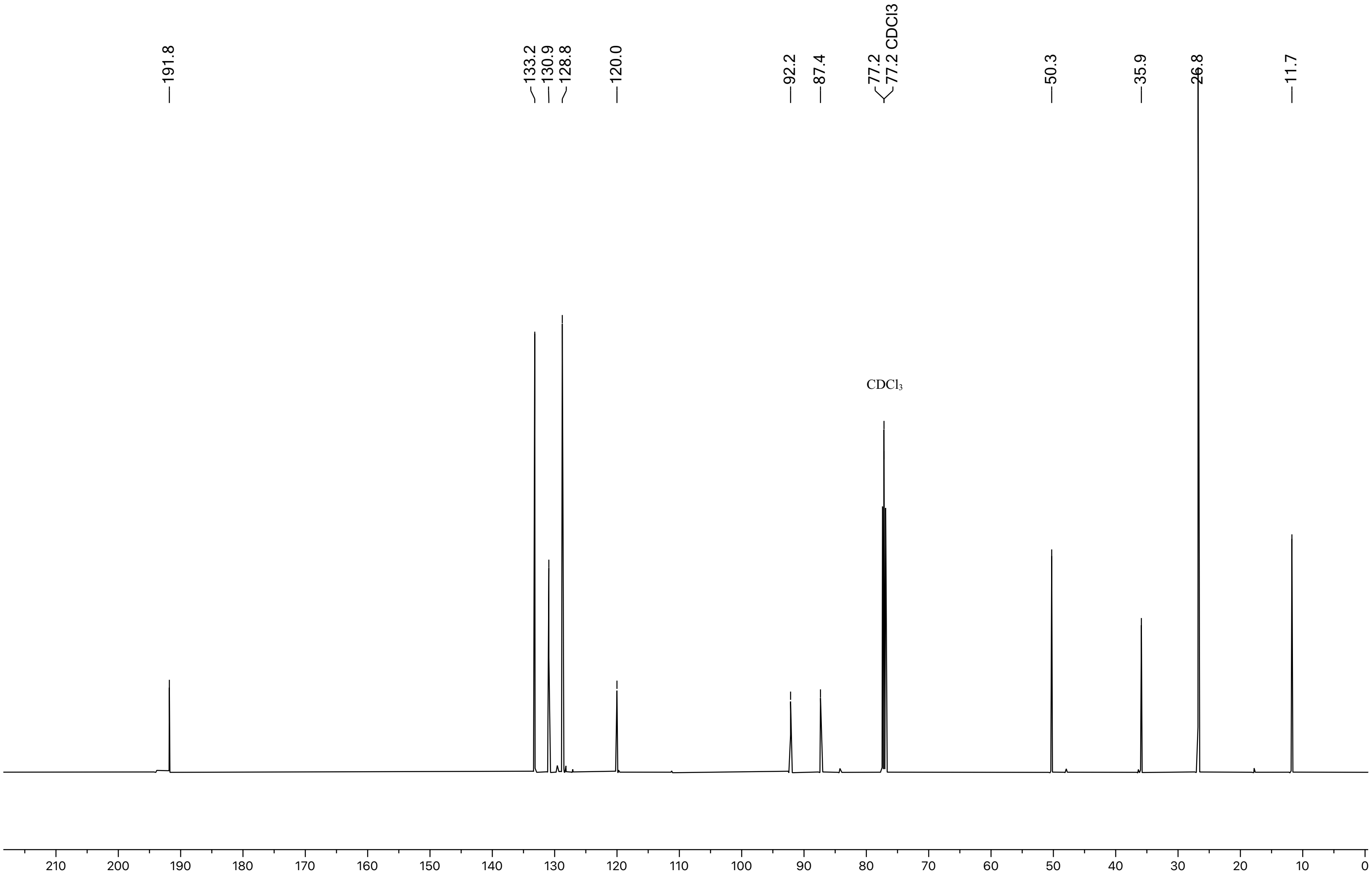

[1]H NMR, 500 MHz, $CDCl_3$, **23**

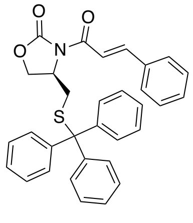


**23**

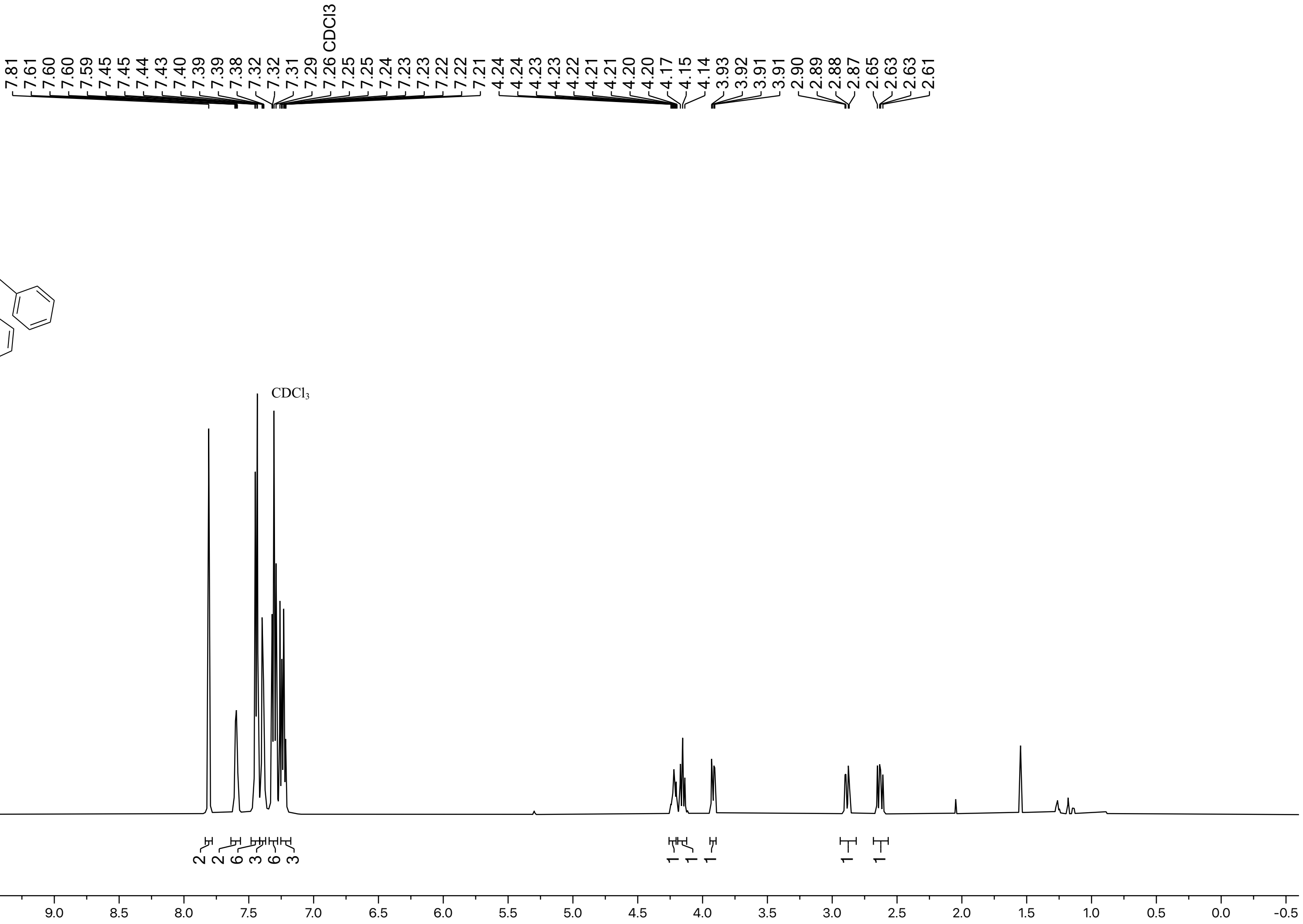

$^{13}C\{^{1}H\}$ NMR, 126 MHz, $CDCl_3$, **23**

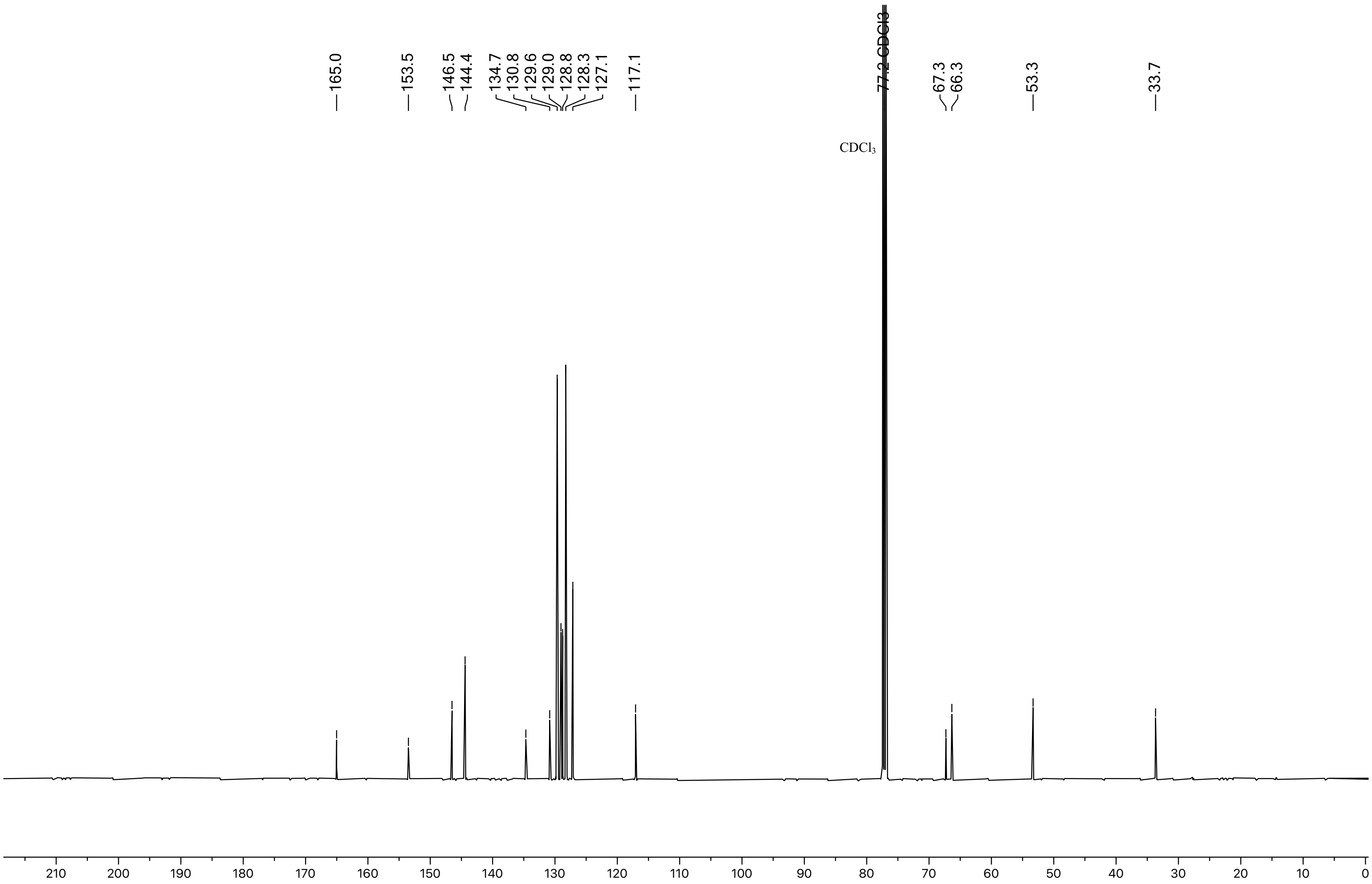

$^{1}H$ NMR, 500 MHz, $CDCl_3$, **24**

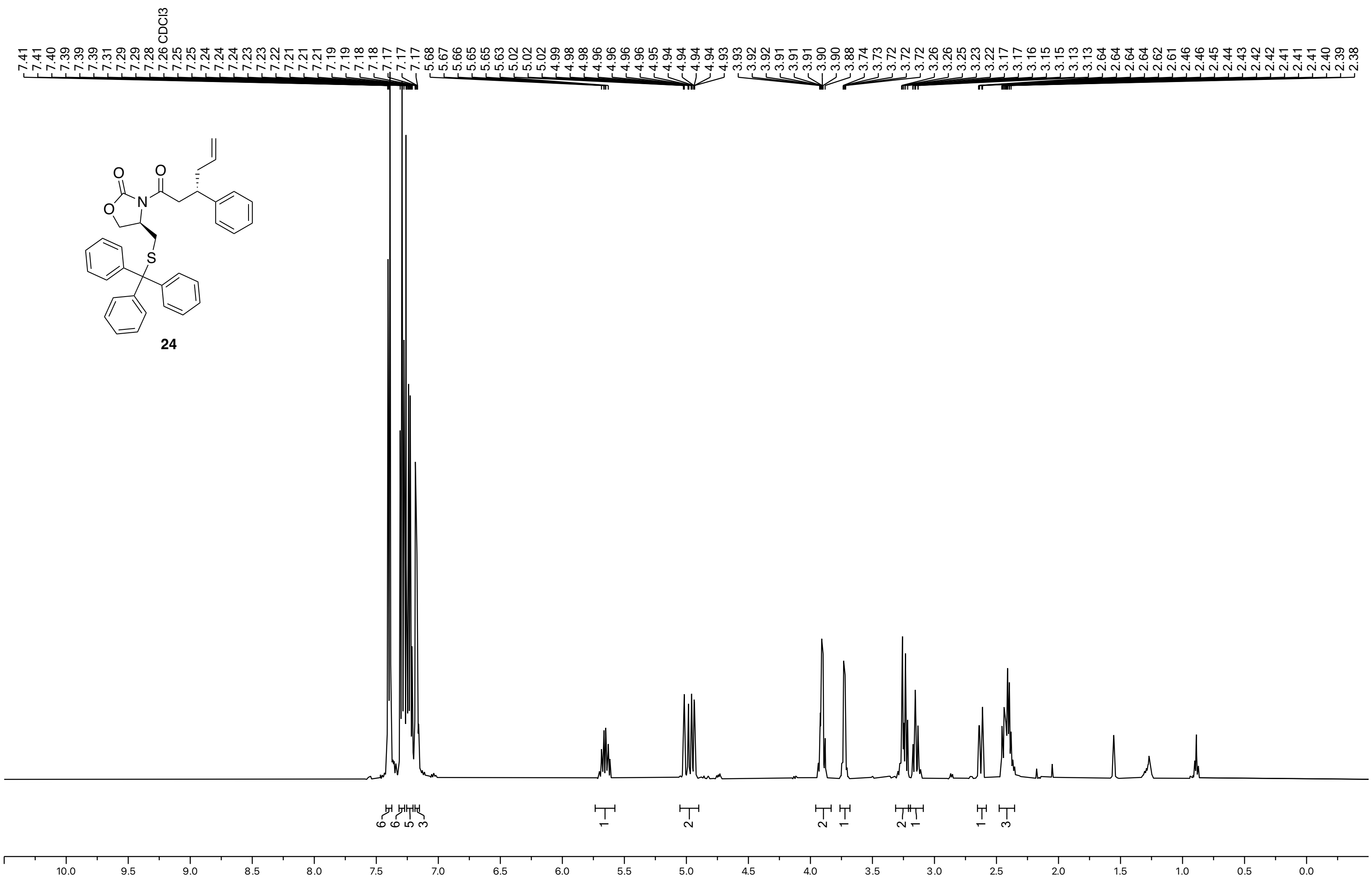

$^{13}C\{^{1}H\}$ NMR, 126 MHz, $CDCl_3$, **24**

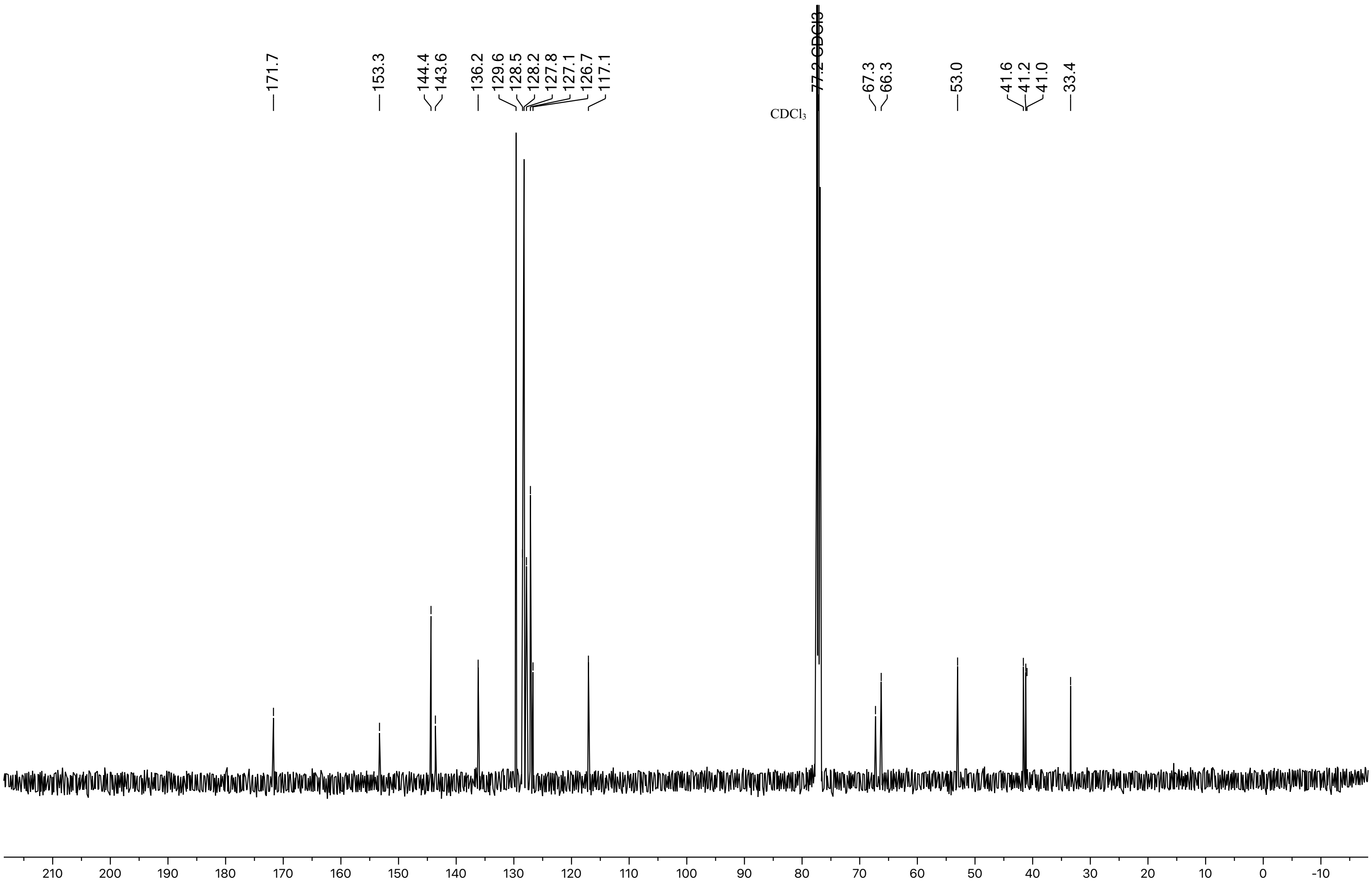

$^1H$ NMR, 500 MHz, $CDCl_3$, **25**

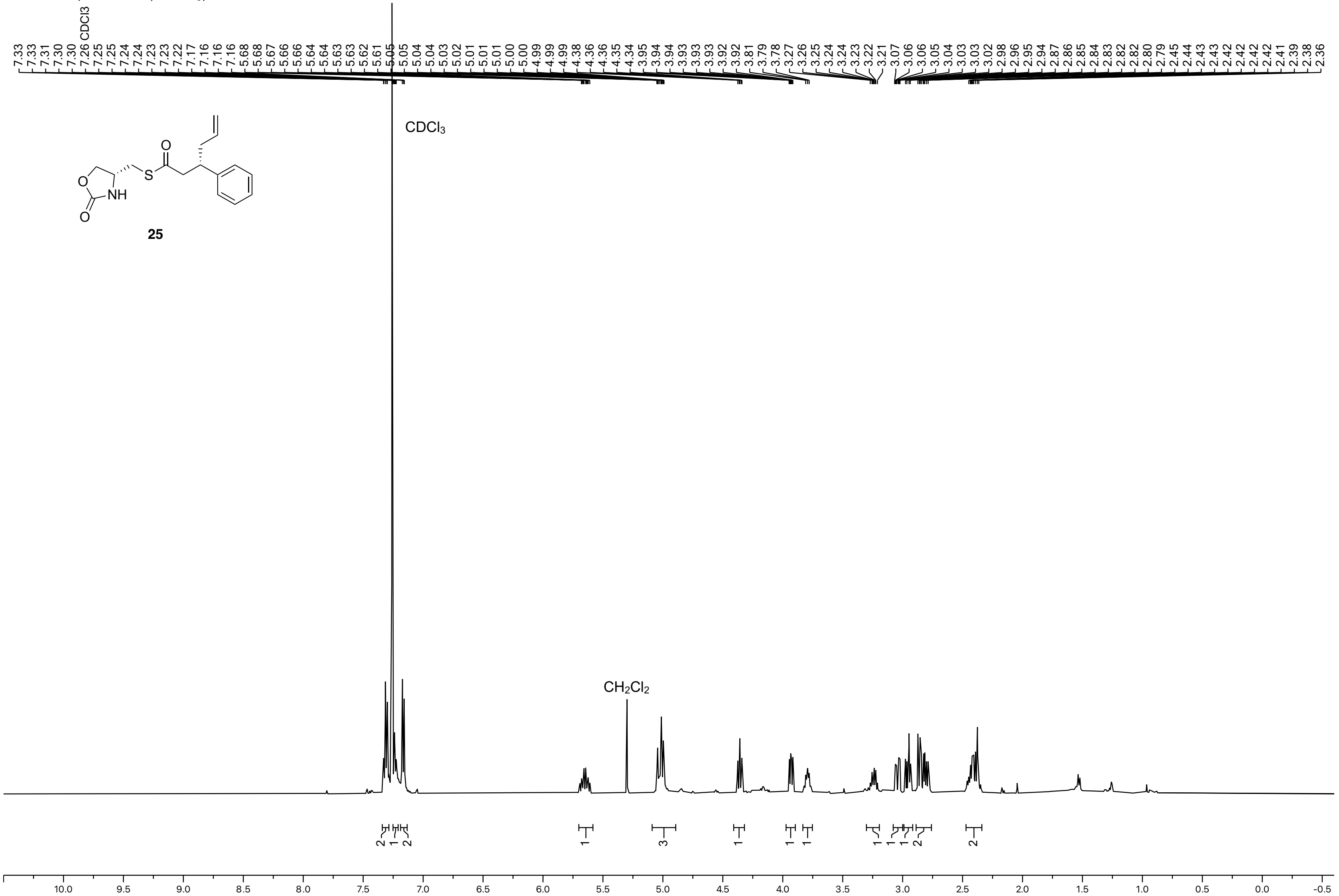

$^{13}C\{^{1}H\}$ NMR, 126 MHz, $CDCl_3$, **25**

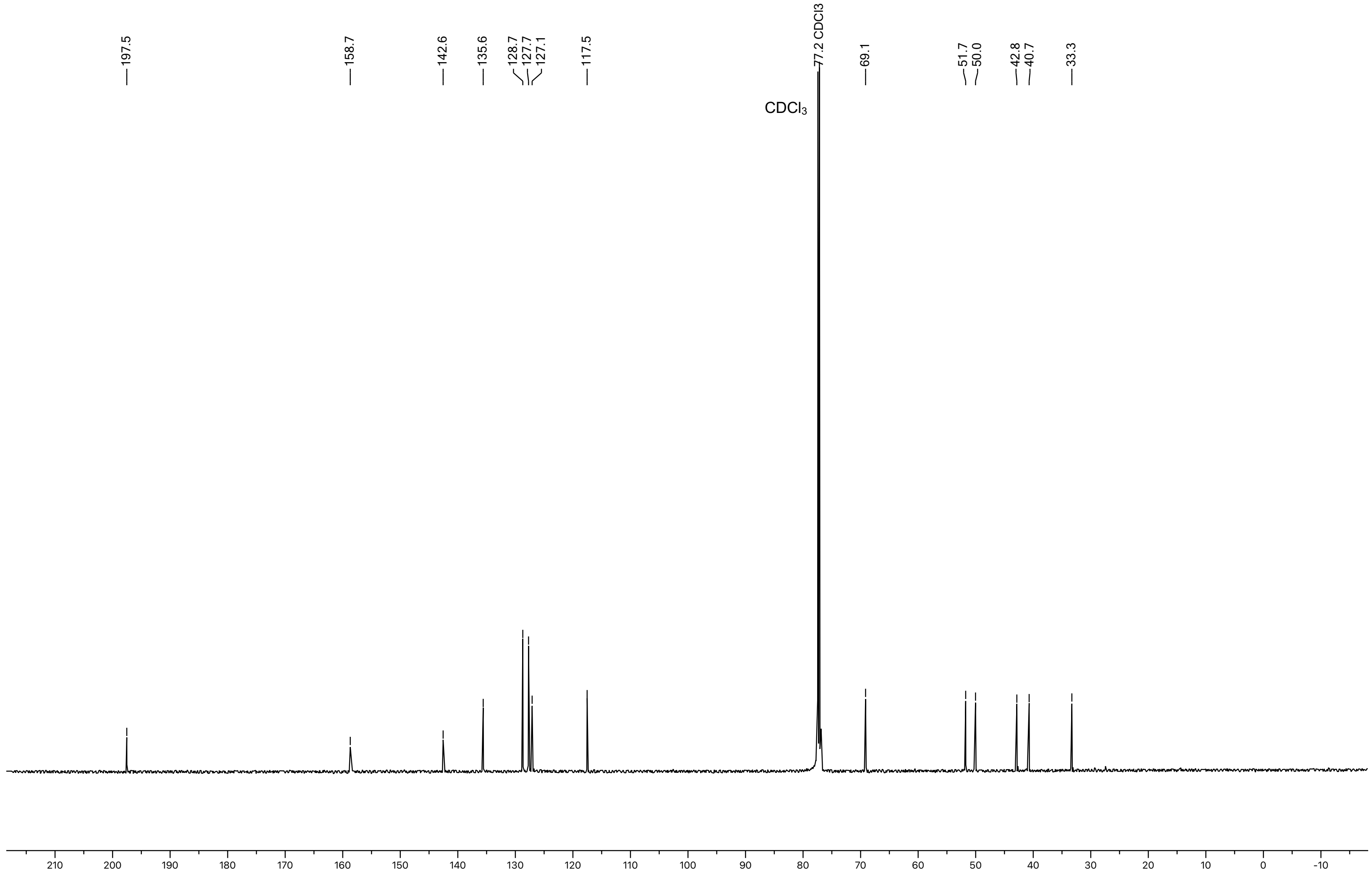

$^1H$ NMR, 500 MHz, $CDCl_3$, **27**

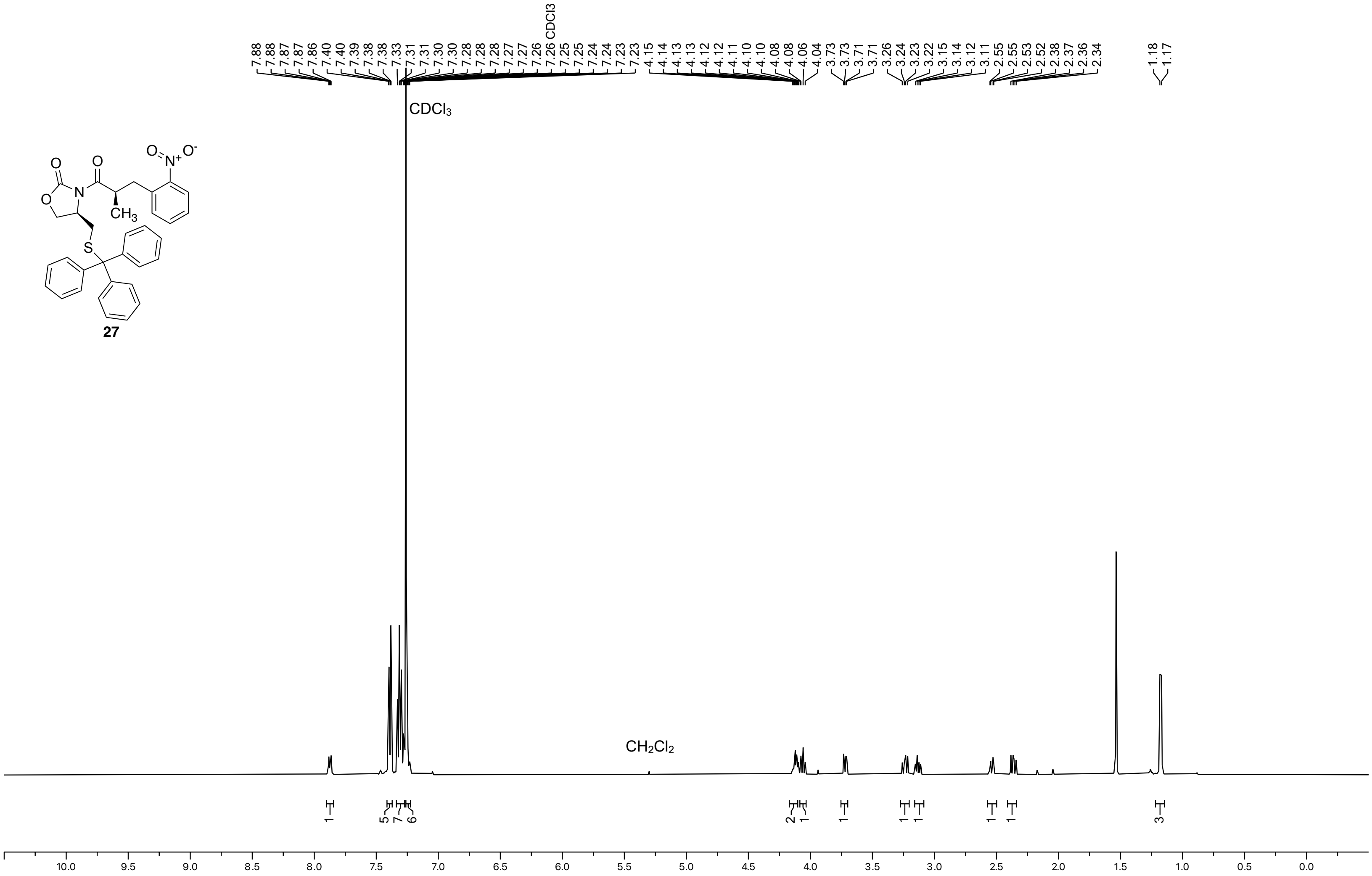

$^{13}C\{^{1}H\}$ NMR, 126 MHz, $CDCl_3$, **27**

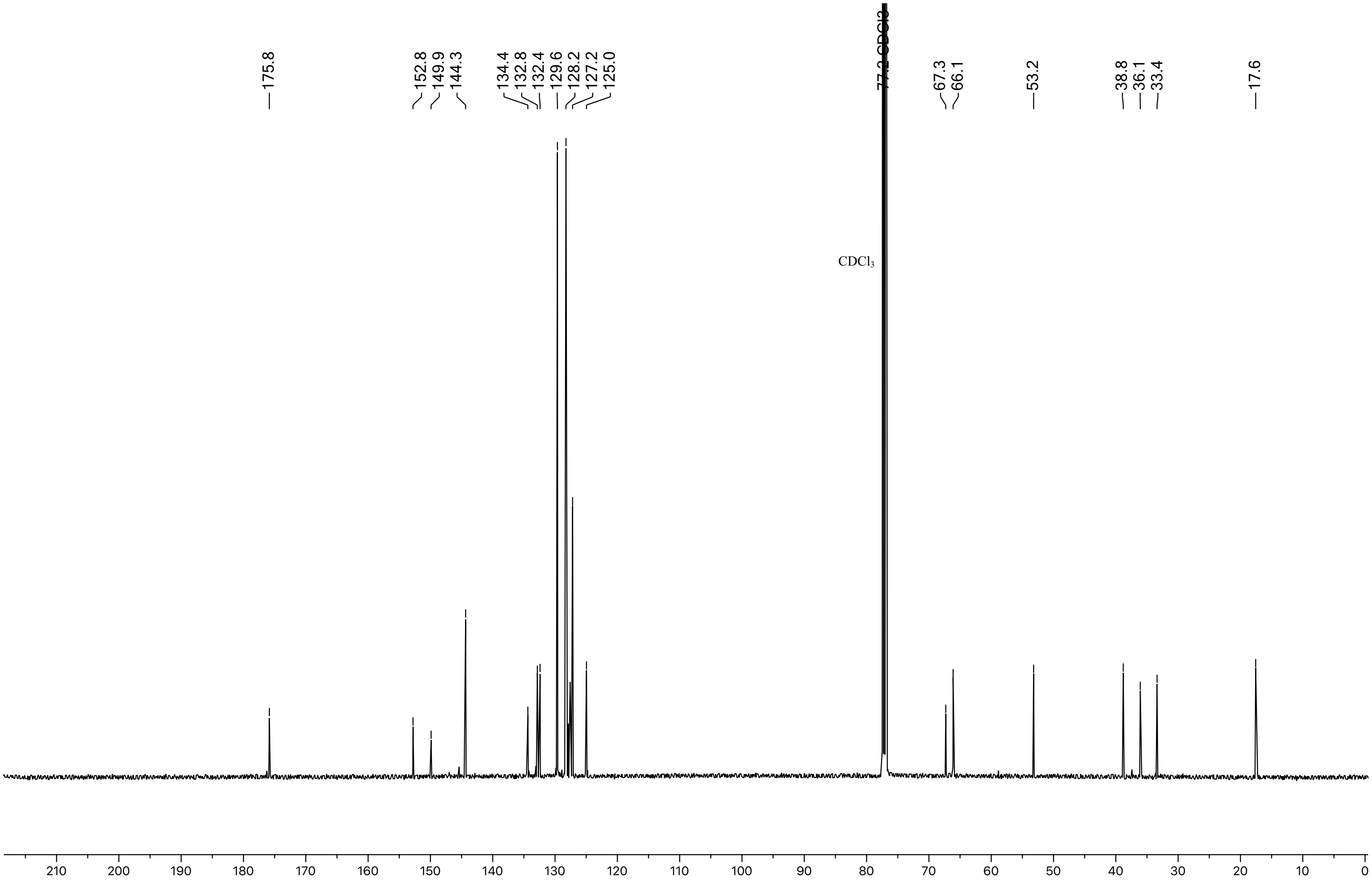

$^{1}H$ NMR, 500 MHz, $CDCl_3$, **28**

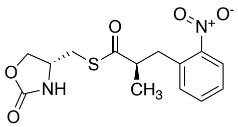

**28**

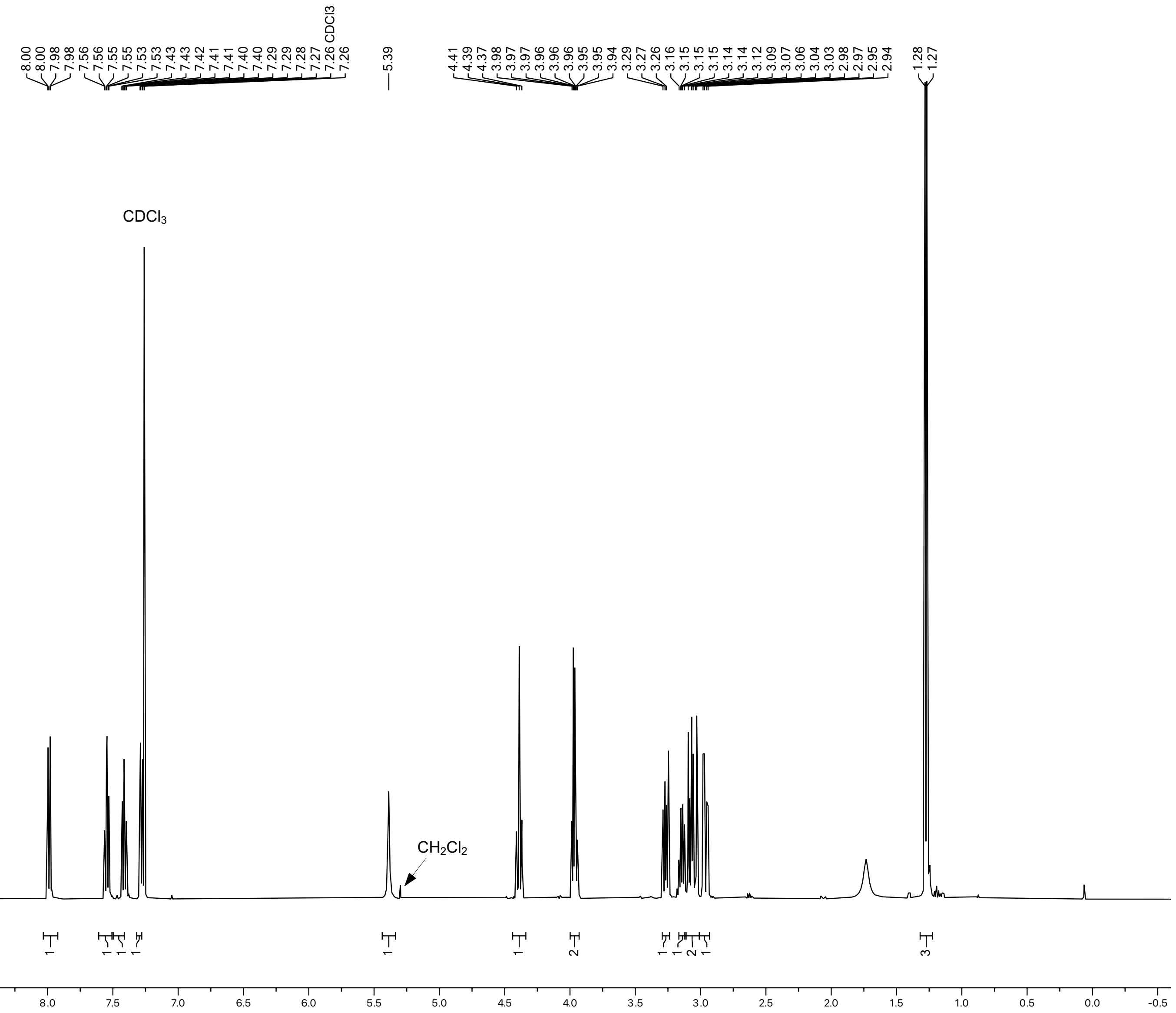

$^{13}C\{^{1}H\}$ NMR, 126 MHz, $CDCl_3$, **28**

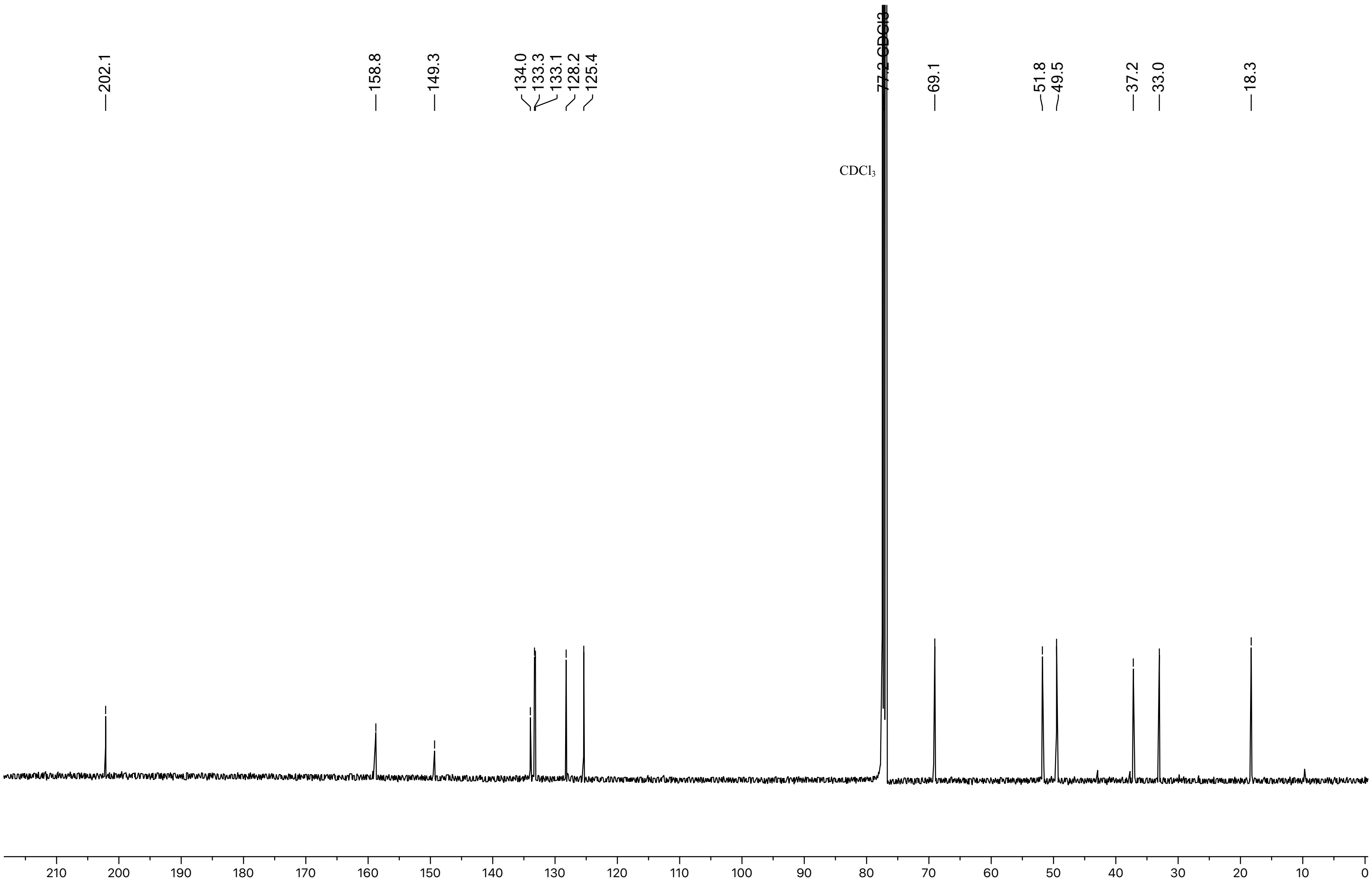

$^1$H NMR, 500 MHz, $CDCl_3$, **29**

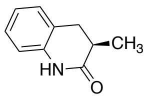


**29**

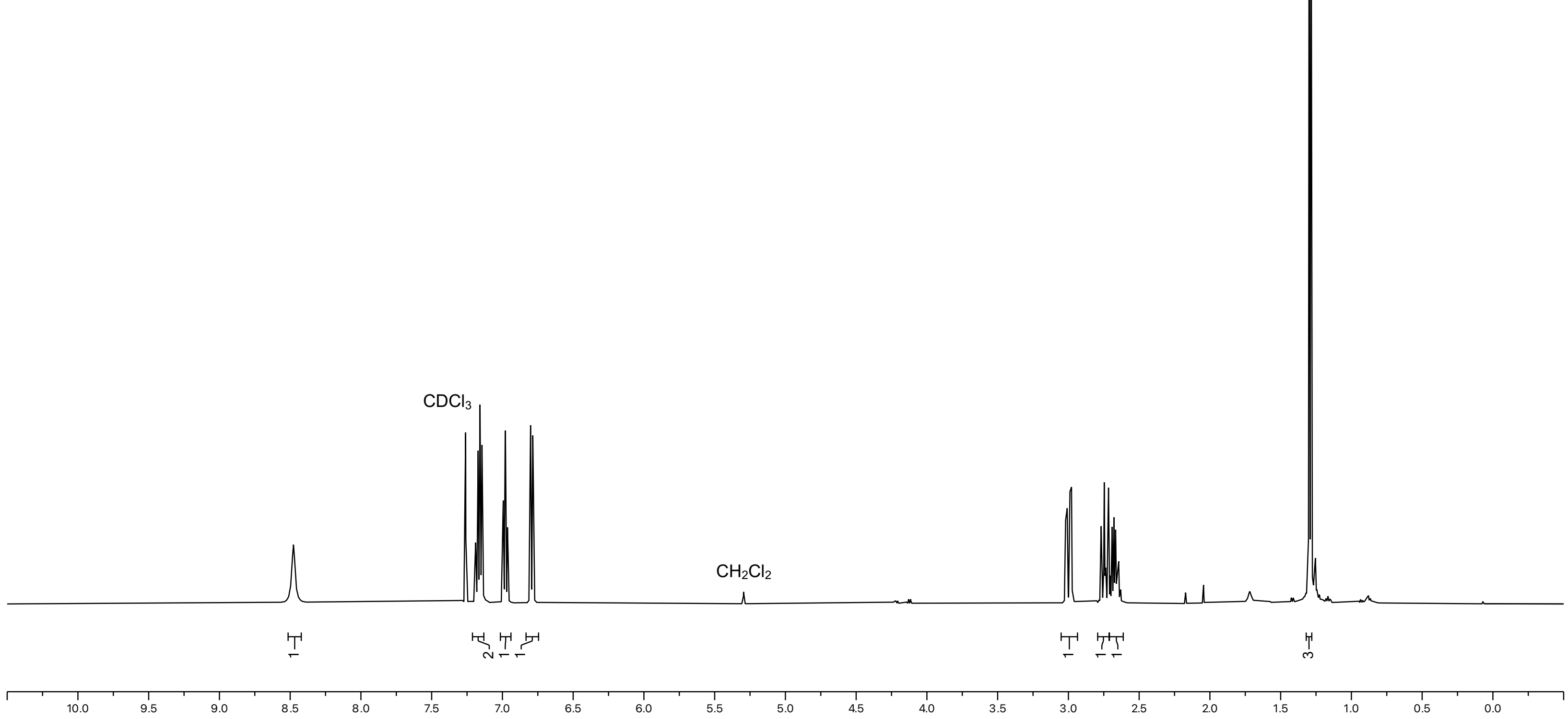

$^{13}C\{^{1}H\}$ NMR, 126 MHz, $CDCl_3$, **29**

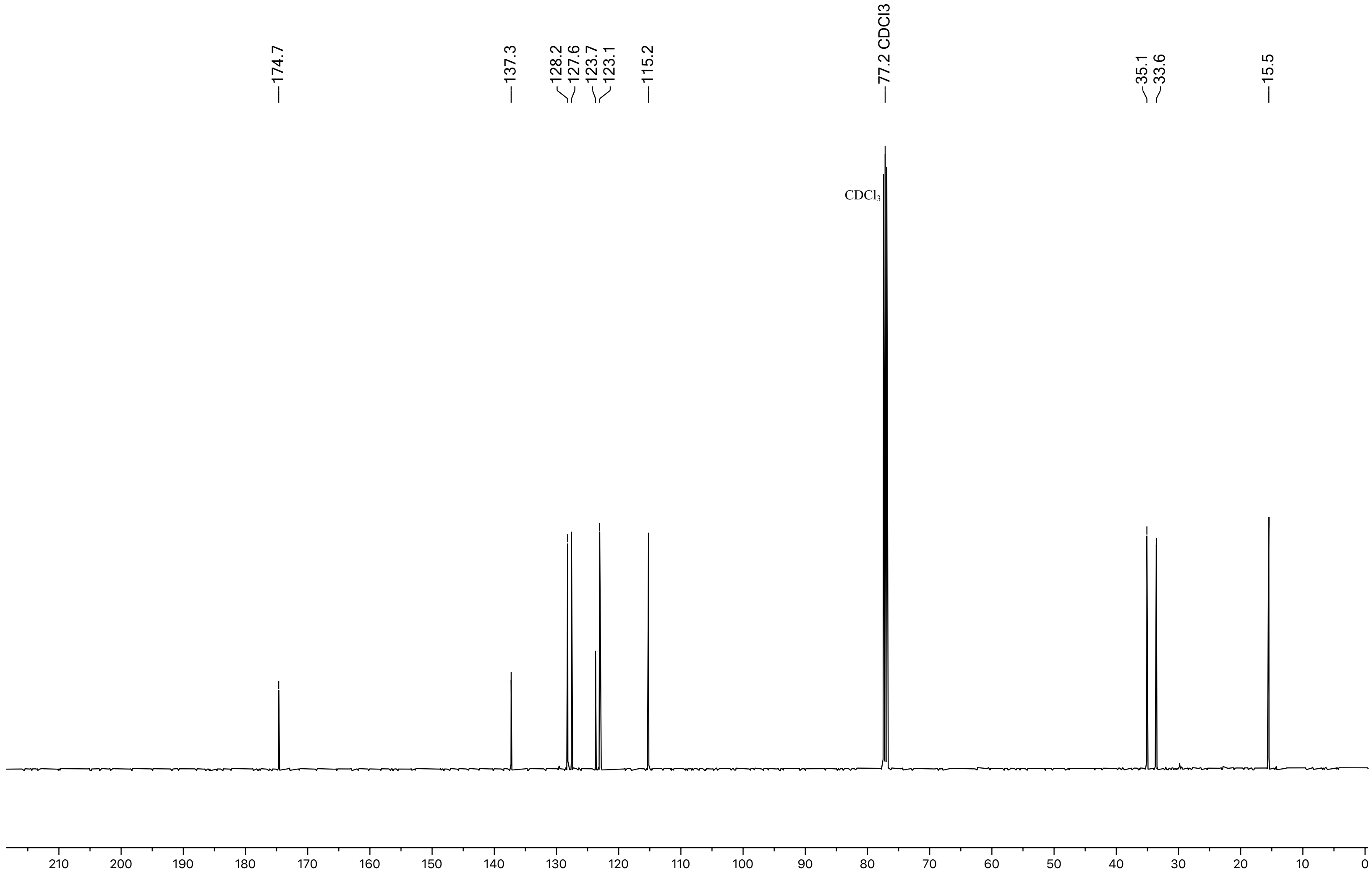

$^{1}H$ NMR, 500 MHz, $CDCl_3$, **30**

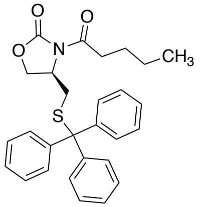


**30**

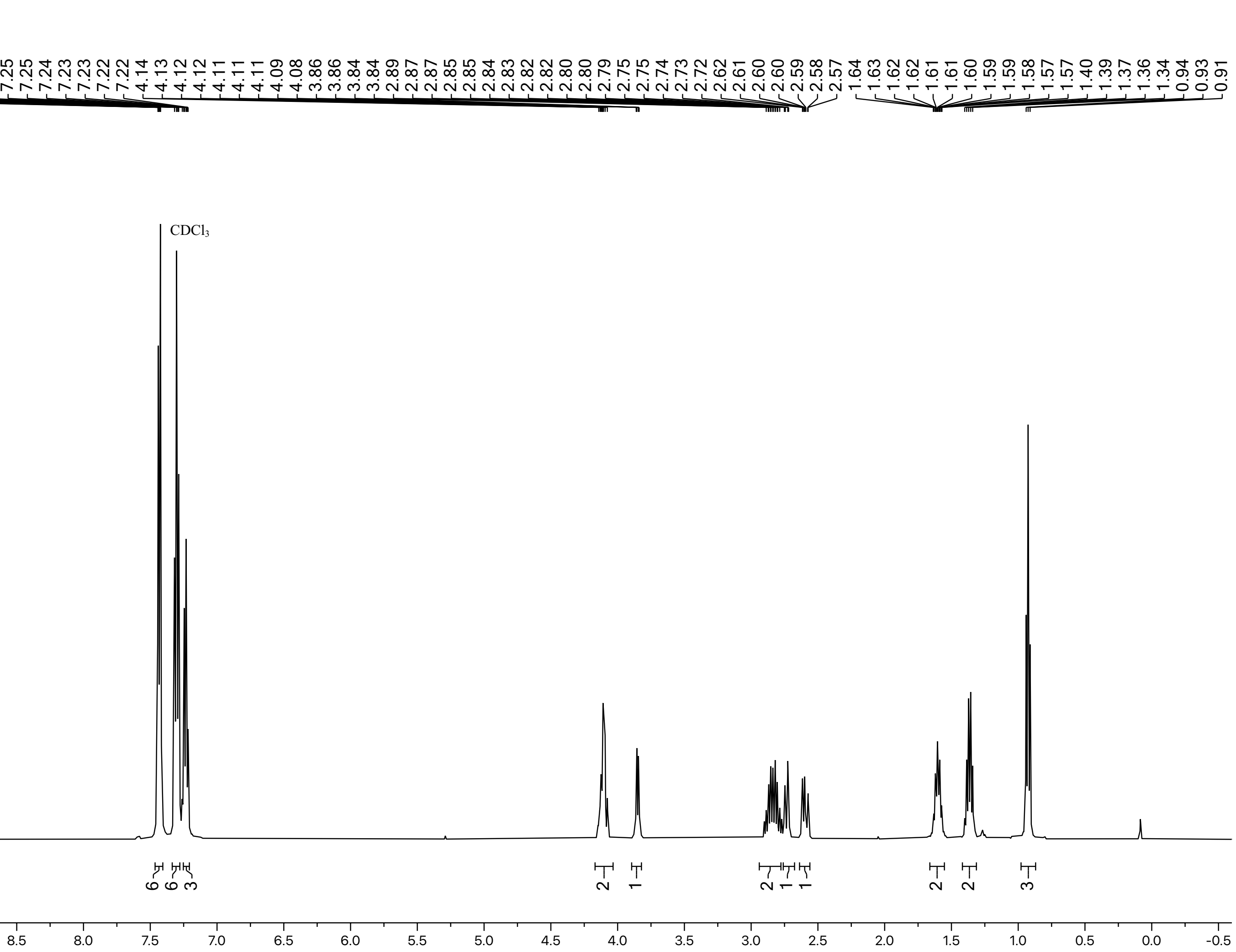

$^{13}C\{^{1}H\}$ NMR, 126 MHz, $CDCl_3$, **30**

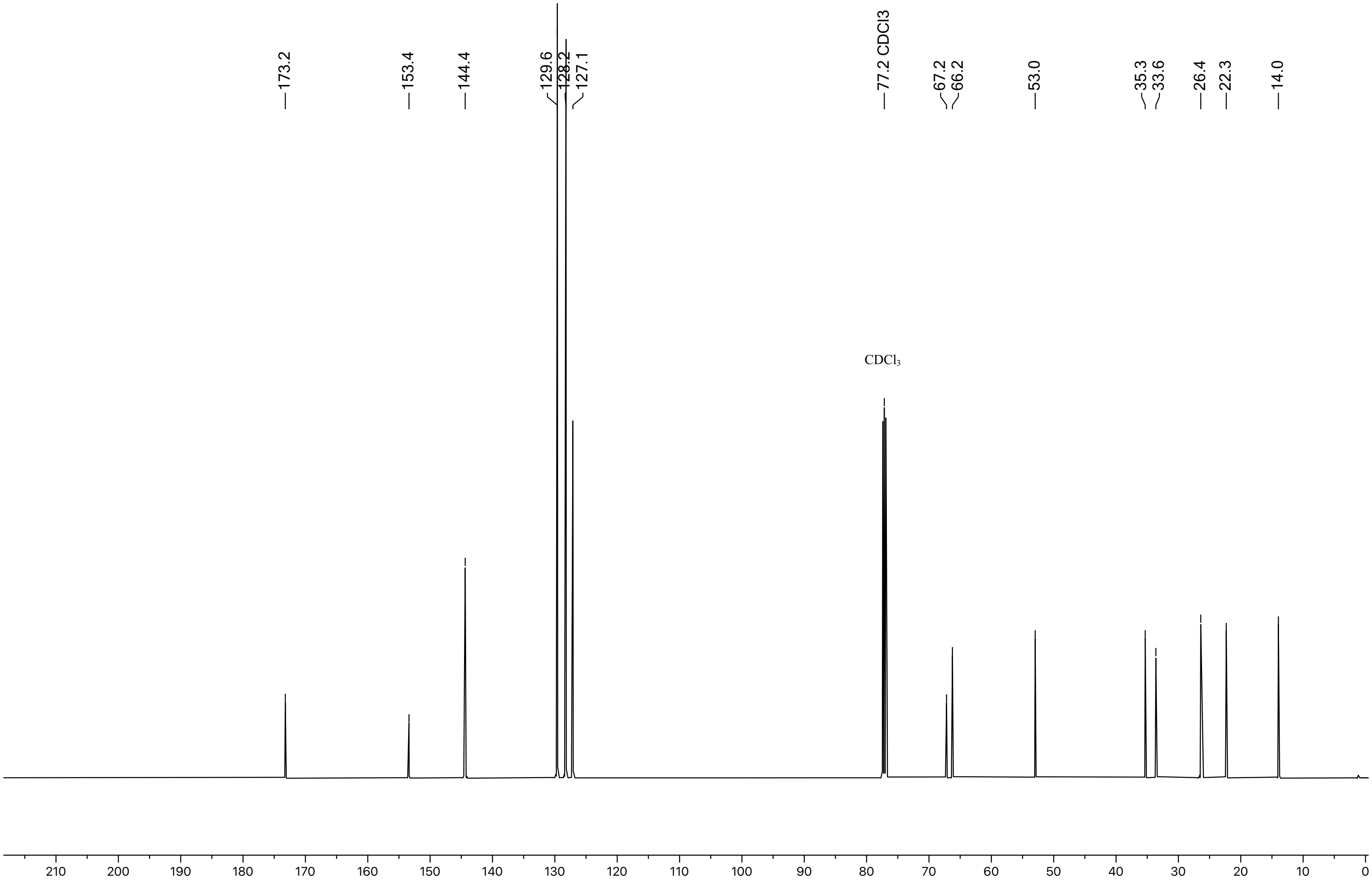

$^{1}$H NMR, 500 MHz, $CDCl_3$, **S13**

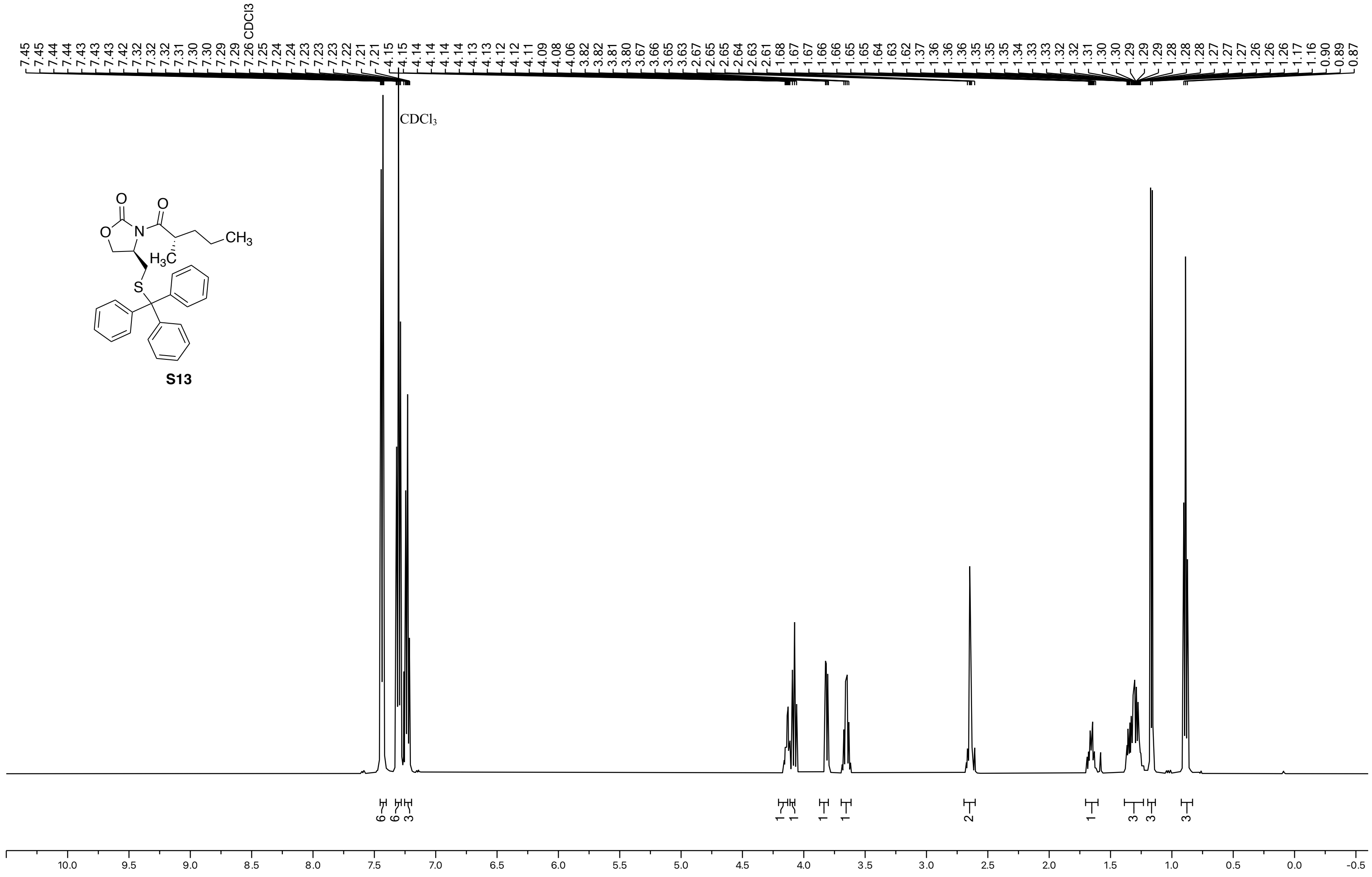

$^{13}C\{^1H\}$ NMR, 126 MHz, $CDCl_3$, **S13**

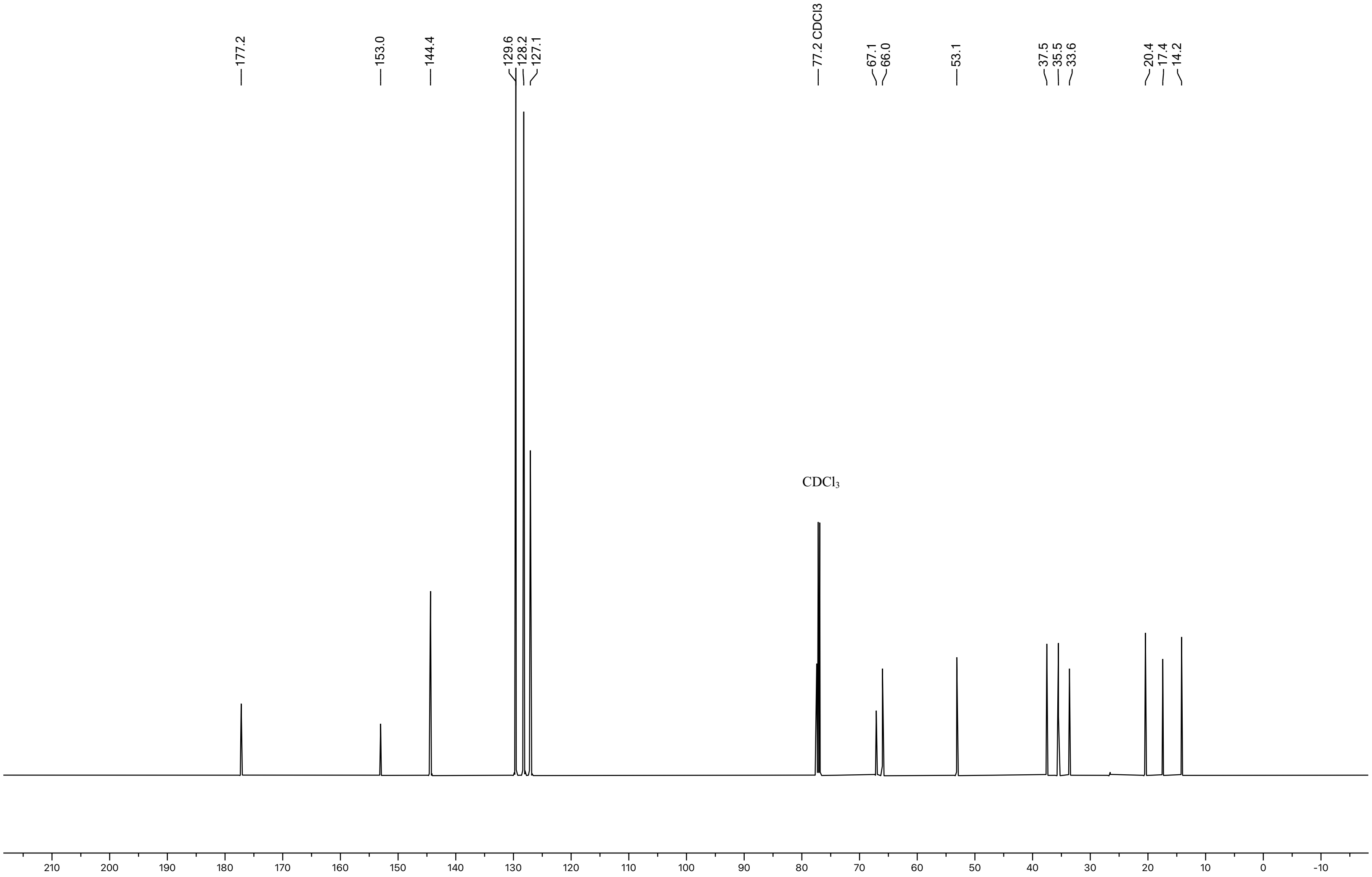

[1]H NMR, 500 MHz, $CDCl_3$, **31**

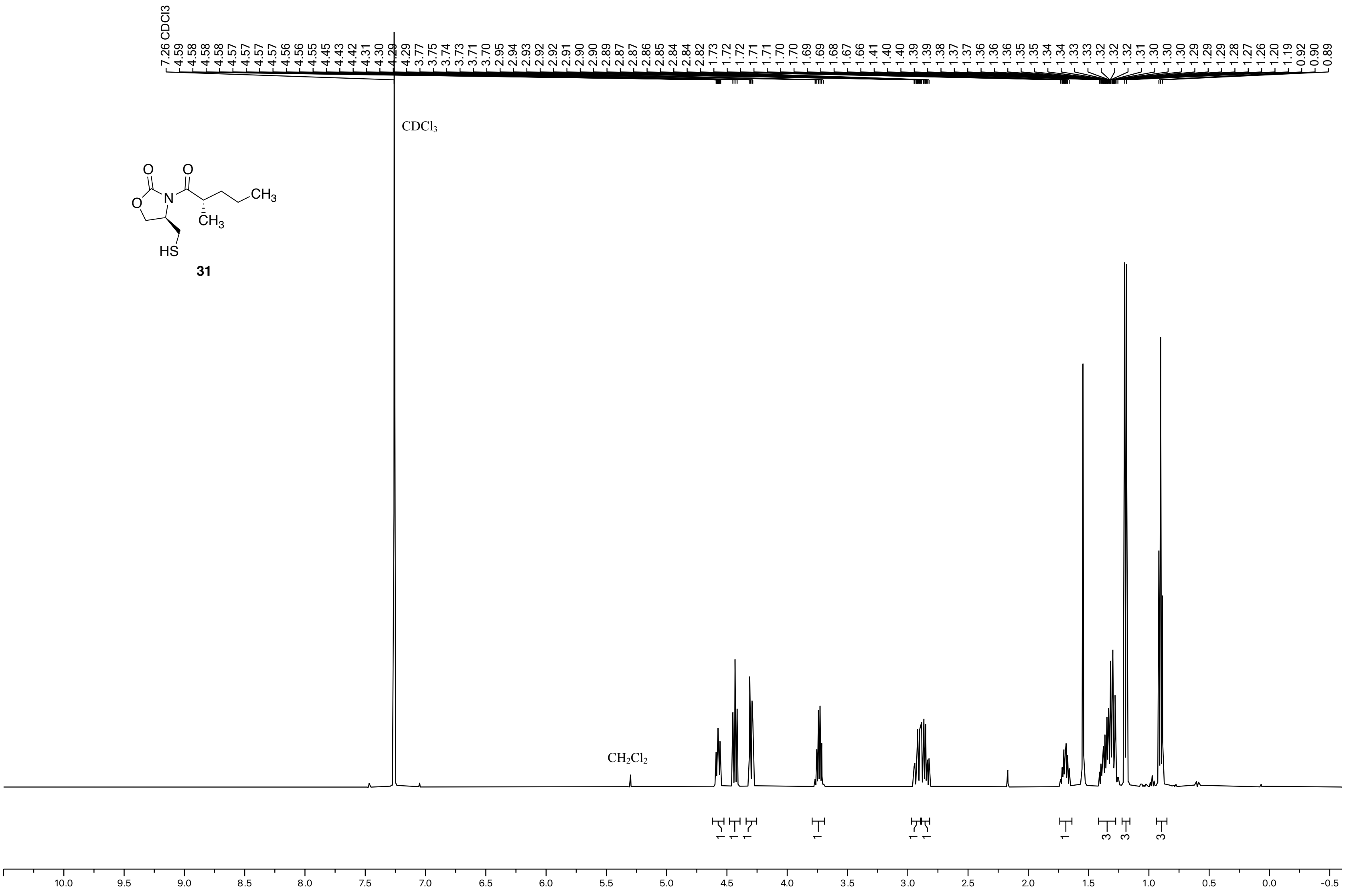

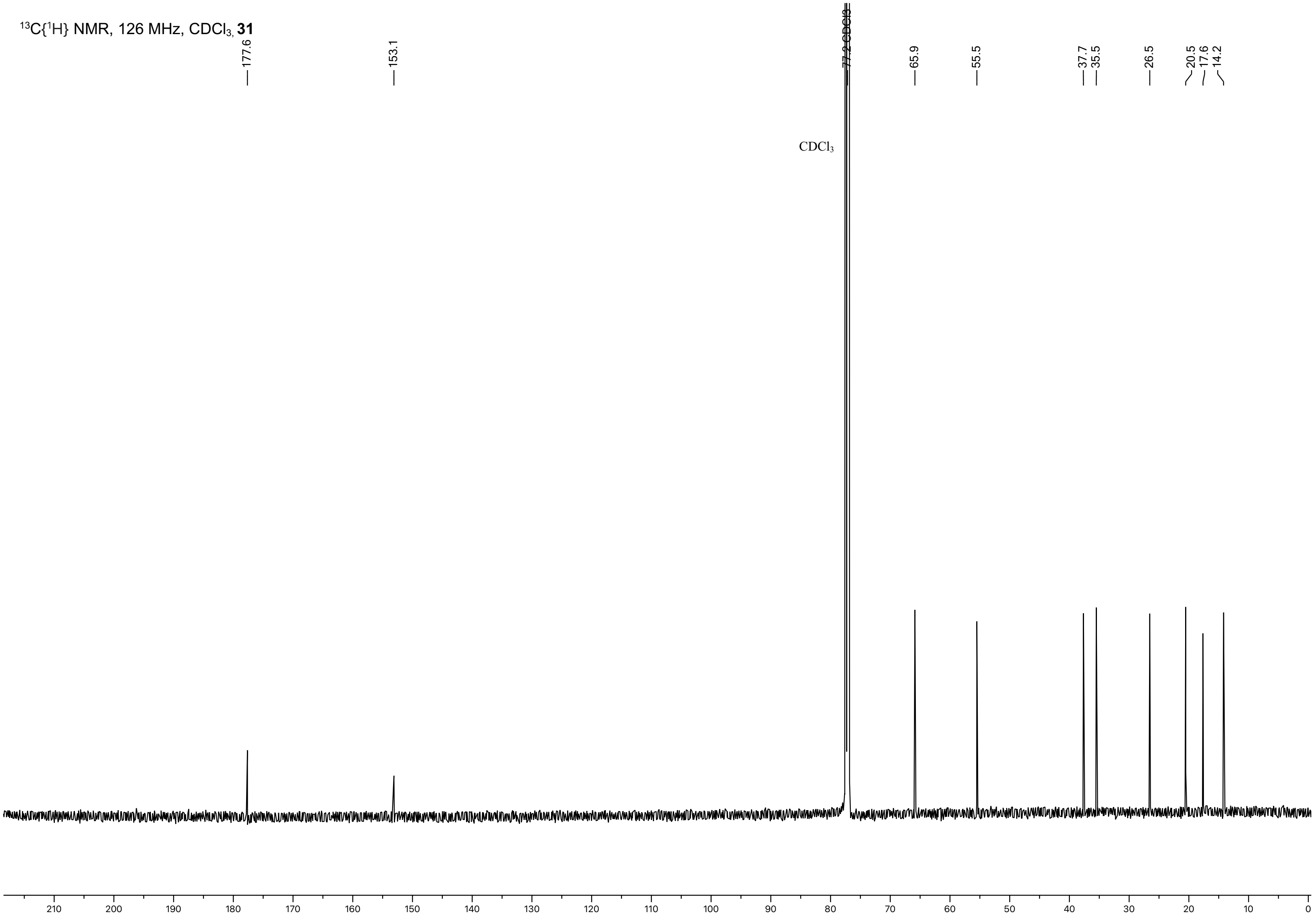

$^{13}C\{^1H\}$ NMR, 126 MHz, $CDCl_3$, **31**
177.6
153.1
77.2 CDCl3
65.9
55.5
37.7
35.5
26.5
20.5
17.6
14.2
$CDCl_3$
210
200
190
180
170
160
150
140
130
120
110
100
90
80
70
60
50
40
30
20
10
0

$^1H$ NMR, 500 MHz, $CDCl_3$, **32**

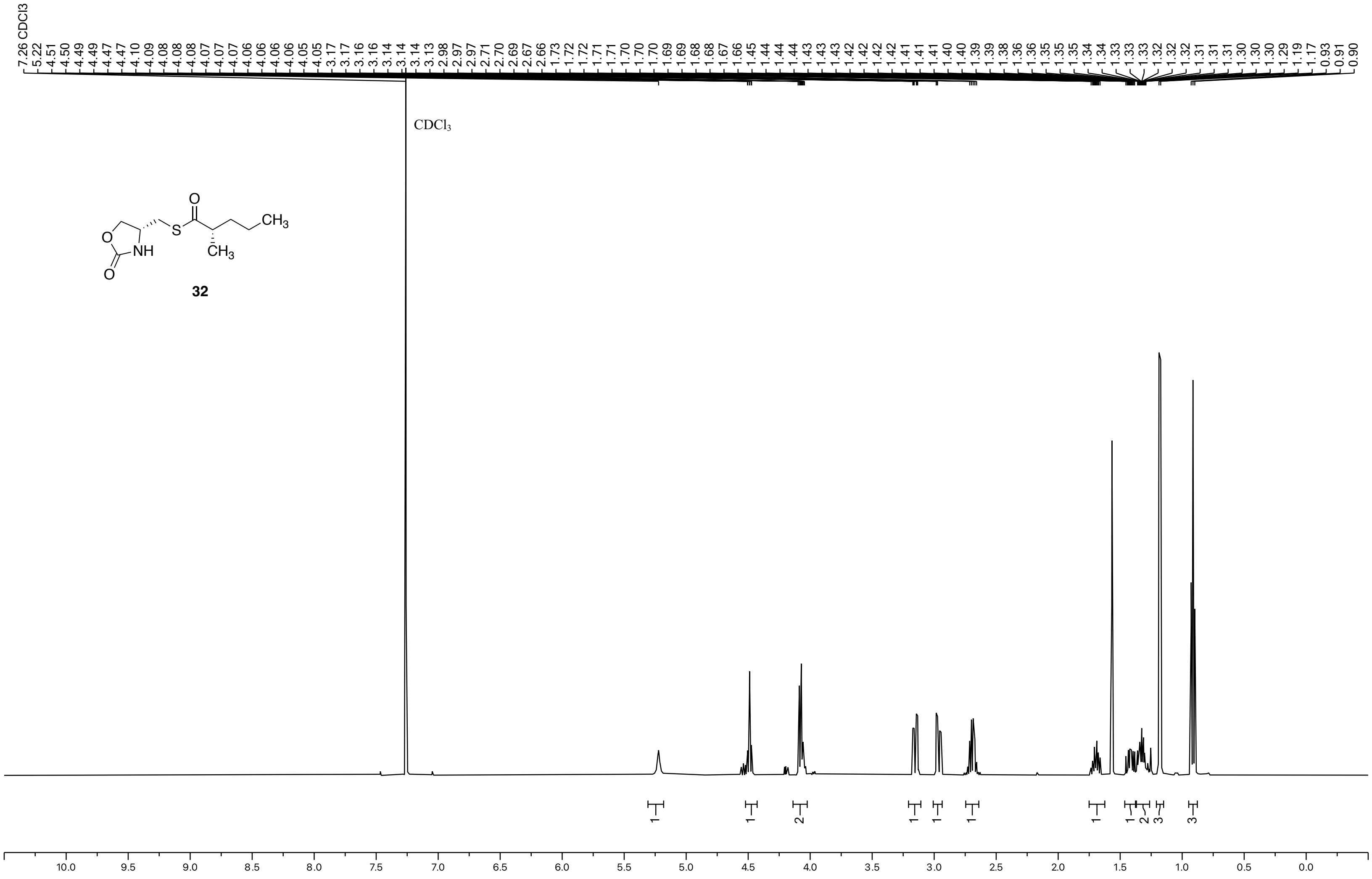

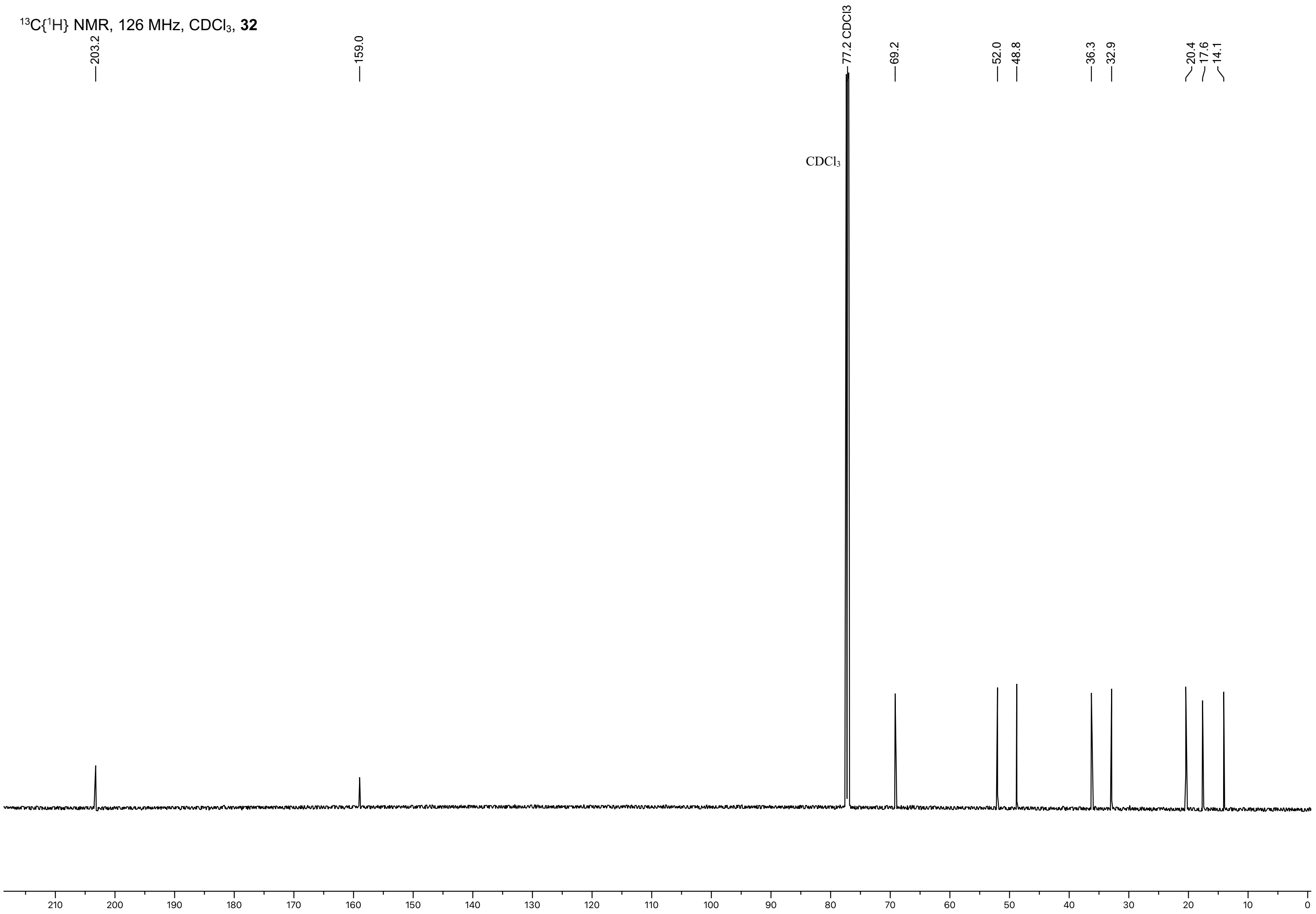

$^{13}C\{^1H\}$ NMR, 126 MHz, $CDCl_3$, **32**
203.2
159.0
77.2 CDCl3
69.2
52.0
48.8
36.3
32.9
20.4
17.6
14.1
CDCl3
210
200
190
180
170
160
150
140
130
120
110
100
90
80
70
60
50
40
30
20
10
0

$^{1}H$ NMR, 500 MHz, $CDCl_3$, **33**

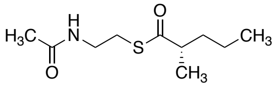


**33**

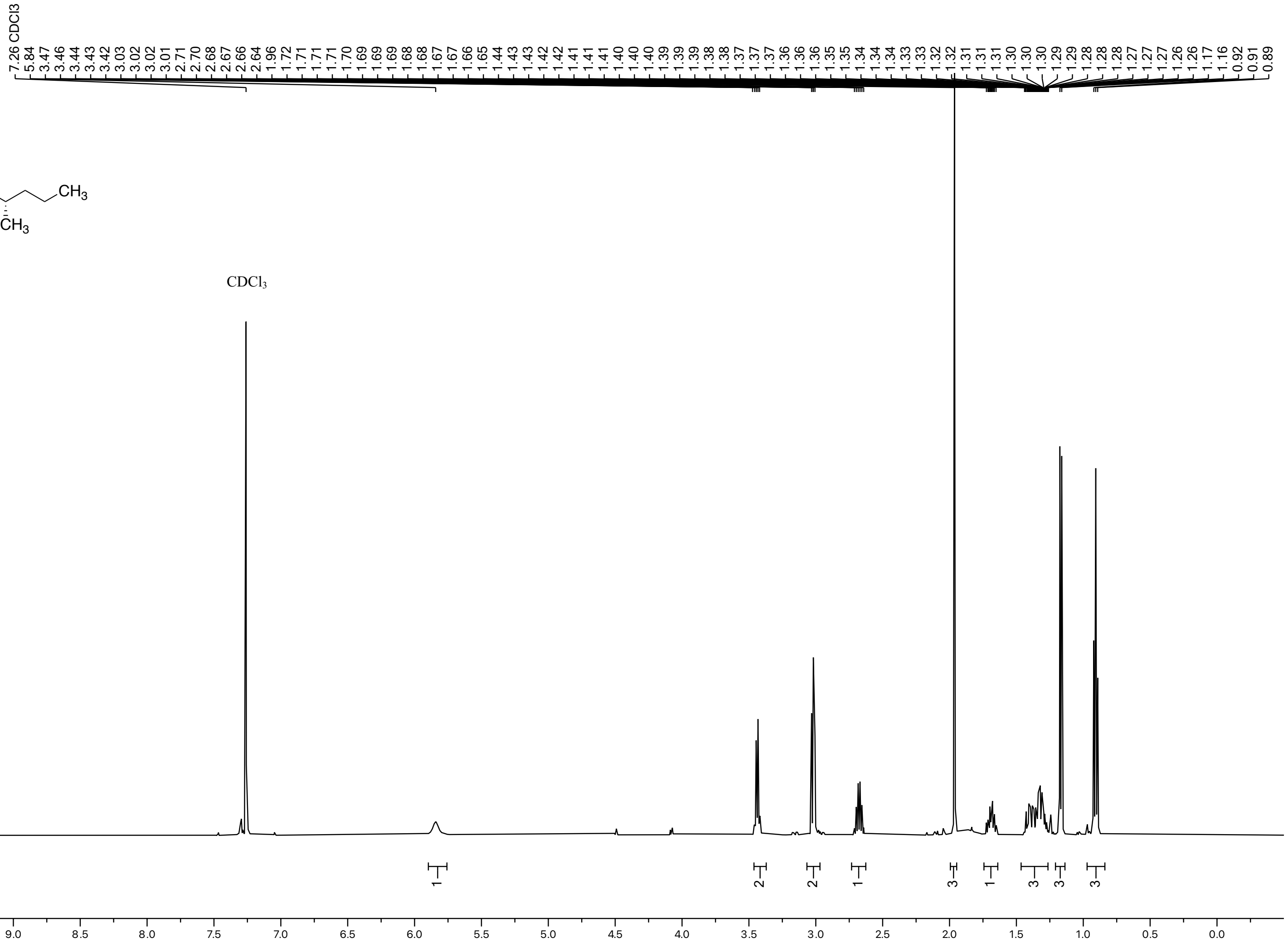

$^{13}C\{^1H\}$ NMR, 126 MHz, $CDCl_3$, **33**

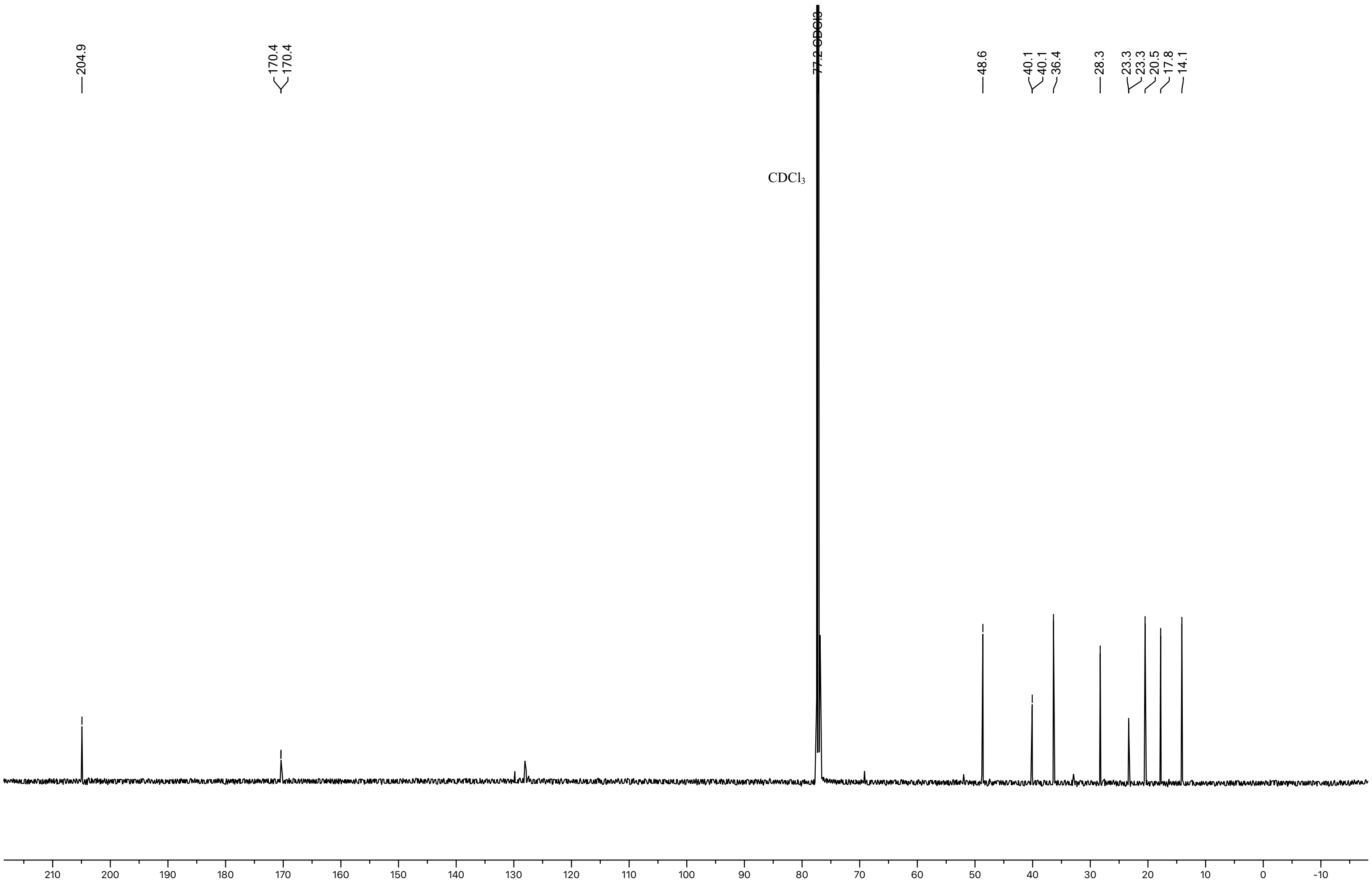

[1]H NMR, 600 MHz, $CD_3OD$, **34**

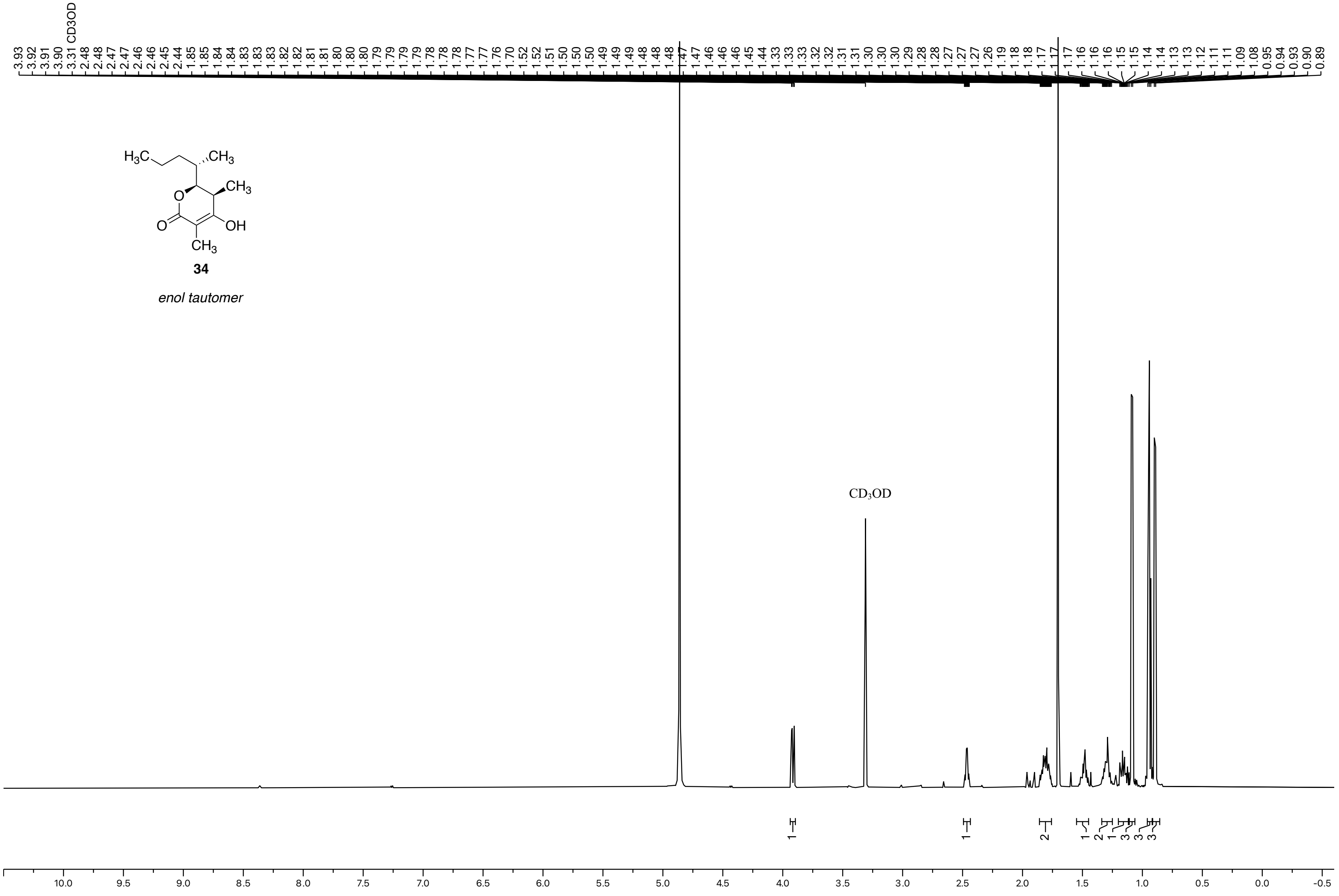


**34**

*enol tautomer*

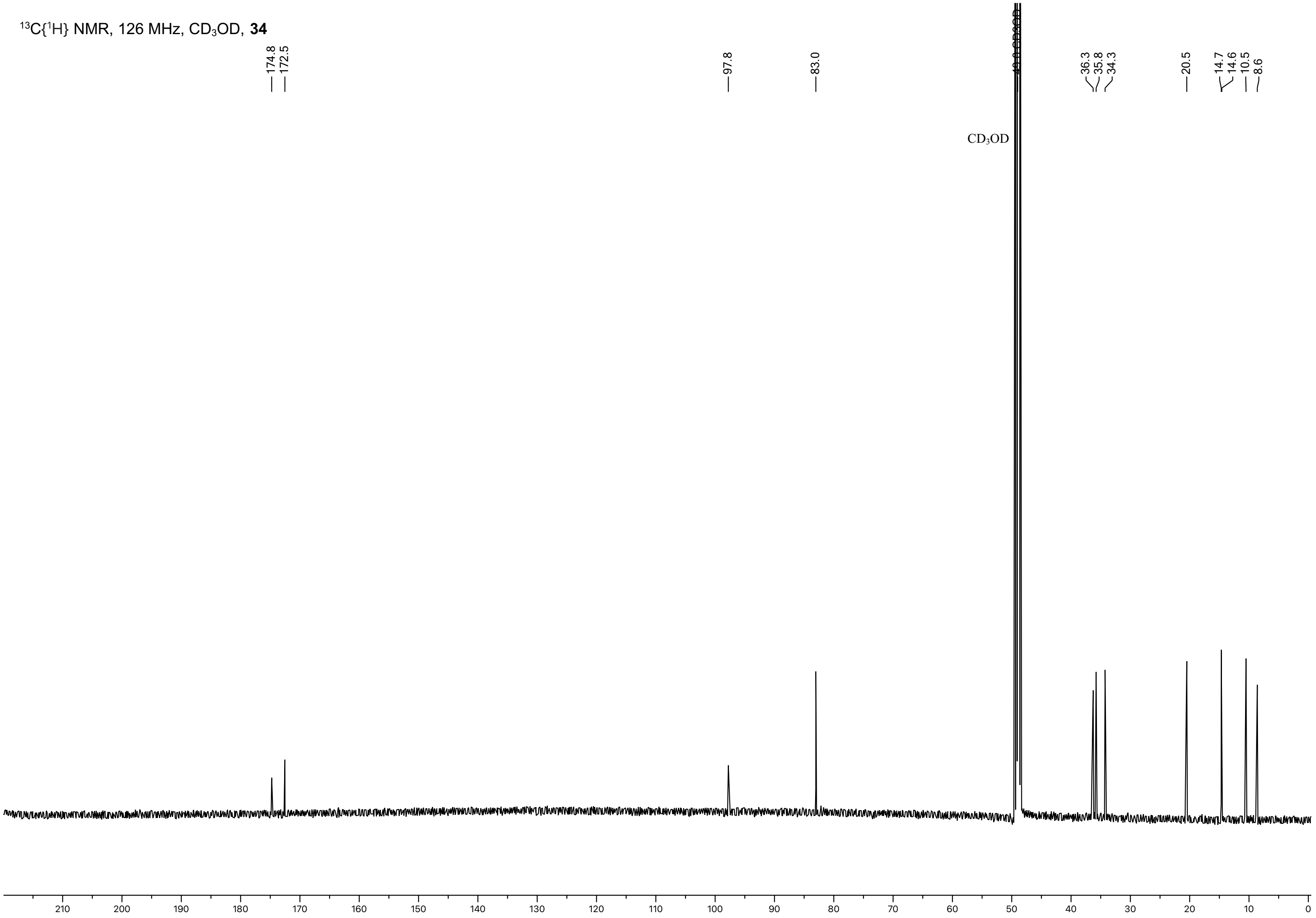

$^{13}C\{^{1}H\}$ NMR, 126 MHz, $CD_3OD$, **34**
174.8
172.5
97.8
83.0
36.3
35.8
34.3
20.5
14.7
14.6
10.5
8.6
$CD_3OD$
210 200 190 180 170 160 150 140 130 120 110 100 90 80 70 60 50 40 30 20 10 0

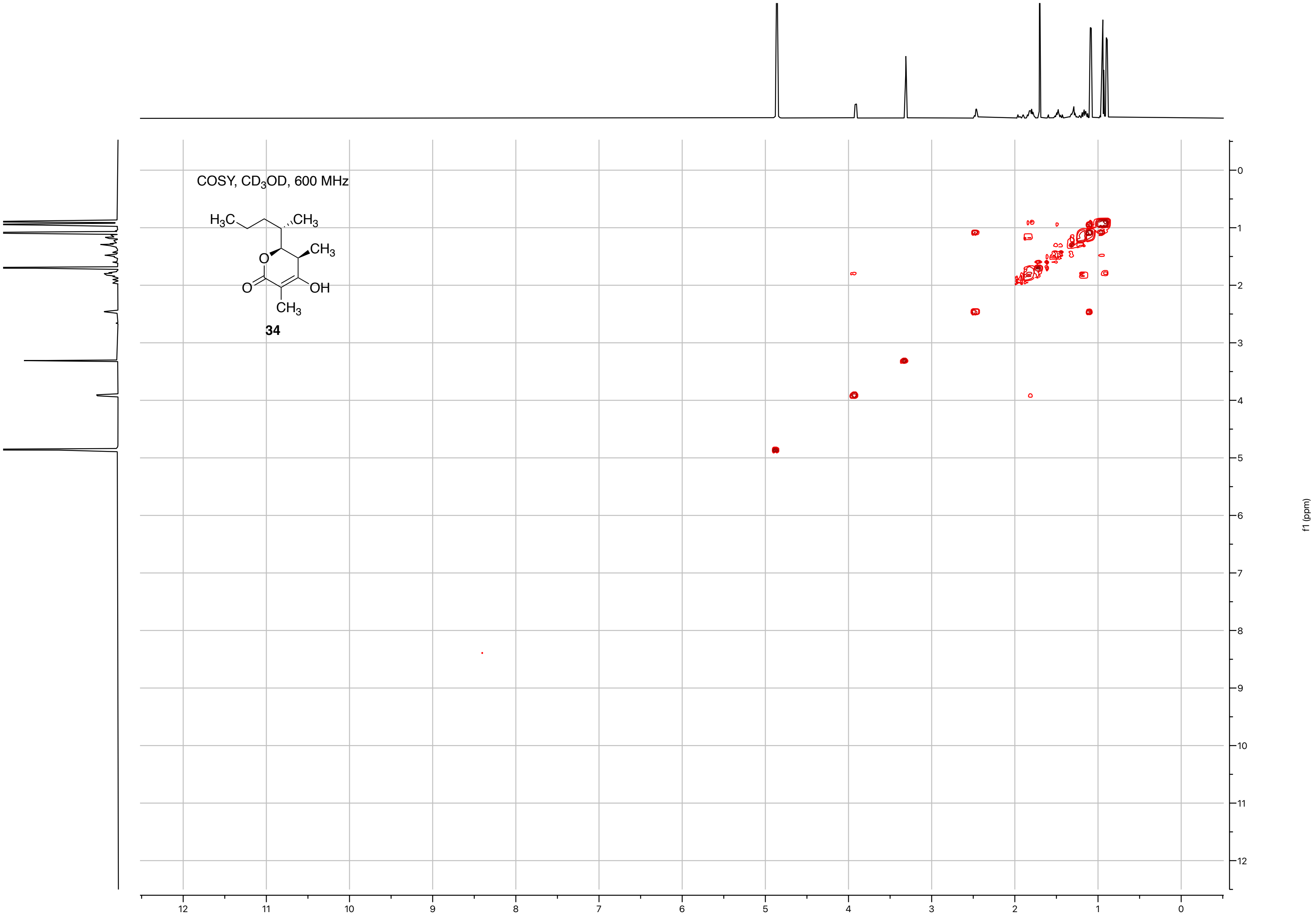
COSY, CD3OD, 600 MHz
H3C
CH3
CH3
O
O
OH
CH3
34
12
11
10
9
8
7
6
5
4
3
2
1
0
f1 (ppm)

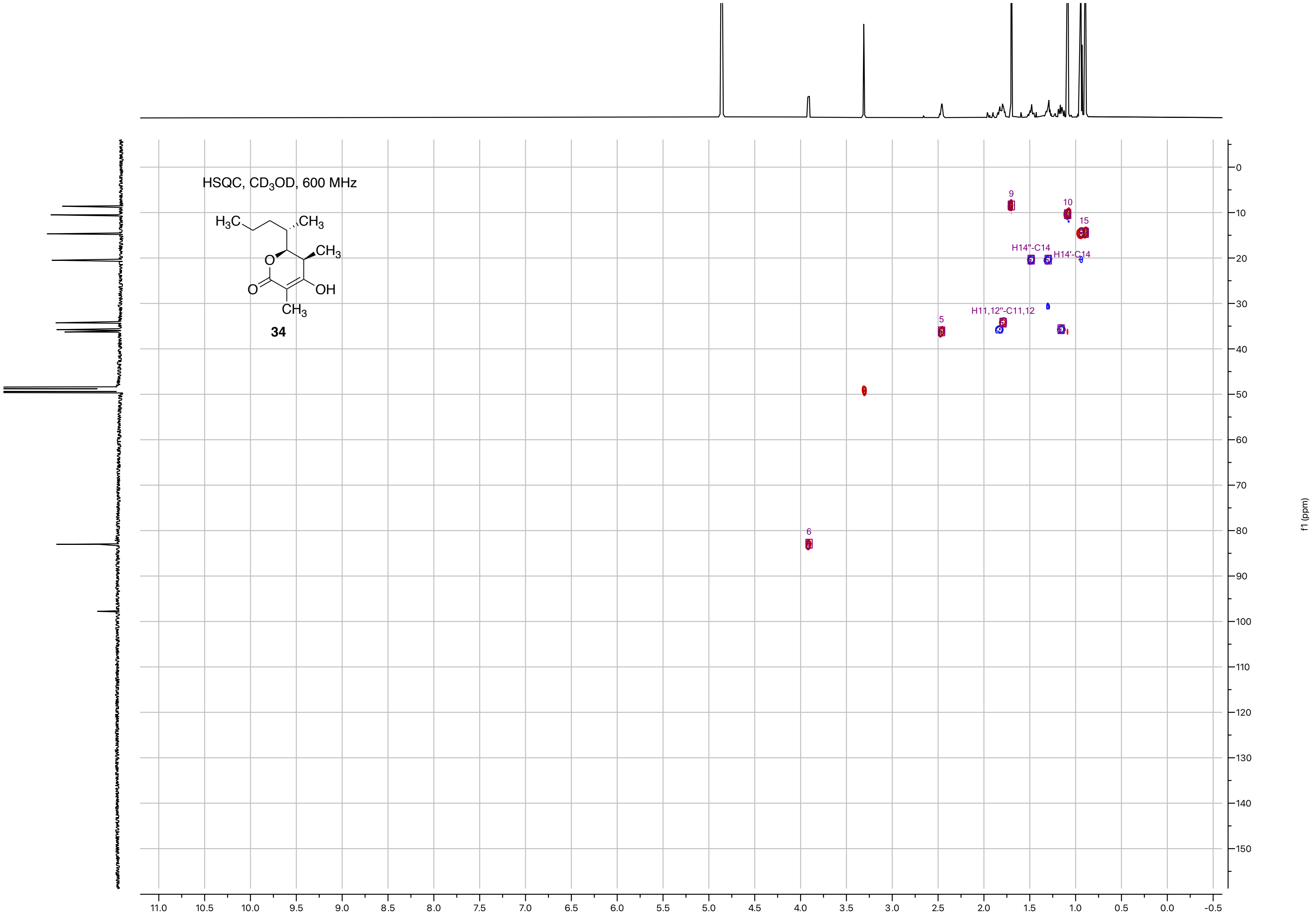

HSQC, CD3OD, 600 MHz
H3C
CH3
CH3
O
O
OH
CH3
34
9
10
15
H14"-C14
H14'-C14
H11,12"-C11,12
5
6
f1 (ppm)

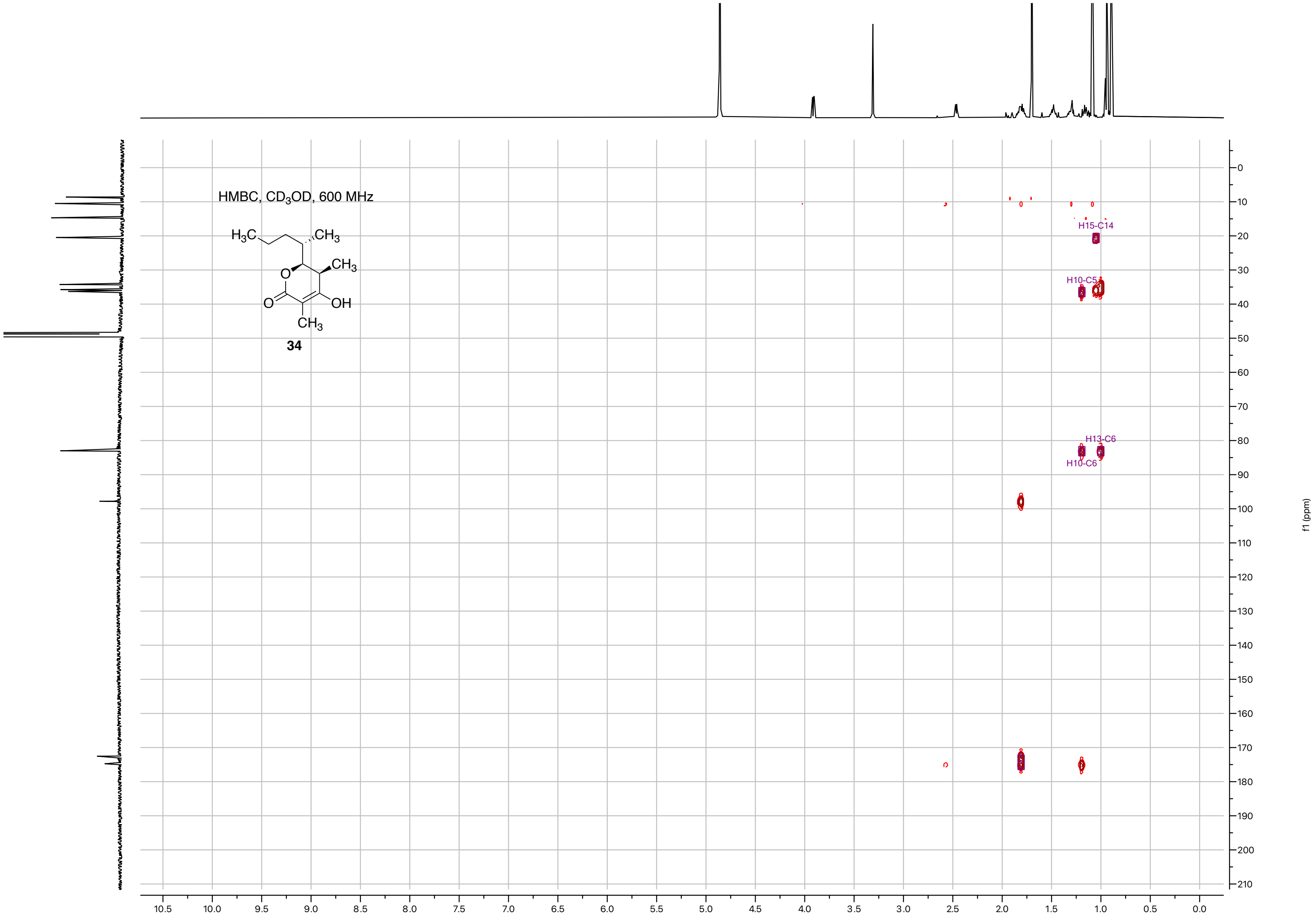

HMBC, $CD_3OD$, 600 MHz
$H_3C$
$CH_3$
$CH_3$
O
O
OH
$CH_3$
34
H15-C14
H10-C5
H13-C6
H10-C6
f1 (ppm)

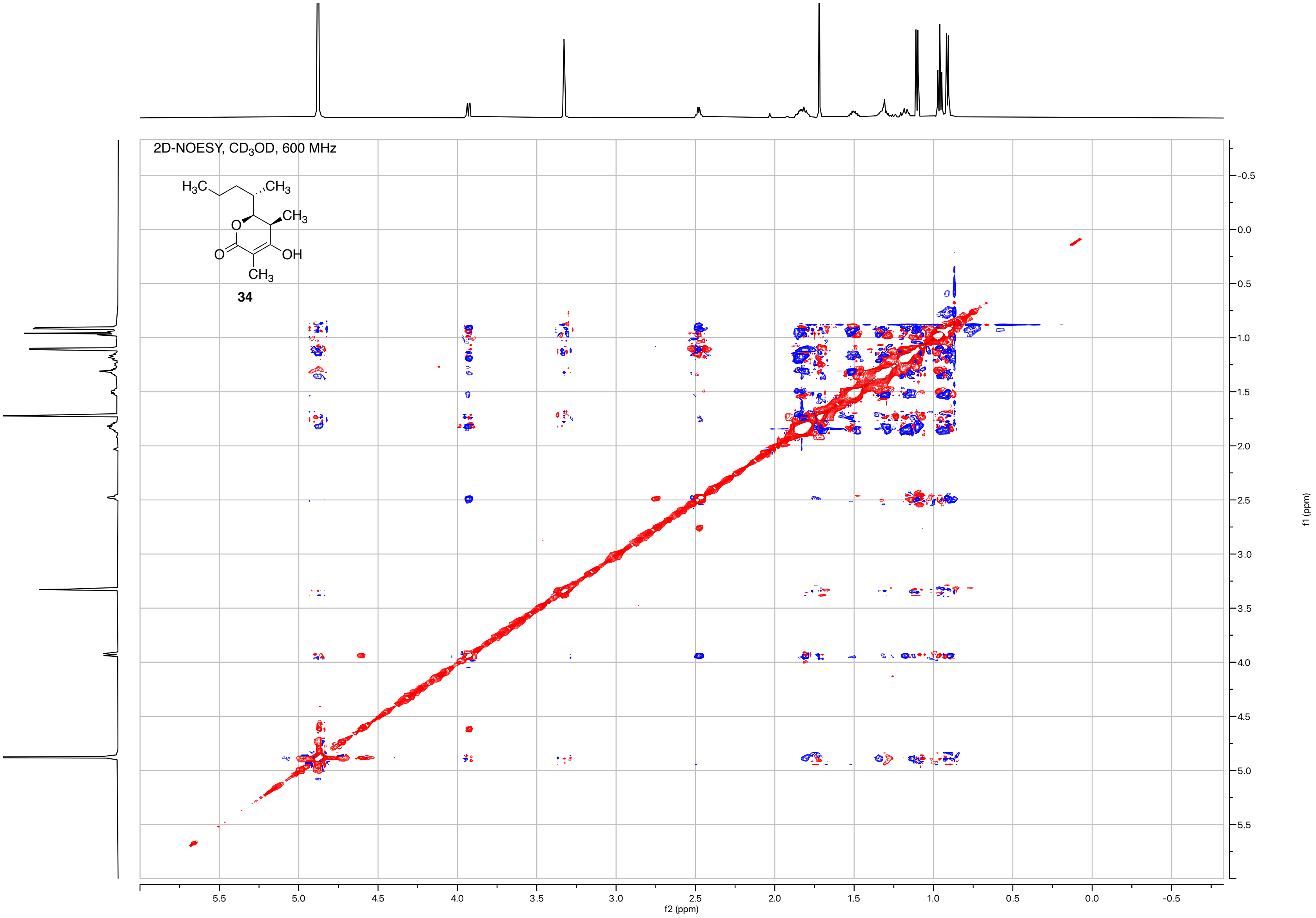
2D-NOESY, CD3OD, 600 MHz
H3C
CH3
O
CH3
O
OH
CH3
34
f2 (ppm)
f1 (ppm)

$^{1}H$ NMR, 600 MHz, $CDCl_3$, **34**

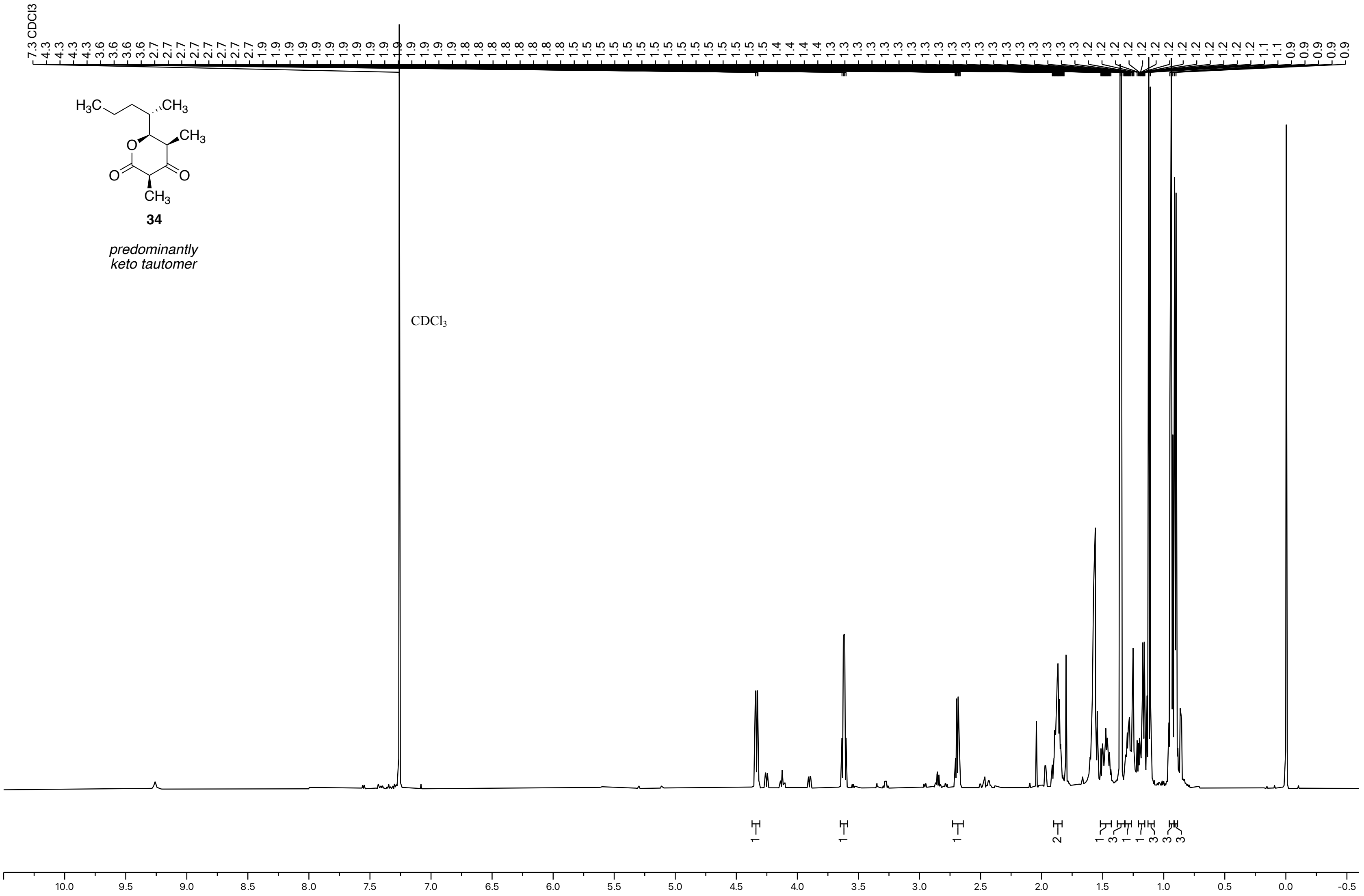


**34**

*predominantly*
*keto tautomer*

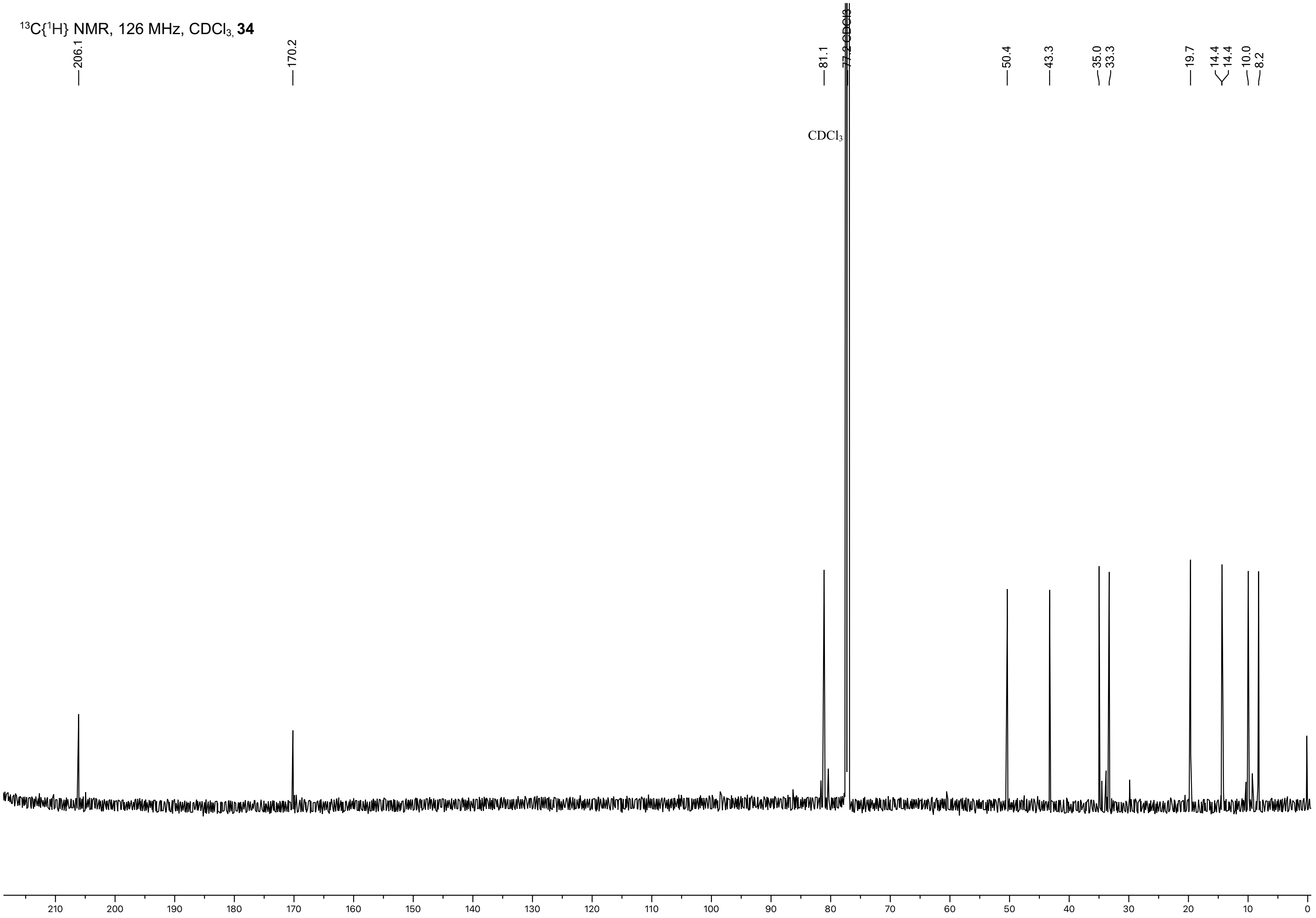

$^{13}C\{^1H\}$ NMR, 126 MHz, $CDCl_3$, **34**
206.1
170.2
81.1
77.2 CDCl3
CDCl3
50.4
43.3
35.0
33.3
19.7
14.4
14.4
10.0
8.2
210
200
190
180
170
160
150
140
130
120
110
100
90
80
70
60
50
40
30
20
10
0

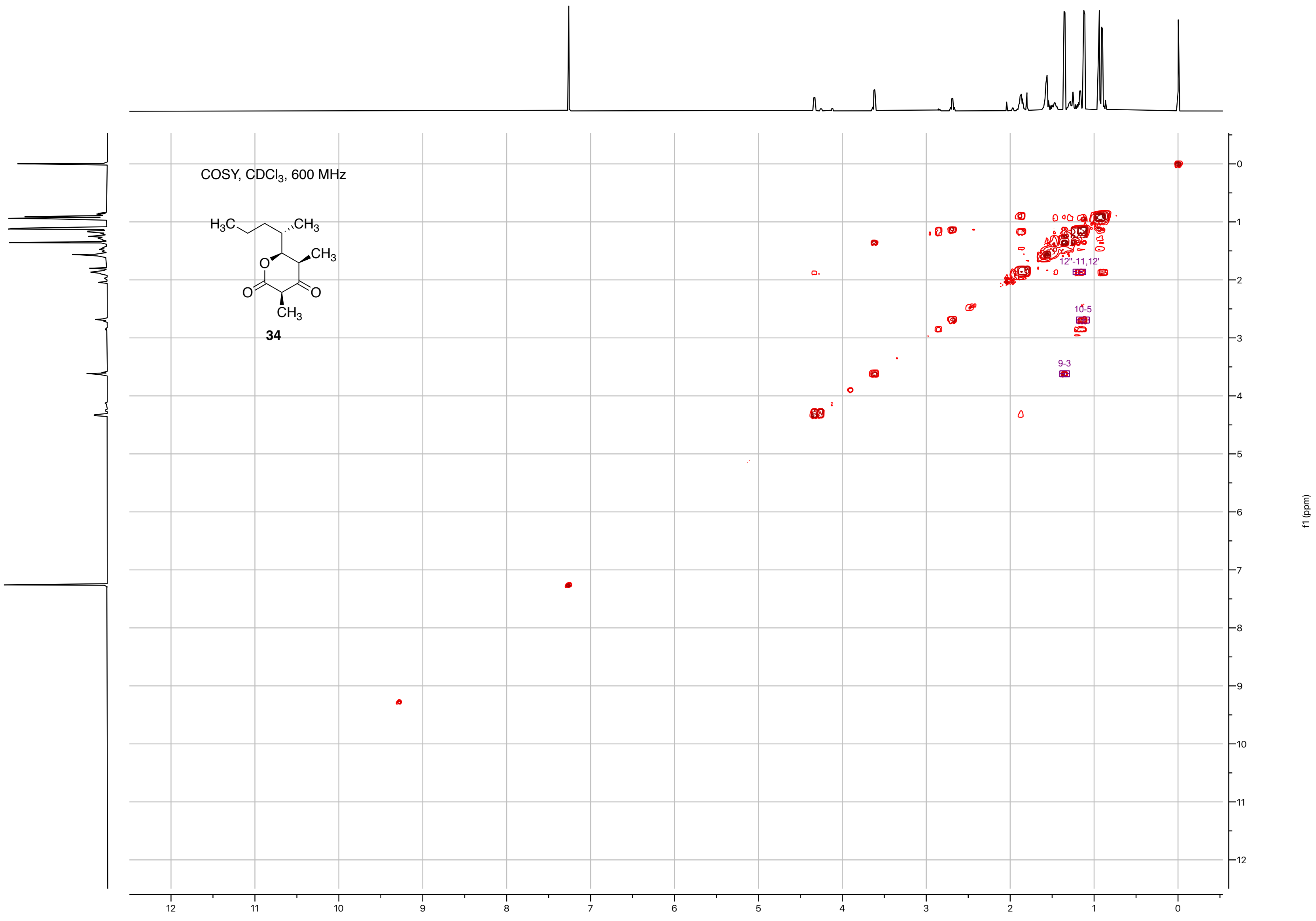

COSY, CDCl3, 600 MHz
H3C
CH3
O
CH3
O
O
CH3
34
12''-11,12'
10-5
9-3
f1 (ppm)

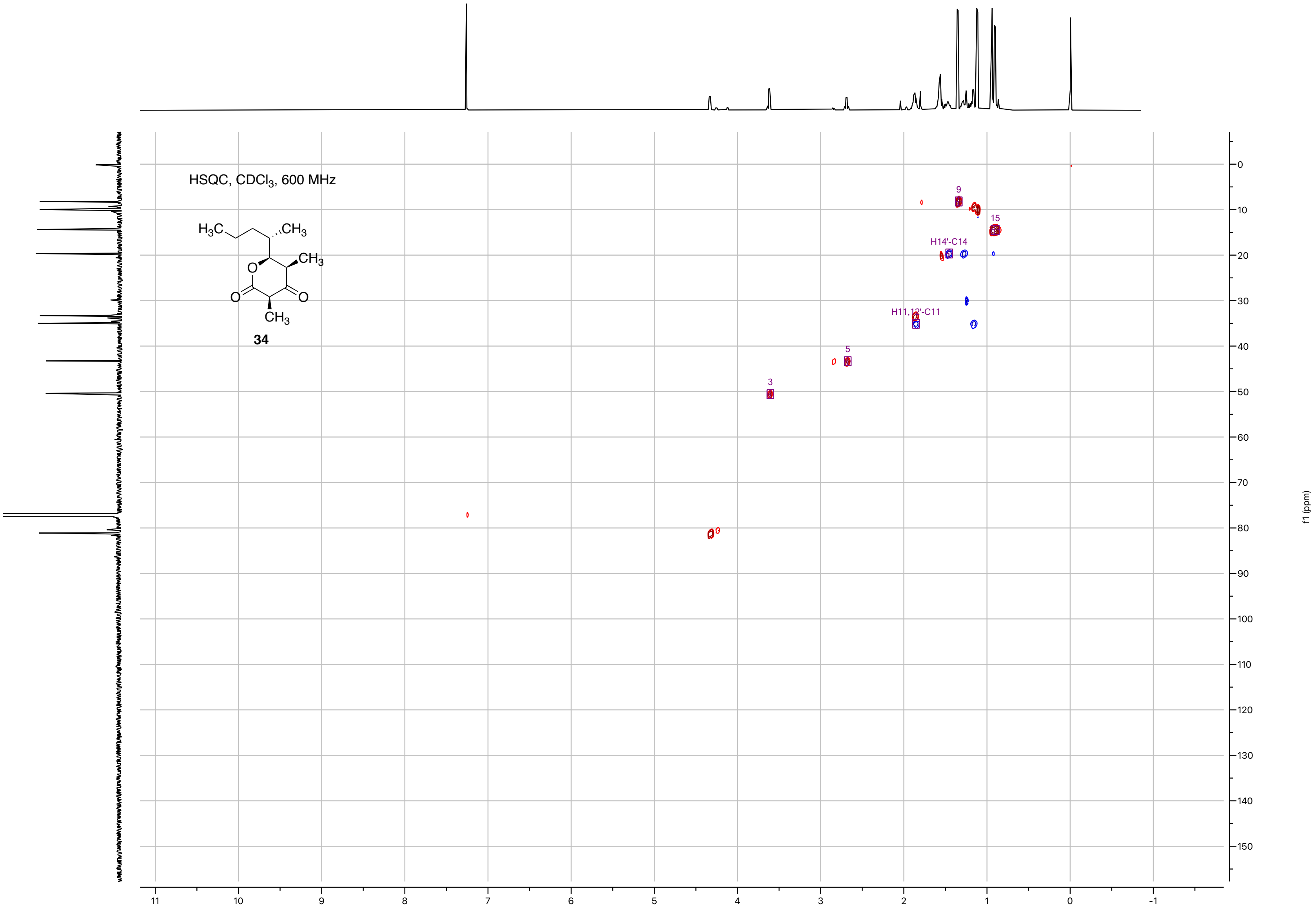
HSQC, CDCl$_3$, 600 MHz
H$_3$C
CH$_3$
CH$_3$
O
O
O
CH$_3$
34
9
15
H14'-C14
H11,12'-C11
5
3
f1 (ppm)

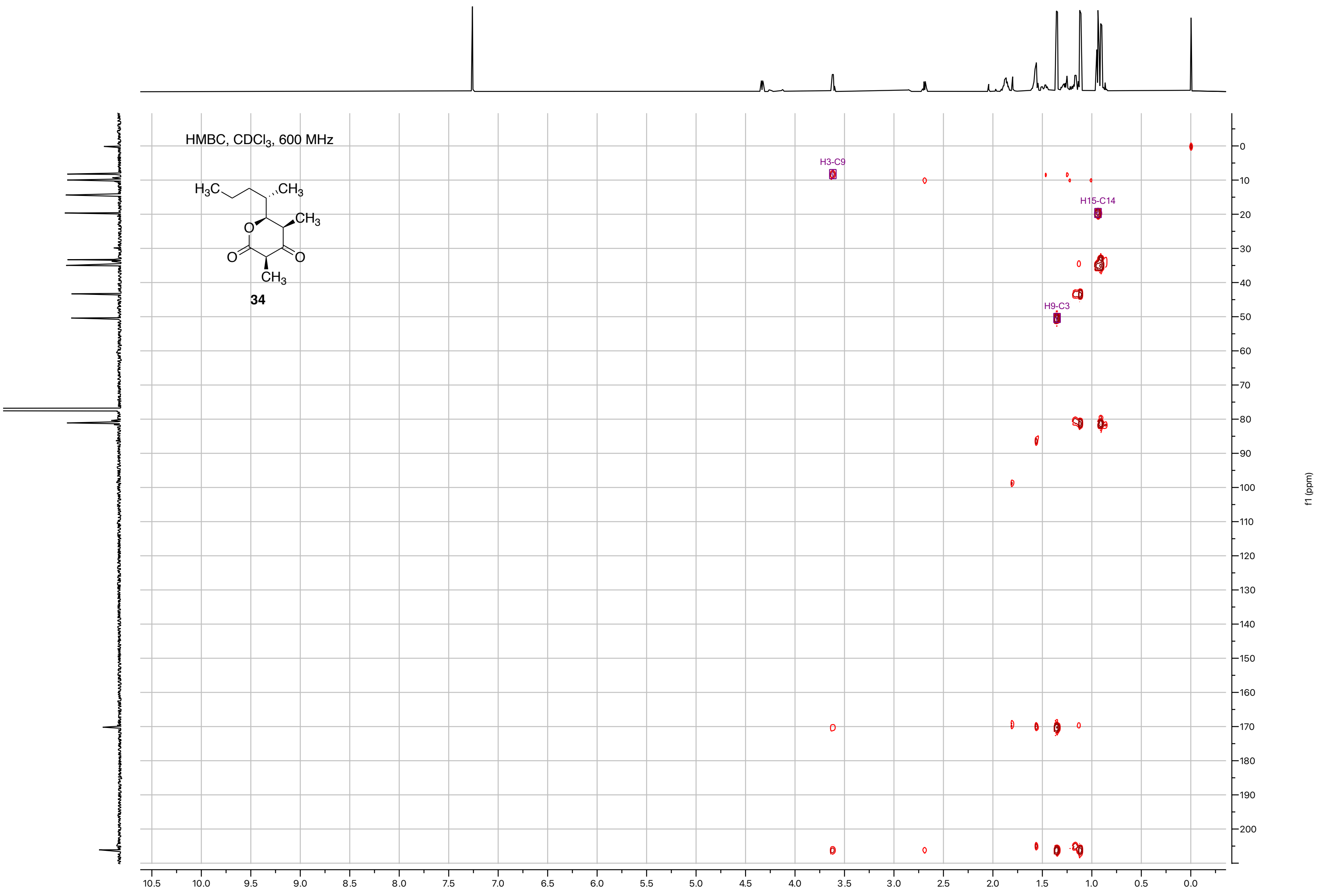
HMBC, CDCl3, 600 MHz
H3-C9
H15-C14
H9-C3
34
f1 (ppm)

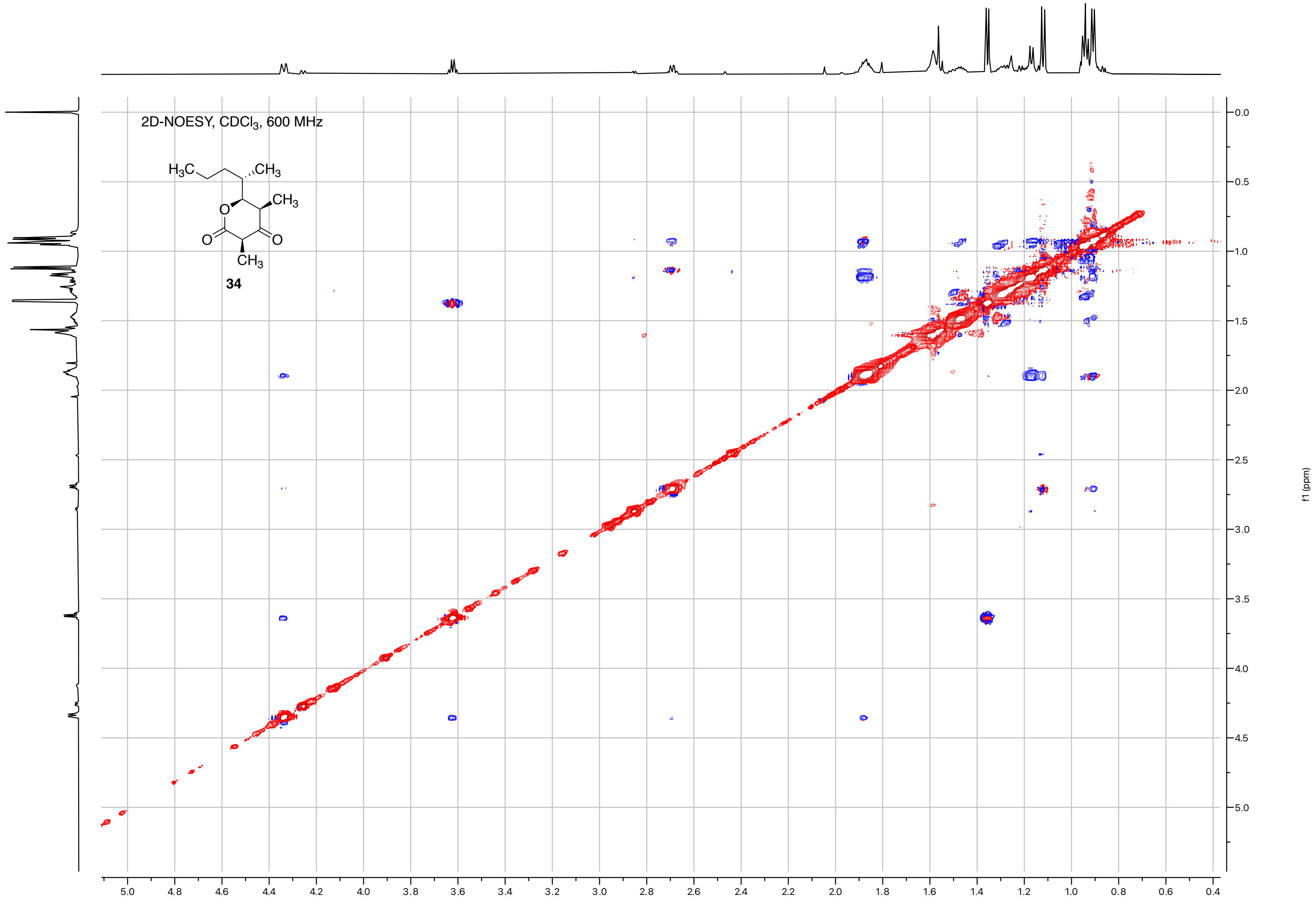

2D-NOESY, $CDCl_3$, 600 MHz
34
f1 (ppm)

[1]H NMR, 500 MHz, $CDCl_3$, **S4**

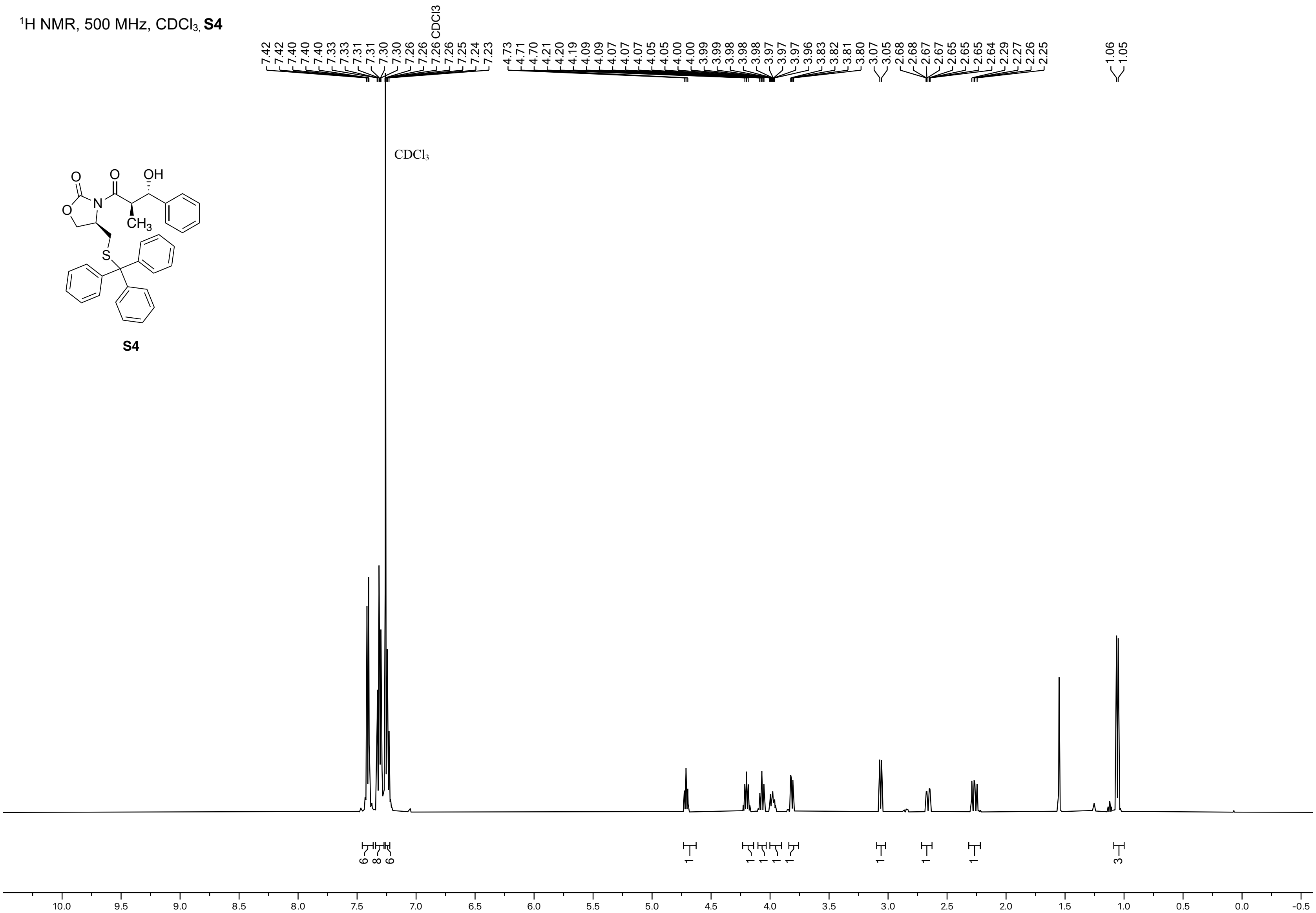

$^{13}C\{^1H\}$ NMR, 126 MHz, $CDCl_3$, **S4**

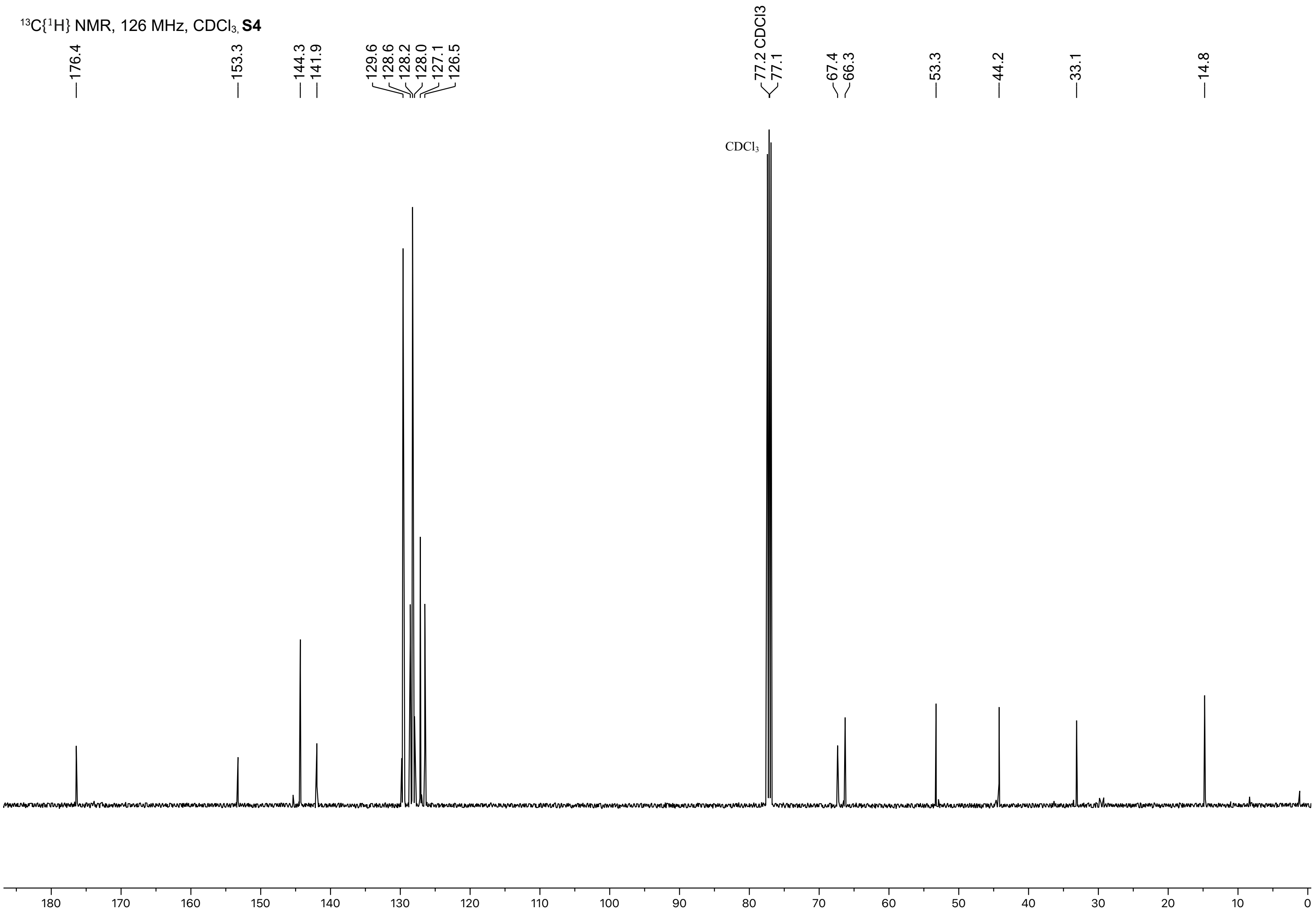

$^{1}H$ NMR, 500 MHz, $CDCl_3$, **S5**

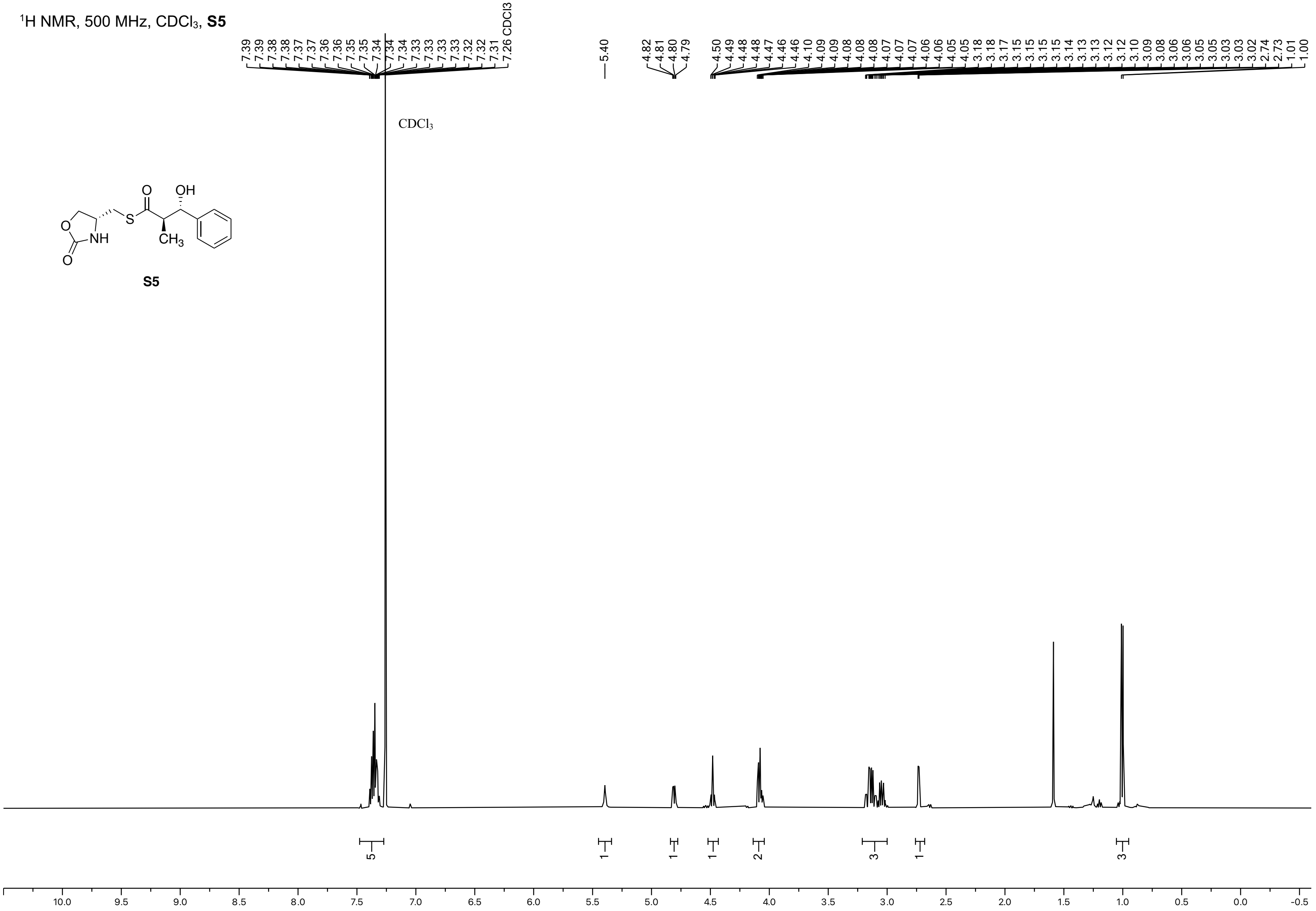


**S5**

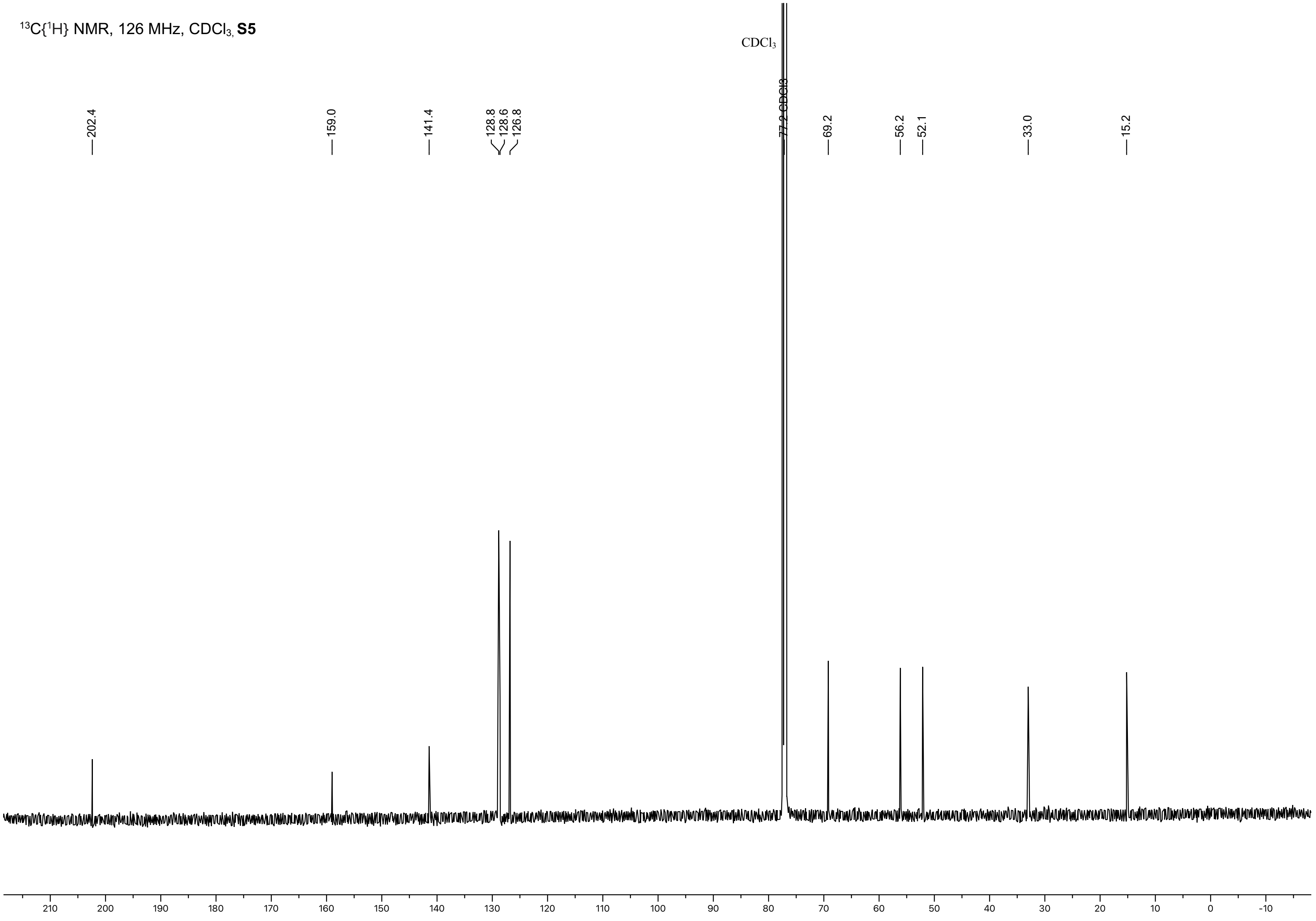

$^{13}C\{^1H\}$ NMR, 126 MHz, $CDCl_3$, **S5**
$CDCl_3$
202.4
159.0
141.4
128.8
128.6
126.8
77.2 CDCl3
69.2
56.2
52.1
33.0
15.2
210
200
190
180
170
160
150
140
130
120
110
100
90
80
70
60
50
40
30
20
10
0
-10

**Catalog of X-ray data:**

a) Crystal data for **22**

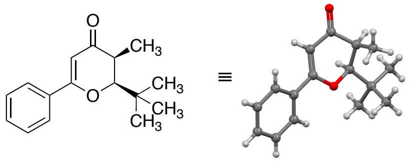


**Table 1 Crystal data and structure refinement for 22.**

| | |
|---|---|
| Identification code | **22** |
| Empirical formula | $C_{1.56}H_{1.95}O_{0.2}$ |
| Formula weight | 23.84 |
| Temperature/K | 200 |
| Crystal system | monoclinic |
| Space group | $P2_1/c$ |
| a/Å | 9.0101(4) |
| b/Å | 19.3519(9) |
| c/Å | 8.7999(4) |
| α/° | 90 |
| β/° | 116.1200(10) |
| γ/° | 90 |
| Volume/Å$^3$ | 1377.67(11) |
| Z | 41 |
| $\rho_{calc}$g/cm$^3$ | 1.178 |
| μ/mm$^{-1}$ | 0.598 |
| F(000) | 528.0 |
| Crystal size/mm$^3$ | 0.5 × 0.4 × 0.2 |
| Radiation | CuKα (λ = 1.54178) |
| 2Θ range for data collection/° | 11.856 to 132.656 |
| Index ranges | $-10 \le h \le 10$, $-22 \le k \le 22$, $-10 \le l \le 10$ |
| Reflections collected | 38197 |
| Independent reflections | 2383 [$R_{int}$ = 0.0253, $R_{sigma}$ = 0.0130] |
| Data/restraints/parameters | 2383/0/167 |
| Goodness-of-fit on F$^2$ | 0.968 |
| Final R indexes [I>=2σ (I)] | $R_1$ = 0.0403, w$R_2$ = 0.1372 |
| Final R indexes [all data] | $R_1$ = 0.0407, w$R_2$ = 0.1377 |
| Largest diff. peak/hole / e Å$^{-3}$ | 0.29/-0.32 |

**Table 2 Fractional Atomic Coordinates (×10⁴) and Equivalent Isotropic Displacement Parameters (Å²×10³) for 22. $U_{eq}$ is defined as 1/3 of the trace of the orthogonalised $U_{IJ}$ tensor.**

| Atom | *x* | *y* | *z* | U(eq) |
|---|---|---|---|---|
| O001 | 2883.3(9) | 5260.8(4) | 2136.3(9) | 27.7(3) |
| O002 | 381.1(14) | 6246.0(5) | 4371.0(14) | 52.6(3) |
| C003 | 797.1(14) | 3720.1(6) | 2529.7(14) | 30.9(3) |
| C004 | 1530.7(13) | 4199.8(6) | 1885.5(13) | 26.2(3) |
| C005 | 2696.5(15) | 6269.2(6) | 3716.1(15) | 31.4(3) |
| C006 | 619.1(15) | 3037.8(6) | 1995.9(15) | 35.7(3) |
| C007 | 792.3(15) | 5250.1(6) | 3079.9(16) | 33.6(3) |
| C008 | 1702.3(13) | 4931.6(6) | 2411.6(13) | 26.1(3) |
| C009 | 1933.1(16) | 3293.1(6) | 220.9(17) | 36.5(3) |
| C00A | 2814.4(13) | 6012.5(5) | 2130.3(14) | 27.0(3) |
| C00B | 2102.3(14) | 3977.6(6) | 731.2(15) | 30.5(3) |
| C00C | 5908.3(16) | 5959.2(8) | 2865.2(19) | 45.8(4) |
| C00D | 4168.8(18) | 6117.0(7) | 5421.7(16) | 42.6(3) |
| C00E | 4217.5(14) | 6269.3(6) | 1718.7(15) | 31.7(3) |
| C00F | 1194.3(16) | 2821.9(6) | 855.4(16) | 37.3(3) |
| C00G | 1169.8(16) | 5946.1(6) | 3738.9(16) | 35.0(3) |
| C00H | 3787.9(17) | 6060.6(8) | -107.7(17) | 45.4(4) |
| C00I | 4286(2) | 7058.8(7) | 1845(2) | 51.8(4) |

**Table 3 Anisotropic Displacement Parameters (Å²×10³) for 22. The Anisotropic displacement factor exponent takes the form: $-2\pi^2[h^2a^{*2}U_{11}+2hka^*b^*U_{12}+\ldots]$.**

| Atom | $U_{11}$ | $U_{22}$ | $U_{33}$ | $U_{23}$ | $U_{13}$ | $U_{12}$ |
|---|---|---|---|---|---|---|
| O001 | 27.8(4) | 25.6(5) | 34.8(5) | -2.1(3) | 18.5(4) | -1.9(3) |
| O002 | 66.4(7) | 46.8(6) | 67.3(7) | -4.6(5) | 50.2(6) | 9.6(5) |
| C003 | 31.1(6) | 35.6(6) | 26.3(6) | 3.2(4) | 12.8(5) | -1.3(4) |
| C004 | 22.2(5) | 29.1(6) | 25.2(6) | 1.1(4) | 8.3(4) | -0.5(4) |
| C005 | 37.9(7) | 25.1(6) | 32.3(6) | -0.5(4) | 16.5(5) | 4.5(4) |
| C006 | 38.4(7) | 32.7(6) | 32.8(6) | 7.7(5) | 12.7(5) | -5.8(5) |
| C007 | 32.4(6) | 35.7(6) | 39.5(7) | 0.7(5) | 22.0(5) | 0.9(5) |
| C008 | 23.8(6) | 30.5(6) | 24.3(6) | 2.1(4) | 10.8(4) | -0.3(4) |
| C009 | 41.6(7) | 33.8(6) | 38.5(7) | -5.5(5) | 21.7(6) | -1.7(5) |
| C00A | 26.8(6) | 24.7(6) | 28.1(6) | 1.9(4) | 10.7(5) | 2.0(4) |
| C00B | 31.1(6) | 30.0(6) | 33.1(6) | -0.6(4) | 16.7(5) | -3.3(4) |
| C00C | 28.4(7) | 57.4(8) | 50.4(8) | 9.8(6) | 16.1(6) | -0.9(5) |
| C00D | 48.4(8) | 44.4(7) | 29.4(7) | -3.4(5) | 12.0(6) | 1.4(6) |
| C00E | 29.4(6) | 32.5(6) | 34.4(6) | 3.1(5) | 15.1(5) | -2.7(4) |

**Table 3 Anisotropic Displacement Parameters (Å²×10³) for 22. The Anisotropic displacement factor exponent takes the form: $-2\pi^2[h^2a^{*2}U_{11}+2hka^*b^*U_{12}+\ldots]$.**

| Atom | $U_{11}$ | $U_{22}$ | $U_{33}$ | $U_{23}$ | $U_{13}$ | $U_{12}$ |
|---|---|---|---|---|---|---|
| C00F | 43.5(7) | 26.3(6) | 38.5(7) | -0.7(5) | 14.9(6) | -2.6(5) |
| C00G | 40.4(7) | 35.9(6) | 34.3(6) | 4.1(5) | 21.6(6) | 9.9(5) |
| C00H | 38.2(7) | 64.7(9) | 39.7(7) | 1.9(6) | 23.1(6) | -4.8(6) |
| C00I | 58.9(9) | 35.7(7) | 69.3(10) | 5.8(6) | 35.9(8) | -8.0(6) |

**Table 4 Bond Lengths for 22.**

| Atom | Atom | Length/Å | Atom | Atom | Length/Å |
|---|---|---|---|---|---|
| O001 | C008 | 1.3504(13) | C006 | C00F | 1.3815(18) |
| O001 | C00A | 1.4560(12) | C007 | C008 | 1.3501(16) |
| O002 | C00G | 1.2244(15) | C007 | C00G | 1.4464(17) |
| C003 | C004 | 1.3963(16) | C009 | C00B | 1.3854(16) |
| C003 | C006 | 1.3869(17) | C009 | C00F | 1.3833(18) |
| C004 | C008 | 1.4768(15) | C00A | C00E | 1.5425(15) |
| C004 | C00B | 1.3937(16) | C00C | C00E | 1.5315(17) |
| C005 | C00A | 1.5284(15) | C00E | C00H | 1.5324(18) |
| C005 | C00D | 1.5317(17) | C00E | C00I | 1.5312(17) |
| C005 | C00G | 1.5192(17) | | | |

**Table 5 Bond Angles for 2.**

| Atom | Atom | Atom | Angle/° | Atom | Atom | Atom | Angle/° |
|---|---|---|---|---|---|---|---|
| C008 | O001 | C00A | 115.86(8) | O001 | C00A | C005 | 109.96(8) |
| C006 | C003 | C004 | 120.02(11) | O001 | C00A | C00E | 106.54(8) |
| C003 | C004 | C008 | 120.87(10) | C005 | C00A | C00E | 120.22(9) |
| C00B | C004 | C003 | 118.92(11) | C009 | C00B | C004 | 120.53(11) |
| C00B | C004 | C008 | 120.21(10) | C00C | C00E | C00A | 113.55(9) |
| C00A | C005 | C00D | 116.99(10) | C00C | C00E | C00H | 107.85(11) |
| C00G | C005 | C00A | 107.80(9) | C00C | C00E | C00I | 110.29(11) |
| C00G | C005 | C00D | 107.85(10) | C00H | C00E | C00A | 107.86(9) |
| C00F | C006 | C003 | 120.63(11) | C00I | C00E | C00A | 108.37(10) |
| C008 | C007 | C00G | 121.27(10) | C00I | C00E | C00H | 108.81(11) |
| O001 | C008 | C004 | 112.21(9) | C006 | C00F | C009 | 119.67(11) |
| C007 | C008 | O001 | 122.71(10) | O002 | C00G | C005 | 121.39(12) |
| C007 | C008 | C004 | 125.07(10) | O002 | C00G | C007 | 122.85(12) |
| C00F | C009 | C00B | 120.21(11) | C007 | C00G | C005 | 115.62(9) |

**Table 6 Torsion Angles for 22.**

| A | B | C | D | Angle/° | A | B | C | D | Angle/° |
|---|---|---|---|---|---|---|---|---|---|
| O001 | C00A | C00E | C00C | -52.42(13) | C008 | C007 | C00G | C005 | -3.36(17) |
| O001 | C00A | C00E | C00H | 67.03(12) | C00A | O001 | C008 | C004 | 162.32(9) |
| O001 | C00A | C00E | C00I | -175.32(10) | C00A | O001 | C008 | C007 | -17.16(15) |
| C003 | C004 | C008 | O001 | 159.36(10) | C00A | C005 | C00G | O002 | -149.06(12) |
| C003 | C004 | C008 | C007 | -21.18(17) | C00A | C005 | C00G | C007 | 35.12(13) |
| C003 | C004 | C00B | C009 | 0.52(17) | C00B | C004 | C008 | O001 | -20.67(14) |
| C003 | C006 | C00F | C009 | -0.96(19) | C00B | C004 | C008 | C007 | 158.79(12) |
| C004 | C003 | C006 | C00F | 1.42(18) | C00B | C009 | C00F | C006 | 0.28(19) |
| C005 | C00A | C00E | C00C | 73.39(14) | C00D | C005 | C00A | O001 | 63.95(13) |
| C005 | C00A | C00E | C00H | -167.16(10) | C00D | C005 | C00A | C00E | -60.24(14) |
| C005 | C00A | C00E | C00I | -49.52(14) | C00D | C005 | C00G | O002 | 83.77(14) |
| C006 | C003 | C004 | C008 | 178.78(10) | C00D | C005 | C00G | C007 | -92.04(12) |
| C006 | C003 | C004 | C00B | -1.19(17) | C00F | C009 | C00B | C004 | -0.07(19) |
| C008 | O001 | C00A | C005 | 50.64(12) | C00G | C005 | C00A | O001 | -57.70(11) |
| C008 | O001 | C00A | C00E | -177.57(9) | C00G | C005 | C00A | C00E | 178.11(9) |
| C008 | C004 | C00B | C009 | -179.45(10) | C00G | C007 | C008 | O001 | -7.78(18) |
| C008 | C007 | C00G | O002 | -179.10(12) | C00G | C007 | C008 | C004 | 172.81(10) |

**Table 7 Hydrogen Atom Coordinates (Å×$10^4$) and Isotropic Displacement Parameters (Å$^2$×$10^3$) for 22.**

| Atom | *x* | *y* | *z* | U(eq) |
|---|---|---|---|---|
| H003 | 427.82 | 3858.26 | 3316.8 | 37 |
| H005 | 2534.38 | 6770.9 | 3617.27 | 38 |
| H006 | 107.5 | 2722.65 | 2409.72 | 43 |
| H007 | -94.31 | 5017.6 | 3117.37 | 40 |
| H009 | 2317.42 | 3149.87 | -550.8 | 44 |
| H00A | 1775.44 | 6142.78 | 1166.1 | 32 |
| H00B | 2601.36 | 4291.3 | 300.17 | 37 |
| H00C | 6234.7 | 6104.33 | 4011.5 | 69 |
| H00D | 6710.21 | 6114.88 | 2499 | 69 |
| H00E | 5840.03 | 5464.18 | 2803.91 | 69 |
| H00F | 4462.77 | 5637.82 | 5472.08 | 64 |
| H00G | 3874.24 | 6220.04 | 6321.05 | 64 |
| H00H | 5092.32 | 6397.7 | 5540.82 | 64 |
| H00I | 1085.41 | 2362.08 | 515.98 | 45 |
| H00J | 3715.03 | 5566.43 | -206.76 | 68 |
| H00K | 4633.26 | 6223.61 | -403.12 | 68 |
| H00L | 2746.18 | 6261.23 | -857.33 | 68 |

**Table 7 Hydrogen Atom Coordinates (Å×10⁴) and Isotropic Displacement Parameters (Å²×10³) for 22.**

| Atom | *x* | *y* | *z* | U(eq) |
|---|---|---|---|---|
| H00M | 3200.24 | 7244.96 | 1190.9 | 78 |
| H00N | 5025.33 | 7232.43 | 1415.52 | 78 |
| H00O | 4673.56 | 7194.02 | 3006.78 | 78 |

CCDC 2287271 contains the supplementary crystallographic data for this paper, including structure factors and refinement instructions. These data can be obtained free of charge from The Cambridge Crystallographic Data Centre, 12 Union Road.

b) Crystal data of **25**

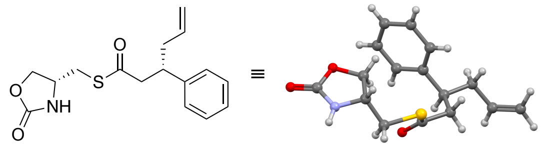

**Table 1 Crystal data and structure refinement for 25.**

| | |
|---|---|
| Identification code | **25** |
| Empirical formula | $C_{16}H_{18}NO_3S$ |
| Formula weight | 304.37 |
| Temperature/K | 140 |
| Crystal system | monoclinic |
| Space group | $P2_1$ |
| a/Å | 5.6041(2) |
| b/Å | 9.2910(4) |
| c/Å | 14.5164(6) |
| α/° | 90 |
| β/° | 91.0070(10) |
| γ/° | 90 |
| Volume/Å$^3$ | 755.72(5) |
| Z | 2 |
| $\rho_{calc}$g/cm$^3$ | 1.338 |
| μ/mm$^{-1}$ | 1.986 |
| F(000) | 322.0 |
| Crystal size/mm$^3$ | 0.2 × 0.03 × 0.03 |
| Radiation | CuKα (λ = 1.54178) |
| 2Θ range for data collection/° | 6.09 to 132.662 |
| Index ranges | $-6 \leq h \leq 6$, $-10 \leq k \leq 10$, $-17 \leq l \leq 17$ |
| Reflections collected | 16353 |
| Independent reflections | 2573 [$R_{int}$ = 0.0239, $R_{sigma}$ = 0.0234] |
| Data/restraints/parameters | 2573/1/198 |
| Goodness-of-fit on $F^2$ | 0.973 |
| Final R indexes [I>=2σ (I)] | $R_1$ = 0.0209, $wR_2$ = 0.0562 |
| Final R indexes [all data] | $R_1$ = 0.0210, $wR_2$ = 0.0563 |
| Largest diff. peak/hole / e Å$^{-3}$ | 0.15/-0.19 |
| Flack parameter | 0.127(4) |

**Table 2 Fractional Atomic Coordinates (×10⁴) and Equivalent Isotropic Displacement Parameters (Å²×10³) for 25. $U_{eq}$ is defined as 1/3 of the trace of the orthogonalised $U_{IJ}$ tensor.**

| Atom | *x* | *y* | *z* | U(eq) |
|---|---|---|---|---|
| S001 | 4323.6(8) | 7756.6(5) | 7493.1(3) | 27.50(14) |
| O002 | 7304(2) | 6272.1(15) | 6472.5(9) | 27.6(3) |
| O003 | 8245(3) | 4903.9(15) | 9614.9(10) | 28.9(3) |
| O004 | 11622(3) | 5558.8(19) | 10351.8(11) | 36.3(4) |
| N005 | 9342(3) | 7173.9(19) | 9514.3(11) | 27.5(4) |
| C006 | 9914(4) | 5897(2) | 9873.3(13) | 25.8(4) |
| C007 | 3393(3) | 4537(2) | 5759.5(12) | 20.3(4) |
| C008 | 5254(3) | 6637(2) | 6575.8(13) | 21.6(4) |
| C009 | 3554(3) | 3695.7(19) | 6656.1(12) | 19.7(4) |
| C00A | 1894(3) | 2982(2) | 8117.7(12) | 26.1(4) |
| C00B | 1277(3) | 4023(2) | 5155.9(12) | 22.9(4) |
| C00C | 7015(3) | 7180(2) | 9054.9(13) | 24.5(4) |
| C00D | 5506(3) | 2811(2) | 6844.0(12) | 22.5(3) |
| C00E | 1753(3) | 3777(2) | 7307.5(12) | 22.7(4) |
| C00F | 3852(4) | 2102(2) | 8292.6(13) | 25.9(4) |
| C00G | 1184(3) | 4694(2) | 4217.3(12) | 23.7(4) |
| C00H | -733(4) | 5286(2) | 3842.7(15) | 29.1(4) |
| C00I | 3235(3) | 6169(2) | 5943.0(12) | 22.0(4) |
| C00J | 7105(4) | 7964(2) | 8133.6(13) | 29.3(4) |
| C00K | 6566(4) | 5559(2) | 8972.2(14) | 27.6(4) |
| C00L | 5661(3) | 2028(2) | 7656.3(14) | 25.7(4) |

**Table 3 Anisotropic Displacement Parameters (Å²×10³) for 25. The Anisotropic displacement factor exponent takes the form: $-2\pi^2[h^2a^{*2}U_{11}+2hka^*b^*U_{12}+\ldots]$.**

| Atom | $U_{11}$ | $U_{22}$ | $U_{33}$ | $U_{23}$ | $U_{13}$ | $U_{12}$ |
|---|---|---|---|---|---|---|
| S001 | 31.0(2) | 27.1(2) | 24.2(2) | -3.0(2) | -4.62(16) | 6.2(2) |
| O002 | 20.4(7) | 29.0(7) | 33.4(7) | -3.7(6) | 0.0(5) | -1.2(5) |
| O003 | 36.6(8) | 18.6(7) | 31.2(7) | 2.3(6) | -4.5(6) | 0.7(6) |
| O004 | 39.7(9) | 35.3(9) | 33.6(8) | 2.1(7) | -11.1(7) | 6.8(7) |
| N005 | 36.5(9) | 20.5(8) | 25.1(8) | 0.5(6) | -7.3(7) | -3.9(7) |
| C006 | 31.7(10) | 24.2(9) | 21.7(9) | -2.2(7) | 0.9(8) | 2.0(8) |
| C007 | 21.4(8) | 20.9(9) | 18.4(8) | 0.8(7) | 0.6(7) | -0.7(7) |
| C008 | 25.7(9) | 17.1(8) | 22.1(9) | 3.8(7) | -1.0(7) | -1.5(7) |
| C009 | 20.5(8) | 18.3(9) | 20.2(8) | -0.5(7) | -2.1(6) | -3.0(7) |
| C00A | 29.0(9) | 29.2(12) | 20.3(8) | -0.5(7) | 3.5(7) | -1.5(9) |
| C00B | 25.4(9) | 21.7(9) | 21.4(9) | 0.6(7) | -1.9(7) | -2.0(7) |

**Table 3 Anisotropic Displacement Parameters (Å$^2$×10$^3$) for 25. The Anisotropic displacement factor exponent takes the form: $-2\pi^2[h^2a^{*2}U_{11}+2hka^*b^*U_{12}+\ldots]$.**

| Atom | $U_{11}$ | $U_{22}$ | $U_{33}$ | $U_{23}$ | $U_{13}$ | $U_{12}$ |
|---|---|---|---|---|---|---|
| C00C | 30.3(9) | 20.7(9) | 22.3(8) | -2.9(7) | -3.1(7) | 2.5(8) |
| C00D | 21.5(7) | 19.8(8) | 26.1(8) | 0.2(9) | 1.0(6) | -1.0(8) |
| C00E | 21.9(9) | 23.1(9) | 23.0(8) | -0.9(7) | -0.7(7) | 1.2(7) |
| C00F | 32.9(10) | 24.4(9) | 20.2(9) | 5.1(8) | -4.0(7) | -4.0(8) |
| C00G | 29.0(9) | 22.0(9) | 20.1(8) | -2.4(7) | -0.6(7) | -2.1(8) |
| C00H | 34.2(11) | 24.9(10) | 27.8(10) | 1.8(8) | -6.9(8) | -2.7(8) |
| C00I | 22.5(9) | 20.3(9) | 23.2(9) | 2.7(7) | -2.7(7) | 0.2(7) |
| C00J | 37.1(9) | 23.9(11) | 26.5(9) | 1.0(8) | -8.9(7) | -3.7(9) |
| C00K | 29.8(10) | 23.2(10) | 29.7(10) | 2.9(8) | -5.0(8) | -2.0(8) |
| C00L | 25.7(9) | 20.9(10) | 30.1(10) | 2.3(8) | -4.8(8) | 2.1(7) |

**Table 4 Bond Lengths for 25.**

| Atom | Atom | Length/Å | Atom | Atom | Length/Å |
|---|---|---|---|---|---|
| S001 | C008 | 1.7752(19) | C008 | C00I | 1.509(3) |
| S001 | C00J | 1.8113(19) | C009 | C00D | 1.391(3) |
| O002 | C008 | 1.210(2) | C009 | C00E | 1.397(3) |
| O003 | C006 | 1.362(3) | C00A | C00E | 1.390(3) |
| O003 | C00K | 1.448(3) | C00A | C00F | 1.388(3) |
| O004 | C006 | 1.214(3) | C00B | C00G | 1.499(2) |
| N005 | C006 | 1.333(3) | C00C | C00J | 1.524(3) |
| N005 | C00C | 1.454(3) | C00C | C00K | 1.531(3) |
| C007 | C009 | 1.520(2) | C00D | C00L | 1.387(3) |
| C007 | C00B | 1.538(2) | C00F | C00L | 1.385(3) |
| C007 | C00I | 1.542(3) | C00G | C00H | 1.316(3) |

**Table 5 Bond Angles for 25.**

| Atom | Atom | Atom | Angle/° | Atom | Atom | Atom | Angle/° |
|---|---|---|---|---|---|---|---|
| C008 | S001 | C00J | 100.71(9) | C00E | C009 | C007 | 121.28(16) |
| C006 | O003 | C00K | 109.15(15) | C00F | C00A | C00E | 120.05(17) |
| C006 | N005 | C00C | 112.98(16) | C00G | C00B | C007 | 113.74(15) |
| O004 | C006 | O003 | 120.86(19) | N005 | C00C | C00J | 111.14(16) |
| O004 | C006 | N005 | 129.5(2) | N005 | C00C | C00K | 100.19(15) |
| N005 | C006 | O003 | 109.64(17) | C00J | C00C | C00K | 114.14(16) |
| C009 | C007 | C00B | 111.19(15) | C00L | C00D | C009 | 120.93(15) |
| C009 | C007 | C00I | 111.14(14) | C00A | C00E | C009 | 120.86(18) |

**Table 5 Bond Angles for 25.**

| Atom | Atom | Atom | Angle/° | Atom | Atom | Atom | Angle/° |
|---|---|---|---|---|---|---|---|
| C00B | C007 | C00I | 110.97(15) | C00L | C00F | C00A | 119.53(17) |
| O002 | C008 | S001 | 123.31(15) | C00H | C00G | C00B | 124.39(18) |
| O002 | C008 | C00I | 123.18(17) | C008 | C00I | C007 | 110.12(15) |
| C00I | C008 | S001 | 113.50(13) | C00C | C00J | S001 | 110.98(13) |
| C00D | C009 | C007 | 120.43(16) | O003 | C00K | C00C | 105.02(15) |
| C00D | C009 | C00E | 118.29(16) | C00F | C00L | C00D | 120.33(17) |

**Table 6 Torsion Angles for 25.**

| A | B | C | D | Angle/° | A | B | C | D | Angle/° |
|---|---|---|---|---|---|---|---|---|---|
| S001 | C008 | C00I | C007 | -132.98(13) | C00B | C007 | C00I | C008 | 177.07(14) |
| O002 | C008 | C00I | C007 | 46.3(2) | C00C | N005 | C006 | O003 | 6.8(2) |
| N005 | C00C | C00J | S001 | 172.11(13) | C00C | N005 | C006 | O004 | -174.07(19) |
| N005 | C00C | C00K | O003 | 16.72(18) | C00D | C009 | C00E | C00A | -0.7(3) |
| C006 | O003 | C00K | C00C | -14.3(2) | C00E | C009 | C00D | C00L | 0.1(3) |
| C006 | N005 | C00C | C00J | -135.85(17) | C00E | C00A | C00F | C00L | 0.2(3) |
| C006 | N005 | C00C | C00K | -14.9(2) | C00F | C00A | C00E | C009 | 0.6(3) |
| C007 | C009 | C00D | C00L | -179.42(18) | C00I | C007 | C009 | C00D | -120.79(18) |
| C007 | C009 | C00E | C00A | 178.77(17) | C00I | C007 | C009 | C00E | 59.8(2) |
| C007 | C00B | C00G | C00H | -130.8(2) | C00I | C007 | C00B | C00G | 62.30(19) |
| C008 | S001 | C00J | C00C | -110.84(14) | C00J | S001 | C008 | O002 | -3.71(19) |
| C009 | C007 | C00B | C00G | -173.46(15) | C00J | S001 | C008 | C00I | 175.58(13) |
| C009 | C007 | C00I | C008 | 52.80(19) | C00J | C00C | C00K | O003 | 135.54(17) |
| C009 | C00D | C00L | C00F | 0.7(3) | C00K | O003 | C006 | O004 | -173.87(17) |
| C00A | C00F | C00L | C00D | -0.9(3) | C00K | O003 | C006 | N005 | 5.3(2) |
| C00B | C007 | C009 | C00D | 115.06(19) | C00K | C00C | C00J | S001 | 59.7(2) |
| C00B | C007 | C009 | C00E | -64.4(2) | | | | | |

**Table 7 Hydrogen Atom Coordinates (Å×10$^4$) and Isotropic Displacement Parameters (Å$^2$×10$^3$) for 25.**

| Atom | *x* | *y* | *z* | U(eq) |
|---|---|---|---|---|
| H005 | 10275.56 | 7934.16 | 9551.22 | 33 |
| H007 | 4889.61 | 4354.31 | 5412.95 | 24 |
| H00A | 650 | 3040.47 | 8552.17 | 31 |
| H00B | 1375.7 | 2964.7 | 5086.8 | 27 |
| H00C | -225.76 | 4244.92 | 5474.97 | 27 |
| H00D | 5798.83 | 7633.08 | 9459.22 | 29 |

**Table 7 Hydrogen Atom Coordinates (Å×$10^4$) and Isotropic Displacement Parameters (Å$^2$×$10^3$) for 25.**

| Atom | *x* | *y* | *z* | U(eq) |
|---|---|---|---|---|
| H00E | 6749.72 | 2742.28 | 6409.81 | 27 |
| H00F | 414.26 | 4382.83 | 7195.12 | 27 |
| H00G | 3950.44 | 1554.68 | 8844.72 | 31 |
| H00H | 2604.41 | 4692.62 | 3869.49 | 28 |
| H00K | 3320.13 | 6699.16 | 5353.04 | 26 |
| H00L | 1688.57 | 6396.62 | 6228.47 | 26 |
| H00M | 8431.22 | 7573.28 | 7767.47 | 35 |
| H00N | 7416.92 | 8999.22 | 8242.29 | 35 |
| H00O | 4904.63 | 5321.78 | 9136.57 | 33 |
| H00P | 6851.73 | 5223.3 | 8336.38 | 33 |
| H00Q | 7015.54 | 1437.65 | 7776.97 | 31 |
| H00J | -690(40) | 5680(30) | 3255(19) | 32(6) |
| H00I | -2190(60) | 5330(30) | 4200(20) | 46(7) |

CCDC 2286532 contains the supplementary crystallographic data for this paper, including structure factors and refinement instructions. These data can be obtained free of charge from The Cambridge Crystallographic Data Centre, 12 Union Road.

c) Crystal data of **27**

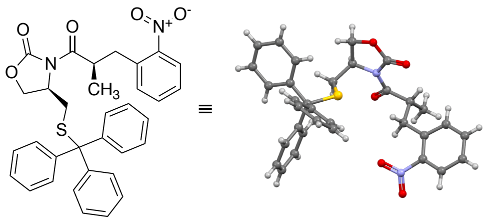


Table 1 Crystal data and structure refinement for **27**

| | |
|---|---|
| Identification code | **27** |
| Empirical formula | $C_{33}H_{30}N_2O_5S$ |
| Formula weight | 566.65 |
| Temperature/K | 230 |
| Crystal system | monoclinic |
| Space group | C2 |
| a/Å | 24.6329(16) |
| b/Å | 8.4402(5) |
| c/Å | 14.6479(9) |
| α/° | 90 |
| β/° | 113.287(2) |
| γ/° | 90 |
| Volume/Å$^3$ | 2797.3(3) |
| Z | 4 |
| $\rho_{calc}$g/cm$^3$ | 1.345 |
| μ/mm$^{-1}$ | 1.405 |
| F(000) | 1192.0 |
| Crystal size/mm$^3$ | 0.2 × 0.03 × 0.03 |
| Radiation | CuKα (λ = 1.54178) |
| 2Θ range for data collection/° | 6.57 to 132.856 |
| Index ranges | $-29 \leq h \leq 27$, $-9 \leq k \leq 9$, $-16 \leq l \leq 17$ |
| Reflections collected | 18035 |
| Independent reflections | 4660 [$R_{int}$ = 0.0367, $R_{sigma}$ = 0.0471] |
| Data/restraints/parameters | 4660/1/371 |
| Goodness-of-fit on F$^2$ | 0.994 |
| Final R indexes [I>=2σ (I)] | $R_1$ = 0.0283, $wR_2$ = 0.0756 |
| Final R indexes [all data] | $R_1$ = 0.0307, $wR_2$ = 0.0773 |
| Largest diff. peak/hole / e Å$^{-3}$ | 0.17/-0.23 |
| Flack parameter | 0.088(4) |

**Table 2 Fractional Atomic Coordinates (×10[4]) and Equivalent Isotropic Displacement Parameters (Å[2]×10[3]) for 27. $U_{eq}$ is defined as 1/3 of the trace of the orthogonalised $U_{IJ}$ tensor.**

| **Atom** | ***x*** | ***y*** | ***z*** | **U(eq)** |
|---|---|---|---|---|
| S001 | 6162.0(2) | 4274.1(5) | 7427.4(3) | 32.35(14) |
| O002 | 6002.9(7) | 577(2) | 8474.7(13) | 47.3(4) |
| O003 | 5390.4(7) | 1705(3) | 9056.5(13) | 55.4(5) |
| O004 | 4489.7(7) | 2730(2) | 6016.7(12) | 49.8(4) |
| O005 | 4963.5(9) | 8080(2) | 7717.3(15) | 60.7(5) |
| N006 | 5245.0(7) | 1920.1(19) | 7388.4(12) | 33.4(4) |
| N007 | 4529.8(9) | 8430(2) | 7887.8(16) | 50.1(5) |
| C008 | 7096.8(8) | 4083(2) | 6820.3(14) | 32.3(4) |
| C009 | 5523.2(9) | 1437(3) | 8370.9(18) | 38.3(5) |
| C00A | 6742.0(8) | 5304(2) | 7123.2(14) | 29.8(4) |
| O00B | 4228.7(11) | 9598(2) | 7541.1(18) | 75.7(6) |
| C00C | 7134.6(9) | 6119(2) | 8105.5(14) | 31.6(4) |
| C00D | 6467.0(8) | 6591(2) | 6323.3(14) | 32.9(4) |
| C00E | 4397.5(9) | 5787(3) | 8523.2(17) | 40.3(5) |
| C00F | 4365.0(9) | 3225(3) | 7531.7(17) | 37.8(5) |
| C00G | 4681.8(9) | 2595(2) | 6916.9(16) | 34.9(5) |
| C00H | 6765.0(11) | 7990(3) | 6352.0(18) | 47.1(5) |
| C00I | 4371.5(9) | 7437(3) | 8562.6(17) | 41.3(5) |
| C00J | 7177.2(10) | 4195(3) | 5942.8(17) | 45.6(5) |
| C00K | 6879.8(9) | 6997(3) | 8639.4(16) | 40.2(5) |
| C00L | 5815.1(10) | 2864(3) | 6421.5(16) | 38.5(5) |
| C00M | 4207.3(10) | 8248(3) | 9239(2) | 54.2(7) |
| C00N | 4584.5(11) | 4942(3) | 7785.9(19) | 43.4(5) |
| C00O | 6524.9(13) | 9154(3) | 5633(2) | 55.2(6) |
| C00P | 5991.4(13) | 8944(3) | 4880(2) | 59.4(7) |
| C00Q | 7225.1(11) | 7841(3) | 9477.1(19) | 52.9(6) |
| C00R | 7748.4(9) | 6119(3) | 8440.9(17) | 41.3(5) |
| C00S | 5581.9(9) | 1448(3) | 6797.7(17) | 37.6(5) |
| C00T | 7832.2(11) | 7818(3) | 9806.6(19) | 55.3(7) |
| C00U | 3697.5(10) | 3122(3) | 6946(2) | 55.5(6) |
| C00V | 7378.4(9) | 2849(3) | 7471.6(16) | 40.3(5) |
| C00W | 4243.5(11) | 4987(3) | 9208(2) | 49.3(6) |
| C00X | 7741.9(10) | 1802(3) | 7255(2) | 54.4(7) |
| C00Y | 4069.3(11) | 7392(4) | 9918(2) | 58.2(7) |
| C00Z | 6053.5(11) | 397(3) | 7538(2) | 49.1(6) |
| C010 | 8090.4(10) | 6963(3) | 9292.6(18) | 51.7(6) |
| C011 | 7828.7(12) | 1957(4) | 6387(2) | 63.1(8) |
| C012 | 4083.6(12) | 5772(4) | 9896(2) | 56.7(7) |

**Table 2 Fractional Atomic Coordinates (×10[4]) and Equivalent Isotropic Displacement Parameters (Å[2]×10[3]) for 27. $U_{eq}$ is defined as 1/3 of the trace of the orthogonalised $U_{IJ}$ tensor.**

| Atom | *x* | *y* | *z* | U(eq) |
|---|---|---|---|---|
| C013 | 7540.4(13) | 3121(4) | 5730(2) | 62.9(7) |
| C014 | 5698.4(15) | 7559(5) | 4827(3) | 93.2(14) |
| C015 | 5932.0(13) | 6402(4) | 5540(3) | 83.7(12) |

**Table 3 Anisotropic Displacement Parameters (Å[2]×10[3]) for 27. The Anisotropic displacement factor exponent takes the form: $-2\pi^2[h^2a^{*2}U_{11}+2hka^*b^*U_{12}+\ldots]$.**

| Atom | $U_{11}$ | $U_{22}$ | $U_{33}$ | $U_{23}$ | $U_{13}$ | $U_{12}$ |
|---|---|---|---|---|---|---|
| S001 | 36.2(2) | 32.8(3) | 31.3(2) | -2.09(18) | 16.90(18) | -4.32(17) |
| O002 | 38.8(8) | 47.7(10) | 54.8(10) | 9.0(7) | 18.0(7) | 8.3(6) |
| O003 | 49.9(9) | 76.7(13) | 40.0(9) | 4.7(8) | 18.0(8) | 13.7(9) |
| O004 | 52.5(9) | 50.9(10) | 39.8(9) | 0.5(7) | 11.6(7) | 5.4(7) |
| O005 | 69.7(11) | 48.4(10) | 74.4(13) | -8.1(9) | 39.5(10) | -14.6(9) |
| N006 | 34.8(8) | 29.0(8) | 39.1(9) | 1.2(7) | 17.5(7) | 0.2(6) |
| N007 | 56.3(12) | 32.6(11) | 58.2(13) | -8.0(8) | 19.3(10) | -8.4(8) |
| C008 | 30.6(9) | 33.9(11) | 31.7(10) | -3.9(8) | 11.7(7) | -1.0(7) |
| C009 | 32.8(9) | 36.8(11) | 43.1(13) | 2.8(9) | 12.6(9) | -0.4(8) |
| C00A | 31.3(9) | 29.2(10) | 29.5(10) | 0.6(7) | 12.7(8) | 0.4(7) |
| O00B | 96.2(15) | 36.5(12) | 92.4(15) | 7.8(9) | 35.0(12) | 4.5(9) |
| C00C | 35.7(10) | 29.6(10) | 29.2(10) | 2.6(7) | 12.3(8) | -1.8(7) |
| C00D | 39.1(10) | 31.4(10) | 31.7(10) | 3.5(8) | 17.9(8) | 5.2(8) |
| C00E | 34.9(11) | 38.1(12) | 47.5(13) | -2.8(9) | 15.9(9) | -0.2(8) |
| C00F | 37.9(10) | 29.9(11) | 45.2(12) | -0.3(8) | 16.2(9) | 1.7(8) |
| C00G | 37.3(10) | 25.1(10) | 40.0(12) | -1.0(8) | 12.7(9) | -1.2(7) |
| C00H | 53.4(12) | 40.9(12) | 47.1(13) | 6.7(10) | 20.0(10) | -2.7(10) |
| C00I | 38.0(11) | 37.5(12) | 46.9(13) | -8.2(9) | 15.0(10) | -2.4(8) |
| C00J | 53.8(12) | 48.7(13) | 40.3(11) | -0.1(10) | 25.0(9) | 7.4(10) |
| C00K | 39.3(10) | 43.9(12) | 37.8(11) | -6.9(9) | 15.8(9) | -1.9(9) |
| C00L | 45.8(11) | 37.2(12) | 35.5(11) | -5.5(9) | 19.2(9) | -9.2(8) |
| C00M | 44.3(12) | 50.2(15) | 65.9(16) | -17.9(12) | 19.4(11) | 0.2(10) |
| C00N | 52.3(13) | 30.4(11) | 54.2(14) | -2.9(9) | 28.2(11) | -3.2(9) |
| C00O | 78.1(17) | 39.5(13) | 56.6(14) | 11.8(11) | 35.9(13) | 1.7(12) |
| C00P | 72.5(17) | 50.0(16) | 62.1(16) | 28.7(12) | 33.3(14) | 22.0(12) |
| C00Q | 57.1(14) | 57.9(16) | 47.6(14) | -17.7(11) | 25.0(11) | -9.2(11) |
| C00R | 37.4(10) | 45.7(13) | 40.9(12) | -4.1(9) | 15.5(9) | -4.2(8) |
| C00S | 42.0(10) | 30.4(10) | 44.8(12) | -6.0(8) | 21.9(9) | -3.2(8) |
| C00T | 55.7(14) | 63.9(16) | 40.9(13) | -17.6(11) | 13.2(11) | -19.9(12) |

**Table 3 Anisotropic Displacement Parameters ($Å^2 \times 10^3$) for 27. The Anisotropic displacement factor exponent takes the form: $-2\pi^2[h^2a^{*2}U_{11}+2hka^*b^*U_{12}+\ldots]$.**

| Atom | $U_{11}$ | $U_{22}$ | $U_{33}$ | $U_{23}$ | $U_{13}$ | $U_{12}$ |
|---|---|---|---|---|---|---|
| C00U | 39.5(12) | 49.1(14) | 72.2(17) | -5.0(12) | 16.2(11) | 4.4(10) |
| C00V | 41.1(11) | 41.6(12) | 35.2(11) | 1.2(8) | 11.9(9) | 8.1(9) |
| C00W | 52.9(13) | 43.6(13) | 56.6(15) | 2.2(10) | 27.0(11) | -0.1(9) |
| C00X | 45.9(12) | 52.3(15) | 55.0(15) | -6.2(11) | 9.2(11) | 18.0(11) |
| C00Y | 45.6(13) | 74.2(19) | 58.6(16) | -24.1(13) | 24.6(12) | -2.6(11) |
| C00Z | 53.0(13) | 36.9(13) | 68.2(17) | 6.2(11) | 35.5(12) | 9.5(9) |
| C010 | 38.1(11) | 62.5(16) | 49.0(14) | -6.8(12) | 11.2(10) | -12.0(10) |
| C011 | 52.2(14) | 70.5(19) | 67.5(18) | -15.8(14) | 24.5(13) | 19.7(13) |
| C012 | 52.2(14) | 74(2) | 50.7(15) | -3.2(12) | 28.1(12) | -1.3(12) |
| C013 | 70.3(16) | 76(2) | 55.5(16) | -10.0(14) | 39.1(14) | 11.3(14) |
| C014 | 67.6(18) | 93(3) | 82(2) | 49(2) | -10.9(16) | -10.4(17) |
| C015 | 65.6(16) | 69.5(19) | 77(2) | 40.2(16) | -13.1(15) | -22.3(15) |

**Table 4 Bond Lengths for 27.**

| Atom | Atom | Length/Å | Atom | Atom | Length/Å |
|---|---|---|---|---|---|
| S001 | C00A | 1.870(2) | C00E | C00W | 1.381(4) |
| S001 | C00L | 1.823(2) | C00F | C00G | 1.503(3) |
| O002 | C009 | 1.343(3) | C00F | C00N | 1.540(3) |
| O002 | C00Z | 1.435(3) | C00F | C00U | 1.527(3) |
| O003 | C009 | 1.195(3) | C00H | C00O | 1.390(3) |
| O004 | C00G | 1.217(3) | C00I | C00M | 1.389(3) |
| O005 | N007 | 1.226(3) | C00J | C013 | 1.392(4) |
| N006 | C009 | 1.388(3) | C00K | C00Q | 1.382(3) |
| N006 | C00G | 1.403(3) | C00L | C00S | 1.521(3) |
| N006 | C00S | 1.471(3) | C00M | C00Y | 1.376(4) |
| N007 | O00B | 1.218(3) | C00O | C00P | 1.352(4) |
| N007 | C00I | 1.462(3) | C00P | C014 | 1.360(5) |
| C008 | C00A | 1.526(3) | C00Q | C00T | 1.378(4) |
| C008 | C00J | 1.379(3) | C00R | C010 | 1.394(3) |
| C008 | C00V | 1.398(3) | C00S | C00Z | 1.522(3) |
| C00A | C00C | 1.542(3) | C00T | C010 | 1.368(4) |
| C00A | C00D | 1.544(3) | C00V | C00X | 1.382(3) |
| C00C | C00K | 1.394(3) | C00W | C012 | 1.386(4) |
| C00C | C00R | 1.393(3) | C00X | C011 | 1.376(4) |
| C00D | C00H | 1.382(3) | C00Y | C012 | 1.369(4) |
| C00D | C015 | 1.372(3) | C011 | C013 | 1.362(4) |
| C00E | C00I | 1.396(3) | C014 | C015 | 1.379(4) |

**Table 4 Bond Lengths for 27.**

| Atom | Atom | Length/Å | Atom | Atom | Length/Å |
|---|---|---|---|---|---|
| C00E | C00N | 1.510(3) | | | |

**Table 5 Bond Angles for 27.**

| Atom | Atom | Atom | Angle/˚ | Atom | Atom | Atom | Angle/˚ |
|---|---|---|---|---|---|---|---|
| C00L | S001 | C00A | 105.60(10) | O004 | C00G | N006 | 117.1(2) |
| C009 | O002 | C00Z | 110.75(18) | O004 | C00G | C00F | 123.01(19) |
| C009 | N006 | C00G | 128.06(19) | N006 | C00G | C00F | 119.69(18) |
| C009 | N006 | C00S | 111.58(17) | C00D | C00H | C00O | 121.3(2) |
| C00G | N006 | C00S | 119.90(18) | C00E | C00I | N007 | 121.0(2) |
| O005 | N007 | C00I | 119.1(2) | C00M | C00I | N007 | 115.5(2) |
| O00B | N007 | O005 | 123.1(2) | C00M | C00I | C00E | 123.5(2) |
| O00B | N007 | C00I | 117.7(2) | C008 | C00J | C013 | 120.4(2) |
| C00J | C008 | C00A | 122.32(18) | C00Q | C00K | C00C | 121.1(2) |
| C00J | C008 | C00V | 118.1(2) | C00S | C00L | S001 | 110.11(15) |
| C00V | C008 | C00A | 119.45(18) | C00Y | C00M | C00I | 118.8(2) |
| O002 | C009 | N006 | 109.1(2) | C00E | C00N | C00F | 116.1(2) |
| O003 | C009 | O002 | 121.9(2) | C00P | C00O | C00H | 120.7(2) |
| O003 | C009 | N006 | 129.0(2) | C00O | C00P | C014 | 118.8(2) |
| C008 | C00A | S001 | 109.55(13) | C00T | C00Q | C00K | 120.4(2) |
| C008 | C00A | C00C | 110.64(15) | C010 | C00R | C00C | 120.2(2) |
| C008 | C00A | C00D | 112.60(16) | N006 | C00S | C00L | 112.44(18) |
| C00C | C00A | S001 | 103.96(13) | N006 | C00S | C00Z | 100.86(18) |
| C00C | C00A | C00D | 108.63(15) | C00Z | C00S | C00L | 115.15(18) |
| C00D | C00A | S001 | 111.13(12) | C010 | C00T | C00Q | 119.4(2) |
| C00K | C00C | C00A | 120.35(17) | C00X | C00V | C008 | 120.8(2) |
| C00R | C00C | C00A | 121.46(19) | C00E | C00W | C012 | 122.2(3) |
| C00R | C00C | C00K | 117.95(19) | C00V | C00X | C011 | 120.2(2) |
| C00H | C00D | C00A | 120.64(17) | C012 | C00Y | C00M | 119.4(3) |
| C015 | C00D | C00A | 122.7(2) | O002 | C00Z | C00S | 106.80(18) |
| C015 | C00D | C00H | 116.6(2) | C00T | C010 | C00R | 120.9(2) |
| C00I | C00E | C00N | 122.2(2) | C013 | C011 | C00X | 119.5(2) |
| C00W | C00E | C00I | 115.3(2) | C00Y | C012 | C00W | 120.8(3) |
| C00W | C00E | C00N | 122.5(2) | C011 | C013 | C00J | 120.9(3) |
| C00G | C00F | C00N | 105.16(18) | C00P | C014 | C015 | 120.8(3) |
| C00G | C00F | C00U | 110.02(19) | C00D | C015 | C014 | 121.7(3) |
| C00U | C00F | C00N | 112.73(19) | | | | |

**Table 6 Torsion Angles for 27.**

| A | B | C | D | Angle/° | A | B | C | D | Angle/° |
|---|---|---|---|---|---|---|---|---|---|
| S001 | C00A | C00C | C00K | -47.7(2) | C00H | C00D | C015 | C014 | -1.1(6) |
| S001 | C00A | C00C | C00R | 137.98(18) | C00H | C00O | C00P | C014 | -1.3(5) |
| S001 | C00A | C00D | C00H | 144.39(19) | C00I | C00E | C00N | C00F | -156.4(2) |
| S001 | C00A | C00D | C015 | -37.6(3) | C00I | C00E | C00W | C012 | -1.0(3) |
| S001 | C00L | C00S | N006 | 47.1(2) | C00I | C00M | C00Y | C012 | -1.5(4) |
| S001 | C00L | C00S | C00Z | -67.6(2) | C00J | C008 | C00A | S001 | 127.04(19) |
| O005 | N007 | C00I | C00E | -41.9(3) | C00J | C008 | C00A | C00C | -118.9(2) |
| O005 | N007 | C00I | C00M | 137.1(2) | C00J | C008 | C00A | C00D | 2.8(3) |
| N006 | C00S | C00Z | O002 | -9.0(2) | C00J | C008 | C00V | C00X | 2.0(3) |
| N007 | C00I | C00M | C00Y | -178.1(2) | C00K | C00C | C00R | C010 | 0.8(3) |
| C008 | C00A | C00C | C00K | -165.19(19) | C00K | C00Q | C00T | C010 | 0.6(4) |
| C008 | C00A | C00C | C00R | 20.5(3) | C00L | S001 | C00A | C008 | -46.35(15) |
| C008 | C00A | C00D | C00H | -92.3(2) | C00L | S001 | C00A | C00C | -164.62(13) |
| C008 | C00A | C00D | C015 | 85.7(3) | C00L | S001 | C00A | C00D | 78.71(15) |
| C008 | C00J | C013 | C011 | -0.9(4) | C00L | C00S | C00Z | O002 | 112.3(2) |
| C008 | C00V | C00X | C011 | -0.4(4) | C00M | C00Y | C012 | C00W | 1.0(4) |
| C009 | O002 | C00Z | C00S | 7.4(2) | C00N | C00E | C00I | N007 | -0.4(3) |
| C009 | N006 | C00G | O004 | -170.02(19) | C00N | C00E | C00I | C00M | -179.3(2) |
| C009 | N006 | C00G | C00F | 14.5(3) | C00N | C00E | C00W | C012 | 178.7(2) |
| C009 | N006 | C00S | C00L | -115.0(2) | C00N | C00F | C00G | O004 | -88.6(2) |
| C009 | N006 | C00S | C00Z | 8.2(2) | C00N | C00F | C00G | N006 | 86.5(2) |
| C00A | S001 | C00L | C00S | 151.20(14) | C00O | C00P | C014 | C015 | 1.7(6) |
| C00A | C008 | C00J | C013 | 175.2(2) | C00P | C014 | C015 | C00D | -0.5(7) |
| C00A | C008 | C00V | C00X | -174.7(2) | C00Q | C00T | C010 | C00R | 0.1(4) |
| C00A | C00C | C00K | C00Q | -174.7(2) | C00R | C00C | C00K | C00Q | -0.1(3) |
| C00A | C00C | C00R | C010 | 175.2(2) | C00S | N006 | C009 | O002 | -4.2(2) |
| C00A | C00D | C00H | C00O | 179.5(2) | C00S | N006 | C009 | O003 | 175.0(2) |
| C00A | C00D | C015 | C014 | -179.1(4) | C00S | N006 | C00G | O004 | 1.6(3) |
| O00B | N007 | C00I | C00E | 140.0(2) | C00S | N006 | C00G | C00F | -173.91(17) |
| O00B | N007 | C00I | C00M | -41.0(3) | C00U | C00F | C00G | O004 | 33.0(3) |
| C00C | C00A | C00D | C00H | 30.6(3) | C00U | C00F | C00G | N006 | -151.81(19) |
| C00C | C00A | C00D | C015 | -151.4(3) | C00U | C00F | C00N | C00E | 68.3(3) |
| C00C | C00K | C00Q | C00T | -0.5(4) | C00V | C008 | C00A | S001 | -56.4(2) |
| C00C | C00R | C010 | C00T | -0.8(4) | C00V | C008 | C00A | C00C | 57.6(2) |
| C00D | C00A | C00C | C00K | 70.7(2) | C00V | C008 | C00A | C00D | 179.36(18) |
| C00D | C00A | C00C | C00R | -103.6(2) | C00V | C008 | C00J | C013 | -1.4(3) |
| C00D | C00H | C00O | C00P | -0.3(4) | C00V | C00X | C011 | C013 | -1.9(4) |
| C00E | C00I | C00M | C00Y | 0.9(3) | C00W | C00E | C00I | N007 | 179.26(19) |
| C00E | C00W | C012 | C00Y | 0.3(4) | C00W | C00E | C00I | C00M | 0.4(3) |
| C00G | N006 | C009 | O002 | 167.96(18) | C00W | C00E | C00N | C00F | 23.9(3) |

**Table 6 Torsion Angles for 27.**

| A | B | C | D | Angle/° | A | B | C | D | Angle/° |
|---|---|---|---|---|---|---|---|---|---|
| C00G | N006 | C009 | O003 | -12.8(4) | C00X | C011 | C013 | C00J | 2.6(5) |
| C00G | N006 | C00S | C00L | 72.1(2) | C00Z | O002 | C009 | O003 | 178.5(2) |
| C00G | N006 | C00S | C00Z | -164.72(18) | C00Z | O002 | C009 | N006 | -2.2(2) |
| C00G | C00F | C00N | C00E | -171.86(18) | C015 | C00D | C00H | C00O | 1.4(4) |

**Table 7 Hydrogen Atom Coordinates (Å×10[4]) and Isotropic Displacement Parameters (Å[2]×10[3]) for 27.**

| Atom | *x* | *y* | *z* | U(eq) |
|---|---|---|---|---|
| H00F | 4477.22 | 2605.19 | 8144.99 | 45 |
| H00H | 7133.23 | 8156.05 | 6862.2 | 57 |
| H00J | 6987.92 | 4992.56 | 5490.49 | 55 |
| H00K | 6470.69 | 7016.35 | 8428.52 | 48 |
| H00A | 5492.15 | 3375.38 | 5885.94 | 46 |
| H00B | 6101.3 | 2510.17 | 6162.92 | 46 |
| H00M | 4191.02 | 9348.84 | 9232.53 | 65 |
| H00C | 5012.93 | 4935.47 | 8046.43 | 52 |
| H00D | 4445.27 | 5550.96 | 7174.65 | 52 |
| H00O | 6733.36 | 10086.79 | 5670.07 | 66 |
| H00P | 5827.75 | 9733.37 | 4407.18 | 71 |
| H00Q | 7046.76 | 8427.61 | 9820.07 | 63 |
| H00R | 7930.63 | 5554.02 | 8095.3 | 50 |
| H00S | 5329.53 | 806.33 | 6231.11 | 45 |
| H00T | 8064.31 | 8378.47 | 10373.45 | 66 |
| H00E | 3588.17 | 3736 | 6348.28 | 83 |
| H00G | 3498.7 | 3529.25 | 7343.37 | 83 |
| H00I | 3586.05 | 2036.68 | 6778.89 | 83 |
| H00V | 7320.51 | 2731.36 | 8057.97 | 48 |
| H00W | 4247.23 | 3885.42 | 9208.36 | 59 |
| H00X | 7928.39 | 989.16 | 7696.26 | 65 |
| H00Y | 3967.22 | 7910.52 | 10387.52 | 70 |
| H00L | 5991.39 | -700.4 | 7323.97 | 59 |
| H00N | 6443.3 | 718.43 | 7593.03 | 59 |
| H010 | 8500.11 | 6944.85 | 9514.49 | 62 |
| H011 | 8082.52 | 1271.86 | 6249.87 | 76 |
| H012 | 3984.78 | 5190.82 | 10347.26 | 68 |
| H013 | 7586.62 | 3200.05 | 5131.67 | 75 |
| H014 | 5336.02 | 7389.78 | 4302.49 | 112 |
| H015 | 5721.39 | 5468.25 | 5489.17 | 100 |

CCDC 2286531 contains the supplementary crystallographic data for this paper, including structure factors and refinement instructions. These data can be obtained free of charge from The Cambridge Crystallographic Data Centre, 12 Union Road.

d) Crystal data of **29**

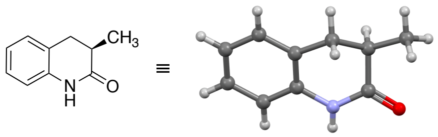


**Table 1 Crystal data and structure refinement for 29.**

| | |
|---|---|
| Identification code | **29** |
| Empirical formula | $C_{10}H_{11}NO$ |
| Formula weight | 161.20 |
| Temperature/K | 200 |
| Crystal system | monoclinic |
| Space group | $P2_1$ |
| a/Å | 7.2252(4) |
| b/Å | 11.5516(7) |
| c/Å | 10.3806(6) |
| α/° | 90 |
| β/° | 101.4620(10) |
| γ/° | 90 |
| Volume/Å$^3$ | 849.11(9) |
| Z | 4 |
| $\rho_{calc}$g/cm$^3$ | 1.261 |
| μ/mm$^{-1}$ | 0.652 |
| F(000) | 344.0 |
| Crystal size/mm$^3$ | 0.3 × 0.1 × 0.1 |
| Radiation | CuKα (λ = 1.54178) |
| 2Θ range for data collection/° | 14.672 to 132.748 |
| Index ranges | $-8 \le h \le 8$, $-13 \le k \le 13$, $-12 \le l \le 12$ |
| Reflections collected | 18929 |
| Independent reflections | 2891 [$R_{int}$ = 0.0221, $R_{sigma}$ = 0.0187] |
| Data/restraints/parameters | 2891/1/230 |
| Goodness-of-fit on F$^2$ | 0.908 |
| Final R indexes [I>=2σ (I)] | $R_1$ = 0.0298, $wR_2$ = 0.0923 |
| Final R indexes [all data] | $R_1$ = 0.0299, $wR_2$ = 0.0924 |
| Largest diff. peak/hole / e Å$^{-3}$ | 0.18/-0.20 |
| Flack parameter | 0.2(2) |

**Table 2 Fractional Atomic Coordinates (×10⁴) and Equivalent Isotropic Displacement Parameters (Å²×10³) for 29. $U_{eq}$ is defined as 1/3 of the trace of the orthogonalised $U_{IJ}$ tensor.**

| Atom | *x* | *y* | *z* | U(eq) |
|---|---|---|---|---|
| O001 | 6311.8(18) | 6232.8(10) | 7149.3(14) | 42.6(3) |
| O002 | 3319.2(18) | 3823.9(10) | 7485.3(13) | 43.0(3) |
| N003 | 4152(2) | 6138.9(12) | 8438.6(15) | 36.9(4) |
| N004 | 5765(2) | 3867.2(12) | 6441.9(14) | 35.2(3) |
| C005 | 3963(2) | 2069.7(14) | 6387.8(17) | 37.3(4) |
| C006 | 6989(2) | 3361.9(14) | 5706.3(16) | 33.7(4) |
| C007 | 5049(2) | 8374.3(14) | 9352.5(17) | 37.6(4) |
| C008 | 3339(2) | 7777.3(15) | 9666.9(16) | 34.6(4) |
| C009 | 1460(3) | 6032.5(15) | 9506.2(19) | 42.1(4) |
| C00A | 4318(2) | 3319.2(15) | 6831.4(16) | 35.2(4) |
| C00B | 5342(2) | 6728.3(14) | 7830.4(16) | 33.8(4) |
| C00C | 6998(2) | 2159.9(15) | 5552.2(16) | 35.4(4) |
| C00D | 7153(3) | 8553.7(15) | 7673.3(19) | 41.2(4) |
| C00E | 5365(2) | 8035.7(14) | 7986.9(16) | 34.4(4) |
| C00F | 5801(3) | 1460.0(15) | 6292.0(18) | 40.0(4) |
| C00G | 2962(2) | 6655.0(14) | 9204.1(16) | 33.5(4) |
| C00H | 2883(3) | 1431.7(18) | 7300(2) | 51.7(5) |
| C00I | 9331(3) | 2378.7(17) | 4191(2) | 44.8(5) |
| C00J | 8191(2) | 4060.5(15) | 5157.9(18) | 38.2(4) |
| C00K | 2168(2) | 8268.3(16) | 10434.0(18) | 39.6(4) |
| C00L | 8155(2) | 1690.4(16) | 4774.3(19) | 41.1(4) |
| C00M | 326(3) | 6532.3(17) | 10287(2) | 45.5(5) |
| C00N | 9357(2) | 3563.1(18) | 4398.7(19) | 43.9(4) |
| C00O | 678(3) | 7646.0(18) | 10748.0(19) | 44.1(4) |

**Table 3 Anisotropic Displacement Parameters (Å²×10³) for 29. The Anisotropic displacement factor exponent takes the form: $-2\pi^2[h^2a^{*2}U_{11}+2hka^*b^*U_{12}+\ldots]$.**

| Atom | $U_{11}$ | $U_{22}$ | $U_{33}$ | $U_{23}$ | $U_{13}$ | $U_{12}$ |
|---|---|---|---|---|---|---|
| O001 | 47.6(7) | 33.7(6) | 51.6(8) | -5.1(5) | 22.2(6) | 0.0(5) |
| O002 | 50.4(7) | 37.9(6) | 45.9(7) | -6.7(5) | 22.3(6) | -1.1(5) |
| N003 | 47.7(8) | 26.3(7) | 40.0(8) | -1.7(6) | 16.4(7) | 1.2(6) |
| N004 | 45.0(8) | 27.7(7) | 35.4(7) | -2.5(6) | 13.9(6) | 0.5(5) |
| C005 | 44.6(9) | 34.1(8) | 33.7(8) | -4.4(7) | 9.1(7) | -2.6(7) |
| C006 | 35.2(8) | 33.5(9) | 30.9(8) | -1.7(6) | 2.7(6) | 5.0(6) |
| C007 | 43.1(8) | 33.2(8) | 37.1(9) | -3.6(7) | 9.7(7) | -1.0(7) |
| C008 | 40.0(8) | 34.4(8) | 29.0(8) | 2.7(7) | 6.3(6) | 5.2(7) |

**Table 3 Anisotropic Displacement Parameters ($Å^2×10^3$) for 29. The Anisotropic displacement factor exponent takes the form: $-2\pi^2[h^2a^{*2}U_{11}+2hka^*b^*U_{12}+…]$.**

| Atom | $U_{11}$ | $U_{22}$ | $U_{33}$ | $U_{23}$ | $U_{13}$ | $U_{12}$ |
|---|---|---|---|---|---|---|
| C009 | 46.2(10) | 37.3(9) | 44.7(10) | -0.7(7) | 13.5(8) | -1.6(7) |
| C00A | 42.2(9) | 33.3(8) | 30.1(8) | -0.3(6) | 7.0(6) | 2.0(7) |
| C00B | 35.7(8) | 33.2(9) | 33.1(8) | -0.3(6) | 8.7(7) | 3.5(6) |
| C00C | 37.4(9) | 31.8(8) | 34.8(8) | 0.0(7) | 2.0(6) | 5.6(6) |
| C00D | 48.5(9) | 31.0(8) | 46.3(9) | 2.6(7) | 15.0(7) | -2.9(7) |
| C00E | 39.9(8) | 30.5(8) | 33.0(8) | 1.0(6) | 7.8(6) | 1.6(6) |
| C00F | 50.4(10) | 28.0(8) | 40.0(9) | -0.3(7) | 5.2(7) | 2.8(7) |
| C00G | 37.1(8) | 33.6(8) | 31.2(8) | 2.5(7) | 10.1(6) | 5.2(6) |
| C00H | 71.0(13) | 40.2(10) | 49.1(11) | -9.4(8) | 24.6(9) | -13.1(9) |
| C00I | 36.3(8) | 50.8(12) | 47.5(9) | -7.5(8) | 8.6(7) | 12.2(8) |
| C00J | 38.1(8) | 34.9(8) | 42.4(9) | -2.8(7) | 9.9(7) | 1.0(7) |
| C00K | 45.0(9) | 39.1(8) | 34.6(8) | -1.3(7) | 7.8(7) | 8.5(7) |
| C00L | 39.0(9) | 37.0(9) | 45.1(9) | -5.1(7) | 2.7(7) | 11.7(7) |
| C00M | 40.0(9) | 51.8(11) | 47.8(10) | 3.4(9) | 16.4(7) | 0.2(8) |
| C00N | 35.4(8) | 48.6(10) | 49.2(10) | -1.2(8) | 12.1(7) | 2.7(8) |
| C00O | 42.1(9) | 53.0(11) | 39.2(9) | 1.5(8) | 13.4(7) | 11.5(8) |

**Table 4 Bond Lengths for 29.**

| Atom | Atom | Length/Å | Atom | Atom | Length/Å |
|---|---|---|---|---|---|
| O001 | C00B | 1.2308(19) | C008 | C00G | 1.391(3) |
| O002 | C00A | 1.231(2) | C008 | C00K | 1.392(2) |
| N003 | C00B | 1.348(2) | C009 | C00G | 1.389(2) |
| N003 | C00G | 1.413(2) | C009 | C00M | 1.388(3) |
| N004 | C006 | 1.405(2) | C00B | C00E | 1.519(2) |
| N004 | C00A | 1.351(2) | C00C | C00F | 1.502(2) |
| C005 | C00A | 1.521(2) | C00C | C00L | 1.383(2) |
| C005 | C00F | 1.524(2) | C00D | C00E | 1.516(2) |
| C005 | C00H | 1.530(2) | C00I | C00L | 1.387(3) |
| C006 | C00C | 1.398(2) | C00I | C00N | 1.385(3) |
| C006 | C00J | 1.388(2) | C00J | C00N | 1.387(2) |
| C007 | C008 | 1.506(2) | C00K | C00O | 1.386(3) |
| C007 | C00E | 1.531(2) | C00M | C00O | 1.378(3) |

**Table 5 Bond Angles for 29.**

| Atom | Atom | Atom | Angle/° | Atom | Atom | Atom | Angle/° |
|---|---|---|---|---|---|---|---|
| C00B | N003 | C00G | 124.49(14) | N003 | C00B | C00E | 116.51(13) |
| C00A | N004 | C006 | 125.18(14) | C006 | C00C | C00F | 117.38(15) |
| C00A | C005 | C00F | 111.19(14) | C00L | C00C | C006 | 118.25(16) |
| C00A | C005 | C00H | 110.23(14) | C00L | C00C | C00F | 124.32(16) |
| C00F | C005 | C00H | 112.08(17) | C00B | C00E | C007 | 110.65(13) |
| C00C | C006 | N004 | 119.50(15) | C00D | C00E | C007 | 112.84(14) |
| C00J | C006 | N004 | 119.61(14) | C00D | C00E | C00B | 111.16(13) |
| C00J | C006 | C00C | 120.88(15) | C00C | C00F | C005 | 112.49(15) |
| C008 | C007 | C00E | 111.32(14) | C008 | C00G | N003 | 119.23(14) |
| C00G | C008 | C007 | 117.68(14) | C009 | C00G | N003 | 119.91(15) |
| C00G | C008 | C00K | 118.56(15) | C009 | C00G | C008 | 120.85(15) |
| C00K | C008 | C007 | 123.72(16) | C00N | C00I | C00L | 119.26(17) |
| C00M | C009 | C00G | 119.67(17) | C00N | C00J | C006 | 119.48(17) |
| O002 | C00A | N004 | 121.07(15) | C00O | C00K | C008 | 120.77(17) |
| O002 | C00A | C005 | 122.30(15) | C00C | C00L | C00I | 121.57(17) |
| N004 | C00A | C005 | 116.61(14) | C00O | C00M | C009 | 120.10(17) |
| O001 | C00B | N003 | 121.54(14) | C00I | C00N | C00J | 120.46(18) |
| O001 | C00B | C00E | 121.91(14) | C00M | C00O | C00K | 120.04(16) |

**Table 6 Torsion Angles for 29.**

| A | B | C | D | Angle/° | A | B | C | D | Angle/° |
|---|---|---|---|---|---|---|---|---|---|
| O001 | C00B | C00E | C007 | -147.64(16) | C00B | N003 | C00G | C009 | 163.43(16) |
| O001 | C00B | C00E | C00D | -21.4(2) | C00C | C006 | C00J | C00N | 2.9(3) |
| N003 | C00B | C00E | C007 | 34.7(2) | C00E | C007 | C008 | C00G | 35.5(2) |
| N003 | C00B | C00E | C00D | 160.89(15) | C00E | C007 | C008 | C00K | -147.19(16) |
| N004 | C006 | C00C | C00F | -5.3(2) | C00F | C005 | C00A | O002 | -148.98(16) |
| N004 | C006 | C00C | C00L | 177.13(14) | C00F | C005 | C00A | N004 | 32.7(2) |
| N004 | C006 | C00J | C00N | -178.03(15) | C00F | C00C | C00L | C00I | -175.27(17) |
| C006 | N004 | C00A | O002 | 179.36(15) | C00G | N003 | C00B | O001 | -178.18(15) |
| C006 | N004 | C00A | C005 | -2.3(2) | C00G | N003 | C00B | C00E | -0.5(2) |
| C006 | C00C | C00F | C005 | 35.7(2) | C00G | C008 | C00K | C00O | 1.0(2) |
| C006 | C00C | C00L | C00I | 2.1(3) | C00G | C009 | C00M | C00O | 0.7(3) |
| C006 | C00J | C00N | C00I | -0.3(3) | C00H | C005 | C00A | O002 | -24.1(2) |
| C007 | C008 | C00G | N003 | -1.7(2) | C00H | C005 | C00A | N004 | 157.65(16) |
| C007 | C008 | C00G | C009 | 177.07(16) | C00H | C005 | C00F | C00C | -172.11(13) |
| C007 | C008 | C00K | C00O | -176.30(16) | C00J | C006 | C00C | C00F | 173.82(14) |
| C008 | C007 | C00E | C00B | -50.70(18) | C00J | C006 | C00C | C00L | -3.8(3) |
| C008 | C007 | C00E | C00D | -175.97(14) | C00K | C008 | C00G | N003 | -179.17(15) |

**Table 6 Torsion Angles for 29.**

| A | B | C | D | Angle/° | A | B | C | D | Angle/° |
|---|---|---|---|---|---|---|---|---|---|
| C008 | C00K | C00O | C00M | -0.8(3) | C00K | C008 | C00G | C009 | -0.4(2) |
| C009 | C00M | C00O | C00K | -0.1(3) | C00L | C00C | C00F | C005 | -146.90(17) |
| C00A | N004 | C006 | C00C | -12.9(2) | C00L | C00I | C00N | C00J | -1.4(3) |
| C00A | N004 | C006 | C00J | 167.99(16) | C00M | C009 | C00G | N003 | 178.29(15) |
| C00A | C005 | C00F | C00C | -48.23(19) | C00M | C009 | C00G | C008 | -0.4(3) |
| C00B | N003 | C00G | C008 | -17.8(2) | C00N | C00I | C00L | C00C | 0.4(3) |

**Table 7 Hydrogen Atom Coordinates (Å×10$^4$) and Isotropic Displacement Parameters (Å$^2$×10$^3$) for 29.**

| Atom | *x* | *y* | *z* | U(eq) |
|---|---|---|---|---|
| H005 | 3146.68 | 2079.55 | 5488.8 | 45 |
| H00A | 6176.89 | 8162.26 | 10020.22 | 45 |
| H00B | 4879.08 | 9223.19 | 9389.64 | 45 |
| H009 | 1209.95 | 5268.28 | 9179.75 | 51 |
| H00C | 7189.99 | 9385.04 | 7864.95 | 62 |
| H00D | 7167.47 | 8434.15 | 6740.78 | 62 |
| H00E | 8256.1 | 8176.69 | 8211.64 | 62 |
| H00F | 4278.51 | 8349.64 | 7330.06 | 41 |
| H00G | 6526.65 | 1305.56 | 7189.36 | 48 |
| H00H | 5505.41 | 705.79 | 5845.74 | 48 |
| H00I | 3596.93 | 1475.47 | 8205.81 | 78 |
| H00J | 2719.12 | 618.81 | 7031.12 | 78 |
| H00L | 1642.54 | 1792.17 | 7246.19 | 78 |
| H00N | 10110.57 | 2041.17 | 3655.6 | 54 |
| H00P | 8215.47 | 4873.12 | 5301.3 | 46 |
| H00K | 2393.45 | 9038.8 | 10745.93 | 47 |
| H00Q | 8144.7 | 877.27 | 4635.97 | 49 |
| H00M | -693.62 | 6106.23 | 10504.62 | 55 |
| H00R | 10177.95 | 4038.45 | 4018.39 | 53 |
| H00O | -101.23 | 7987.2 | 11281.05 | 53 |
| H004 | 5990(30) | 4650(20) | 6670(30) | 51(6) |
| H003 | 3930(30) | 5460(20) | 8230(20) | 40(5) |

CCDC 2286539 contains the supplementary crystallographic data for this paper, including structure factors and refinement instructions. These data can be obtained free of charge from The Cambridge Crystallographic Data Centre, 12 Union Road.

e) Crystal data for **S5**

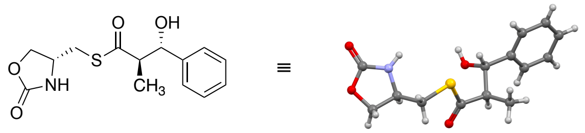

**Table 1 Crystal data and structure refinement for S5.**

| | |
|---|---|
| Identification code | **S5** |
| Empirical formula | $C_{2.95}H_{3.58}N_{0.21}O_{0.84}S_{0.21}$ |
| Formula weight | 62.18 |
| Temperature/K | 150 |
| Crystal system | monoclinic |
| Space group | C2 |
| a/Å | 27.4096(11) |
| b/Å | 6.0777(2) |
| c/Å | 17.6985(7) |
| α/° | 90 |
| β/° | 100.556(2) |
| γ/° | 90 |
| Volume/Å$^3$ | 2898.45(19) |
| Z | 38 |
| $\rho_{calc}$g/cm$^3$ | 1.354 |
| μ/mm$^{-1}$ | 2.106 |
| F(000) | 1248.0 |
| Crystal size/mm$^3$ | 0.1 × 0.015 × 0.015 |
| Radiation | CuKα (λ = 1.54178) |
| 2Θ range for data collection/° | 5.078 to 132.858 |
| Index ranges | $-32 \leq h \leq 32$, $-6 \leq k \leq 7$, $-20 \leq l \leq 20$ |
| Reflections collected | 36336 |
| Independent reflections | 4980 [$R_{int}$ = 0.0329, $R_{sigma}$ = 0.0223] |
| Data/restraints/parameters | 4980/1/365 |
| Goodness-of-fit on $F^2$ | 1.019 |
| Final R indexes [I>=2σ (I)] | $R_1$ = 0.0227, $wR_2$ = 0.0593 |
| Final R indexes [all data] | $R_1$ = 0.0242, $wR_2$ = 0.0602 |
| Largest diff. peak/hole / e Å$^{-3}$ | 0.18/-0.26 |
| Flack parameter | 0.098(4) |

**Table 2 Fractional Atomic Coordinates (×10$^4$) and Equivalent Isotropic Displacement Parameters (Å$^2$×10$^3$) for S5. $U_{eq}$ is defined as 1/3 of the trace of the orthogonalised $U_{IJ}$ tensor.**

| Atom | *x* | *y* | *z* | U(eq) |
|---|---|---|---|---|
| S001 | 6254.9(2) | 1616.8(8) | 2525.1(3) | 32.12(13) |
| S002 | 8843.3(2) | 8825.7(9) | 2816.8(3) | 36.66(14) |
| O003 | 7426.2(5) | 6504(3) | 751.6(8) | 35.6(3) |
| O004 | 6841.1(5) | -1061(3) | 1928.4(8) | 33.4(3) |
| O005 | 8310.1(5) | 9417(2) | 4551.0(10) | 37.1(4) |
| O006 | 7481.9(5) | 6160(2) | 3731.5(9) | 38.5(4) |
| O007 | 8371.4(6) | 12564(3) | 2882.0(9) | 40.9(4) |
| O008 | 8089.1(5) | 5151(2) | 4699.9(8) | 35.2(3) |
| O009 | 6476.3(6) | 2683(3) | 485.5(10) | 42.3(4) |
| O00A | 7396.4(7) | 3282(3) | 1364.1(10) | 49.6(4) |
| N00B | 7558.8(6) | 2622(3) | 4013.4(10) | 30.1(4) |
| N00C | 7919.3(7) | 6072(3) | 1870.2(10) | 33.7(4) |
| C00D | 9115.4(7) | 9745(3) | 5377.8(12) | 29.2(4) |
| C00E | 7572.5(8) | 5124(4) | 1343.3(13) | 33.4(5) |
| C00F | 7741.4(8) | 4637(3) | 4196.1(12) | 29.1(4) |
| C00G | 6453.1(7) | -76(3) | 1821.6(11) | 27.3(4) |
| C00H | 8653.3(7) | 11266(4) | 3244.2(11) | 30.2(4) |
| C00I | 7975.5(7) | 8395(3) | 1726.2(11) | 26.9(4) |
| C00J | 7198.2(7) | 2626(3) | 3301.1(11) | 27.3(4) |
| C00K | 8894.5(7) | 11632(4) | 4078.0(11) | 29.4(4) |
| C00L | 9445.0(8) | 8083(4) | 5670.8(12) | 34.2(5) |
| C00M | 8830.2(7) | 9600(3) | 4564.8(12) | 29.6(4) |
| C00N | 6305.2(8) | 466(4) | 397.5(12) | 31.0(4) |
| C00O | 6748.0(7) | 1205(4) | 3347.7(11) | 31.5(4) |
| C00P | 5585.8(8) | 2014(4) | -537.3(13) | 38.8(5) |
| C00Q | 6069.9(7) | -213(3) | 1087.8(11) | 29.2(4) |
| C00R | 5933.1(7) | 338(4) | -342.4(12) | 32.6(5) |
| C00S | 7076.1(8) | 5082(4) | 3234.1(14) | 35.8(5) |
| C00T | 9315.8(8) | 11605(5) | 6594.7(12) | 42.0(5) |
| C00U | 9052.9(8) | 11509(4) | 5849.4(12) | 36.2(5) |
| C00V | 5857.0(10) | -2548(4) | 1022.6(14) | 45.0(6) |
| C00W | 9435.2(8) | 12349(4) | 4121.5(13) | 38.1(5) |
| C00X | 8511.1(8) | 9150(4) | 1840.9(11) | 34.5(5) |
| C00Y | 7721.0(8) | 8491(4) | 879.6(11) | 32.4(4) |
| C00Z | 9707.2(9) | 8183(4) | 6418.5(13) | 42.7(6) |
| C010 | 5238.1(8) | 1896(5) | -1212.2(14) | 46.6(6) |
| C011 | 9643.3(9) | 9949(5) | 6877.6(13) | 43.7(6) |
| C012 | 5922.3(9) | -1434(5) | -830.0(13) | 47.1(6) |

**Table 2 Fractional Atomic Coordinates (×10$^4$) and Equivalent Isotropic Displacement Parameters (Å$^2$×10$^3$) for S5. $U_{eq}$ is defined as 1/3 of the trace of the orthogonalised $U_{IJ}$ tensor.**

| Atom | *x* | *y* | *z* | U(eq) |
|---|---|---|---|---|
| C013 | 5576.2(10) | -1553(6) | -1508.0(14) | 57.7(7) |
| C014 | 5236.0(9) | 122(5) | -1697.8(14) | 52.3(7) |

**Table 3 Anisotropic Displacement Parameters (Å$^2$×10$^3$) for S5. The Anisotropic displacement factor exponent takes the form: $-2\pi^2[h^2a^{*2}U_{11}+2hka^*b^*U_{12}+\ldots]$.**

| Atom | $U_{11}$ | $U_{22}$ | $U_{33}$ | $U_{23}$ | $U_{13}$ | $U_{12}$ |
|---|---|---|---|---|---|---|
| S001 | 29.8(2) | 34.3(3) | 32.0(3) | -6.7(2) | 4.99(19) | 1.0(2) |
| S002 | 32.9(3) | 38.6(3) | 34.3(3) | -6.8(2) | -4.7(2) | 3.3(2) |
| O003 | 41.9(8) | 31.7(8) | 28.6(7) | -3.7(6) | -5.3(6) | -2.9(7) |
| O004 | 34.0(8) | 33.9(8) | 31.3(7) | -0.4(6) | 3.6(6) | 6.7(7) |
| O005 | 28.8(8) | 26.2(8) | 52.0(9) | 6.5(6) | -4.0(7) | -3.0(6) |
| O006 | 36.1(8) | 22.7(8) | 51.8(9) | 1.5(6) | -4.5(7) | -0.5(6) |
| O007 | 48.5(9) | 33.2(8) | 36.5(8) | 6.5(7) | -3.9(7) | 5.6(7) |
| O008 | 37.9(8) | 28.1(8) | 36.7(8) | -4.7(6) | -1.3(7) | -1.5(6) |
| O009 | 39.4(9) | 39.4(9) | 44.7(9) | 5.8(7) | -1.5(7) | -10.8(7) |
| O00A | 51.6(10) | 29.9(9) | 59.8(11) | 0.4(7) | -9.9(8) | -9.6(7) |
| N00B | 35.4(10) | 21.8(8) | 30.8(9) | 1.4(7) | 0.2(7) | 1.0(7) |
| N00C | 35.2(9) | 27.4(10) | 33.4(9) | 6.1(7) | -7.1(7) | -2.8(7) |
| C00D | 24.9(10) | 31.1(11) | 31.2(10) | 1.9(8) | 3.8(8) | -1.9(8) |
| C00E | 34.3(11) | 27.9(12) | 36.3(11) | -2.6(8) | 1.8(9) | 0.5(9) |
| C00F | 32.0(11) | 26.0(11) | 30.3(10) | -0.7(8) | 8.1(9) | 2.0(8) |
| C00G | 30.2(10) | 23.8(10) | 28.6(10) | 1.0(8) | 6.9(8) | -2.3(8) |
| C00H | 27.5(10) | 29.5(11) | 32.4(10) | 2.3(9) | 1.9(8) | -4.7(8) |
| C00I | 29.6(10) | 25.7(11) | 24.4(9) | -0.3(7) | 2.2(8) | -0.9(8) |
| C00J | 29.4(10) | 25.6(10) | 26.1(10) | 0.2(7) | 3.3(8) | 1.9(8) |
| C00K | 28.6(10) | 26.5(10) | 31.8(10) | -0.5(9) | 1.8(8) | 2.3(9) |
| C00L | 35.9(12) | 30.6(12) | 35.0(11) | 4.6(9) | 3.8(9) | 0.4(9) |
| C00M | 28.6(10) | 25.2(10) | 32.2(10) | -1.2(8) | -1.5(8) | 2.6(8) |
| C00N | 28.2(10) | 33.4(11) | 30.7(11) | -2.8(8) | 3.4(8) | 0.4(9) |
| C00O | 37.9(11) | 29.6(11) | 27.0(9) | -3.1(8) | 6.1(8) | -4.1(9) |
| C00P | 36.8(12) | 41.3(14) | 37.8(12) | 6.6(10) | 6.0(9) | -0.6(10) |
| C00Q | 28.1(10) | 30.5(11) | 28.4(10) | -2.1(8) | 3.4(8) | 0.1(8) |
| C00R | 27.5(10) | 42.7(13) | 27.9(10) | 1.0(9) | 5.6(8) | -1.2(9) |
| C00S | 30.4(11) | 27.7(12) | 45.8(13) | 4.6(9) | -2.0(9) | 1.7(9) |
| C00T | 45.2(12) | 50.3(14) | 32.2(11) | -7.5(10) | 11.9(9) | -8.7(11) |
| C00U | 34.2(11) | 38.9(12) | 35.6(11) | -2.3(10) | 7.2(9) | 2.9(10) |

**Table 3 Anisotropic Displacement Parameters (Å$^2$×10$^3$) for S5. The Anisotropic displacement factor exponent takes the form: $-2\pi^2[h^2a^{*2}U_{11}+2hka^*b^*U_{12}+\ldots]$.**

| Atom | $U_{11}$ | $U_{22}$ | $U_{33}$ | $U_{23}$ | $U_{13}$ | $U_{12}$ |
|---|---|---|---|---|---|---|
| C00V | 49.9(14) | 41.8(13) | 41.1(13) | -2.0(10) | 2.5(11) | -15.3(11) |
| C00W | 32.4(11) | 45.2(14) | 35.0(12) | -1.7(9) | 1.6(9) | -5.8(9) |
| C00X | 32.3(11) | 43.1(13) | 27.5(10) | -2.1(9) | 4.1(8) | -7.1(9) |
| C00Y | 38.1(11) | 29.7(11) | 27.3(10) | 2.5(8) | 0.0(8) | -2.5(9) |
| C00Z | 36.6(12) | 48.2(15) | 40.1(12) | 17.2(10) | -1.0(10) | -1.8(10) |
| C010 | 33.4(12) | 58.3(16) | 45.4(13) | 16.6(12) | 0.2(10) | 0.1(11) |
| C011 | 40.0(13) | 63.5(17) | 25.6(11) | 7.2(10) | 0.6(9) | -15.7(11) |
| C012 | 39.7(13) | 61.4(17) | 37.7(12) | -12.4(12) | 0.2(10) | 10.3(12) |
| C013 | 50.5(15) | 80(2) | 38.4(13) | -18.0(14) | -2.9(11) | 1.3(14) |
| C014 | 39.2(13) | 83(2) | 30.6(12) | 8.0(13) | -4.5(10) | -8.9(13) |

**Table 4 Bond Lengths for S5.**

| Atom | Atom | Length/Å | Atom | Atom | Length/Å |
|---|---|---|---|---|---|
| S001 | C00G | 1.775(2) | C00G | C00Q | 1.515(3) |
| S001 | C00O | 1.813(2) | C00H | C00K | 1.519(3) |
| S002 | C00H | 1.786(2) | C00I | C00X | 1.516(3) |
| S002 | C00X | 1.810(2) | C00I | C00Y | 1.534(3) |
| O003 | C00E | 1.344(3) | C00J | C00O | 1.521(3) |
| O003 | C00Y | 1.448(3) | C00J | C00S | 1.530(3) |
| O004 | C00G | 1.205(2) | C00K | C00M | 1.534(3) |
| O005 | C00M | 1.426(2) | C00K | C00W | 1.533(3) |
| O006 | C00F | 1.351(3) | C00L | C00Z | 1.387(3) |
| O006 | C00S | 1.443(3) | C00N | C00Q | 1.539(3) |
| O007 | C00H | 1.203(3) | C00N | C00R | 1.507(3) |
| O008 | C00F | 1.220(3) | C00P | C00R | 1.394(3) |
| O009 | C00N | 1.426(3) | C00P | C010 | 1.387(3) |
| O00A | C00E | 1.222(3) | C00Q | C00V | 1.531(3) |
| N00B | C00F | 1.340(3) | C00R | C012 | 1.377(3) |
| N00B | C00J | 1.452(3) | C00T | C00U | 1.384(3) |
| N00C | C00E | 1.334(3) | C00T | C011 | 1.380(4) |
| N00C | C00I | 1.448(3) | C00Z | C011 | 1.376(4) |
| C00D | C00L | 1.391(3) | C010 | C014 | 1.378(4) |
| C00D | C00M | 1.509(3) | C012 | C013 | 1.389(3) |
| C00D | C00U | 1.388(3) | C013 | C014 | 1.380(4) |

**Table 5 Bond Angles for S5.**

| Atom | Atom | Atom | Angle/° | Atom | Atom | Atom | Angle/° |
|---|---|---|---|---|---|---|---|
| C00G | S001 | C00O | 101.80(10) | C00H | C00K | C00W | 109.58(16) |
| C00H | S002 | C00X | 100.32(10) | C00M | C00K | C00W | 114.20(17) |
| C00E | O003 | C00Y | 108.68(15) | C00Z | C00L | C00D | 120.8(2) |
| C00F | O006 | C00S | 108.75(15) | O005 | C00M | C00D | 111.26(17) |
| C00F | N00B | C00J | 111.90(17) | O005 | C00M | C00K | 105.69(15) |
| C00E | N00C | C00I | 112.53(18) | C00D | C00M | C00K | 113.30(16) |
| C00L | C00D | C00M | 120.05(19) | O009 | C00N | C00Q | 110.11(17) |
| C00U | C00D | C00L | 118.7(2) | O009 | C00N | C00R | 107.47(18) |
| C00U | C00D | C00M | 121.27(19) | C00R | C00N | C00Q | 111.14(16) |
| O00A | C00E | O003 | 122.1(2) | C00J | C00O | S001 | 111.78(14) |
| O00A | C00E | N00C | 127.5(2) | C010 | C00P | C00R | 120.4(2) |
| N00C | C00E | O003 | 110.35(19) | C00G | C00Q | C00N | 109.90(16) |
| O008 | C00F | O006 | 121.51(19) | C00G | C00Q | C00V | 108.06(17) |
| O008 | C00F | N00B | 128.16(19) | C00V | C00Q | C00N | 113.46(18) |
| N00B | C00F | O006 | 110.33(18) | C00P | C00R | C00N | 120.0(2) |
| O004 | C00G | S001 | 123.55(15) | C012 | C00R | C00N | 121.2(2) |
| O004 | C00G | C00Q | 124.09(17) | C012 | C00R | C00P | 118.8(2) |
| C00Q | C00G | S001 | 112.32(14) | O006 | C00S | C00J | 105.21(16) |
| O007 | C00H | S002 | 121.82(16) | C011 | C00T | C00U | 120.4(2) |
| O007 | C00H | C00K | 123.0(2) | C00T | C00U | C00D | 120.4(2) |
| C00K | C00H | S002 | 115.12(14) | C00I | C00X | S002 | 113.61(14) |
| N00C | C00I | C00X | 113.78(18) | O003 | C00Y | C00I | 105.18(16) |
| N00C | C00I | C00Y | 99.51(16) | C011 | C00Z | C00L | 119.9(2) |
| C00X | C00I | C00Y | 112.04(16) | C014 | C010 | C00P | 120.1(2) |
| N00B | C00J | C00O | 112.45(16) | C00Z | C011 | C00T | 119.9(2) |
| N00B | C00J | C00S | 99.97(17) | C00R | C012 | C013 | 121.0(2) |
| C00O | C00J | C00S | 112.95(17) | C014 | C013 | C012 | 119.7(3) |
| C00H | C00K | C00M | 110.69(17) | C010 | C014 | C013 | 120.0(2) |

**Table 6 Torsion Angles for S5.**

| A | B | C | D | Angle/° | A | B | C | D | Angle/° |
|---|---|---|---|---|---|---|---|---|---|
| S001 | C00G | C00Q | C00N | 124.99(15) | C00L | C00Z | C011 | C00T | 0.5(3) |
| S001 | C00G | C00Q | C00V | -110.72(17) | C00M | C00D | C00L | C00Z | -179.51(19) |
| S002 | C00H | C00K | C00M | -53.12(19) | C00M | C00D | C00U | C00T | 179.62(19) |
| S002 | C00H | C00K | C00W | 73.7(2) | C00N | C00R | C012 | C013 | 179.5(2) |
| O004 | C00G | C00Q | C00N | -57.4(3) | C00O | S001 | C00G | O004 | -3.7(2) |
| O004 | C00G | C00Q | C00V | 66.9(3) | C00O | S001 | C00G | C00Q | 173.93(14) |
| O007 | C00H | C00K | C00M | 130.1(2) | C00O | C00J | C00S | O006 | 138.42(16) |

**Table 6 Torsion Angles for S5.**

| A | B | C | D | Angle/° | A | B | C | D | Angle/° |
|---|---|---|---|---|---|---|---|---|---|
| O007 | C00H | C00K | C00W | -103.1(2) | C00P | C00R | C012 | C013 | 0.6(4) |
| O009 | C00N | C00Q | C00G | -61.3(2) | C00P | C010 | C014 | C013 | 0.8(4) |
| O009 | C00N | C00Q | C00V | 177.65(18) | C00Q | C00N | C00R | C00P | 79.0(2) |
| O009 | C00N | C00R | C00P | -41.6(2) | C00Q | C00N | C00R | C012 | -99.9(2) |
| O009 | C00N | C00R | C012 | 139.6(2) | C00R | C00N | C00Q | C00G | 179.77(17) |
| N00B | C00J | C00O | S001 | 169.55(13) | C00R | C00N | C00Q | C00V | 58.7(2) |
| N00B | C00J | C00S | O006 | 18.7(2) | C00R | C00P | C010 | C014 | -0.4(3) |
| N00C | C00I | C00X | S002 | -62.7(2) | C00R | C012 | C013 | C014 | -0.2(4) |
| N00C | C00I | C00Y | O003 | 18.3(2) | C00S | O006 | C00F | O008 | -176.08(18) |
| C00D | C00L | C00Z | C011 | -0.3(3) | C00S | O006 | C00F | N00B | 3.9(2) |
| C00E | O003 | C00Y | C00I | -14.2(2) | C00S | C00J | C00O | S001 | 57.3(2) |
| C00E | N00C | C00I | C00X | -137.02(18) | C00U | C00D | C00L | C00Z | -0.1(3) |
| C00E | N00C | C00I | C00Y | -17.7(2) | C00U | C00D | C00M | O005 | -63.9(2) |
| C00F | O006 | C00S | C00J | -14.8(2) | C00U | C00D | C00M | C00K | 55.0(3) |
| C00F | N00B | C00J | C00O | -137.71(18) | C00U | C00T | C011 | C00Z | -0.4(3) |
| C00F | N00B | C00J | C00S | -17.6(2) | C00W | C00K | C00M | O005 | 170.69(17) |
| C00G | S001 | C00O | C00J | 81.11(16) | C00W | C00K | C00M | C00D | 48.6(2) |
| C00H | S002 | C00X | C00I | -82.74(17) | C00X | S002 | C00H | O007 | 0.7(2) |
| C00H | C00K | C00M | O005 | -65.1(2) | C00X | S002 | C00H | C00K | -176.08(14) |
| C00H | C00K | C00M | C00D | 172.85(16) | C00X | C00I | C00Y | O003 | 138.92(18) |
| C00I | N00C | C00E | O003 | 10.1(2) | C00Y | O003 | C00E | O00A | -177.0(2) |
| C00I | N00C | C00E | O00A | -169.5(2) | C00Y | O003 | C00E | N00C | 3.3(2) |
| C00J | N00B | C00F | O006 | 9.7(2) | C00Y | C00I | C00X | S002 | -174.62(15) |
| C00J | N00B | C00F | O008 | -170.38(19) | C010 | C00P | C00R | C00N | -179.18(19) |
| C00L | C00D | C00M | O005 | 115.5(2) | C010 | C00P | C00R | C012 | -0.3(3) |
| C00L | C00D | C00M | C00K | -125.6(2) | C011 | C00T | C00U | C00D | 0.0(3) |
| C00L | C00D | C00U | C00T | 0.2(3) | C012 | C013 | C014 | C010 | -0.5(4) |

**Table 7 Hydrogen Atom Coordinates (Å×$10^4$) and Isotropic Displacement Parameters (Å$^2$×$10^3$) for S5.**

| Atom | *x* | *y* | *z* | U(eq) |
|---|---|---|---|---|
| H005 | 8238.15 | 8123.35 | 4603.39 | 56 |
| H009 | 6750.6 | 2712.43 | 763.74 | 64 |
| H00B | 7644.71 | 1465.84 | 4285.23 | 36 |
| H00I | 8090.58 | 5379.95 | 2252.88 | 40 |
| H00K | 7790.67 | 9273.42 | 2043.08 | 32 |
| H00J | 7354.05 | 2150.49 | 2872.32 | 33 |
| H00L | 8719.37 | 12853.25 | 4272.56 | 35 |

**Table 7 Hydrogen Atom Coordinates (Å×10$^4$) and Isotropic Displacement Parameters (Å$^2$×10$^3$) for S5.**

| Atom | *x* | *y* | *z* | U(eq) |
|---|---|---|---|---|
| H00M | 9490.13 | 6890.42 | 5361.72 | 41 |
| H00O | 8941.96 | 8297.22 | 4317.99 | 35 |
| H00N | 6585.64 | -507.98 | 365.34 | 37 |
| H00A | 6625.89 | 1560.4 | 3814.05 | 38 |
| H00C | 6845.54 | -330.91 | 3374.28 | 38 |
| H00P | 5587.25 | 3218.33 | -212.73 | 47 |
| H00Q | 5800.78 | 817.73 | 1127.49 | 35 |
| H00D | 7052.16 | 5578.93 | 2707.71 | 43 |
| H00E | 6764.27 | 5383.81 | 3399.2 | 43 |
| H00T | 9271.55 | 12791.93 | 6906.79 | 50 |
| H00U | 8833.09 | 12631.87 | 5663.25 | 43 |
| H00F | 6106.79 | -3560.48 | 923.99 | 68 |
| H00G | 5577.13 | -2608.34 | 608.19 | 68 |
| H00H | 5753.63 | -2939.27 | 1494.6 | 68 |
| H00R | 9627.86 | 11119.87 | 4003 | 57 |
| H00S | 9566.55 | 12866.89 | 4630.55 | 57 |
| H00V | 9449.65 | 13509.21 | 3758.19 | 57 |
| H00W | 8518.5 | 10688.73 | 1698.02 | 41 |
| H00X | 8681.13 | 8319.14 | 1499.32 | 41 |
| H00Y | 7511.73 | 9785.14 | 781.77 | 39 |
| H | 7965.26 | 8531.86 | 546.53 | 39 |
| H00Z | 9925.68 | 7060.06 | 6609.38 | 51 |
| H010 | 5006.1 | 3015.77 | -1337.22 | 56 |
| H011 | 9820.69 | 10025.42 | 7377.96 | 52 |
| H012 | 6150.41 | -2567.7 | -703.27 | 57 |
| H013 | 5573.69 | -2757.66 | -1832.83 | 69 |
| H014 | 5005.3 | 54.27 | -2153.38 | 63 |

CCDC 2286538 contains the supplementary crystallographic data for this paper, including structure factors and refinement instructions. These data can be obtained free of charge from The Cambridge Crystallographic Data Centre, 12 Union Road.

## References

(1) Nanda, J.; Adhikari, B.; Basak, S.; Banerjee, A. Formation of hybrid hydrogels consisting of tripeptide and different silver nanoparticle-capped ligands: modulation of the mechanical strength of gel phase materials. *J. Phys. Chem. B* **2012**, *116* (40), 12235-12244. DOI: https://doi.org/10.1021/jp306262t.

(2) Sarabia, F.; Vivar-García, C.; García-Castro, M.; García-Ruiz, C.; Martín-Gálvez, F.; Sánchez-Ruiz, A.; Chammaa, S. A highly stereoselective synthesis of glycidic amides based on a new class of chiral sulfonium salts: Applications in asymmetric synthesis. *Eur. J. Chem.* **2012**, *18* (47), 15190-15201. DOI: https://doi.org/10.1002/chem.201201332.

(3) Chouhan, G.; Alper, H. Synthesis of Ring-Fused Oxazolo- and Pyrazoloisoquinolinones by a One-Pot Pd-Catalyzed Carboxamidation and Aldol-Type Condensation Cascade Process. *J. Org. Chem.* **2009**, *74* (16), 6181-6189. DOI: https://doi.org/10.1021/jo9010574.

(4) Evans, D. A.; Tedrow, J. S.; Shaw, J. T.; Downey, C. W. Diastereoselective magnesium halide-catalyzed anti-aldol reactions of chiral N-acyloxazolidinones. *J. Am. Chem. Soc.* **2002**, *124* (3), 392-393. DOI: https://doi.org/10.1021/ja0119548.

(5) Inada, H.; Shibuya, M.; Yamamoto, Y. Direct Synthesis of Free α-Amino Acids by Telescoping Three-Step Process from 1,2-Diols. *Org. Lett.* **2019**, *21* (3), 709-713. DOI: https://doi.org/10.1021/acs.orglett.8b03910.

(6) Thottumkara, A. P.; Kurokawa, T.; Du Bois, J. Carbocyclization of unsaturated thioesters under palladium catalysis. *Chem. Sci.* **2013**, *4* (6), 2686-2689. DOI: https://doi.org/10.1039/C3SC50486G.

(7) Huffman, B. J.; Chu, T.; Hanaki, Y.; Wong, J. J.; Chen, S.; Houk, K. N.; Shenvi, R. A. Stereodivergent Attached-Ring Synthesis via Non-Covalent Interactions: A Short Formal Synthesis of Merrilactone A. *Angew. Chem., Int. Ed.* **2022**, *61* (3), e202114514. DOI: https://doi.org/10.1002/anie.202114514.

(8) Liu, M.; Liu, Y.-W.; Xu, H.; Dai, H.-X. Palladium-catalyzed intramolecular CH acylation of indoles with thioester. *Tetrahedron Lett.* **2019**, *60* (39), 151061. DOI: https://doi.org/10.1016/j.tetlet.2019.151061.

(9) Kawakami, T.; Aimoto, S. The use of a cysteinyl prolyl ester (CPE) autoactivating unit in peptide ligation reactions. *Tetrahedron* **2009**, *65* (19), 3871-3877. DOI: https://doi.org/10.1016/j.tet.2009.03.008.

(10) Zheng, J.-S.; Chen, X.; Tang, S.; Chang, H.-N.; Wang, F.-L.; Zuo, C. A new method for synthesis of peptide thioesters via irreversible N-to-S acyl transfer. *Org. Lett.* **2014**, *16* (18), 4908-4911. DOI: https://doi.org/10.1021/ol5024213.

(11) Kawakami, T.; Sumida, M.; Vorherr, T.; Aimoto, S. Peptide thioester preparation based on an NS acyl shift reaction mediated by a thiol ligation auxiliary. *Tetrahedron Lett.* **2005**, *46* (50), 8805-8807. DOI: https://doi.org/10.1016/j.tetlet.2005.09.184.

(12) Ollivier, N.; Behr, J.-B.; El-Mahdi, O.; Blanpain, A.; Melnyk, O. Fmoc solid-phase synthesis of peptide thioesters using an intramolecular N, S-acyl shift. *Org. Lett.* **2005**, *7* (13), 2647-2650. DOI: https://doi.org/10.1021/ol050776a.

(13) Tsuda, S.; Shigenaga, A.; Bando, K.; Otaka, A. N→ S acyl-transfer-mediated synthesis of peptide thioesters using anilide derivatives. *Org. Lett.* **2009**, *11* (4), 823-826. DOI: https://doi.org/10.1021/ol8028093.

(14) Ohta, Y.; Itoh, S.; Shigenaga, A.; Shintaku, S.; Fujii, N.; Otaka, A. Cysteine-derived S-protected oxazolidinones: potential chemical devices for the preparation of peptide thioesters. *Org. Lett.* **2006**, *8* (3), 467-470. DOI: https://doi.org/10.1021/ol052755m.
(15) de Duve, C.; Bracher, P.; Snyder, P.; Bohall, B.; Whitesides, G.; Dawson, P.; Muir, T.; Clark-Lewis, I.; Kent, S.; Wieland, T. Going round in circles with N→ S acyl transfer. *Synlett* **2017**, *28* (13), 1517-1529. DOI: 10.1055/s-0036-1588789.
(16) Koch, A. A.; Hansen, D. A.; Shende, V. V.; Furan, L. R.; Houk, K.; Jiménez-Osés, G.; Sherman, D. H. A single active site mutation in the pikromycin thioesterase generates a more effective macrocyclization catalyst. *J. Am. Chem. Soc.* **2017**, *139* (38), 13456-13465. DOI: https://doi.org/10.1021/jacs.7b06436.
(17) Koch, A. A.; Schmidt, J. J.; Lowell, A. N.; Hansen, D. A.; Coburn, K. M.; Chemler, J. A.; Sherman, D. H. Probing selectivity and creating structural diversity through hybrid polyketide synthases. *Angew. Chem.* **2020**, *132* (32), 13677-13682. DOI: https://doi.org/10.1002/ange.202004991.
(18) Kim, B. H.; Lee, H. B.; Hwang, J. K.; Kim, Y. G. Asymmetric induction in the conjugate addition of thioacetic acid to methacrylamides with chiral auxiliaries. *Tetrahedron: Asymmetry* **2005**, *16* (6), 1215-1220. DOI: https://doi.org/10.1016/j.tetasy.2005.01.037.
(19) Tomioka, K.; Suenaga, T.; Koga, K. Asymmetric conjugate addition reaction by the use of (S)-γ-trityloxymethyl-γ-butyrolactam as a chiral auxiliary. *Tetrahedron Lett.* **1986**, *27* (3), 369-372. DOI: https://doi.org/10.1016/S0040-4039(00)84021-6.
(20) Fleming, I.; Kindon, N. D. Diastereoselectivity in the preparation of β-silyl esters from αβ-unsaturated esters and amides attached to chiral auxiliaries. *J. Chem. Soc., Perkin trans. 1* **1995**, (4), 303-315. DOI: https://doi.org/10.1039/C39870001177.
(21) Myers, A. G.; Gleason, J. L.; Yoon, T.; Kung, D. W. Highly Practical Methodology for the Synthesis of d- and l-α-Amino Acids, N-Protected α-Amino Acids, and N-Methyl-α-amino Acids. *J. Am. Chem. Soc.* **1997**, *119* (4), 656-673. DOI: https://doi.org/10.1021/ja9624073.
(22) Seiple, I. B.; Mercer, J. A.; Sussman, R. J.; Zhang, Z.; Myers, A. G. Stereocontrolled Synthesis of syn-β-Hydroxy-α-Amino Acids by Direct Aldolization of Pseudoephenamine Glycinamide. *Angew. Chem., Int. Ed.* **2014**, *53* (18), 4642-4647. DOI: https://doi.org/10.1002/anie.201400928.
(23) Evans, D.; Takacs, J. Enantioselective alkylation of chiral enolates. *Tetrahedron Lett.* **1980**, *21* (44), 4233-4236. DOI: https://doi.org/10.1016/S0040-4039(00)92870-3.
(24) Hansen, D. A.; Rath, C. M.; Eisman, E. B.; Narayan, A. R. H.; Kittendorf, J. D.; Mortison, J. D.; Yoon, Y. J.; Sherman, D. H. Biocatalytic Synthesis of Pikromycin, Methymycin, Neomethymycin, Novamethymycin, and Ketomethymycin. *J. Am. Chem. Soc.* **2013**, *135* (30), 11232-11238. DOI: https://doi.org/10.1021/ja404134f.
(25) Huther, N.; Beland, F.; Potvin, S.; Tremblay, L. SiliaBond TMA acetate: A new strong anion exchange sorbent and its applications in SPE format. In *ABSTRACTS OF PAPERS OF THE AMERICAN CHEMICAL SOCIETY*, 2009; AMER CHEMICAL SOC 1155 16TH ST, NW, WASHINGTON, DC 20036 USA: Vol. 238.
(26) House, H. O.; Chu, C.-Y.; Wilkins, J. M.; Umen, M. J. Chemistry of carbanions. XXVII. Convenient precursor for the generation of lithium organocuprates. *J. Org. Chem.* **1975**, *40* (10), 1460-1469. DOI: https://doi.org/10.1021/jo00898a019.
(27) Evans, D.; Nelson, J.; Vogel, E.; Taber, T. Stereoselective aldol condensations via boron enolates. *J. Am. Chem. Soc.* **1981**, *103* (11), 3099-3111. DOI: https://doi.org/10.1021/ja00401a031.

(28) Allred, G. D.; Liebeskind, L. S. Copper-mediated cross-coupling of organostannanes with organic iodides at or below room temperature. *J. Am. Chem. Soc.* **1996**, *118* (11), 2748-2749. DOI: https://doi.org/10.1021/ja9541239.
(29) Lee, S.; Love, M. S.; Modukuri, R.; Chatterjee, A. K.; Huerta, L.; Lawson, A. P.; McNamara, C. W.; Mead, J. R.; Hedstrom, L.; Cuny, G. D. Structure-activity relationship of BMS906024 derivatives for Cryptosporidium parvum growth inhibition. *Bioorg. Med. Chem. Lett.* **2023**, *90*, 129328. DOI: https://doi.org/10.1016/j.bmcl.2023.129328.
(30) Papa, A. J. Synthesis and azidolysis of 2-chlorotetramethylguanidine. Synthetic utility of hexa-and tetramethylguanidinium azide. *J. Org. Chem.* **1966**, *31* (5), 1426-1430. DOI: https://doi.org/10.1021/jo01343a026.
(31) Ludin, D. V.; Zaitsev, S. D.; Markin, A. V.; Grishin, I. D.; Sologubov, S. S.; Kovylina, T. A.; Fedushkin, I. L. New method for controlled synthesis of polylactide block copolymers: organoborane/p-quinone system and reversible-deactivation radical polymerization. *Polym. Int.* **2022**, *71* (1), 86-97. DOI: https://doi.org/10.1002/pi.6287.
(32) Tokuyama, H.; Yokoshima, S.; Yamashita, T.; Fukuyama, T. A novel ketone synthesis by a palladium-catalyzed reaction of thiol esters and organozinc reagents. *Tetrahedron Lett.* **1998**, *39* (20), 3189-3192. DOI: https://doi.org/10.1016/S0040-4039(98)00456-0.
(33) Petra, D. G.; Kamer, P. C.; Spek, A. L.; Schoemaker, H. E.; van Leeuwen, P. W. Aminosulf(ox)ides as ligands for iridium (I)-catalyzed asymmetric transfer hydrogenation. *J. Org. Chem.* **2000**, *65* (10), 3010-3017. DOI: https://doi.org/10.1021/jo991700t.
(34) Shi, S. L.; Kanai, M.; Shibasaki, M. Asymmetric synthesis of dihydropyranones from ynones by sequential copper (I)-catalyzed direct aldol and silver (I)-catalyzed oxy-Michael reactions. *Angew. Chem., Int. Ed.* **2012**, *51* (16), 3932-3935. DOI: https://doi.org/10.1002/anie.201109209
(35) Bohman, B.; Tan, M. M.; Phillips, R. D.; Scaffidi, A.; Sobolev, A. N.; Moggach, S. A.; Flematti, G. R.; Peakall, R. A specific blend of drakolide and hydroxymethylpyrazines: An unusual pollinator sexual attractant used by the endangered orchid Drakaea micrantha. *Angew. Chem.* **2020**, *132* (3), 1140-1144. DOI: https://doi.org/10.1002/ange.201911636.
(36) Oh, H.-S.; Yun, J.-S.; Nah, K.-H.; Kang, H.-Y.; Sherman, D. H. Synthesis of the Tetraketide Lactones from the Pikromycin Biosynthetic Pathway. *Eur. J. Org. Chem.* **2007**, *2007* (20), 3369-3379. DOI: https://doi.org/10.1002/ejoc.200700254.
